# Hepatic Ischemia-Reperfusion Impairs Blood-Brain Barrier Partly Due to Release of Arginase From Injured Liver

**DOI:** 10.3389/fphar.2021.724471

**Published:** 2021-10-13

**Authors:** Liang Zhu, Han Zhou, Feng Xu, Hanyu Yang, Ping Li, Yun Sheng, Peihua Liu, Weimin Kong, Xiaonan Liu, Lu Yang, Li Liu, Xiaodong Liu

**Affiliations:** Center of Drug Metabolism and Pharmacokinetics, School of Pharmacy, China Pharmaceutical University, Nanjing, China

**Keywords:** hepatic ischemia-reperfusion, blood-brain barrier, arginase, arginine deficiency, cell proliferation, cell cycle

## Abstract

**Aim:** Hepatic ischemia-reperfusion (HIR) induces remote organs injury, including the brain. The homeostasis of the brain is maintained by the blood-brain barrier (BBB); thus, we aimed to investigate whether HIR impaired BBB and attempted to elucidate its underlying mechanism.

**Methods:** Cell viability of human cerebral microvascular endothelial cells (hCMEC/D3) was measured following 24 h incubation with a serum of HIR rat undergoing 1 h ischemia and 4 h reperfusion, liver homogenate, or lysate of primary hepatocytes of the rat. The liver homogenate was precipitated using (NH_4_)_2_SO_4_ followed by separation on three columns and electrophoresis to identify the toxic molecule. Cell activity, apoptosis, proliferation, cell cycle, and expressions of proteins related to cell cycle were measured in hCMEC/D3 cells incubated with identified toxic molecules. HIR rats undergoing 1 h ischemia and 24 h reperfusion were developed to determine the release of an identified toxic molecule. BBB function was indexed as permeability to fluorescein and brain water. Endothelial cell proliferation and expressions of proteins related to the cell cycle in cerebral microvessels were measured by immunofluorescence and western blot.

**Results:** Toxic molecule to BBB in the liver was identified to be arginase. Arginase inhibitor nor-NOHA efficiently attenuated hCMEC/D3 damage caused by liver homogenate and serum of HIR rats. Both arginase and serum of HIR rats significantly lowered arginine (Arg) in the culture medium. Arg addition efficiently attenuated the impairment of hCMEC/D3 caused by arginase or Arg deficiency, demonstrating that arginase impaired hCMEC/D3 *via* depriving Arg. Both arginase and Arg deficiency damaged hCMEC/D3 cells by inhibiting cell proliferation, retarding the cell cycle to G1 phase, and downregulating expressions of cyclin A, cyclin D, CDK2, and CDK4. HIR notably increased plasma arginase activity and lowered Arg level, increased the BBB permeability accompanied with enhanced brain water, and decreased the proliferative cells (marked by Ki67) in cerebral microvessels (marked by CD31) and protein expressions of cyclin A, cyclin D, CDK2 and CDK4 in isolated brain microvessels. Oral supplement of Arg remarkably attenuated these HIR-induced alterations.

**Conclusion:** HIR leads to substantial release of arginase from the injured liver and then deprives systemic Arg. The Arg deficiency further impairs BBB *via* inhibiting the proliferation of brain microvascular endothelial cells by cell cycle arrest.

## Introduction

Some liver surgical operations such as intrahepatic lesions, trauma surgery, and liver transplantation require a period of ischemia (including hepatic blood flow or total vascular occlusion) followed by restoration of blood flow. During the process, liver injury often occurs, which is termed as hepatic ischemia-reperfusion (HIR) injury (Cannistrà et al., 2016). The HIR also induces remote organ injury such as kidney, gut, and lung and myocardial, adrenal, and pancreatic injury (Nastos et al., 2014). More importantly, HIR is reported to affect the function of the central nervous system (CNS) and induce cognitive dysfunction (Wang et al., 2014; Ma et al., 2017). HIR impairs CNS function *via* activating different neuronal phenotypes in the rat brain (Bundzikova et al., 2011). A similar report has demonstrated the impairment in the passive avoidance test, accompanied by a decrease in turnover of norepinephrine and dopamine but a remarkable increase in the turnover of 5-hydroxytryptamine in the HIR rat brain (Adachi et al., 1998). In general, brain homeostasis is highly controlled by the blood-brain barrier. This indicates that impairment of CNS by HIR may result from brain dyshomeostasis due to alteration of BBB function. Our preliminary experiment data showed that HIR significantly increased fluorescein penetration into rat brain, demonstrating BBB impairment. Consistently, incubation with 10% serum of HIR rats and liver homogenate of rats *in vitro* both impaired cerebral microvascular endothelial cells, the structural component of BBB, indicating that some potential toxic molecules released from injured liver would impair BBB. Furthermore, extracted proteins from liver homogenate with cytotoxicity were further purified and identified to be arginase. Significantly increased activity of arginase was also observed in serum of HIR rats.

Several reports have shown the increased arginase in serum of patients with liver injury (Chrzanowska et al., 2014) and acute liver failure (ALF) animals (Sharma et al., 2012; Ozcelik et al., 2014). Arginase, highly expressed in the hepatocyte, converts arginine (Arg) into urea and ornithine (Caldwell et al., 2018). The release of arginase from the injured liver by HIR is speculated to deprive systemic Arg. Some diseases have demonstrated Arg deficiency due to the substantial release of arginase (Grasemann et al., 2006; Morris, 2012). More recently, Arg deprivation has been gradually demonstrated to play a role in several pathophysiological processes, including immunosuppression by inhibiting T cells to express the CD3ζ (Bronte and Zanovello, 2005; Peranzoni et al., 2007) and decreasing glucose uptake in the intestine by inhibiting GLUT2 (Xia et al., 2016) and cancer suppression (Glazer et al., 2011). These researches have suggested that a large release of arginase from the injured liver would lead to Arg deficiency in plasma and subsequently impair BBB.

The aim of this study was to 1) screen and identify the potential toxic molecule released from the injured liver under HIR that impairs BBB; 2) further investigate whether the toxic component is arginase and explore the underlying mechanisms using cerebral microvessel endothelial cells *in vitro*; 3) explore the role of substantially released arginase during HIR in BBB impairment using HIR rats and investigate whether the supplement of Arg could attenuate BBB impairment caused by HIR. The results would highlight the roles of the released arginase in BBB impairment under HIR.

## Materials and Methods

### Animals

Male Sprague-Dawley rats (aged 8∼9 weeks, weighing 220∼240 g), from Sino British Sippr Bk Lab Animal Co., Ltd. (Shanghai, China), were housed in constant room temperature and humidity on a normal 12 h light/dark cycle with free access to laboratory food and water. All animal studies were performed in accordance with the Guide for the Care and Use of Laboratory Animals (National Institutes of Health) and approved by the Animal Ethics Committee of China Pharmaceutical University.

### Preparations of Hepatic Ischemia-Reperfusion Serum, Liver Homogenate, and Hepatocytes Lysate

HIR rats were developed according to the method previously described (Spiegel and Bahde, 2006). Rats were anesthetized by intraperitoneal injection of pentobarbitone (40 mg/kg) followed by a midline laparotomy. The left branches of the hepatic artery, portal vein, and bile duct were occluded using a microvascular surgical clamp, which induced the blockage of 70% of blood supply to the liver. After 1 h of ischemia, the clamp was removed. Sham-operated (Sham) rats were treated in an identical manner with the omission of vascular occlusion. After 4 h reperfusion, serum was collected from the oculi chorioideae vein and inactivated by heating at 56°C for 30 min followed by sterilization *via* filtration through 0.22 μm. For another group, the rats intravenously received fluorescein (2 mg/kg) *via* tail vein and 45 min later, rats were sacrificed under slight ether anesthesia. Blood was collected for measuring plasma fluorescein. Then, rats were perfused transcardially with normal saline to remove any remaining fluorescein in the circulation and brain samples were obtained for measuring brain fluorescein as previously described (Zhou et al., 2019). BBB permeability was indexed as the ratio of brain to plasma fluorescein (Tsuneoka et al., 2021).

Liver and other tissues from healthy rats were homogenized (0.1 g/ml) in RPMI-1640 medium by Dounce tissue grinders in ice, followed by centrifugation at 16,000 g for 10 min at 4°C. The supernatant was filtered through 0.22 μm and proteins were quantified by BCA assay (Beyotime Biotechnology, Shanghai, China).

Primary hepatocytes from healthy rats prepared as previously described (Shu et al., 2016) were lysed in RPMI-1640 medium in ice by Vibra-Cell™ Ultrasonic Liquid Processors VCX 130 (Sonics & Materials, United States), followed by centrifugation at 16,000 g for 10 min at 4°C. The supernatant was filtered through 0.22 μm and proteins were quantified by BCA assay.

### Cell Culture and Viability Assay

hCMEC/D3 cell, an immortalized human cerebral microvascular endothelial cell, is widely utilized as an *in vitro* BBB model (Qosa et al., 2014; Sánchez-Dengra et al., 2021). It was purchased from FuHeng Cell Center (Shanghai, China) and cultured in RPMI-1640 medium (Invitrogen, Carlsbad, CA, United States) supplemented with 10% fetal bovine serum (FBS) (Invitrogen, Carlsbad, CA, United States) under 5% CO_2_ in an incubator (37°C) as previously described (Qin et al., 2020). For cell viability assay, hCMEC/D3 cells were seeded into 24-well plates at a density of 1×10^5^ cells per well. After 24 h, cells were treated with 10% serum from HIR rats, rat liver or other tissues homogenates (200 μg/ml), lysate of primary rat hepatocytes, S9 (supernatant of 9,000 g centrifugation) (Suling et al., 1983) of the human liver (Research Institute for Liver Diseases, Shanghai, China) (200 μg/ml) or other tested agents for 24 h. Cell viability was assessed using a CCK-8 kit (Beyotime Biotechnology, Shanghai, China) and the results were expressed as the fold of control.

### Screening and Identification of Toxic Molecule From Liver

Rat liver cytoplasm (about 1 g protein) was applied to 50∼65% saturated (NH_4_)_2_SO_4_ precipitation and the protein pellet (242 mg) was dissolved in buffer A (1 M (NH_4_)_2_SO_4_ in 50 mM phosphate buffer saline (PBS), pH 7.0) and applied to Butyl Purose 4 HP column (ion-exchange chromatography) (Qianchun Bio, Yancheng, China) with gradient elution using buffer B (50 mM PBS, pH 7.0) at 2 ml/min. The flow-through part in exchange for buffer A (20 mM PBS, pH = 5.8) was then subjected to CM Purose 6 FF (hydrophilic interaction chromatography) (Qianchun Bio, Yancheng, China) with gradient elution using buffer B (1 M NaCl in 20 mM PBS, pH = 5.8) at 1 ml/min. The active fractions were further fractionated with Sephacryl S-200 HR (gel filtration chromatography) at 3 ml/min. Five fractions (S1–S5) with high abundance were assayed for damage activity to hCMEC/D3 cell viability and applied to sodium dodecyl sulfate–polyacrylamide gel electrophoresis (SDS-PAGE), followed by Coomassie Brilliant Blue staining. The bands with differential activity were compared and then manually cut from the gel and digested to peptides by trypsin. Then, the peptides were separated by NanoLC using 0.1% formic acid and acetonitrile with 0.1% formic acid as mobile phases and analyzed by TripleTOF^®^ 5600+ (AB Sciex) mass spectrometer. TurboSEQUEST V2.7 software was used for data analysis.

### Permeability Assay of *In Vitro* Blood-Brain Barrier Model

hCMEC/D3 cells were seeded on Millicell Hanging Cell Culture Inserts (Millipore Corp, Billerica, MA, United States) at a density of 2 × 10^5^ cells per well. Following the formation of monolayer confluence, the cells were treated with a medium containing 20 μg/ml arginase for 24 h. Fluorescein (10 μg/ml) and FITC-dextran (3 kDa) (1 mg/ml) were added to luminal chamber. Concentrations of FITC-dextran and fluorescein in the receiver at designed times (1, 1.5, 2 h) were measured on SpectraMax Gemini XPS microplate fluorometer (Sunnyvale, CA, United States) at 430 nm (excitation) and 525 nm (emission) for fluorescein and 485 nm (excitation) and 525 nm (emission) for FITC-dextran. Apparent permeability coefficients (P_eff_) were calculated as previously described (Förster et al., 2008).

### Flow Cytometry

hCMEC/D3 cells were seeded into 6-well plates at a density of 3 × 10^5^ cells per well followed by treatment with 20 μg/ml arginase (Sigma-Aldrich, St. Louis, MO, United States) or Arg-free medium for 24 h. Then, the harvested cells were washed by PBS and applied to cell apoptosis, cell proliferation, and cell cycle assay using corresponding kits: Annexin V-FITC/PI kit (Yeasen Biotech, Shanghai, China), YF^®^488 Click-iT EdU Kit (US EVERBRIGHT INC., Suzhou, China), and PI staining kit (Yeasen Biotech, Shanghai, China) as previously described (Vermes et al., 1995; Park et al., 2018; Li et al., 2019). The samples were determined on an MACSQuant flow cytometer (Miltenyi Biotec, Germany) and data were analyzed by Flowjo 10.4 software.

### Role of Arginase in HIR-Induced Blood-Brain Barrier Impairment in Rats

To explore the involvement of liver-released arginase in BBB breakdown of HIR rats. The rats were grouped as Sham, HIR, and HIR supplemented with Arg (HIR + Arg). The surgical procedure followed the above description. HIR + Arg rats received 0.18 g/kg Arg orally (5 ml/kg) before surgery and at 2, 6, and 12 h of reperfusion. HIR rats and Sham rats received normal saline orally. Blood samples were collected from the oculi chorioideae vein before surgical operation and at 4, 8, 12, and 24 h of reperfusion to determine plasma arginase activity using an assay kit (Sigma-Aldrich, St. Louis, MO, United States) and Arg level. At 24 h of reperfusion, fluorescein (2 mg/kg) was injected into rats to detect BBB permeability. After 45 min, the rats were sacrificed under slight ether anesthesia and blood was collected for measuring plasma fluorescein and serum biochemical parameters related to liver function, including alanine transaminase (ALT), aspartate transaminase (AST), ammonia, and total bile acids using corresponding commercial assay kits (Jiancheng Bioengineering Institute, Nanjing, China). Then, rats were perfused transcardially with normal saline to remove any remaining fluorescein in the circulation and brain samples were obtained to measure fluorescein and brain edema. For another group of rats, at 24 h of reperfusion, rats were sacrificed under slight ether anesthesia. The left hemispheres were harvested to prepare paraffin-embedded sections to perform immunofluorescence and cerebral cortices in the right hemisphere were dissected to isolate cerebral microvessels.

### Brain Edema Measurement

Alteration of the content of brain water was measured to assess brain edema according to the method previously described (Bi et al., 2016). In brief, the freshly obtained brain samples were weighed (wet weight). Following 24 h drying in an oven at 100°C, the brain samples were again weighed (dry weight). The brain edema was indexed as brain water (%) = (wet weight-dry weight)/wet weight ×100%.

### Isolation of Rat Cerebral Microvessels

Cerebral microvessels fragments were isolated from rat cortices according to a published protocol (Lee et al., 2019). Briefly, cortices were homogenized in Dulbecco’s phosphate-buffered saline (DPBS) using a loose-fit Dounce grinder and centrifuged at 2,000 g for 5 min. Pellet was suspended in 15% (w/v) 70 kDa dextran solution and centrifuged at 10,000 g for 15 min. Following removal of the top layer, microvessels were retrieved and transferred to a 40 μm cell strainer. After washing with DPBS, microvessels were harvested.

### Immunofluorescence

Paraffin-embedded sections of rat brains were deparaffinized in dimethylbenzene and rehydrated in decreasing ethanol followed with goat serum block and incubation with mixed primary antibodies of mouse anti-CD31 (SANTA CRUZ, 1:100) and rat anti-Ki67 (Cell Signaling Technology, 1:100). Anti-mouse IgG (Alexa fluoresceinor 594 conjugated) (Invitrogen, 1:1,000) and anti-rabbit IgG (Alexa fluoresceinor 488 conjugated) (Abcam, 1:1,000) were utilized as secondary antibodies, respectively. Immunofluorescence images were observed on LSM700 confocal laser scanning microscope. The proliferation of cerebral microvascular endothelial cells was calculated by overlap coefficient (m1) of red-green using Image-Pro Plus 6.0.

### Arg Determination by HPLC

Quantification of Arg in biological samples was developed by ortho-phthaldialdehyde (OPA) derivatization on HPLC instrument based on previous methods (Alkaitis et al., 2016; Schou-Pedersen and Lykkesfeldt, 2018). Briefly, 50 μl samples (plasma, culture medium or cell lysates) were deproteinated by mixing with 150 μl cold trichloroacetic acid (TCA) (1 M). After centrifugation, 100 μl supernatant was neutralized with 75 μl NaOH (1 M) followed by vortex with 175 μl derivatization reagent and loaded to Shimadzu HPLC with a YMC-Triart C18 column (5 μm, 150 × 2.0 mm). The derivatization reagent was a constitution of 7.46 mM OPA and 27.54 mM 3-mercaptopropionic acid in 200 mM potassium tetraborate. Mobile phase A was 25 mM PBS (pH 6.8) and mobile phase B was acetonitrile. Online fluorescence was measured at excitation and emission wavelengths of 340 and 455 nm, respectively. Chromatographic separation was performed at a flow rate of 1 ml/min with 8% phase B for 10 min, which was raised to 90% for 4 min, followed by re-equilibrium at 8% for 10 min. The retention time of Arg was 8.4 min.

### Western Blot

Proteins of cells or rat brain microvessels were lysed in RIPA lysis solution and quantified by BCA assay. Following SDS-PAGE, proteins were transferred to polyvinylidene fluoride (PVDF) membranes and blocked. The membrane was probed with corresponding primary antibodies overnight at 4°C followed by incubation with horseradish peroxidase (HRP)–conjugated secondary antibodies. The immunoreactive bands were visualized with HRP substrate (Vazyme Biotech, Nanjing, China) using 5200 Multi Chemiluminescent Imaging System (Tanon Technology, Shanghai, China). The proteins expressions were normalized by β-actin and expressed as the fold of control. The used primary antibodies and corresponding dilution ratios were as follows: cyclin A (Santa Cruz, 1:200); cyclin B (Wanleibio, 1:1,000); cyclin D (Wanleibio, 1:500); cyclin E (Wanleibio, 1:500); CDK1 (Wanleibio, 1:500); CDK2 (Wanleibio, 1:1,500); CDK4 (Wanleibio, 1:500); CDK6 (Wanleibio, 1:500); occludin (Wanleibio, 1:1,000); claudin-5 (Wanleibio, 1:1,000); ZO-1 (Wanleibio, 1:500); arginase1 (Cell Signaling Technology, 1:1,000); β-actin (Proteintech, 1:8,000). The used secondary antibodies and corresponding dilution ratios were anti-mouse IgG (Cell Signaling Technology, 1:3,000); anti-rabbit IgG (Cell Signaling Technology, 1:3,000).

### Statistical Analysis

All values were expressed as mean ± SD. Statistical significance was determined *via* unpaired 2-tailed *t*-test or 1-way ANOVA followed by Dunnett’s *post hoc* test on Graphpad Prism 8.0.2. All tests were considered statistically significant at *p* < 0.05.

## Results

### Toxic Molecule to Blood-Brain Barrier Under Hepatic Ischemia-Reperfusion Came From Liver Under Hepatic Ischemia-Reperfusion

**FIGURE 1:**
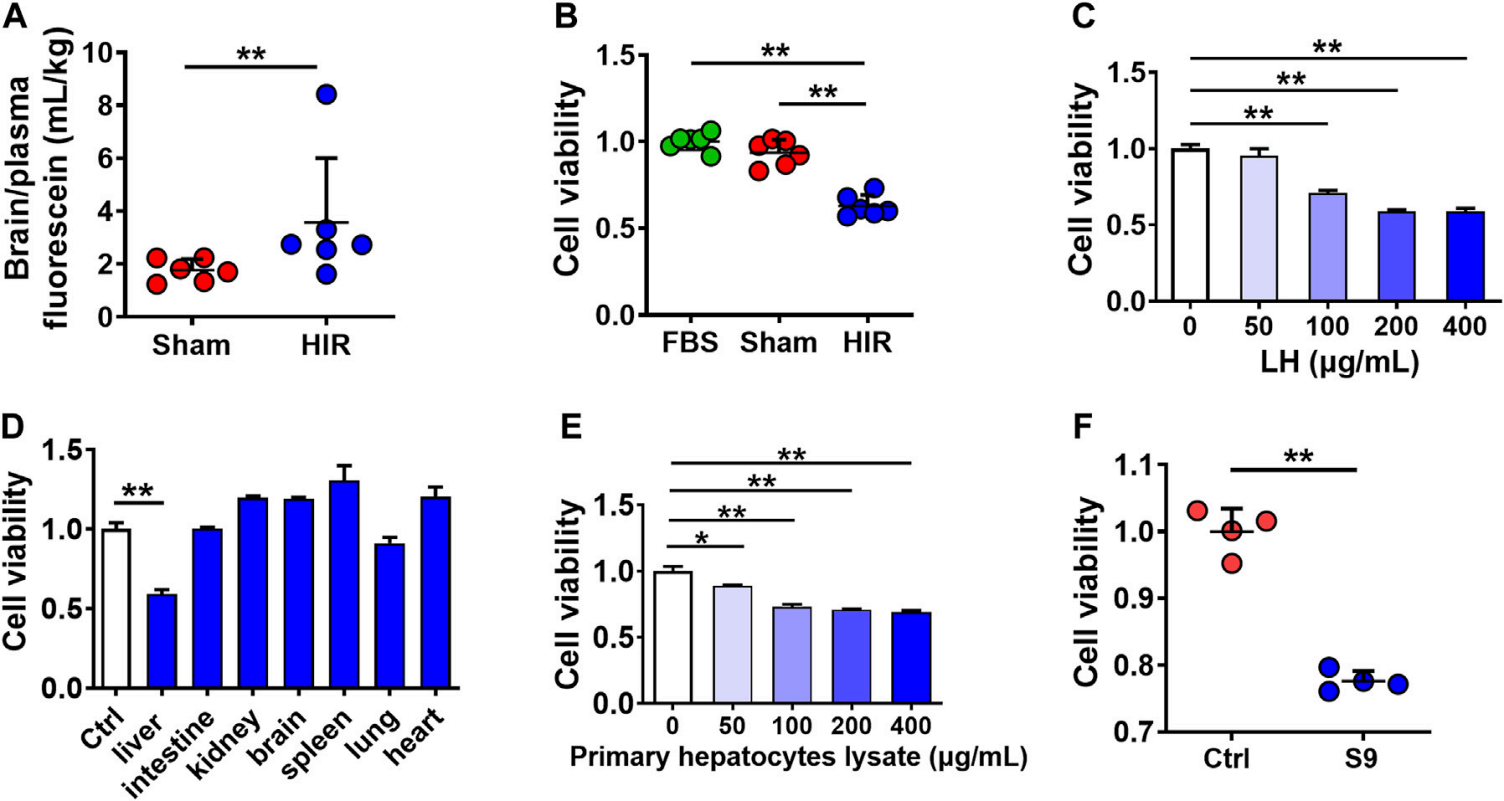
Toxic molecule to BBB under HIR came from the liver under HIR. **(A)** BBB permeability indexed as the ratio of brain to plasma fluorescein following intravenous injection (2 mg/kg) into hepatic ischemia-reperfusion (HIR) rats (1 h ischemia and 4 h reperfusion) and sham-operated (Sham) rats (*n* = 6). **(B)** Effect of 10% serum of HIR rats on hCMEC/D3 cell viability compared with 10% fetal bovine serum (FBS) and serum of sham rats (*n* = 6). **(C)** Effect of healthy rat liver homogenate (LH), **(D)** different tissues homogenates (200 μg/ml), **(E)** primary hepatocyte lysate from healthy rats, and **(F)** human liver S9 (supernatant of 9,000 g centrifugation) (200 μg/ml) on hCMEC/D3 cell viability (*n* = 4). Data were expressed as mean ± SD. *p < 0.05, **p < 0.001 versus control cells (Ctrl) or Sham rats; 1-way ANOVA followed by Dunnett’s *post hoc* test or unpaired *t*-test in panels **(A,F)**.

To explore whether HIR impairs BBB, fluorescein (probe of BBB permeability) was administered to HIR rats undergoing 1 h ischemia and 4 h reperfusion. The results showed that HIR significantly increased the ratio of brain to plasma fluorescein (Figure 1A), demonstrating BBB impairment. To identify the damage compound to BBB under HIR, hCMEC/D3 cells were cultured with 10% HIR rat serum for 24 h. Cell viability was reduced to 60% of cells cultured with 10% FBS or serum of Sham rats (Figure 1B). To imitate the physical damage of the liver under HIR and further test whether the toxic substance to BBB came from the liver, hCMEC/D3 cells were incubated with liver homogenate from healthy rats. It was consistent with our expectation that rat liver homogenate concentration-dependently damaged hCMEC/D3 cells (Figure 1C). However, homogenates of other tissues, such as the kidney, brain, spleen, lung, and heart, did not show toxicity to hCMEC/D3 cells (Figure 1D). Moreover, both primary rat hepatocytes lysate (Figure 1E) and S9 of the human liver (200 μg/ml) also showed remarkable cytotoxicity (Figure 1F). These results suggested that the damage molecule to BBB under HIR was liver-special.

### Screening and Identification of Damage Molecule to Blood-Brain Barrier in Liver Homogenate Using hCMEC/D3 Cells

**FIGURE 2:**
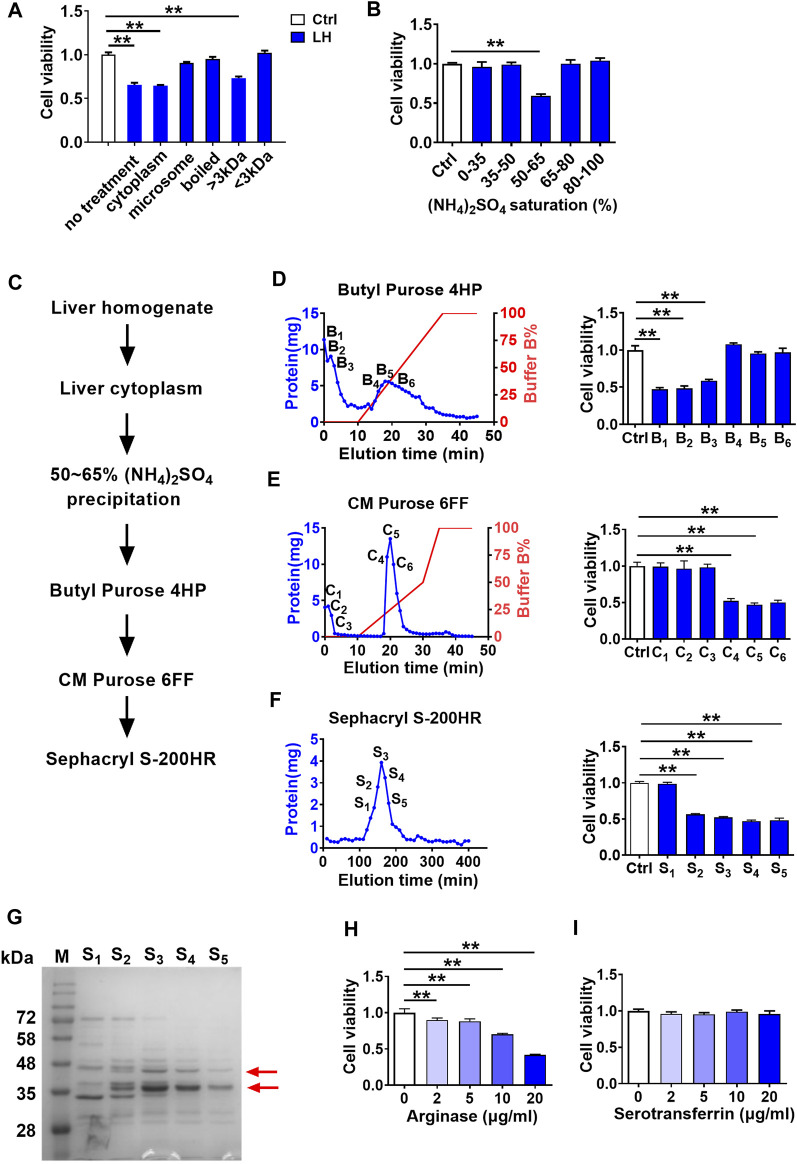
Screening and identification of damage molecule to BBB in liver homogenate using hCMEC/D3 cells. **(A)** Effect of liver homogenate with different treatments on hCMEC/D3 cell viability (*n* = 4). **(B)** Effect of fractional (NH_4_)_2_SO_4_ precipitation of rat liver homogenate on hCMEC/D3 cell viability (*n* = 4). **(C)** Purification scheme for a toxic molecule to BBB from rat liver homogenate. **(D)** Elution curves of proteins in liver homogenate separated by columns of Butyl Purose 4HP, **(E)** CM Purose 6FF, and **(F)** Sephacryl S-200HR (blue line: protein quantity; red line: gradient elution procedure) and the effects of fractions eluted with high abundance on hCMEC/D3 cell viability (*n* = 4). **(G)** Electrophoresis image of the final purified protein samples stained by Coomassie Brilliant Blue (M: molecular weight marker; red arrows indicated bands selected to apply protein identification). **(H)** Effect of arginase and **(I)** serotransferrin on hCMEC/D3 cell viability (*n* = 4). Data were expressed as mean ± SD. **p < 0.01 versus control cells (Ctrl); 1-way ANOVA followed by Dunnett’s *post hoc* test.

To further characterize the toxic molecule, several treatments were applied to rat liver homogenate (200 μg/ml), including differential centrifugation at 100,000 g, boiling at 100°C, and ultrafiltration through a 3 kDa membrane. From the results in Figure 2A, only the cytoplasm (supernatant of 100,000 g centrifugation) and large molecules (molecular weight >3 kDa) parts retained cytotoxicity to hCMEC/D3 cells. Moreover, data in Figure 2B of (NH_4_)_2_SO_4_ fractional precipitation (Hirayama et al., 1998) showed that the precipitation by 50∼65% saturated (NH_4_)_2_SO_4_ of rat liver homogenate impaired hCMEC/D3 cells viability. These results indicated that the toxic molecule to BBB was a protein mainly located in the cytoplasm of hepatocytes.

The toxic compound was further separated and purified (Figure 2C). Liver cytoplasm was precipitated to 50∼65% saturated (NH_4_)_2_SO_4_ followed by sequential separation on columns of Butyl Purose 6HP, CM Purose 6 FF, and Sephacryl S-200 HR (Figures 2D–F) with an assay of cell viability. Finally, four fractions with damage activity (S2∼S5) and a fraction without damage activity (S1) were obtained. These fractionated proteins were electrophoresed on SDS-PAGE (Figure 2G). Two differential bands in S2–S5 compared with S1 fraction as red arrows indicated in Figure 2G were collected for protein identification. Three proteins (annexin, arginase-1, and serotransferrin) possessing high unique peptides and unique sequence coverage were taken into consideration (Table 1). Annexin is a Ca^2+^-triggered phospholipid-binding protein serving as an important component of the plasma membrane repair system without evidence of cytotoxicity (Lauritzen et al., 2015). Arginase was involved in the metabolism of bioactive molecules like Arg and NO. Serotransferrin was reported to regulate ferroptosis (Gao et al., 2015). With this regard, arginase and serotransferrin were taken into consideration. Figures 2H,I demonstrated that arginase (Sigma-Aldrich, St. Louis, MO, United States) but not transferrin (Sino Biological Inc., Beijing, China) damaged hCMEC/D3 cells, primarily suggesting that toxic molecule to BBB in liver was arginase.

**TABLE 1:** List of proteins detected in finally purified protein sample from rat liver using TripleTOF mass and TurboSEQUEST software.

First protein	Gene name	Description	Unique peptides	Unique sequence coverage (%)	Mol. weight (kDa)
Q6IMZ3	Anxa6	Annexin	41	57.7	75.755
P07824	Arg1	Arginase-1	39	98.8	34.973
P12346	Tf	Serotransferrin	38	45.1	76.394
P25093	Fah	Fumarylacetoacetase	27	74.5	45.975
P63018	Hspa8	Heat shock cognate 71	25	40.1	70.87
Q5EBD0	Sec14l2	SEC14-like 2	19	49.1	46.205
P14141	Ca3	Carbonic anhydrase 3	16	76.2	29.431

### Arginase Was Verified to Be the Toxic Molecule to hCMEC/D3 Cells

**FIGURE 3:**
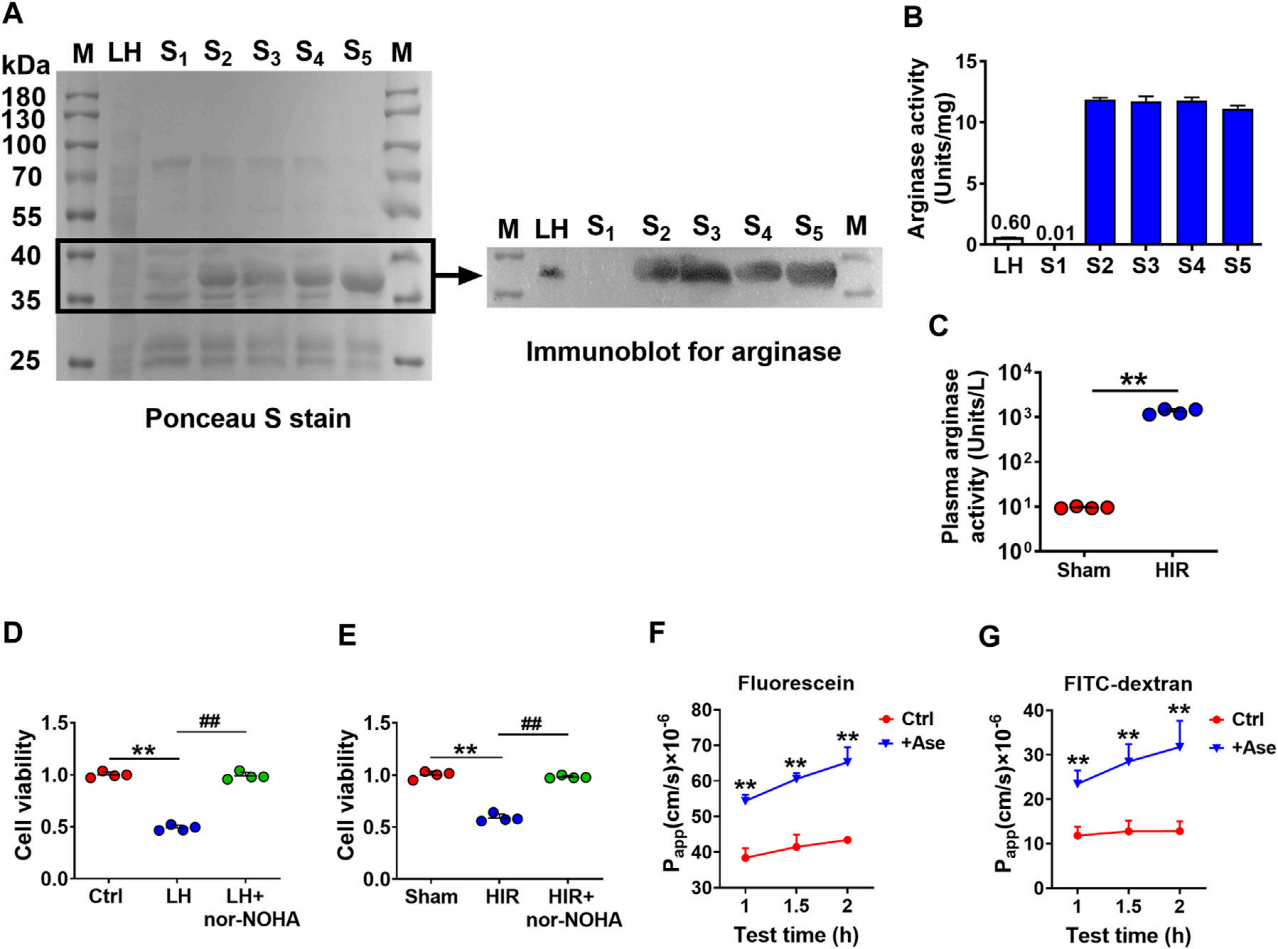
Arginase was verified to be the toxic molecule to hCMEC/D3 cells. **(A)** Ponceau S stain on the whole membrane and immunoblot image for arginase in finally purified protein samples S1∼S5 (20 μg) compared with rat liver homogenate (LH) (20 μg) (M, molecular weight marker). **(B)** Arginase activity assay in S1∼S5 and LH. (C) Plasma arginase activity in hepatic ischemia-reperfusion (HIR) rats (1 h ischemia and 4 h reperfusion) and sham-operated (Sham) rats (*n* = 4). **(D)** Effect of arginase inhibitor nor-NOHA (50 μM) on damaged viability of hCMEC/D3 cells caused by LH (200 μg/ml) or **(E)** 10% serum of HIR rats (*n* = 4). **(F)** Apparent permeability coefficients (P_app_) assay of fluorescein and **(G)** FITC-Dextran across hCMEC/D3 *in vitro* BBB treated with arginase (20 μg/ml) (Ase) (*n* = 6). Data were expressed as mean ± SD. **p < 0.01 versus control cells (Ctrl) or Sham rats; ^##^ *p* < 0.01 versus cells treated with LH or HIR rat serum; 1-way ANOVA followed by Dunnett’s *post hoc* test or unpaired *t*-test in panel **(C)**.

To further confirm that arginase was the toxic molecule to BBB, arginase quantities in purified fractions S1∼S5 (20 μg/ml protein) were measured with rat liver homogenate (20 μg/ml protein) as a positive control using western blot. Since S1∼S5 were purified protein fractions, the conventionally used loading controls such as β-actin or GAPDH were not applicable, the Ponceau S stain was performed on the whole membrane before the immunoblot to visualize the general protein quantity (Figure 3A). The immunoblot result showed that S2∼S5 were determined with more evident arginase bands than liver homogenate, while arginase in S1 was undetectable (Figure 3A). Meanwhile, the arginase activity was also measured in S1∼S5 using the corresponding assay kit. S2∼S5 were detected with higher arginase activity (10 Units/mg) than rat liver homogenate (0.6 Units/mg), whereas arginase activity in S1 was considerably low (0.01 Units/mg) (Figure 3B). Dramatically high arginase activity was also demonstrated in the serum of HIR rats (HIR rats 1,338.06 ± 188.39 Units/L versus Sham rats 9.57 ± 0.46 Units/L) (Figure 3C). Furthermore, coadministration of arginase inhibitor Nω-hydroxy-nor-L-arginine (nor-NOHA) (Sigma-Aldrich, St. Louis, MO, United States) (50 μM) efficiently attenuated impairment of hCMEC/D3 cells induced by 200 μg/ml rat liver homogenate (Figure 3D) or 10% HIR rat serum (Figure 3E). Moreover, 20 μg/ml arginase efficiently increased the permeability of fluorescein and FITC-dextran (3 kDa) across BBB in an *in vitro* model (Figures 3F,G). All these results confirmed that the toxic molecule released from the injured liver was arginase.

### Arginase Damaged hCMEC/D3 Cells *via* Depriving Arg

**FIGURE 4:**
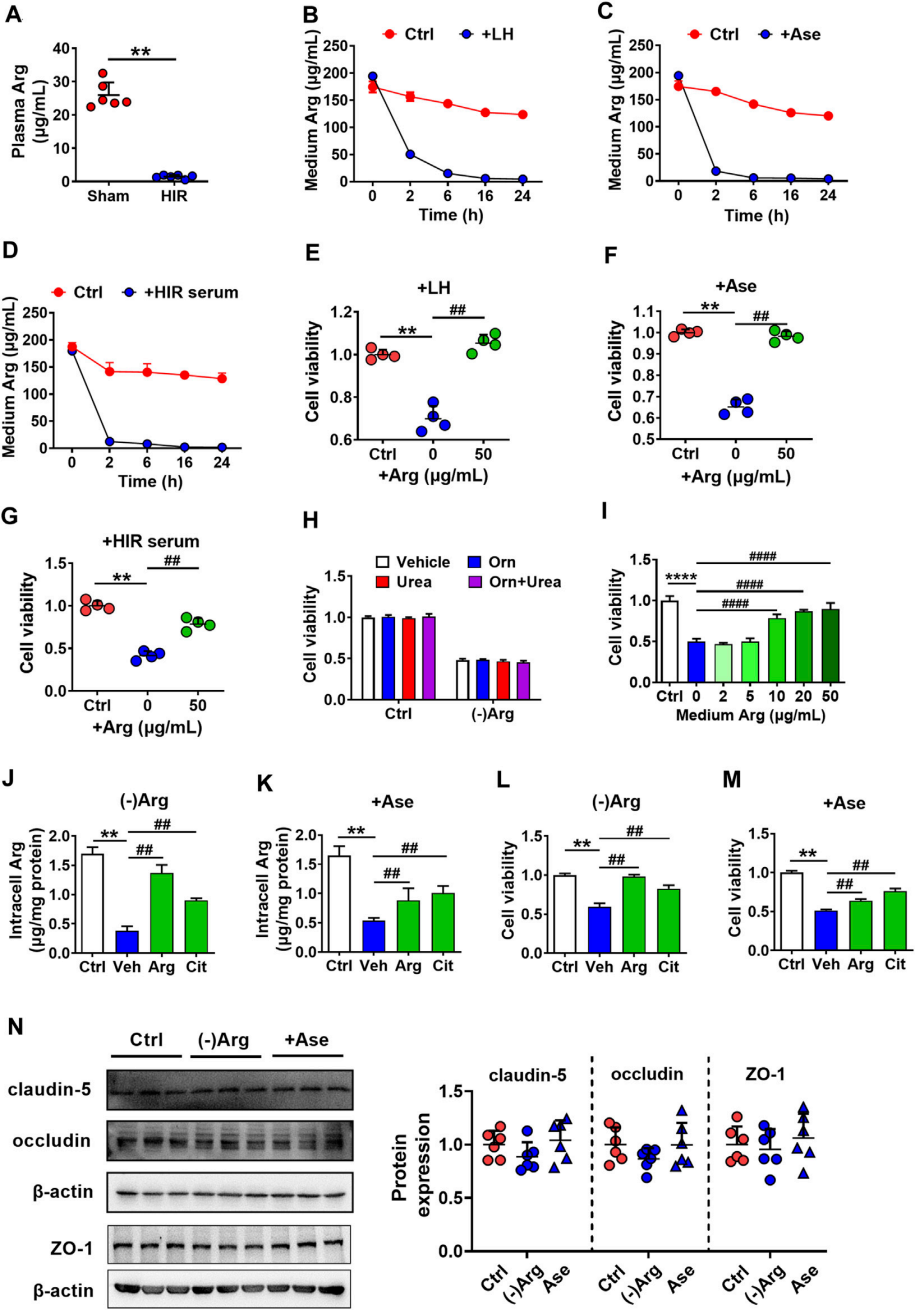
Arginase damaged hCMEC/D3 cells *via* depriving Arg. **(A)** Plasma arginine (Arg) level in hepatic ischemia-reperfusion (HIR) rats (1 h ischemia and 4 h reperfusion) and sham-operated (Sham) rats (*n* = 4). **(B)** Arg levels in culture medium following treatment with rat liver homogenate (LH) (200 μg/ml), **(C)** arginase (20 μg/ml) (Ase), or **(D)** 10% serum of HIR rats (*n* = 3). **(E)** Effect of Arg supplement on damaged viability of hCMEC/D3 cells caused by LH, **(F)** Ase, or **(G)** HIR rat serum (*n* = 4). **(H)** Effect of Arg-free culture ((-)Arg), adding ornithine (Orn) (1 mM), urea (1 mM) or combination of Orn with urea (Orn + Urea) on hCMEC/D3 cell viability (*n* = 4). **(I)** Effect of addition of increasing Arg on hCMEC/D3 cells damage caused by (-)Arg (*n* = 4). **(J)** Effect of adding Arg or citrulline (Cit) (200 μg/ml) on the decrease in intracellular Arg level caused by (-)Arg or **(K)** Ase compared with adding vehicle (Veh) (*n* = 3). **(L)** Effect of adding Arg or Cit (200 μg/ml) on damaged cell viability of hCMEC/D3 caused by (-)Arg or **(M)** Ase compared with adding vehicle (Veh) (*n* = 4). **(N)** Western blot of tight junctions in hCMEC/D3 cells treated with (-)Arg or Ase (*n* = 6). Data were expressed as mean ± SD. **p < 0.01 versus control (Ctrl) cells or Sham rats; ^##^ *p* < 0.01 versus cells treated with LH, Ase, and HIR rat serum or (-)Arg; 1-way ANOVA followed by Dunnett’s *post hoc* test or unpaired *t*-test in panel **(A)**.

Arginase metabolizes Arg, indicating that arginase impaired hCMEC/D3 cells possibly *via* depriving Arg. It was in line with the deduction that obviously lower Arg levels were observed in the serum of HIR rats (HIR rats 1.44 ± 0.51 μg/ml versus Sham rats 25.91 ± 3.87 μg/ml) (Figure 4A). Incubation with liver homogenate (200 μg/ml), arginase (20 μg/ml), or 10% HIR rat serum (10%) (Figures 4B–D) remarkably decreased Arg levels in medium to 50.49 ± 2.63, 18.52 ± 1.57, and 12.63 ± 0.16 μg/ml, respectively, at 2 h after treatments. Moreover, after treatment for 6 h, levels of medium Arg were all declined to lower than 10 μg/ml. Exogenous supplement with 50 μg/ml Arg for four times at an interval of 6 h obviously attenuated the hCMEC/D3 cells damage caused by liver homogenate, arginase, and HIR rat serum (Figures 4E–G). As arginase is responsible for converting Arg to urea and ornithine, effects of Arg deficiency, urea, and ornithine on hCMEC/D3 viability were measured. The Arg-free medium was prepared. Urea (1 mM) and ornithine (1 mM) or their combination were added to normal medium or Arg-free medium of hCMEC/D3 cells for 24 h. Results showed that incubation in Arg-free medium impaired hCMEC/D3, while urea and ornithine and their combination little damaged hCMEC/D3 (Figure 4H). Moreover, the addition of increasing Arg reversed the impairment of hCMEC/D3 caused by Arg-free medium culture (Figure 4I). Furthermore, the supplement of Arg (200 μg/ml) and its precursor citrulline (200 μg/ml) restored intracellular Arg level in hCMEC/D3 cells treated with Arg-free medium or arginase (20 μg/ml) (Figures 4J,K) and attenuated the damaged viability of hCMEC/D3 cells (Figures 4L,M). These results demonstrated that arginase damaged hCMEC/D3 cells *via* depriving Arg. In addition, the tight junctions, highly expressed in endothelial cells, seal the paracellular space and are responsible for the barrier function of BBB. Thus, the effect of Arg deprivation on main tight junctions claudin-5, occluding, and ZO-1 were investigated. Western blot results in Figure 4N showed that tight junctions remained unaltered in hCMEC/D3 cells with treatment of 20 μg/ml arginase or Arg-free culture.

### Both Arginase and Arg Deficiency Impaired hCMEC/D3 Cells *via* Inhibiting Cell Proliferation

**FIGURE 5:**
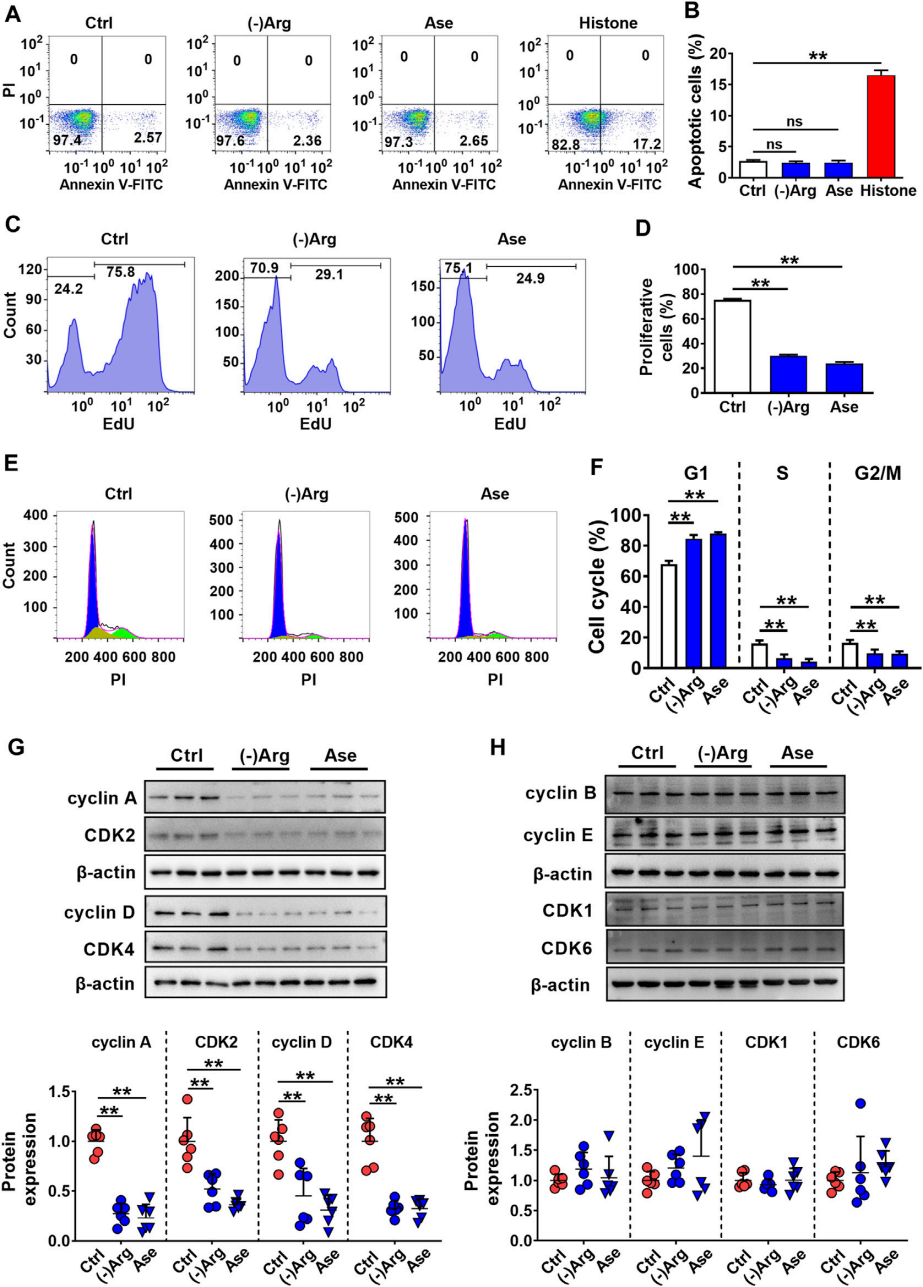
Both arginase and Arg deficiency impaired hCMEC/D3 cells *via* inhibiting cell proliferation. **(A,B)** Cell apoptosis analysis by Annexin Ⅴ-FITC/PI staining of hCMEC/D3 cells treated with Arg-free culture ((-)Arg), arginase (20 μg/ml) (Ase), or histone (10 μg/ml) (*n* = 4). **(C,D)** Cell proliferation assay by EdU incorporation of hCMEC/D3 cells treated with (-)Arg or Ase (*n* = 4). **(E, F)** Cell cycle analysis by PI staining of hCMEC/D3 cells treated with (-)Arg or Ase (*n* = 4). **(G, H)** Western blot of cell cycle–related proteins in hCMEC/D3 cells treated with (-)Arg or Ase (*n* = 6). Data were expressed as mean ± SD. **p < 0.01; ns, no significance versus control (Ctrl) cells; 1-way ANOVA followed by Dunnett’s *post hoc* test.

To further investigate the mechanism of arginase-induced hCMEC/D3 cells damage, cell apoptosis, necrosis, and proliferation were investigated by flow cytometry in hCMEC/D3 cells following treatment with arginase (20 μg/ml) or Arg-free medium culture for 24 h. Histone (10 μg/ml) was utilized as a positive control of cell apoptosis. Data from annexin V/PI staining (Figures 5A,B) showed the distribution of cells in apoptosis (Annexin V^+^/PI^−^) or necrosis (Annexin V^+^/PI^+^) was unaltered upon Arg deprivation. The results indicated that apoptosis or necrosis was not involved in arginase-induced hCMEC/D3 cells damage. EdU incorporation staining showed that both Arg-free culture and arginase extremely decreased EdU-incorporated cells, which was less than 40% of control cells (Figures 5C,D), demonstrating that cell proliferation was inhibited. Cell cycle analysis showed that compared with control, the percentage of G1 phase cells was significantly increased with treatment of arginase or Arg-free medium accompanied by reduced cells distribution in the S and G2/M phase (Figures 5E,F), suggesting cell cycle arrest in G1 phase upon Arg deprivation. To further substantiate this result, cell cycle–related functional proteins were quantified by western blot. Figures 5G,H exhibited that both arginase and Arg-free culture notably downregulated cyclin A, cyclin D, CDK2, and CDK4, while cyclin B, cyclin E, CDK1, and CDK6 proteins remained unaltered, further confirming the cell cycle arrest assessed by flow cytometer.

### Hepatic Ischemia-Reperfusion Impaired Blood-Brain Barrier of Rats *via* Substantially Increasing Arginase Release From the Injured Liver

**FIGURE 6:**
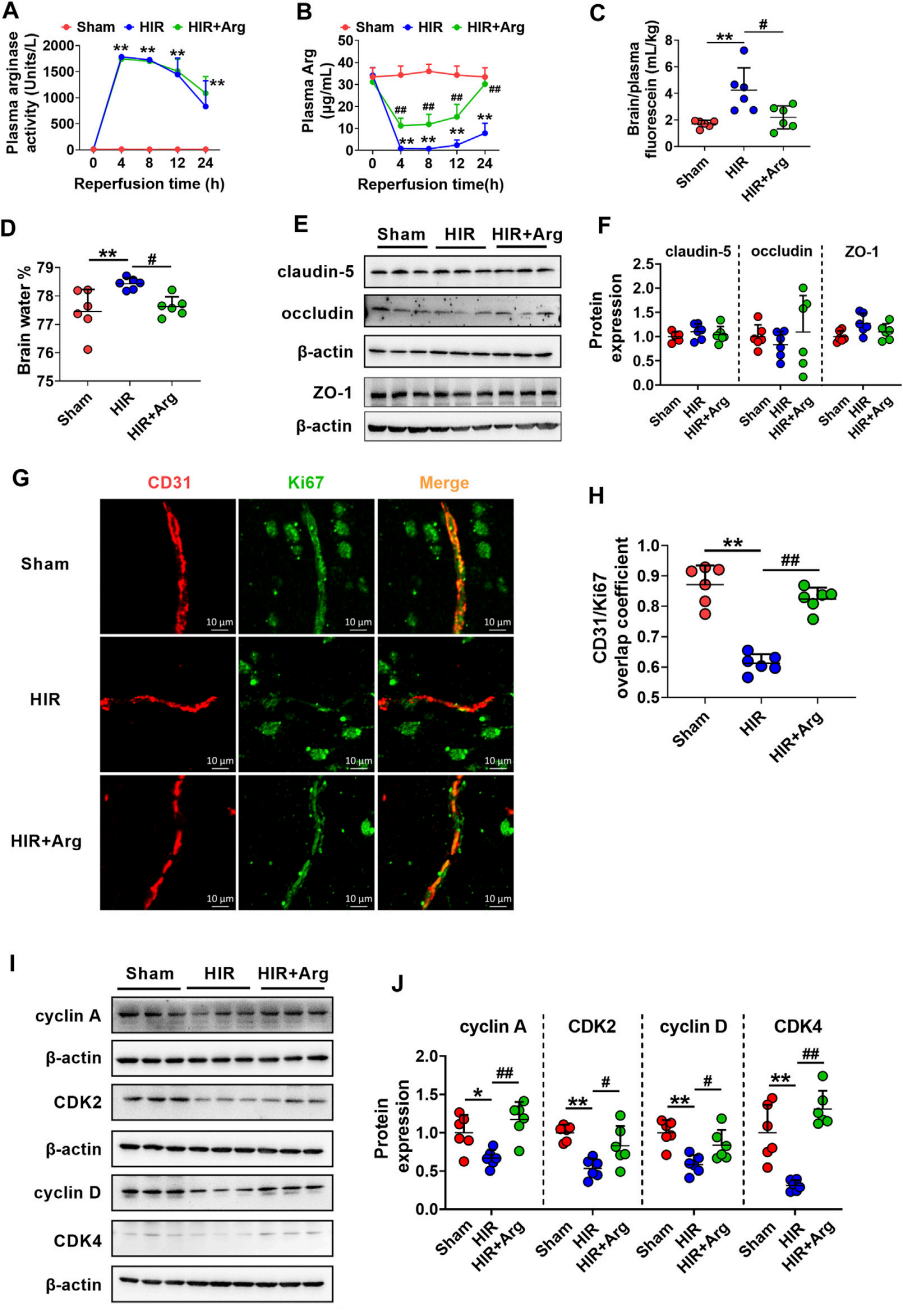
HIR impaired BBB of rats *via* substantially increasing arginase release from the injured liver. **(A)** Plasma arginase activity and **(B)** arginine (Arg) level in sham-operated (Sham) rats, hepatic ischemia-reperfusion (HIR) rats, and HIR rats supplemented with Arg (0.18 g/kg) before surgery and at 2, 6, and 12 h of reperfusion (HIR + Arg) (*n* = 6). **(C)** BBB permeability indexed as the ratio of brain to plasma fluorescein and **(D)** brain water content in experimental rats (*n* = 6). **(E,F)** Western blot of tight junctions in the brain of experimental rats (*n* = 6). **(G)** Image of dual immunofluorescence of CD31 and Ki67 in the brain paraffin section and **(H)** the calculated overlap coefficient of CD31/Ki67 (to CD31) in experimental rats (*n* = 6). **(I,J)** Western blot cell cycle–related proteins in isolated cerebral microvessels from experimental rats (*n* = 6). Data were expressed as mean ± SD. *p < 0.05, **p < 0.01 versus Sham rats; ^#^ *p* < 0.05, ^##^ *p* < 0.01 versus HIR rats; 1-way ANOVA followed by Dunnett’s *post hoc* test.

To further investigate the involvement of released arginase from the liver to BBB impairment under HIR, HIR rats undergoing 1 h hepatic ischemia and 24 h reperfusion were developed and supplemented with Arg by oral administration. Liver failure was evidenced by the remarkably enhanced serum biochemical parameters, including ALT, AST, ammonia, and total bile acids (Table 2). Supplement with Arg did not attenuate the liver injury by HIR. Both plasma arginase activity and Arg levels were simultaneously measured. It was consistent with our expectation that compared with Sham rats (about 10 Units/L), HIR rats showed significantly higher plasma arginase activity, which were almost over 1,000 units/L during 24 h reperfusion (Figure 6A). In line with the remarkable increase in plasma arginase activity, dramatically decreased plasma Arg levels were also observed in HIR rats (Figure 6B). The plasma Arg levels of HIR rats during 12 h reperfusion were less than 5% of Sham rats. The plasma Arg level of HIR rats following 24 h reperfusion showed a trend to recover but was still significantly lower than Sham rats. Supplement with Arg remarkably reversed the decrease of plasma Arg levels caused by HIR but did not affect the increase of plasma arginase activity induced by HIR.

**TABLE 2:** Biochemical parameters related to liver function in serum of sham-operated (Sham) and hepatic ischemia-reperfusion (HIR) (1 h ischemia and 24 h reperfusion) rats and HIR rats orally supplemented with Arg (0.18 g/kg) before surgery and at 2, 6, and 12 h of reperfusion (HIR + Arg) (*n* = 6).

Parameters	Sham	HIR	HIR + Arg
ALT (IU/L)	30.60 ± 15.38	152.16 ± 22.14**	164.87 ± 25.25**
AST (IU/L)	41.34 ± 17.55	166.42 ± 23.78**	155.79 ± 26.99**
Ammonia (μM)	212.43 ± 32.70	282.44 ± 41.75*	295.07 ± 54.56*
Total bile acids (μM)	16.84 ± 7.23	46.86 ± 18.94*	58.32 ± 15.03*

Data were expressed as mean ± SD. *p < 0.05, **p < 0.01 versus Sham rats; 1-way ANOVA followed by Dunnett’s *post hoc* test.

The function of BBB in HIR rats was investigated using permeability to fluorescein and content of brain water. The results showed that HIR considerably increased BBB permeability to fluorescein (Figure 6C) and content of brain water (HIR rats 78.43 ± 0.20% vs. Sham rats 77.45 ± 0.48%) (Figure 6D), demonstrating impairment of BBB function. Western blot in Figures 6E,F showed that expressions of tight junctions claudin-5, occludin, and ZO-1 in brains were unaltered in HIR rats. Relocalization of brain tight junctions was reported to also contribute to BBB breakdown in some pathological processes (Bennett et al., 2010), whether HIR and released arginase impair BBB through relocalization of tight junctions needs further investigation. To detect the cerebral endothelial cells proliferation, dual immunofluorescence of CD31 (a marker of vascular endothelium) and Ki67 (a marker of cell proliferation) was performed on the brain paraffin section. Data from confocal laser scanning microscope (Figure 6G) showed that HIR notably reduced the expression of Ki67 in cerebral microvessels of HIR rats compared with Sham rats, evidenced by a lower overlap coefficient of CD31/Ki67 (to CD31) (Figure 6H). It was in line with the western blot results (Figures 6I,J) that HIR significantly downregulated expressions of cell cycle–related proteins including cyclin A, CDK2, cyclin D, and CDK4 in isolated cerebral microvessels of rats. More importantly, the Arg supplement almost reversed alterations in BBB function, cerebral endothelial cells proliferation, and expressions of proteins related to the cell cycle in isolated cerebral microvessels caused by HIR.

## Discussion

Accumulating evidence has illustrated that liver transplantation could induce neurological complications and long-term cognitive impairment (Erol et al., 2007; Bucuvalas, 2013; Kaller et al., 2013; Gungor et al., 2017). HIR is the main pathophysiological process during liver transplantation. Thus, we developed the HIR rat model to investigate whether BBB was impaired under HIR. We first found that serum of HIR rats impaired hCMEC/D3 cells, indicating the existence of a toxic component in the serum of HIR rats. In order to investigate whether the toxic component came from the liver, the effects of liver homogenate on hCMEC/D3 cells were documented. It was in agreement with our expectation that homogenate of the liver rather than other tissues impaired hCMEC/D3 cells, demonstrating that the toxic component was liver-special. After protein separation and purification from liver homogenate, the toxic compound was ultimately identified to be arginase. Arginase inhibitor nor-NOHA efficiently attenuated the impairment of hCMEC/D3 cells caused by liver homogenate and serum of HIR rats, which further confirmed that the toxic compound from the liver was arginase. High activity of arginase was detected in the serum of HIR rats. Similar results were also observed in patients undergoing acute hepatitis (Ikemoto et al., 2001) and alcoholic liver disease (Gao et al., 2019).

In the liver, arginase converts Arg into urea and ornithine. Both urea and ornithine little affected hCMEC/D3 cell viability, excluding the involvement of these two metabolites in arginase-induced BBB impairment. Substantially released arginase may accelerate Arg metabolism, leading to Arg deficiency. Indeed, it is in line with the observation of plasma Arg deficiency in acute liver failure models of thioacetamide-treated rats (Bekpinar et al., 2015) and devascularized porcine (Sharma et al., 2012). Roles of Arg deficiency in BBB impairment by arginase were further investigated in hCMEC/D3 cells. Both Arg-free medium and arginase impaired cell viability, which were reversed by both Arg and its precursor citrulline. All these results gave a conclusion that arginase impaired BBB *via* depriving Arg.

Cell impairment is often involved in apoptosis, necrosis, and inhibition of cell proliferation. Annexin V/PI double staining demonstrated no obvious apoptosis nor necrosis, but EdU staining showed that both arginase and Arg deficiency significantly lowered numbers of proliferative cells. Progress through each phase of the cell cycle is strictly regulated by coordination of cyclins and cyclin-dependent kinases (CDKs), in which cyclin D-CDK4/CDK6 and cyclin E-CDK2 complexes promote G1-S transition, cyclin A-CDK2 promotes S phase progression, and cyclin A/cyclin B-CDK1 controls G2/M transition (Malumbres and Barbacid, 2009). In the current study, cyclin A, cyclin D, CDK2, and CDK4 were all downregulated in hCMEC/D3 cells treated with Arg deficiency culture or arginase and the cell cycle was detected to be arrested in the G1 phase, suggesting the decreased cyclin D and CDK4 mainly contributed to the final cell cycle progression. It is in line with the reports that cyclin D knockdown and CDK4 knockdown arrested cells in the G1 phase (Chen et al., 2017, 2019).

Different from *in vitro* study that Arg amount is limited in media, Arg as a semi-essential amino acid may be replenished by other sources like citrulline (Rashid et al., 2020) or skeletal muscle breakdown (Wheatley et al., 2003) *in vivo*. Thus, the time course of plasma arginase and Arg under HIR was documented in rats undergoing 1 h ischemia and 24 h reperfusion. During 12 h reperfusion, plasma Arg level was decreased to lower than 1 μg/ml (less than 5% of sham rats) accompanied by the remarkable release of arginase over 1,000 Units/L compared with 10 Units/L in Sham rats, indicating that Arg deprivation by the substantially increased arginase almost abolishes the replenishment of Arg from other sources. At 24 h of reperfusion, accompanied by decreased plasma arginase activity to near 800 Units/L, the effect of Arg replenishment by other sources was reflected. Plasma Arg began to rise to nearly 7.8 μg/ml but still significantly lower than that in Sham rats. It is consistent with clinical evidence that plasma Arg dropped rapidly and then rose to the level under baseline in patients during liver transplantation (Roth et al., 1994; Becker et al., 2009). HIR significantly impaired BBB, evidenced by enhanced permeability to fluorescein and water content accompanied by decreased proliferation and cell cycle–related proteins in endothelial cells of cerebral microvessels. It is in line with other researches that BBB was damaged *via* inhibiting brain microvascular endothelial cells proliferation (Buşu et al., 2013; Castillo-Melendez et al., 2015; Gong et al., 2019) and that BBB leakage was attenuated by promoting endothelial cells proliferation (Gandin et al., 2016; Shi et al., 2019). The oral supplement of Arg increased plasma Arg in HIR rats to over 10 μg/ml and was demonstrated to reverse the above BBB alterations. It is consistent with the *in vitro* result of the recovery of hCMEC/D3 cell viability when Arg level in the medium is above 10 μg/ml (Figure 4I). All these results indicate that HIR impairs BBB *via* inhibiting brain microvascular endothelial cell proliferation, which is partly attributed to Arg deficiency.

Several reports have demonstrated the protective effect of arginase on CNS, but some opposite results have also been documented (Fouda et al., 2020). Arginase knockout mice show increased brain infarction and worsened neurological deficit after stroke compared to wild type (Ahmad et al., 2016). The number of arginase-positive macrophages is correlated with neuroprotection and functional recovery after experimental stroke (Hamzei Taj et al., 2016). In contrast, in Alzheimer’s disease (AD) mice (Kan et al., 2015), areas of hippocampal neuronal death are associated with the increase of extracellular arginase and the fact that arginase inhibitor effectively reversed memory loss. Clinical trials have also shown that mRNA of arginase is acutely upregulated in peripheral blood of ischemic stroke and mild traumatic brain injury patients, which is correlated with ischemic stroke severity and post-stroke immune suppression (Petrone et al., 2017; Yoo et al., 2019). These reports have demonstrated the deleterious role of arginase, which is in line with our results.

Arg is the substrate to produce NO via endothelial-type NO synthase (eNOS) in the vasculature; the arginase-triggered Arg depletion is implicated in eNOS uncoupling, a pathological condition in which eNOS donates electrons received from NADPH to O_2_ instead of Arg and produces superoxide (O_2_^−^) (Li and Förstermann, 2013; Villalba et al., 2017). Our pre-experiments showed that arginase indeed increased the cellular reactive oxide species (ROS) in hCMEC/D3 cells (Supplementary Figure S1A). However, the free radical scavenger N-acetyl-L-cysteine (NAC) failed to attenuate the damaged cell viability caused by arginase (Supplementary Figure S1B). Moreover, eNOS inhibitor L-NAME only slightly damaged hCMEC/D3 cell viability at a high concentration (5 mM) (Supplementary Figure S2A) and NO donor sodium nitroprusside (SNP) also failed to ameliorate the damaging effect of arginase (Supplementary Figure S2B). Thus, the oxidative stress caused by eNOS uncoupling or declined NO production seems not the main contributor to arginase-induced hCMEC/D3 cell damage. Besides, the decreased NO would potentially affect cerebral vessel relaxation. Therefore, the role of eNOS uncoupling and potentially altered cerebral blood flow in arginase-induced BBB impairment under HIR needs further investigation.

It should be noticed that circulating levels of proinflammatory cytokines and transcription factors, including interleukin-6, tumor necrosis factor-alpha, and high mobility group box1, during HIR were also increased under HIR (Nastos et al., 2014), possibly contributing to BBB impairment, which needs further investigation.

In conclusion, our main findings are as follows: HIR induces the substantial release of arginase from the injured liver, leading to systemic Arg deficiency; Arg deficiency further impairs BBB via inhibiting the proliferation of cerebral microvascular endothelial cells by cell cycle arrest.

## Data Availability

The original contributions presented in the study are included in the article/Supplementary Material; further inquiries can be directed to the corresponding authors.
