# Supplementary figures and images for: Hepatic Ischemia-Reperfusion Impairs Blood-Brain Barrier Partly Due to Release of Arginase From Injured Liver (part 1 of 3)
Source: Front Pharmacol. 2021 Oct 13;12:724471. doi: 10.3389/fphar.2021.724471 (PMC8548691; doi:10.3389/fphar.2021.724471)

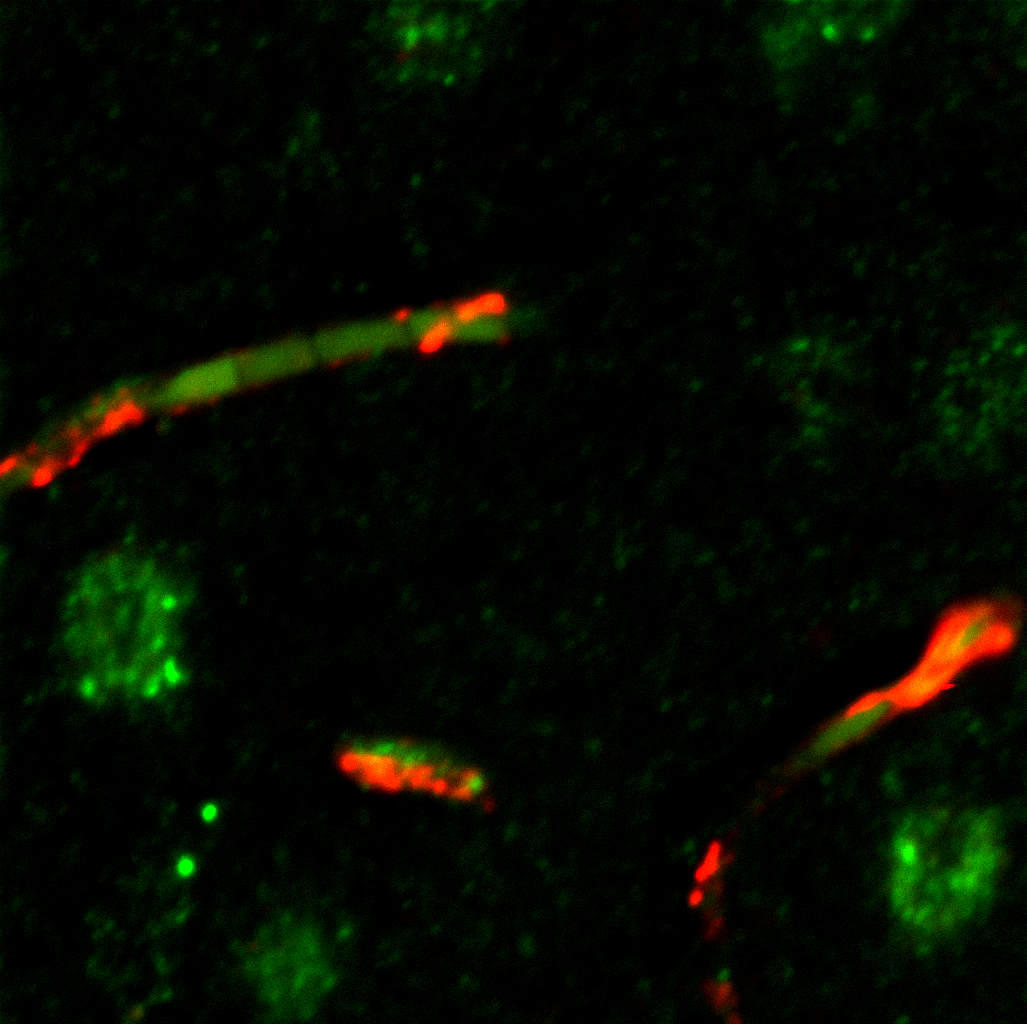

Supplement: Supplementary file 4 [file DataSheet8.ZIP › Immunofluorescence (Figure 6G, part3)/13/1/5s_c1+2.tif]

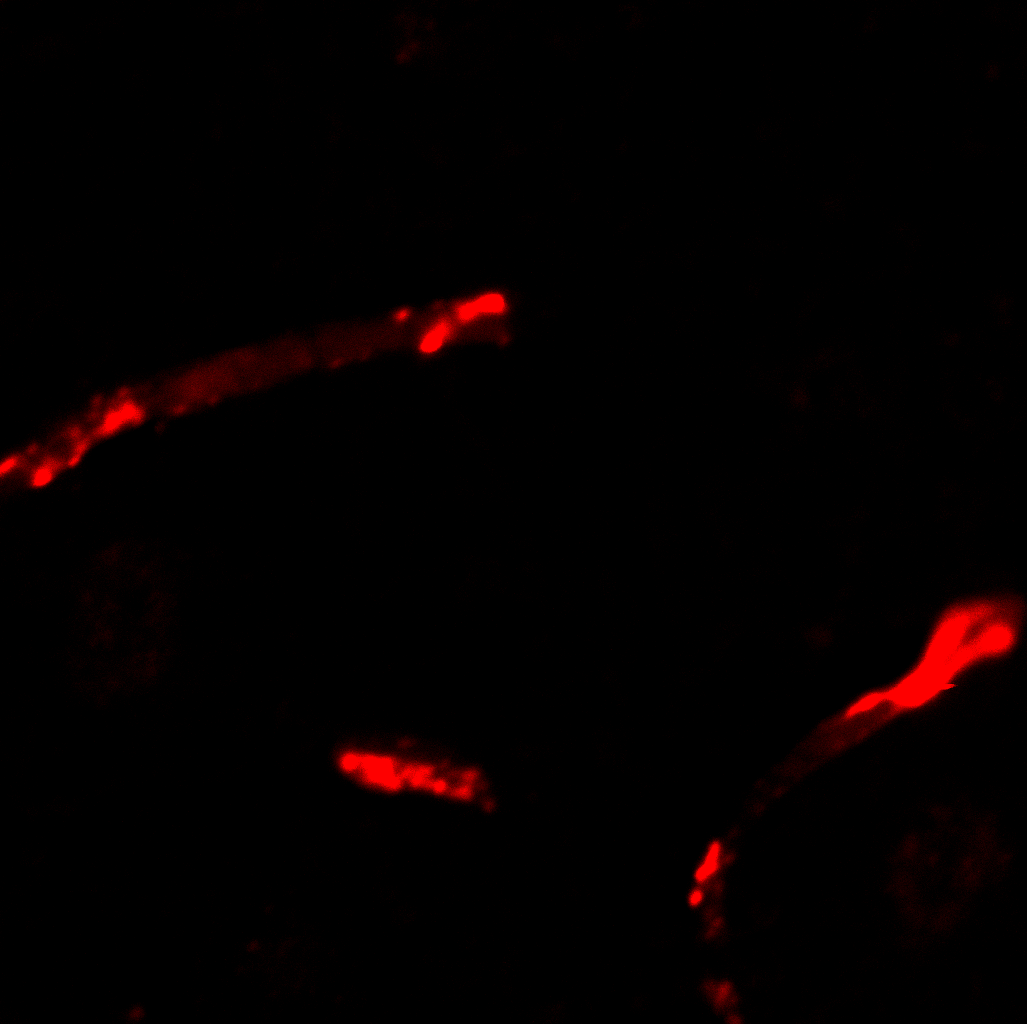

Supplement: Supplementary file 4 [file DataSheet8.ZIP › Immunofluorescence (Figure 6G, part3)/13/1/5s_c1.tif]

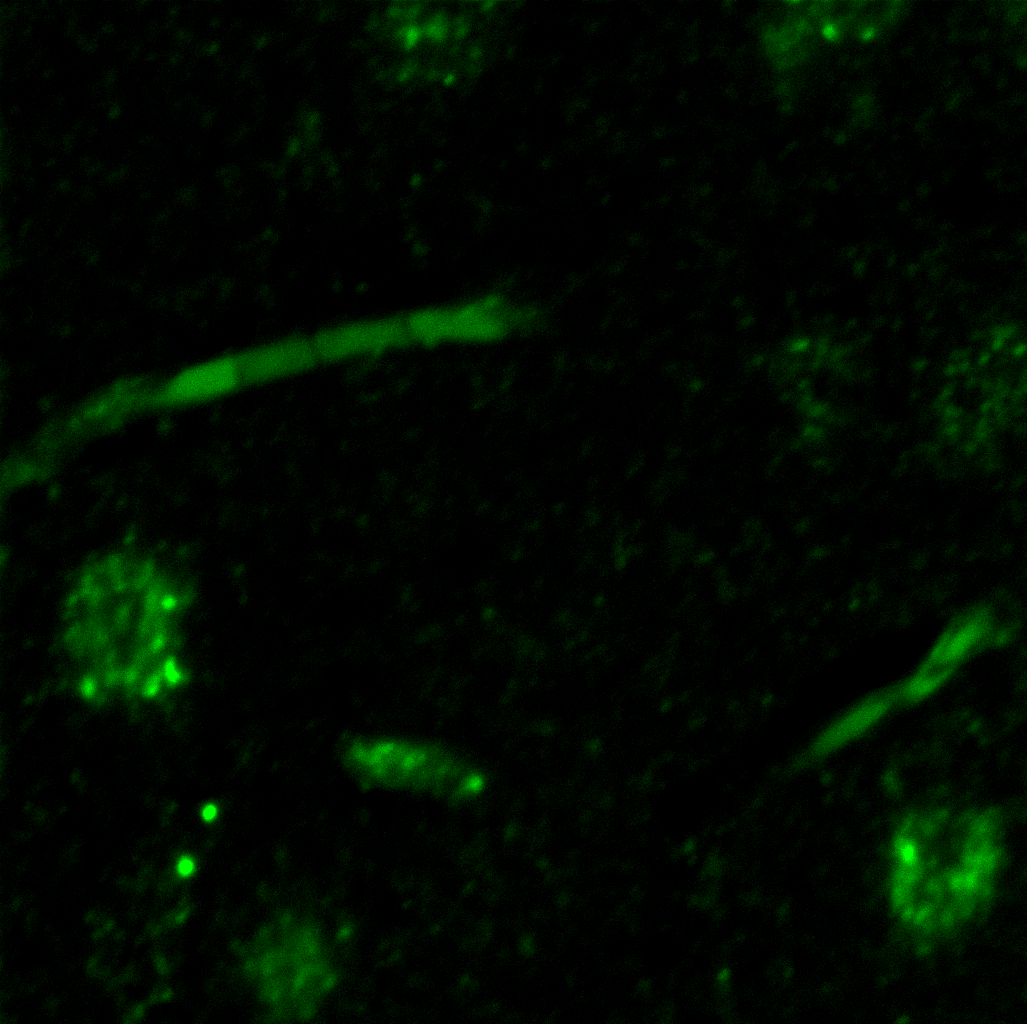

Supplement: Supplementary file 4 [file DataSheet8.ZIP › Immunofluorescence (Figure 6G, part3)/13/1/5s_c2.tif]

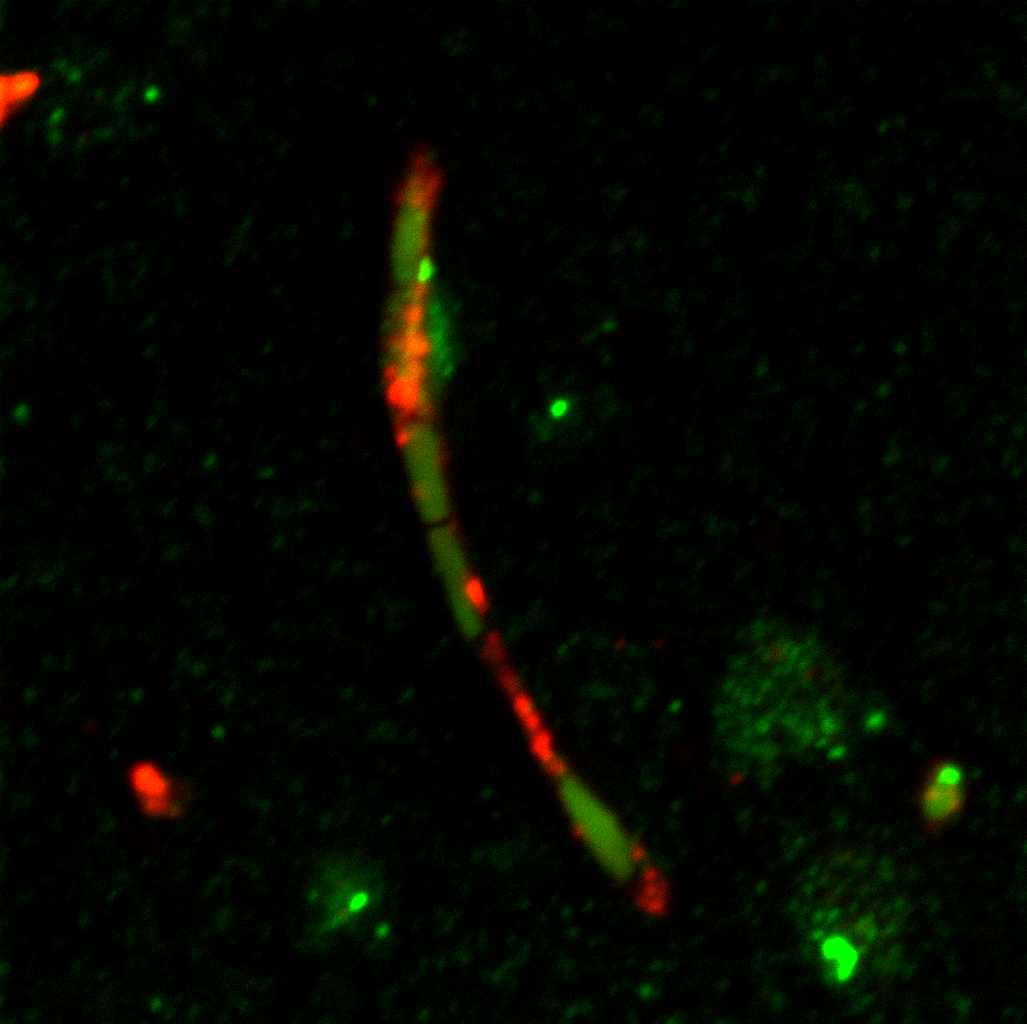

Supplement: Supplementary file 4 [file DataSheet8.ZIP › Immunofluorescence (Figure 6G, part3)/13/2/6s_c1+2.tif]

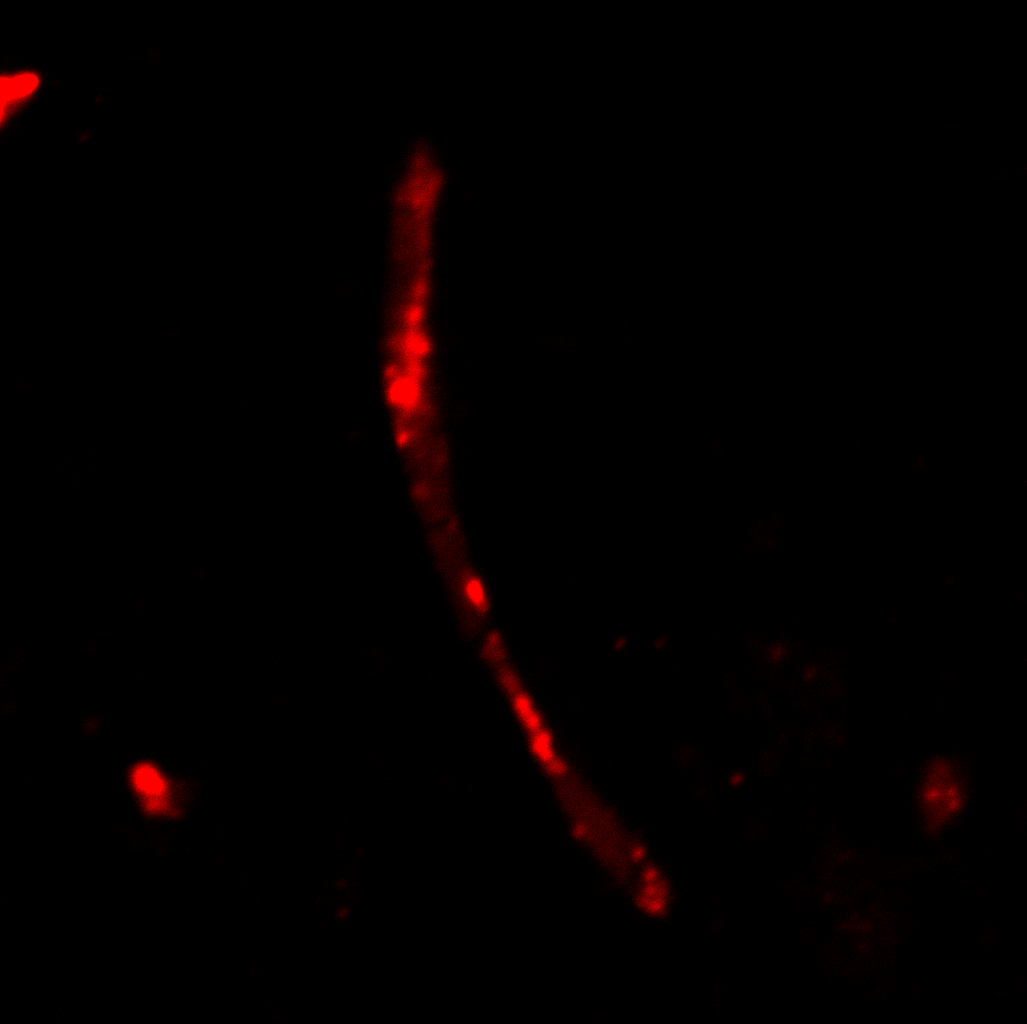

Supplement: Supplementary file 4 [file DataSheet8.ZIP › Immunofluorescence (Figure 6G, part3)/13/2/6s_c1.tif]

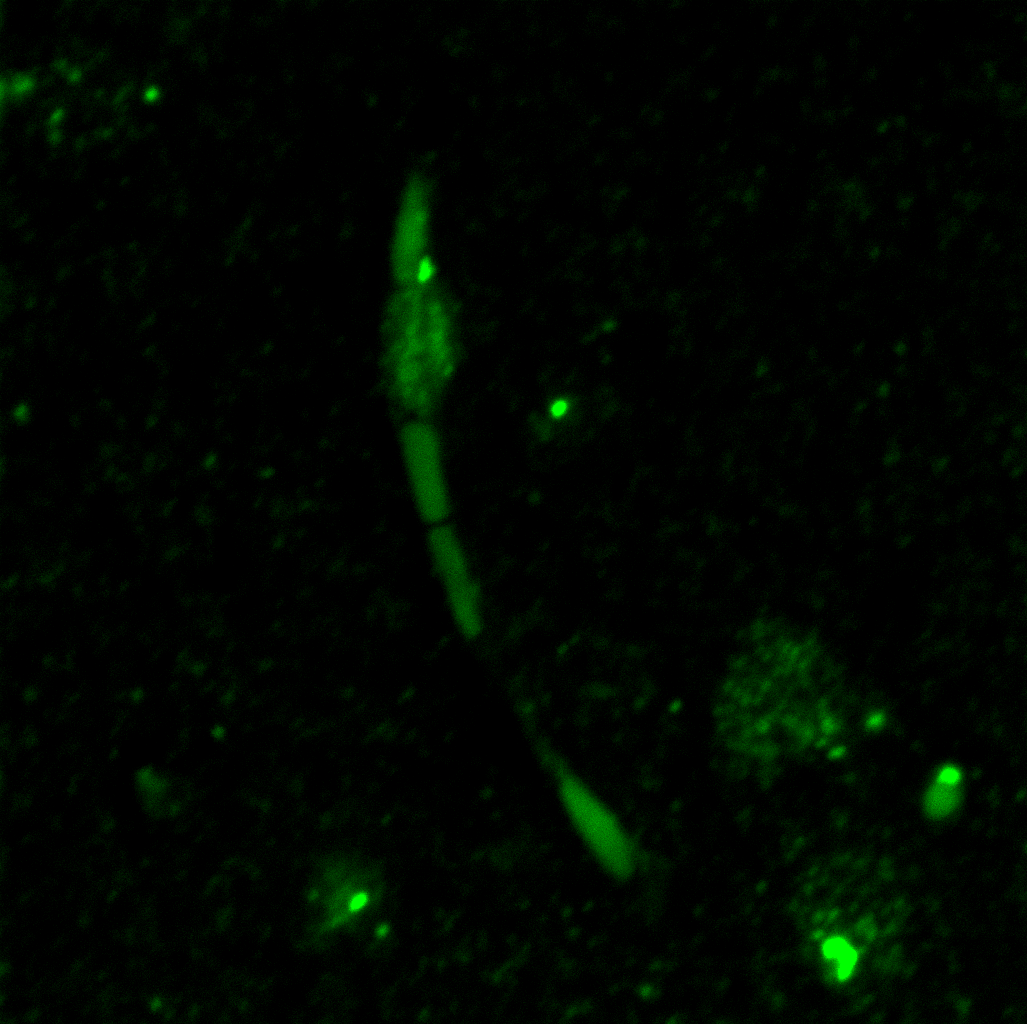

Supplement: Supplementary file 4 [file DataSheet8.ZIP › Immunofluorescence (Figure 6G, part3)/13/2/6s_c2.tif]

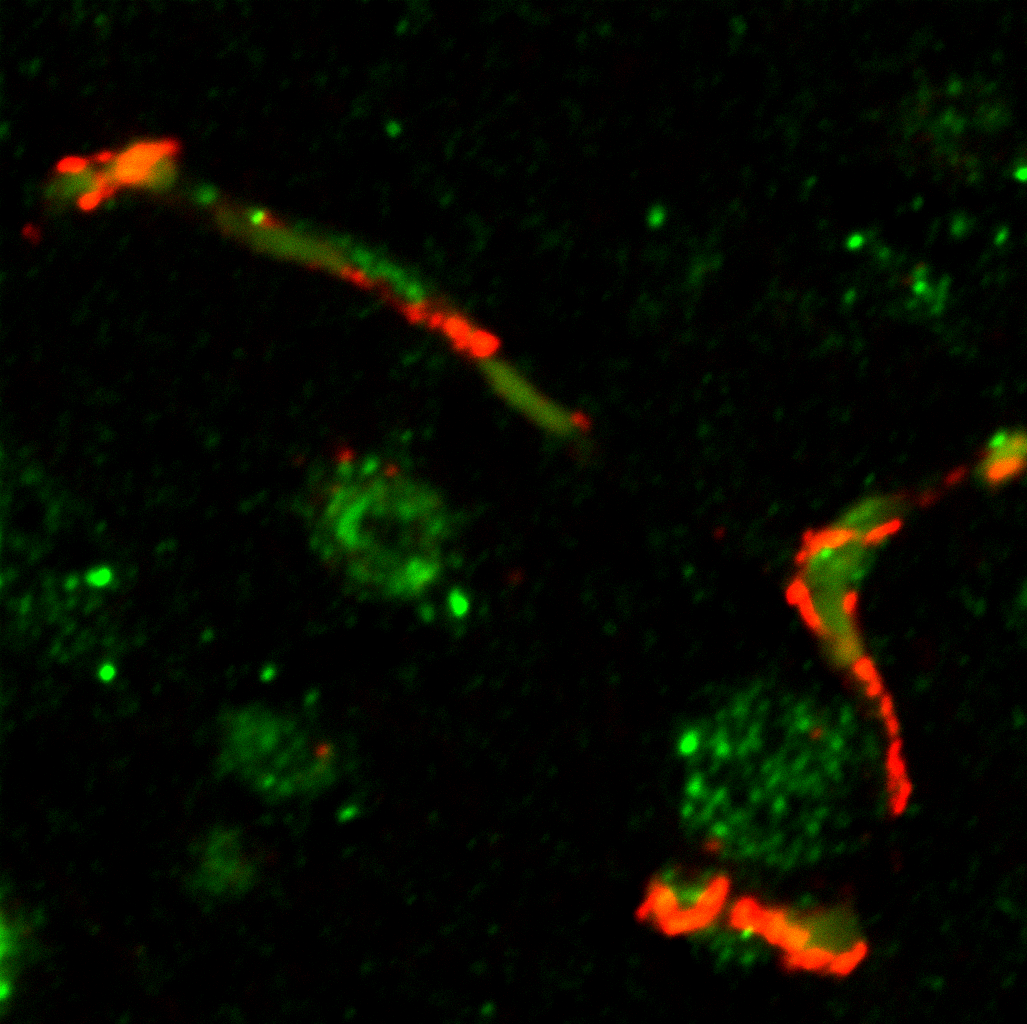

Supplement: Supplementary file 4 [file DataSheet8.ZIP › Immunofluorescence (Figure 6G, part3)/13/3/7s_c1+2.tif]

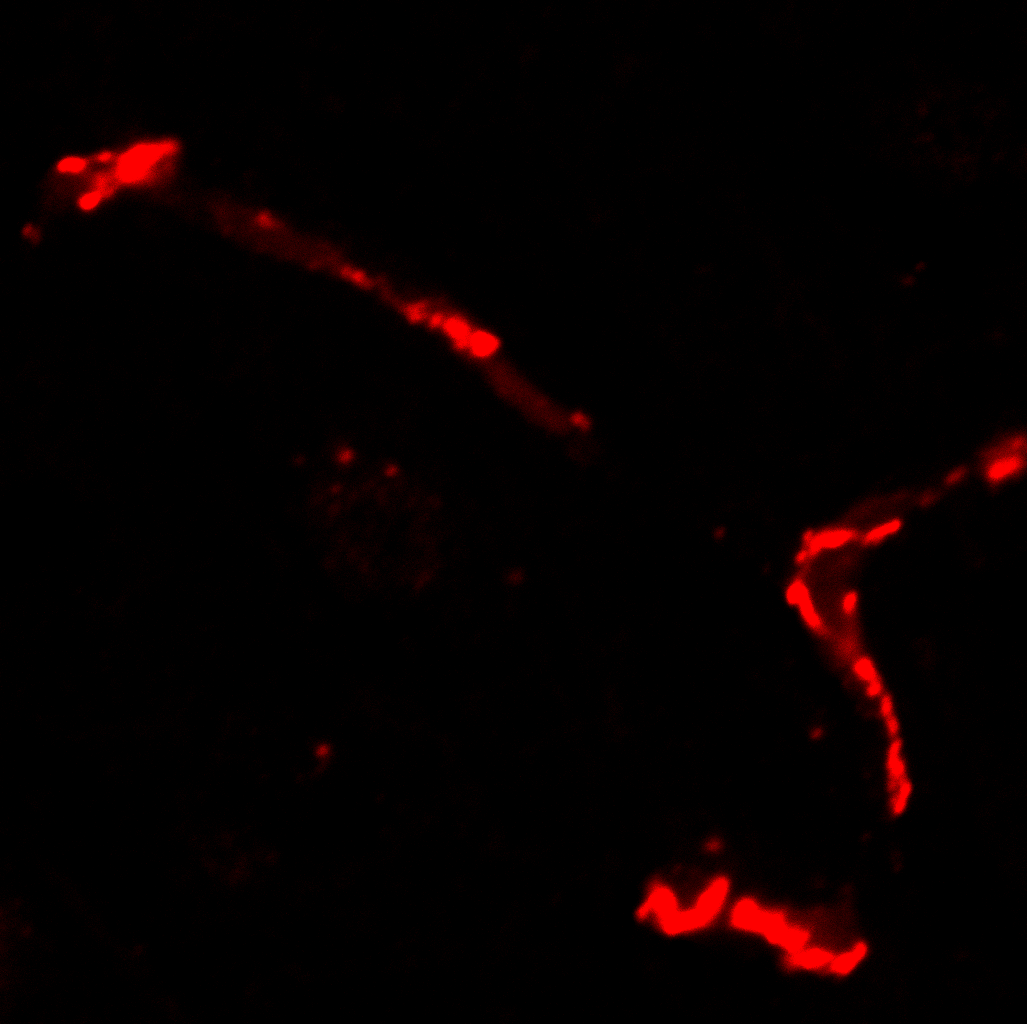

Supplement: Supplementary file 4 [file DataSheet8.ZIP › Immunofluorescence (Figure 6G, part3)/13/3/7s_c1.tif]

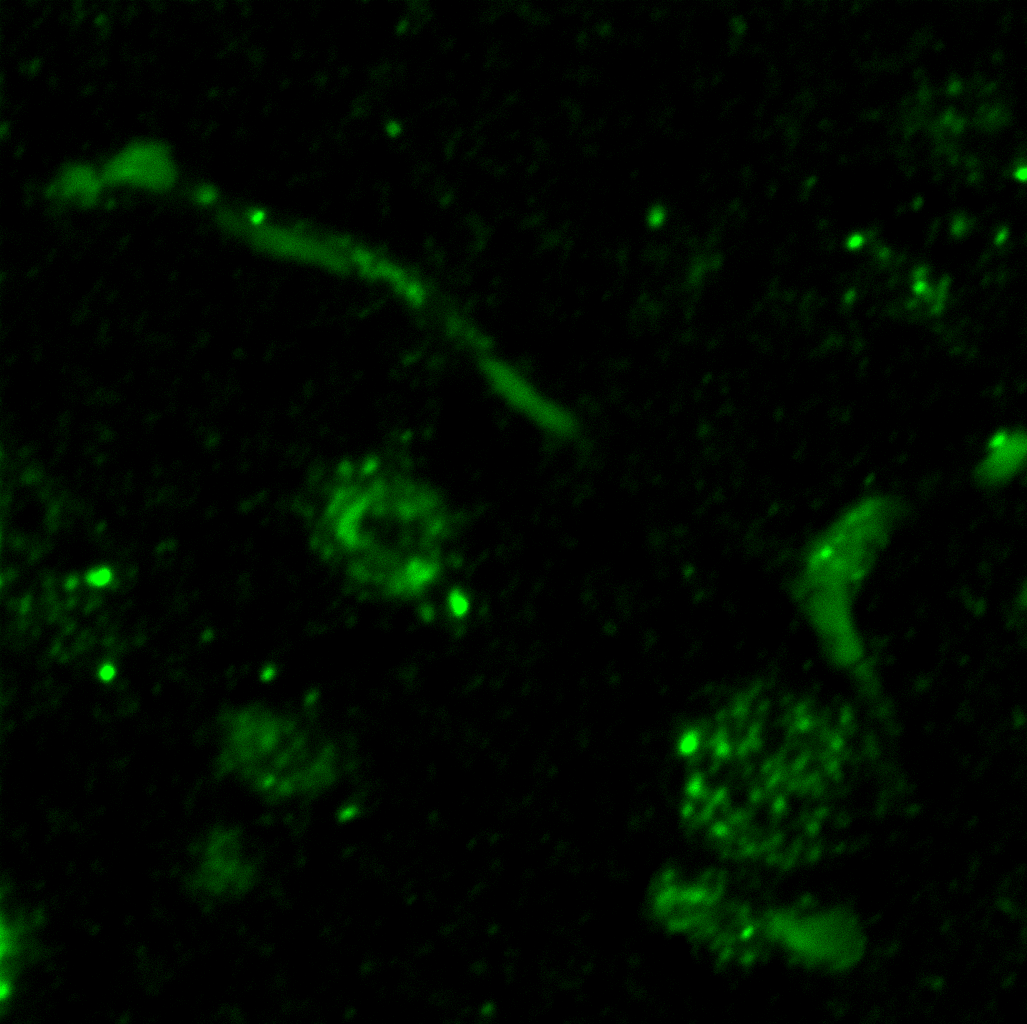

Supplement: Supplementary file 4 [file DataSheet8.ZIP › Immunofluorescence (Figure 6G, part3)/13/3/7s_c2.tif]

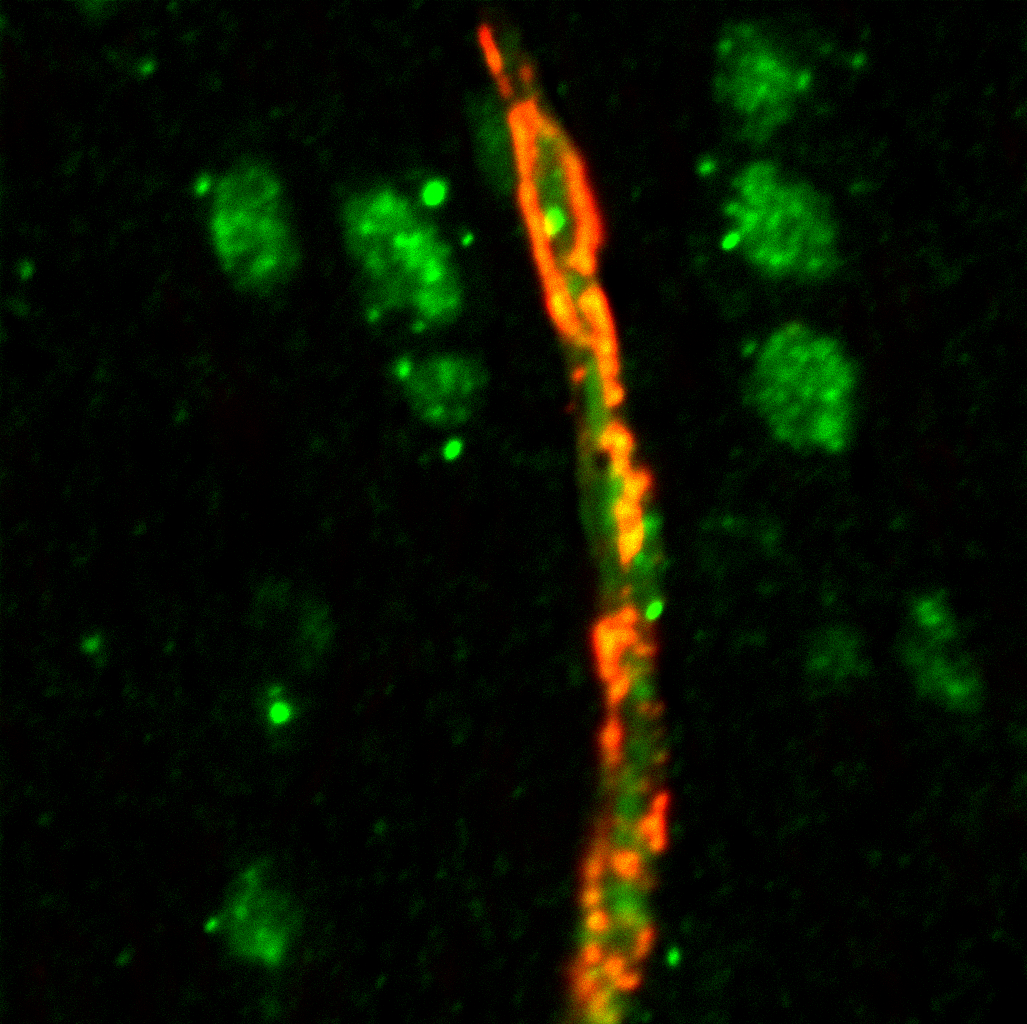

Supplement: Supplementary file 4 [file DataSheet8.ZIP › Immunofluorescence (Figure 6G, part3)/14 (presented in manuscript)/1 (presented in manuscript)/5S_c1+2.tif]

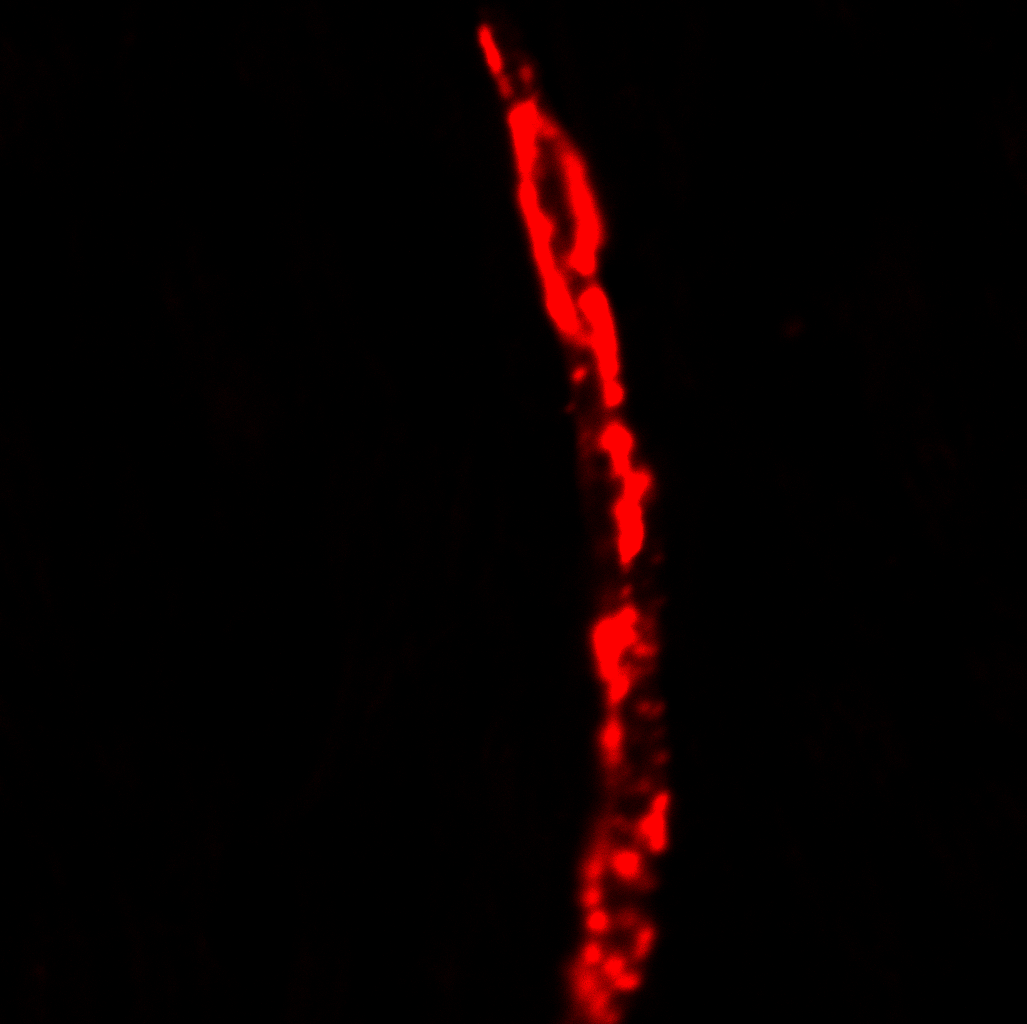

Supplement: Supplementary file 4 [file DataSheet8.ZIP › Immunofluorescence (Figure 6G, part3)/14 (presented in manuscript)/1 (presented in manuscript)/5S_c1.tif]

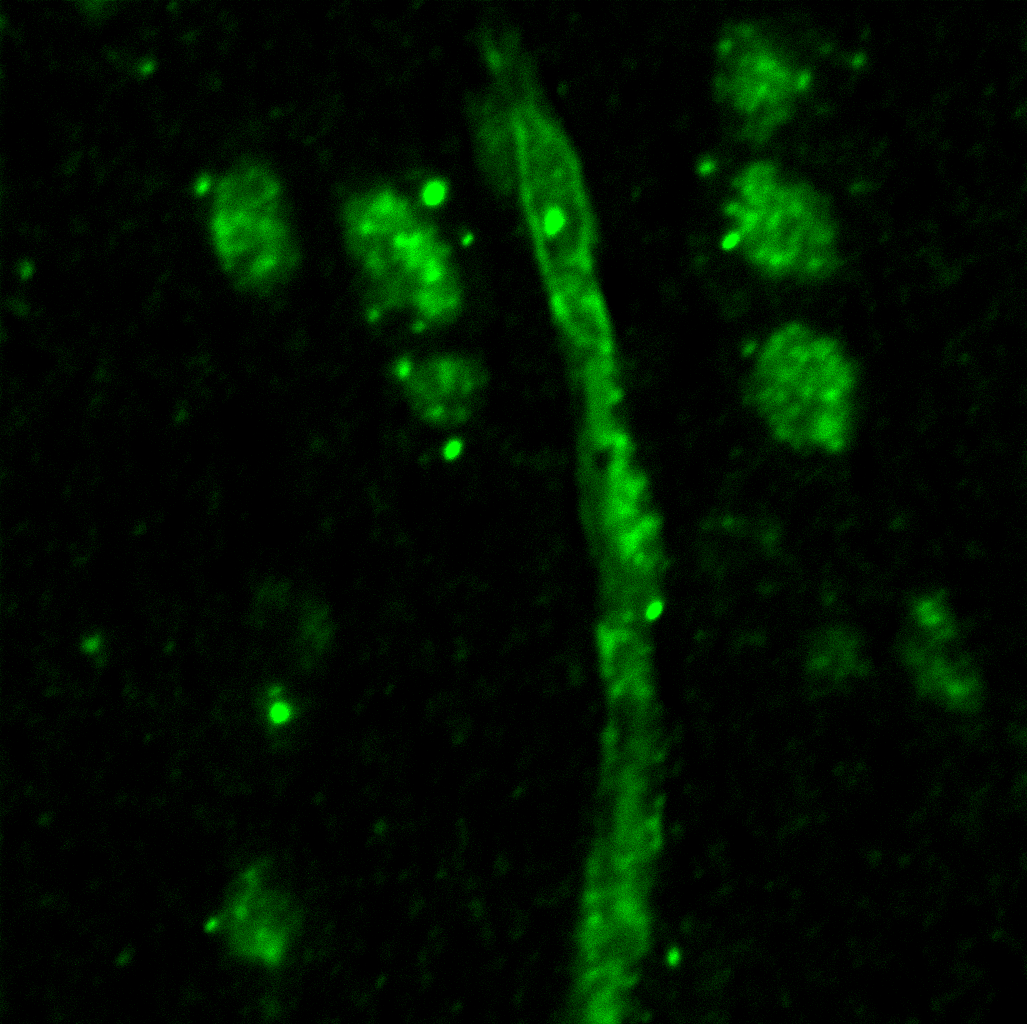

Supplement: Supplementary file 4 [file DataSheet8.ZIP › Immunofluorescence (Figure 6G, part3)/14 (presented in manuscript)/1 (presented in manuscript)/5S_c2.tif]

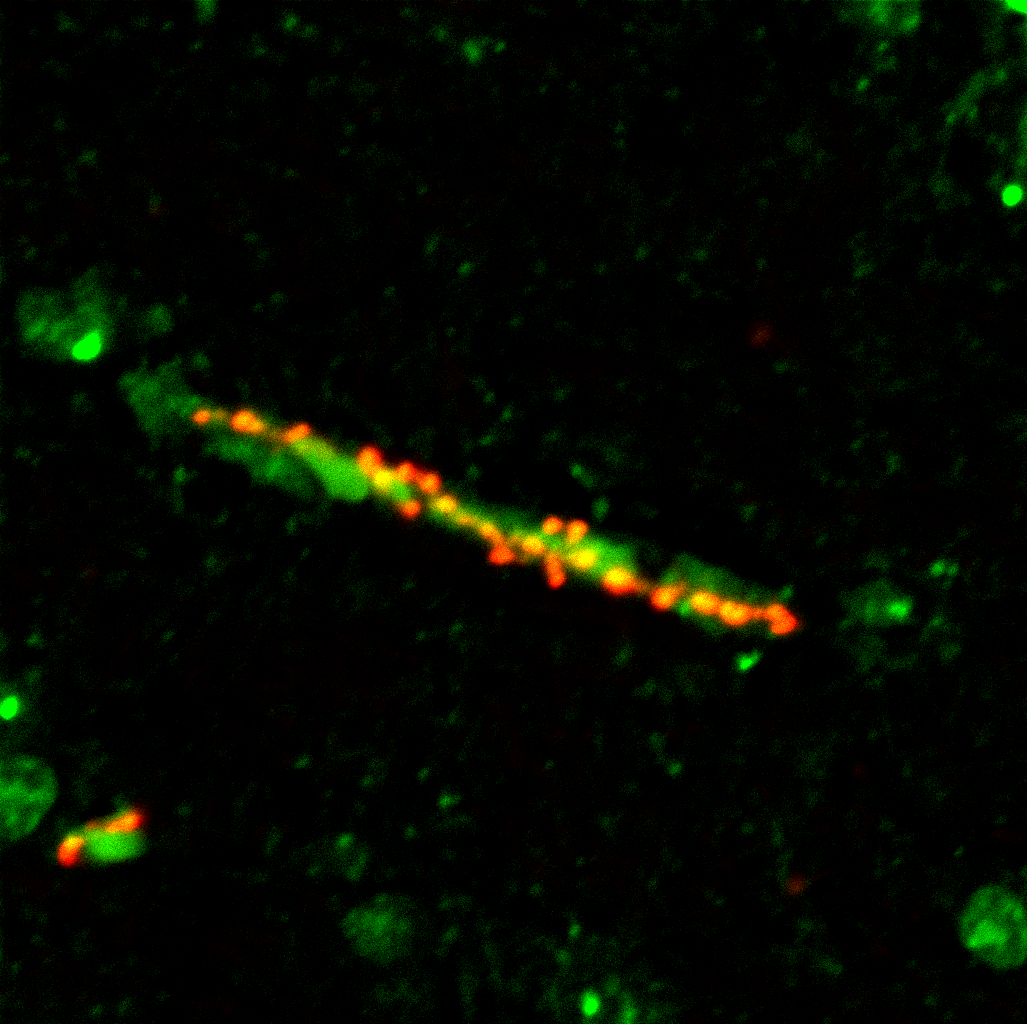

Supplement: Supplementary file 4 [file DataSheet8.ZIP › Immunofluorescence (Figure 6G, part3)/14 (presented in manuscript)/2/6S_c1+2.tif]

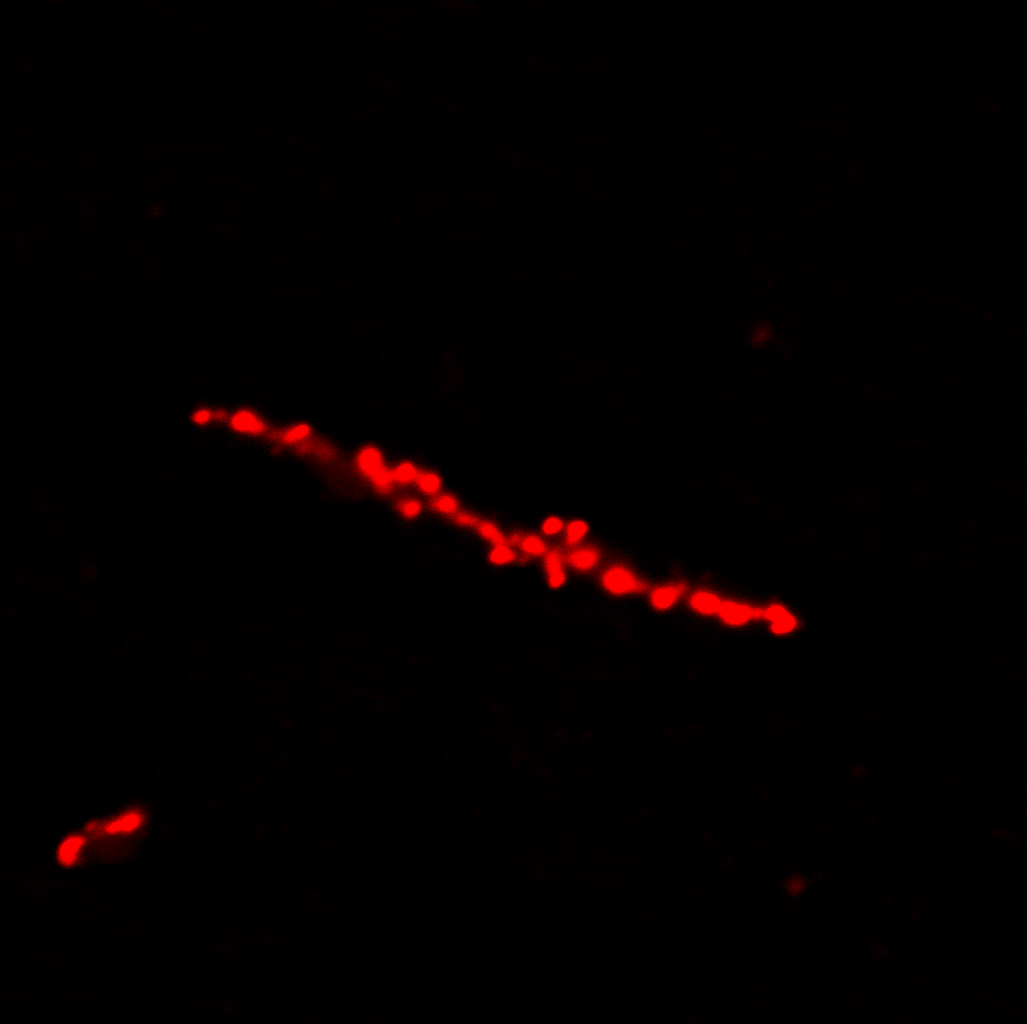

Supplement: Supplementary file 4 [file DataSheet8.ZIP › Immunofluorescence (Figure 6G, part3)/14 (presented in manuscript)/2/6S_c1.tif]

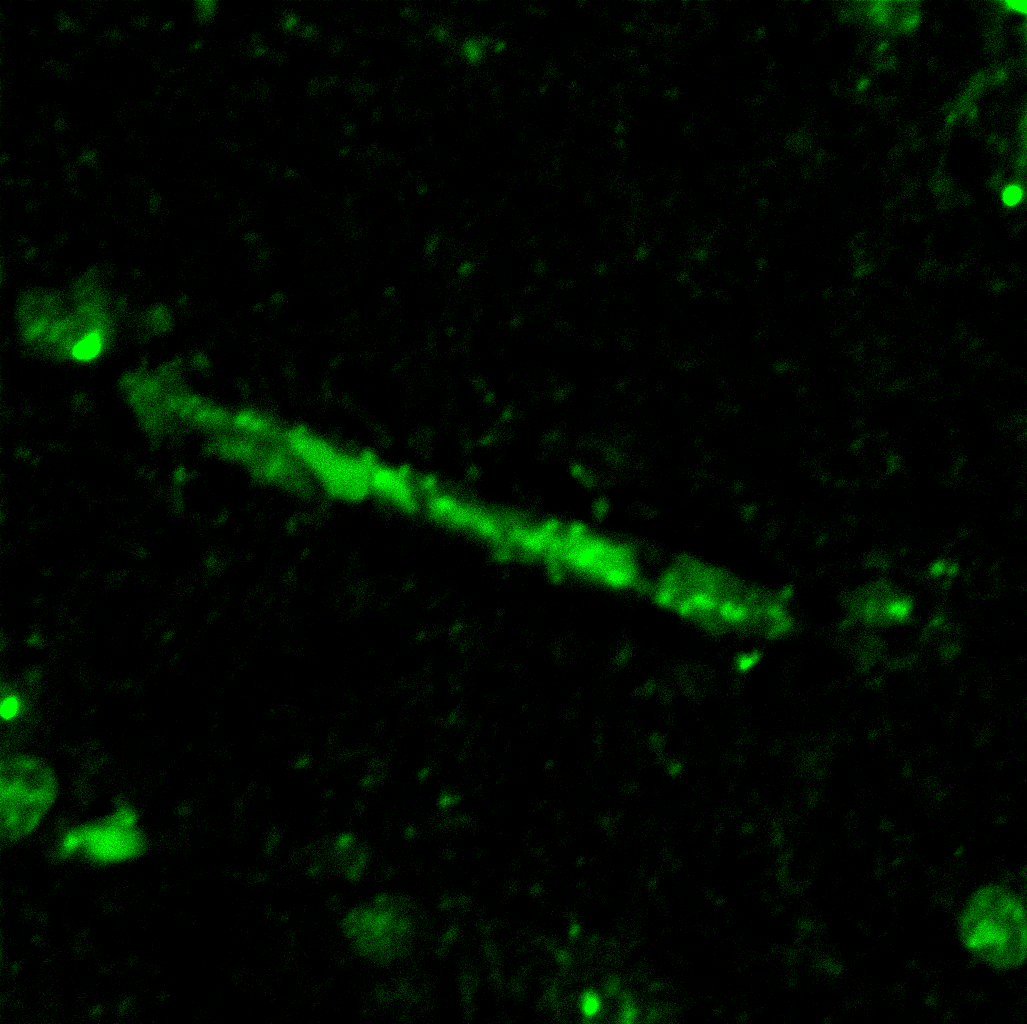

Supplement: Supplementary file 4 [file DataSheet8.ZIP › Immunofluorescence (Figure 6G, part3)/14 (presented in manuscript)/2/6S_c2.tif]

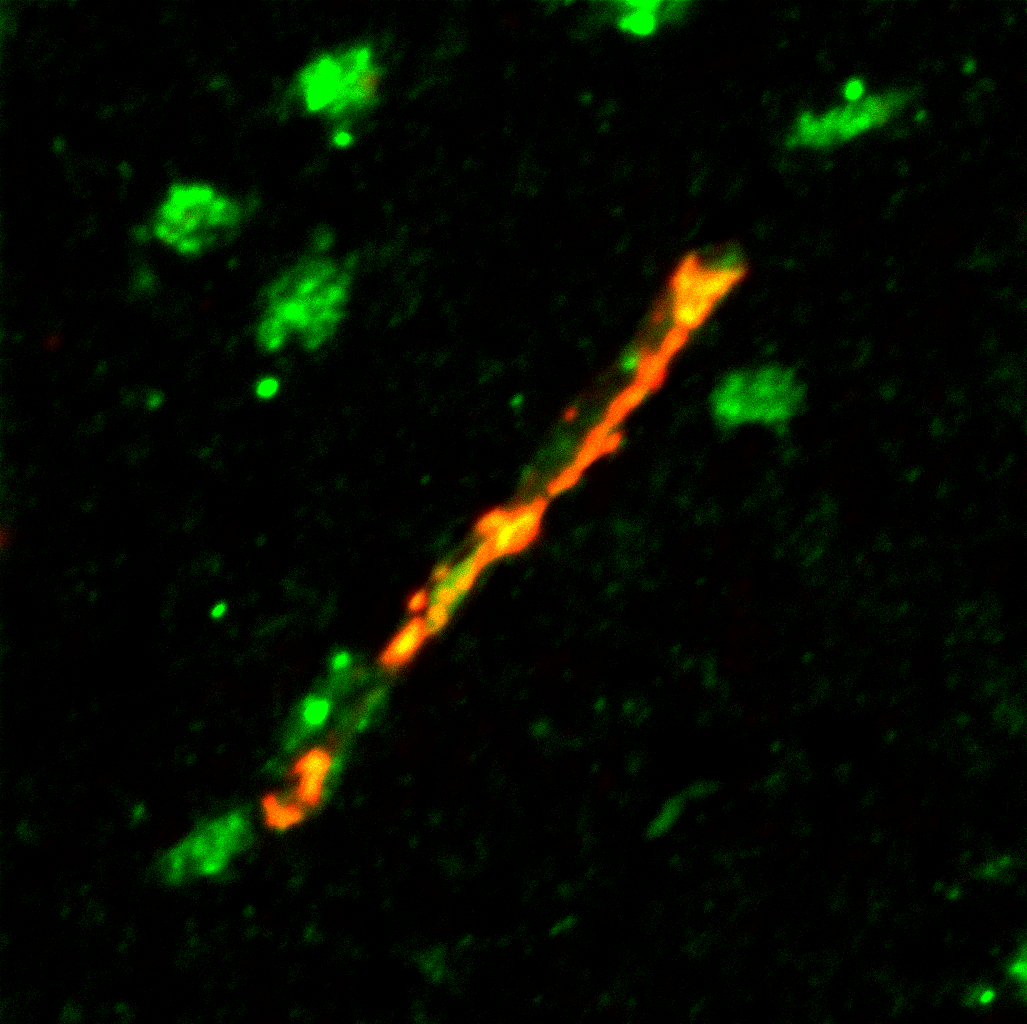

Supplement: Supplementary file 4 [file DataSheet8.ZIP › Immunofluorescence (Figure 6G, part3)/14 (presented in manuscript)/3/7S_c1+2.tif]

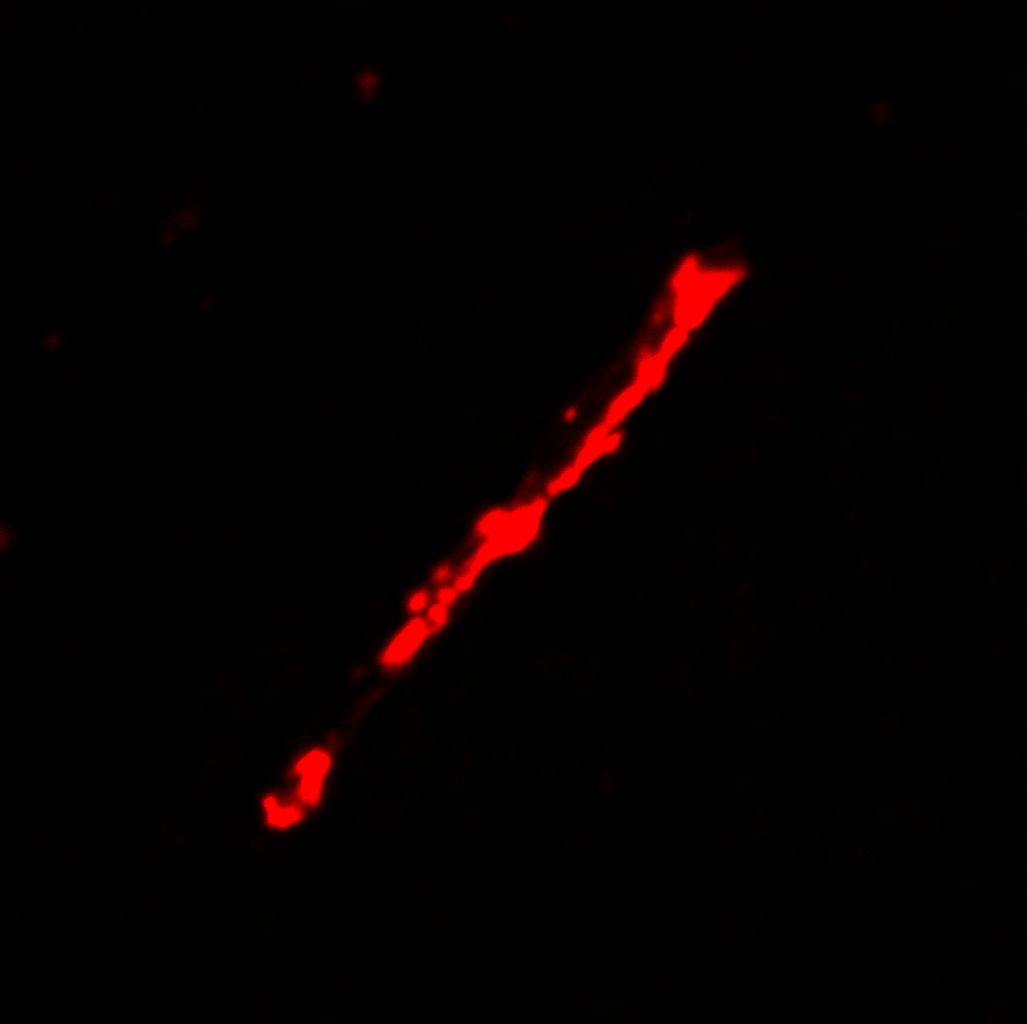

Supplement: Supplementary file 4 [file DataSheet8.ZIP › Immunofluorescence (Figure 6G, part3)/14 (presented in manuscript)/3/7S_c1.tif]

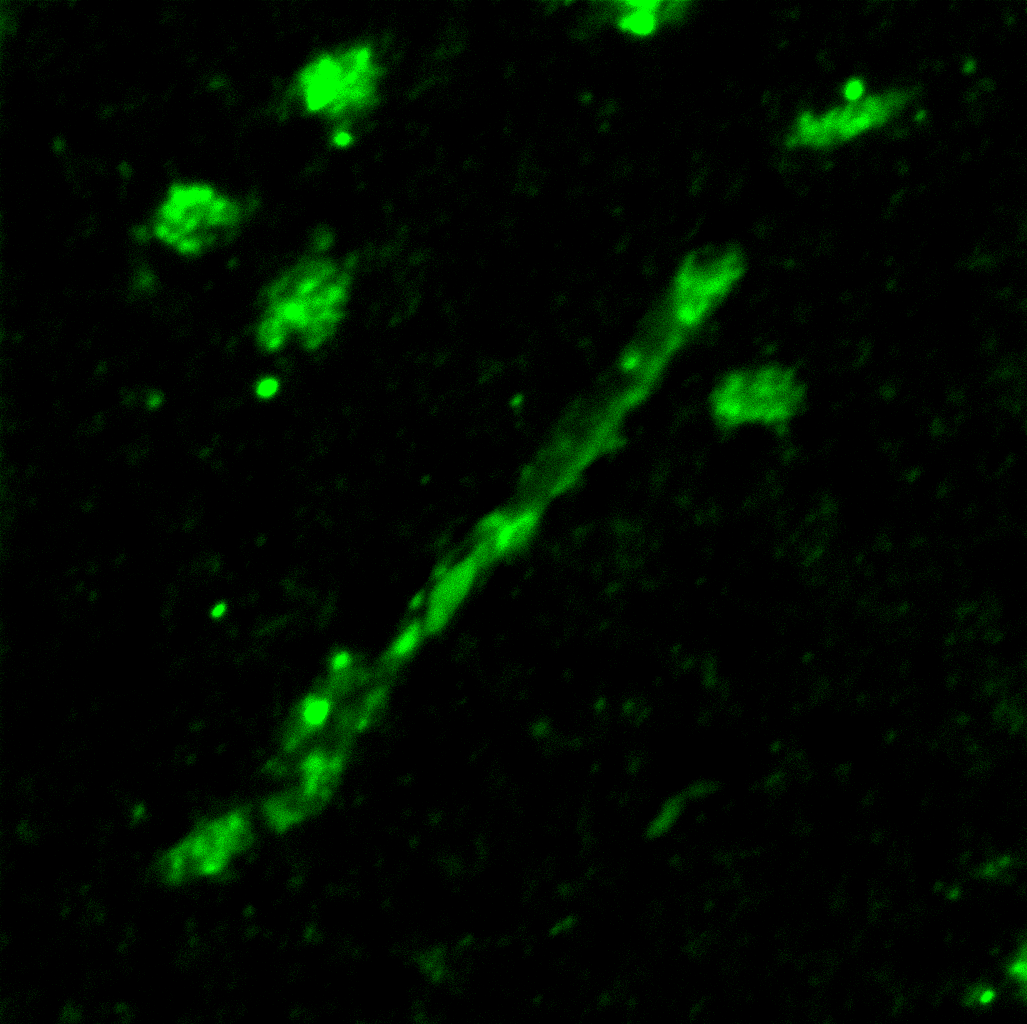

Supplement: Supplementary file 4 [file DataSheet8.ZIP › Immunofluorescence (Figure 6G, part3)/14 (presented in manuscript)/3/7S_c2.tif]

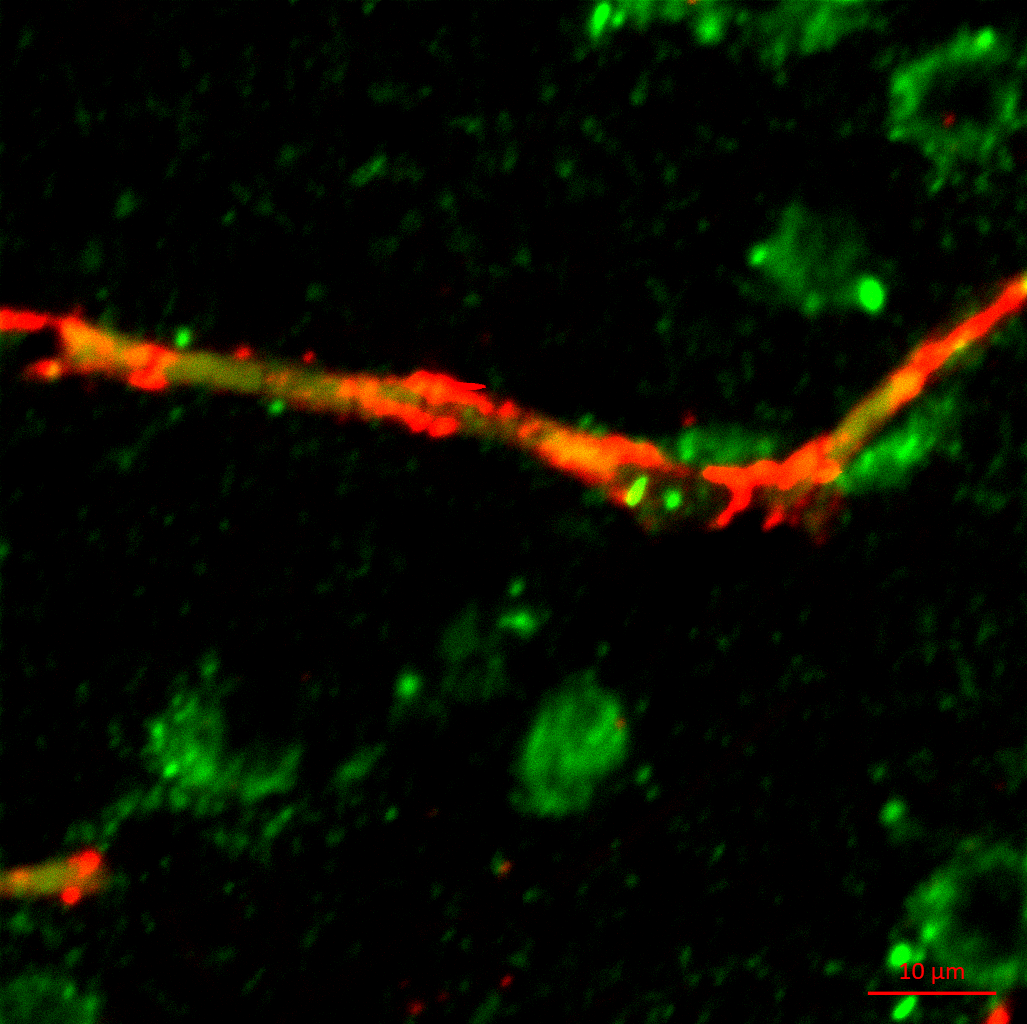

Supplement: Supplementary file 4 [file DataSheet8.ZIP › Immunofluorescence (Figure 6G, part3)/15/1/1S_c1+2.tif]

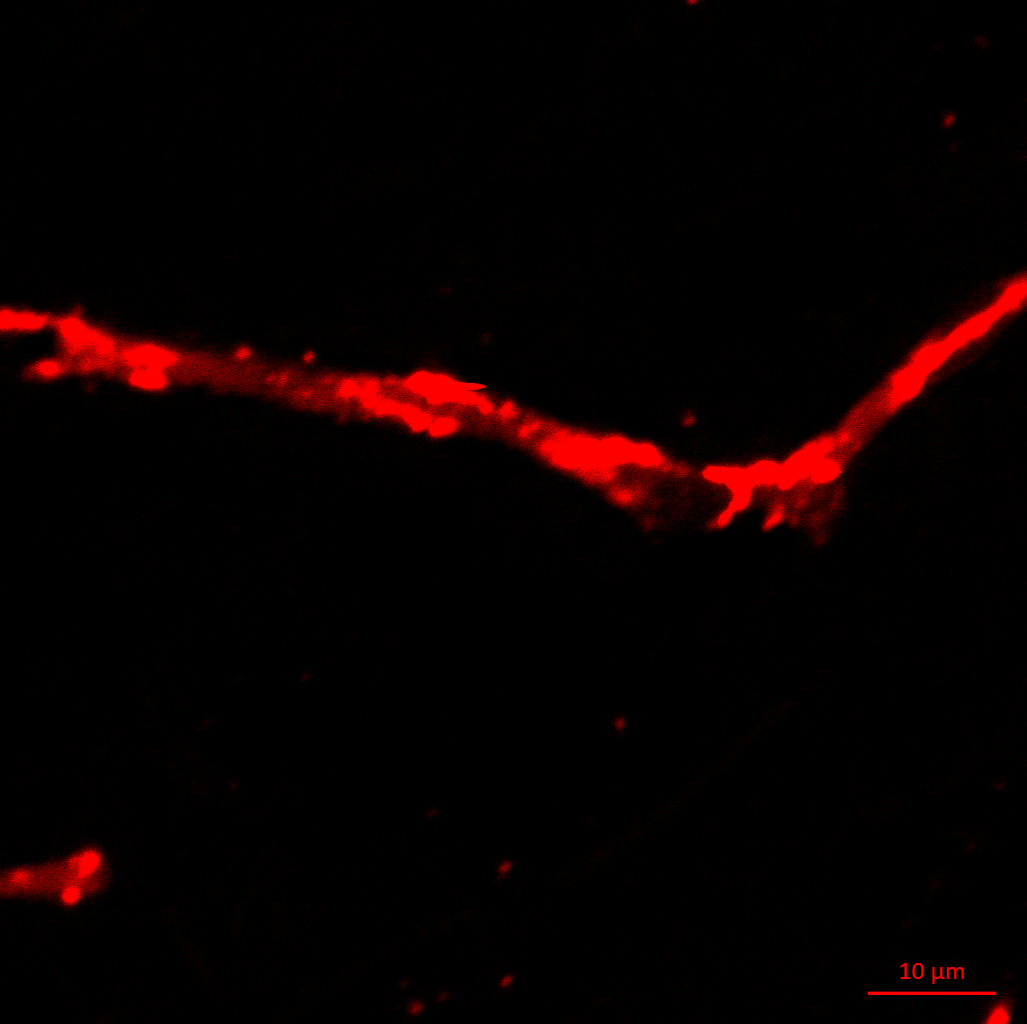

Supplement: Supplementary file 4 [file DataSheet8.ZIP › Immunofluorescence (Figure 6G, part3)/15/1/1S_c1.tif]

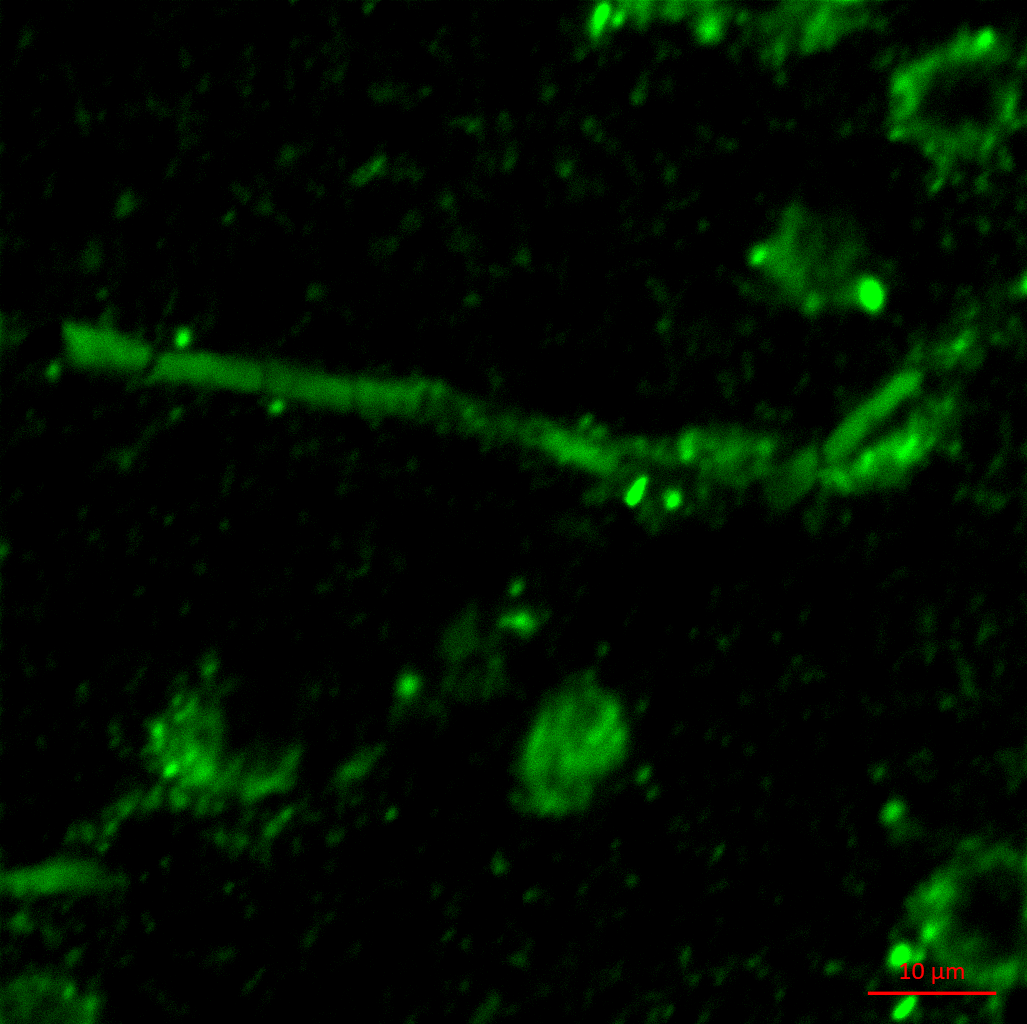

Supplement: Supplementary file 4 [file DataSheet8.ZIP › Immunofluorescence (Figure 6G, part3)/15/1/1S_c2.tif]

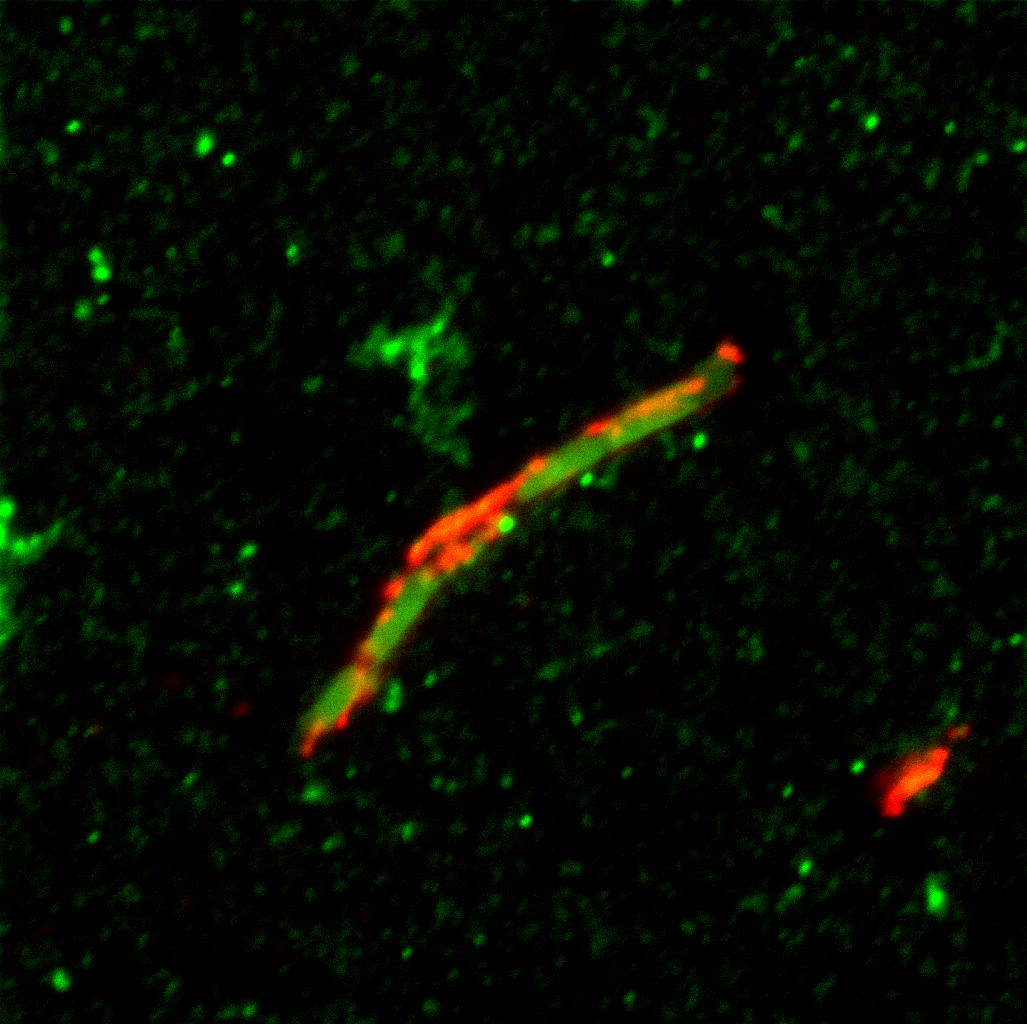

Supplement: Supplementary file 4 [file DataSheet8.ZIP › Immunofluorescence (Figure 6G, part3)/15/2/2S_c1+2.tif]

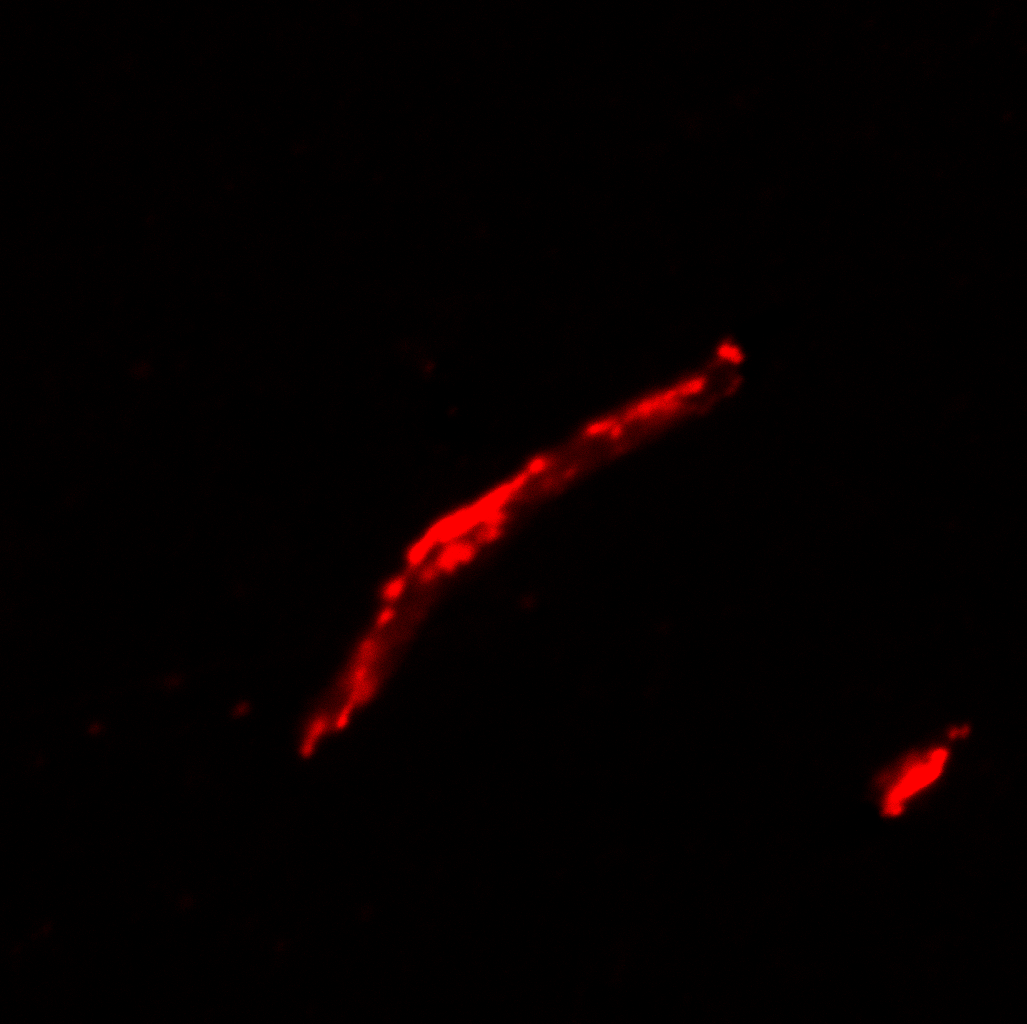

Supplement: Supplementary file 4 [file DataSheet8.ZIP › Immunofluorescence (Figure 6G, part3)/15/2/2S_c1.tif]

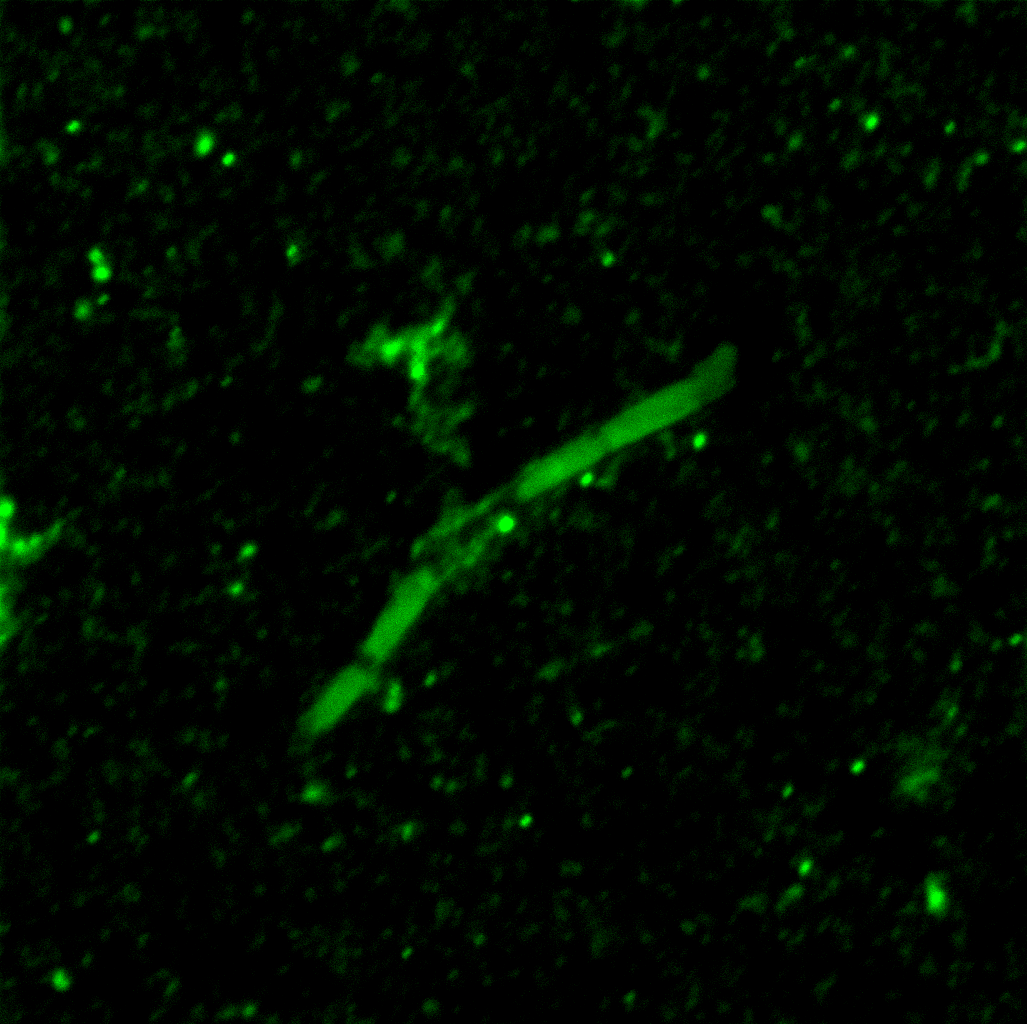

Supplement: Supplementary file 4 [file DataSheet8.ZIP › Immunofluorescence (Figure 6G, part3)/15/2/2S_c2.tif]

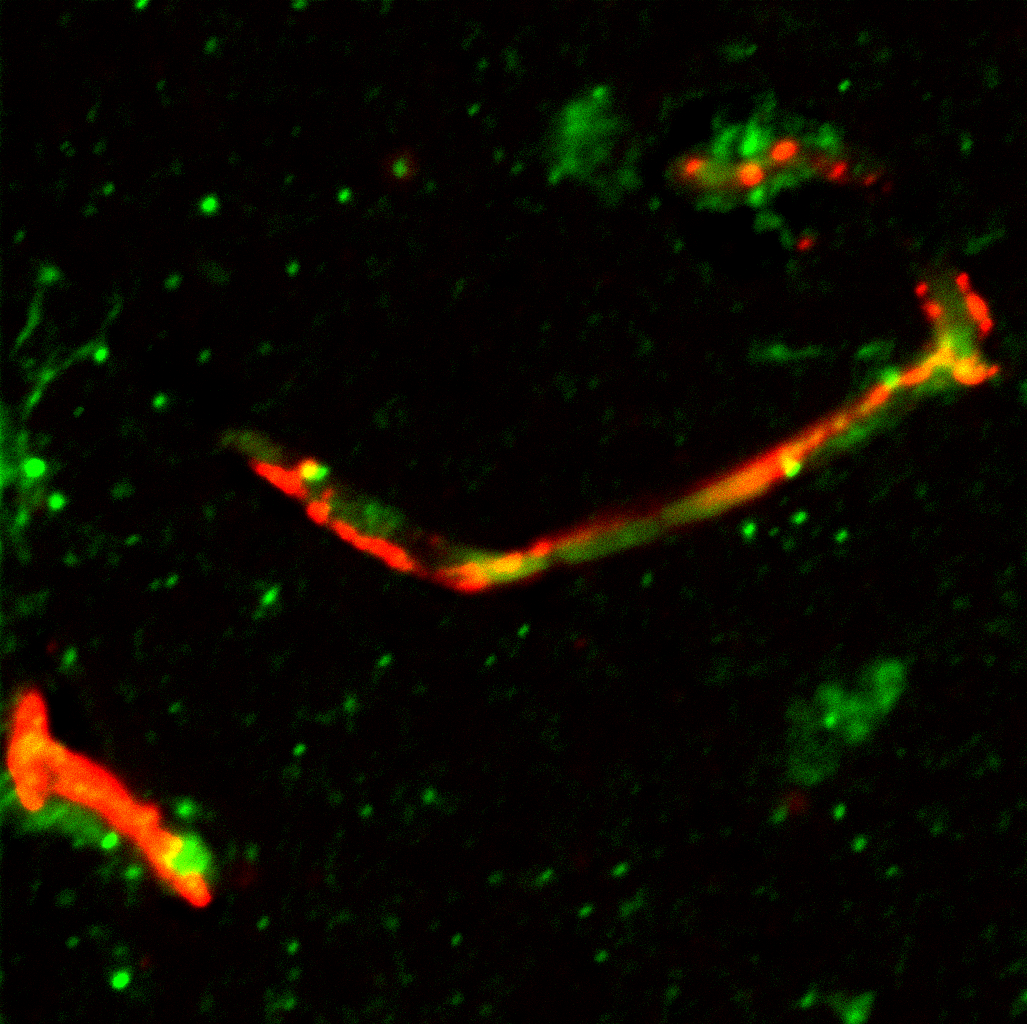

Supplement: Supplementary file 4 [file DataSheet8.ZIP › Immunofluorescence (Figure 6G, part3)/15/3/3S_c1+2.tif]

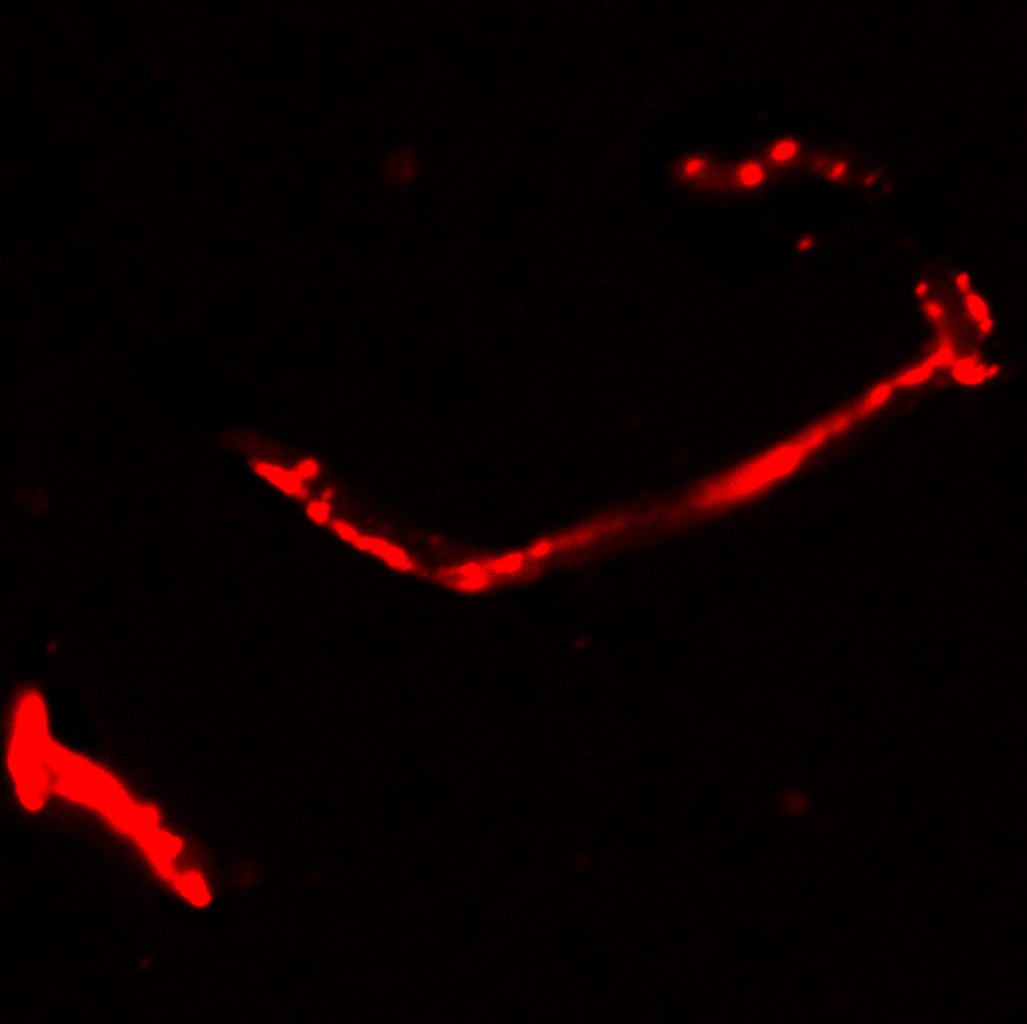

Supplement: Supplementary file 4 [file DataSheet8.ZIP › Immunofluorescence (Figure 6G, part3)/15/3/3S_c1.tif]

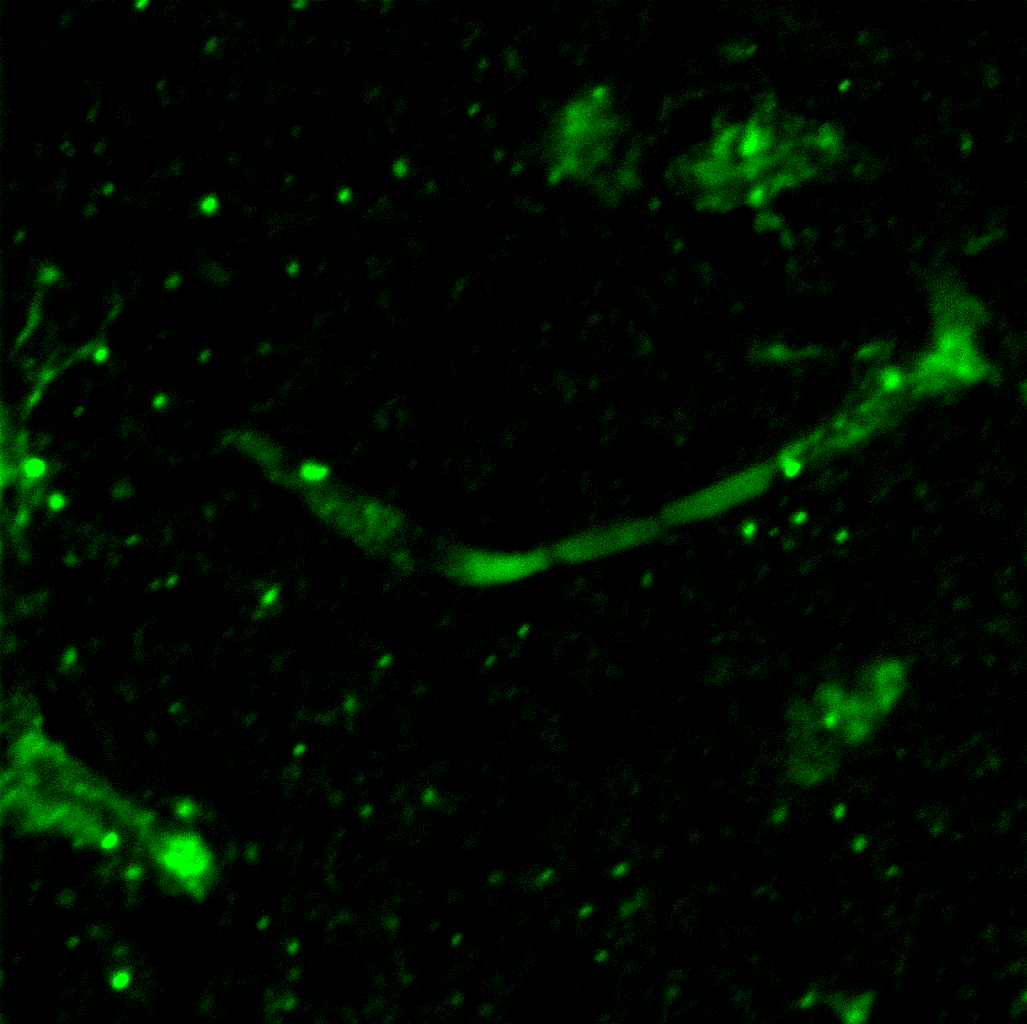

Supplement: Supplementary file 4 [file DataSheet8.ZIP › Immunofluorescence (Figure 6G, part3)/15/3/3S_c2.tif]

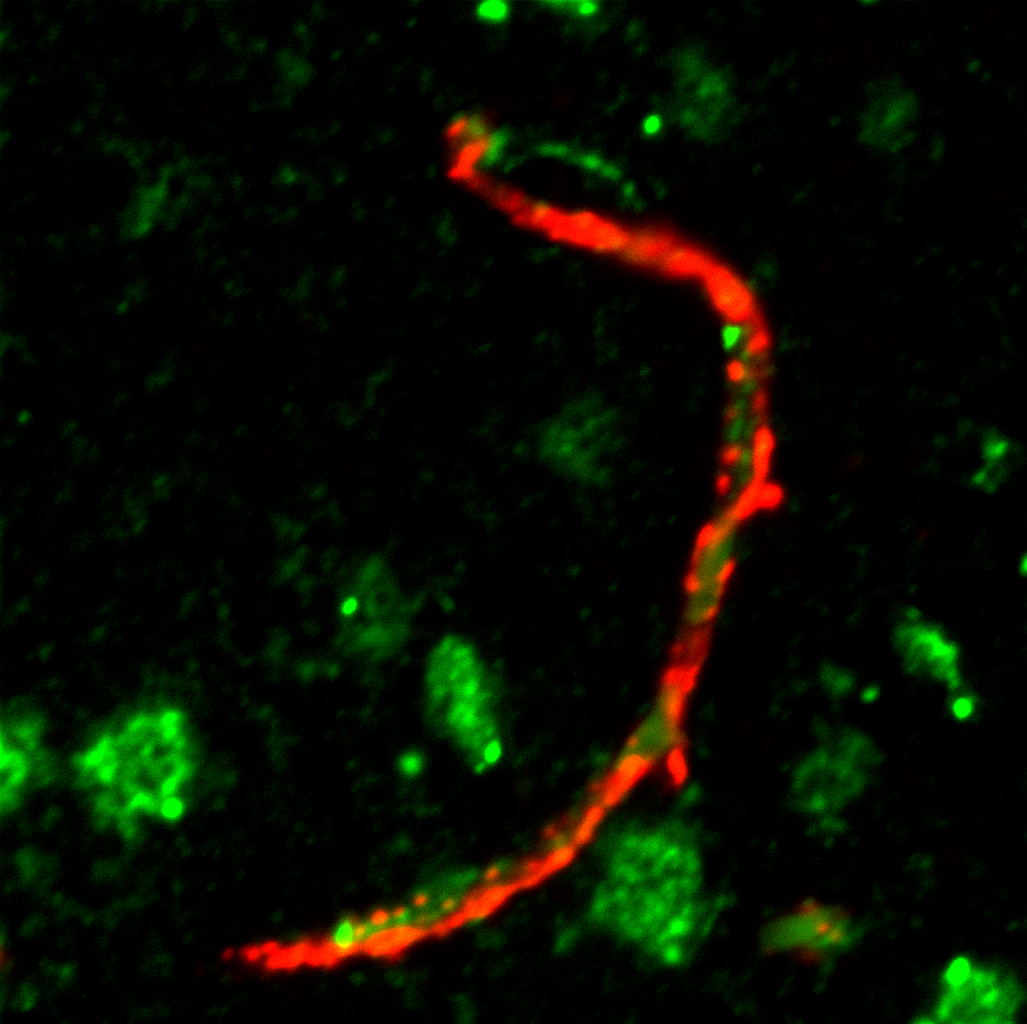

Supplement: Supplementary file 5 [file DataSheet9.ZIP › Immunofluorescence (Figure 6G, part4)/16/1/2s_c1+2.tif]

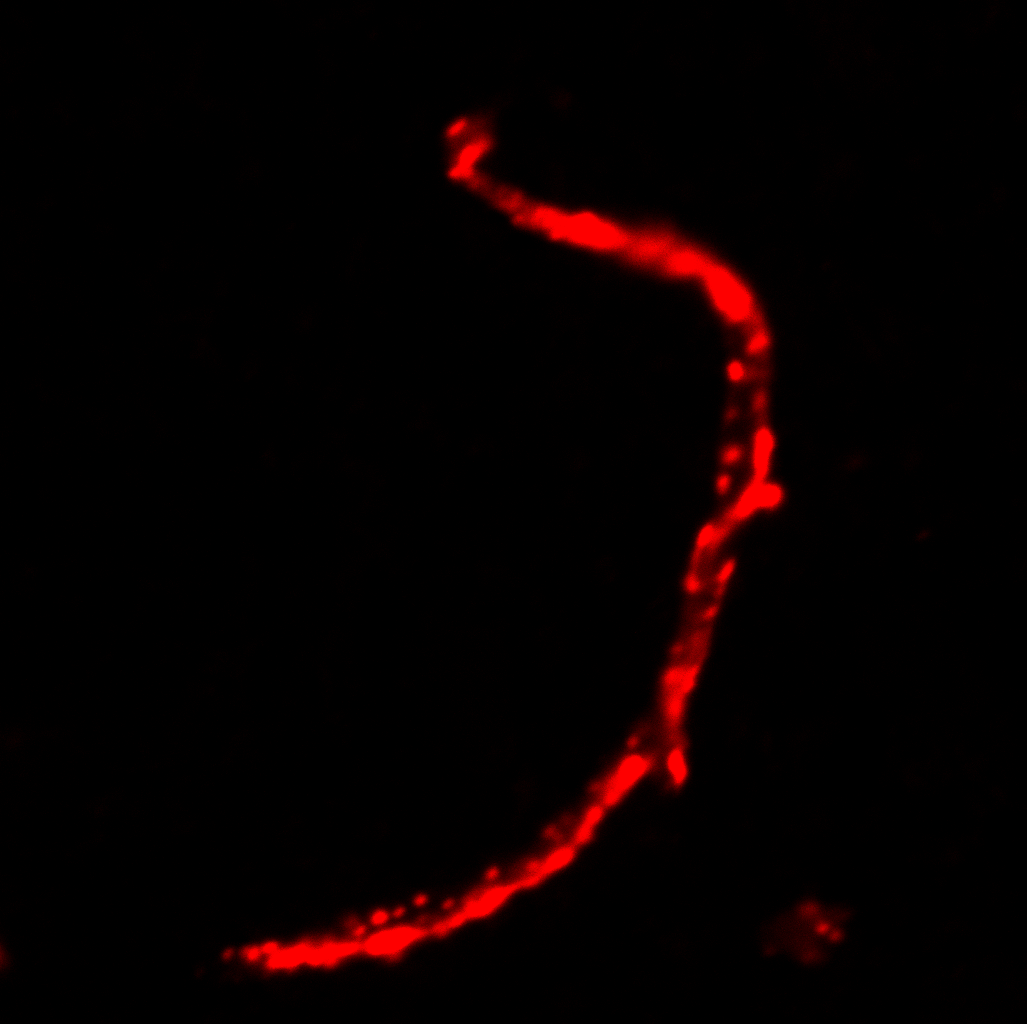

Supplement: Supplementary file 5 [file DataSheet9.ZIP › Immunofluorescence (Figure 6G, part4)/16/1/2s_c1.tif]

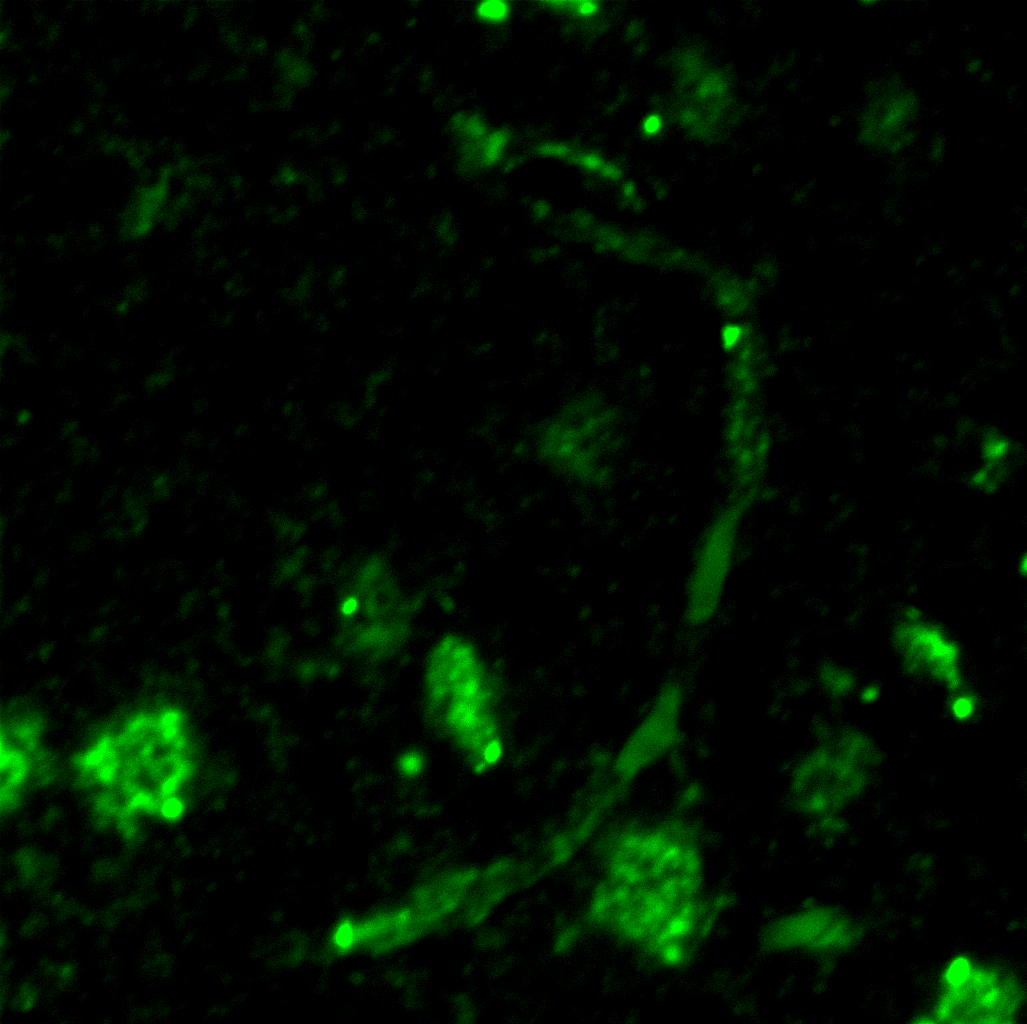

Supplement: Supplementary file 5 [file DataSheet9.ZIP › Immunofluorescence (Figure 6G, part4)/16/1/2s_c2.tif]

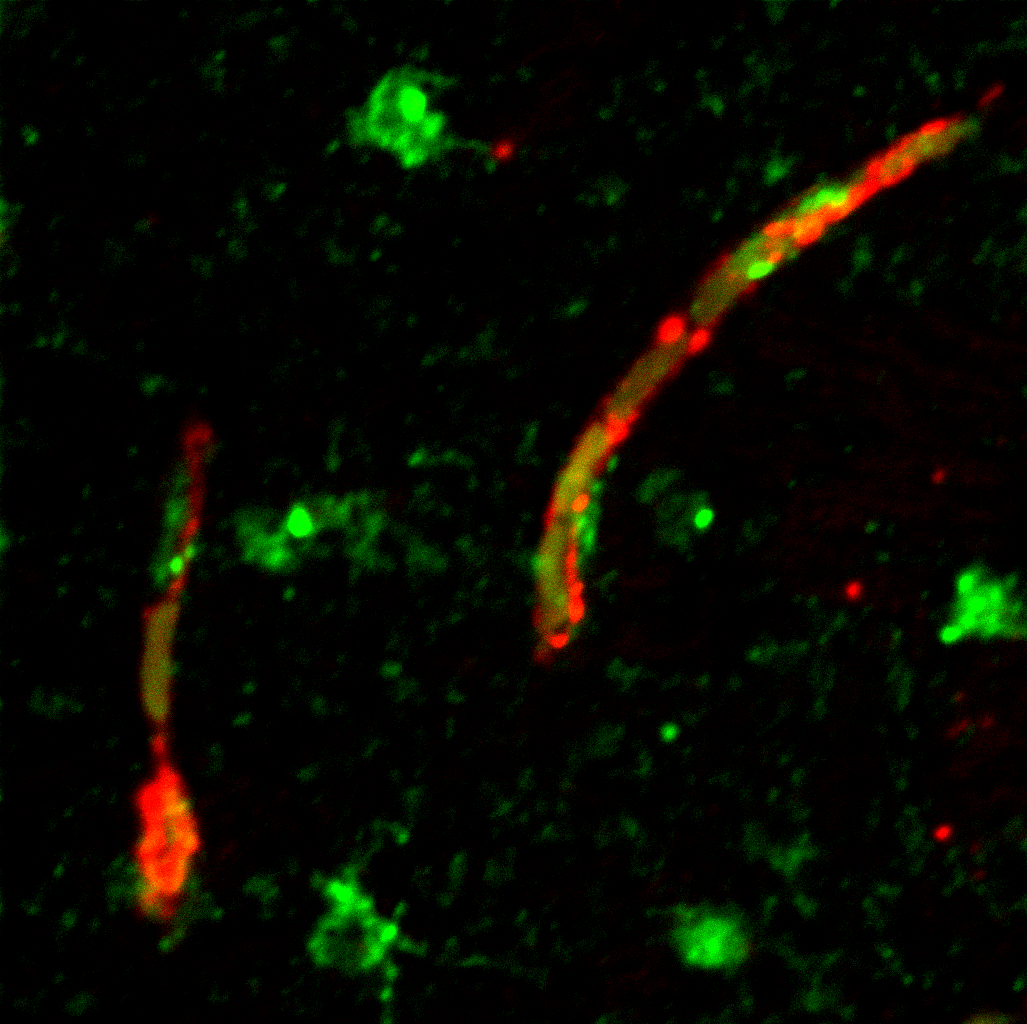

Supplement: Supplementary file 5 [file DataSheet9.ZIP › Immunofluorescence (Figure 6G, part4)/16/2/5s_c1+2.tif]

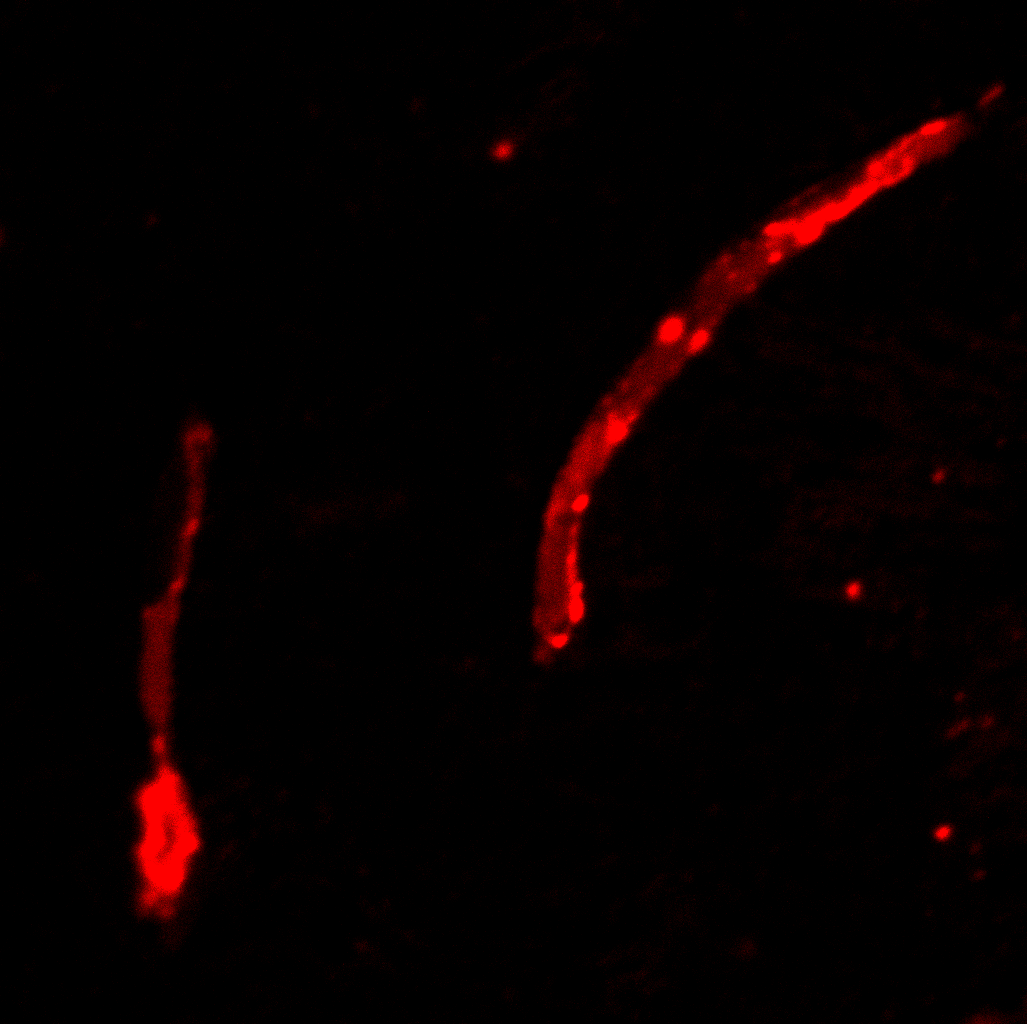

Supplement: Supplementary file 5 [file DataSheet9.ZIP › Immunofluorescence (Figure 6G, part4)/16/2/5s_c1.tif]

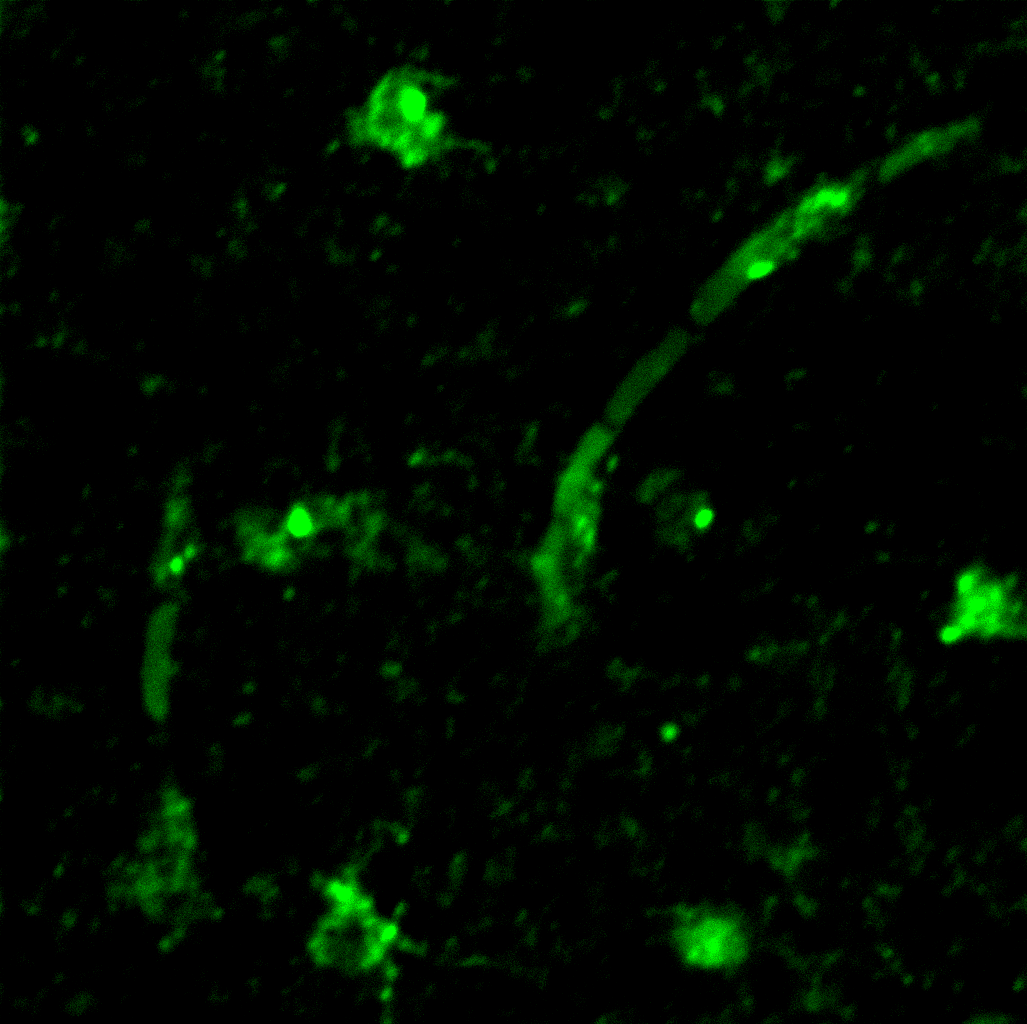

Supplement: Supplementary file 5 [file DataSheet9.ZIP › Immunofluorescence (Figure 6G, part4)/16/2/5s_c2.tif]

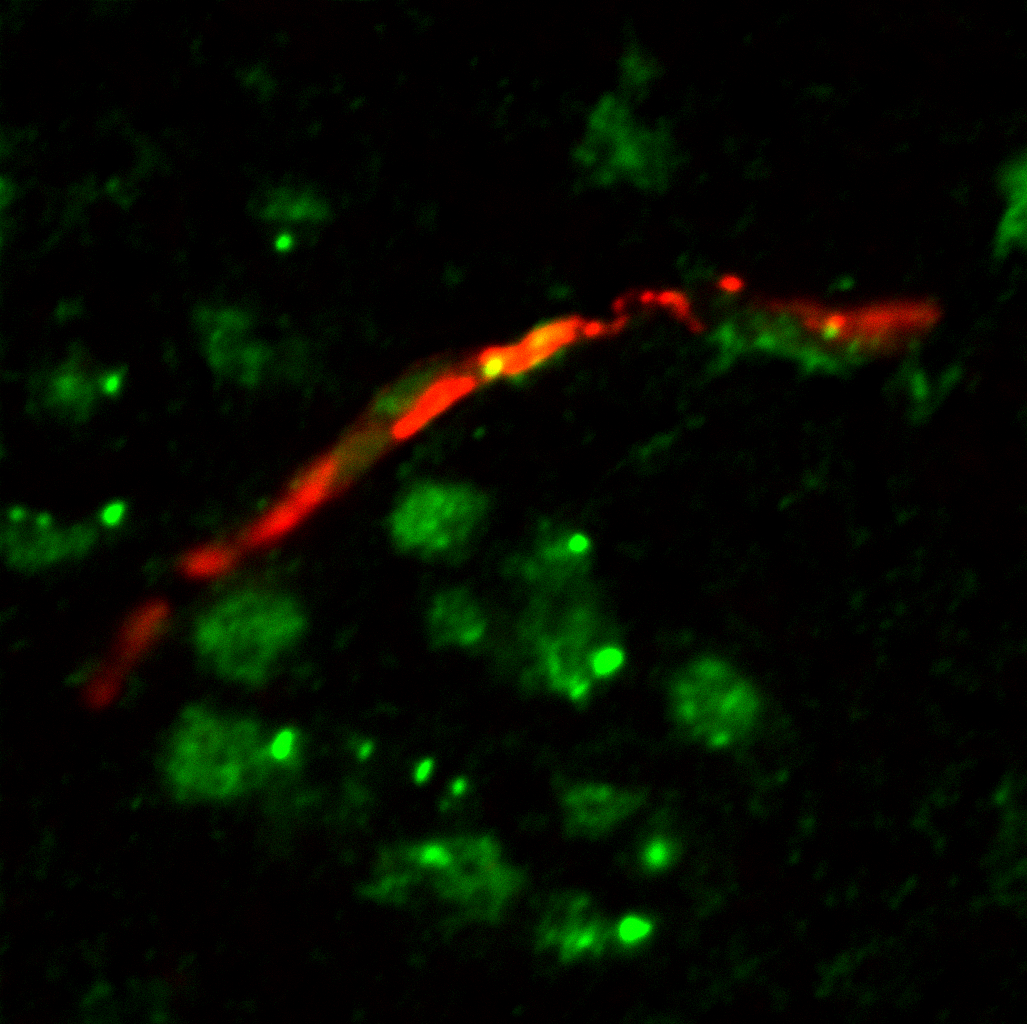

Supplement: Supplementary file 5 [file DataSheet9.ZIP › Immunofluorescence (Figure 6G, part4)/16/3/6s_c1+2.tif]

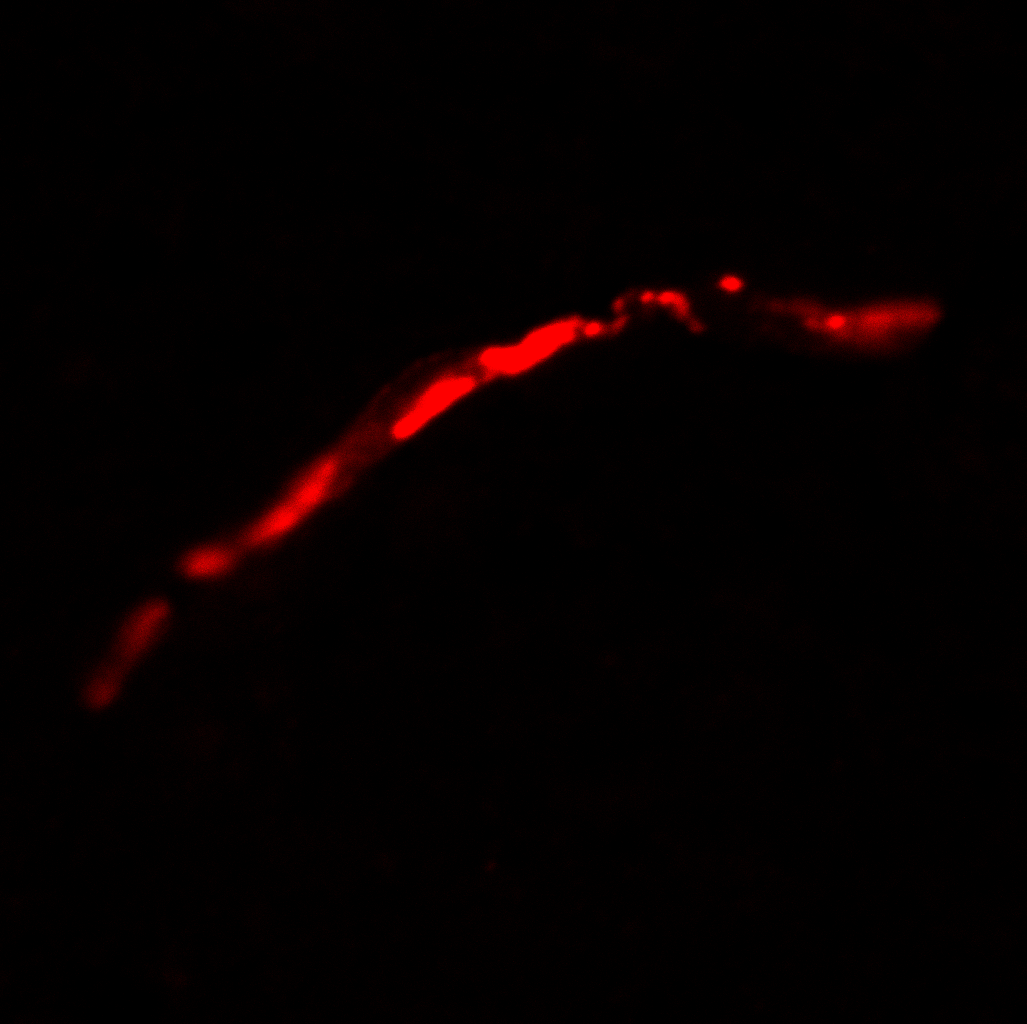

Supplement: Supplementary file 5 [file DataSheet9.ZIP › Immunofluorescence (Figure 6G, part4)/16/3/6s_c1.tif]

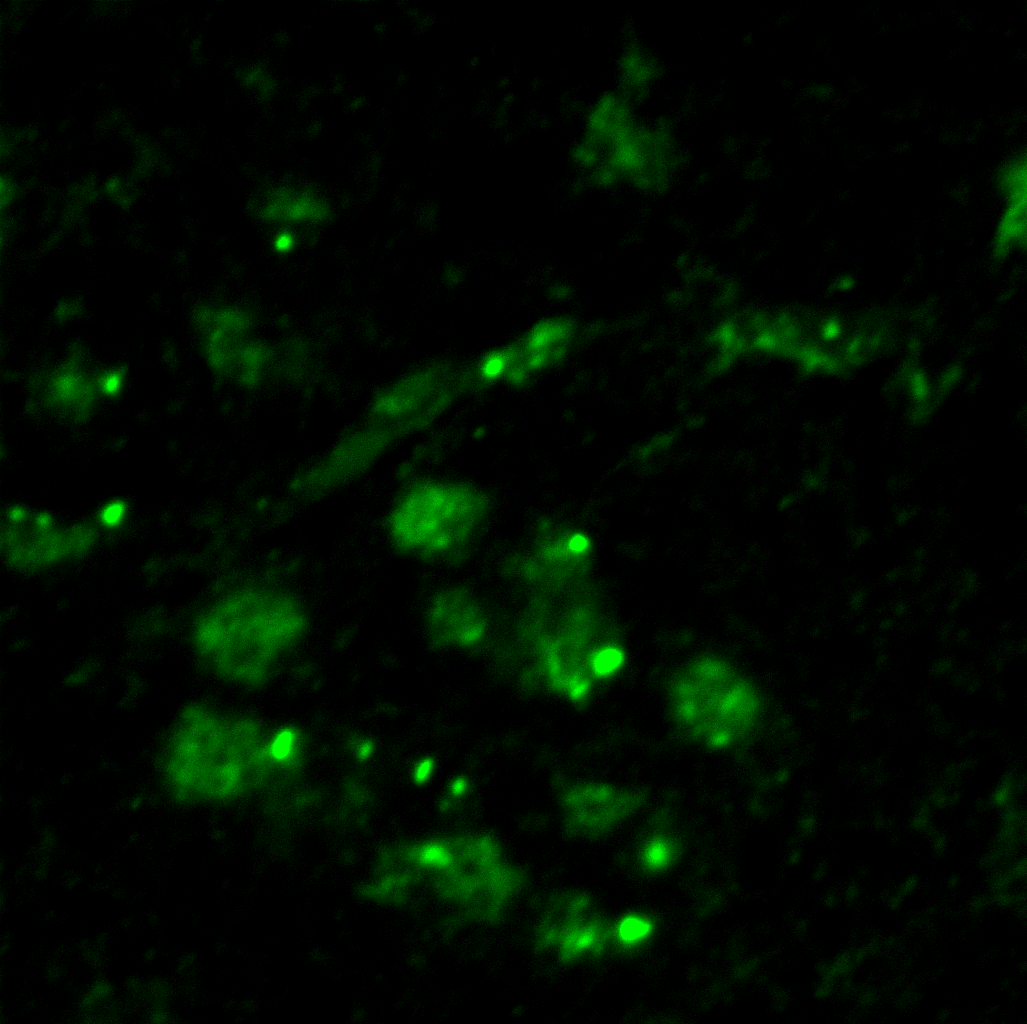

Supplement: Supplementary file 5 [file DataSheet9.ZIP › Immunofluorescence (Figure 6G, part4)/16/3/6s_c2.tif]

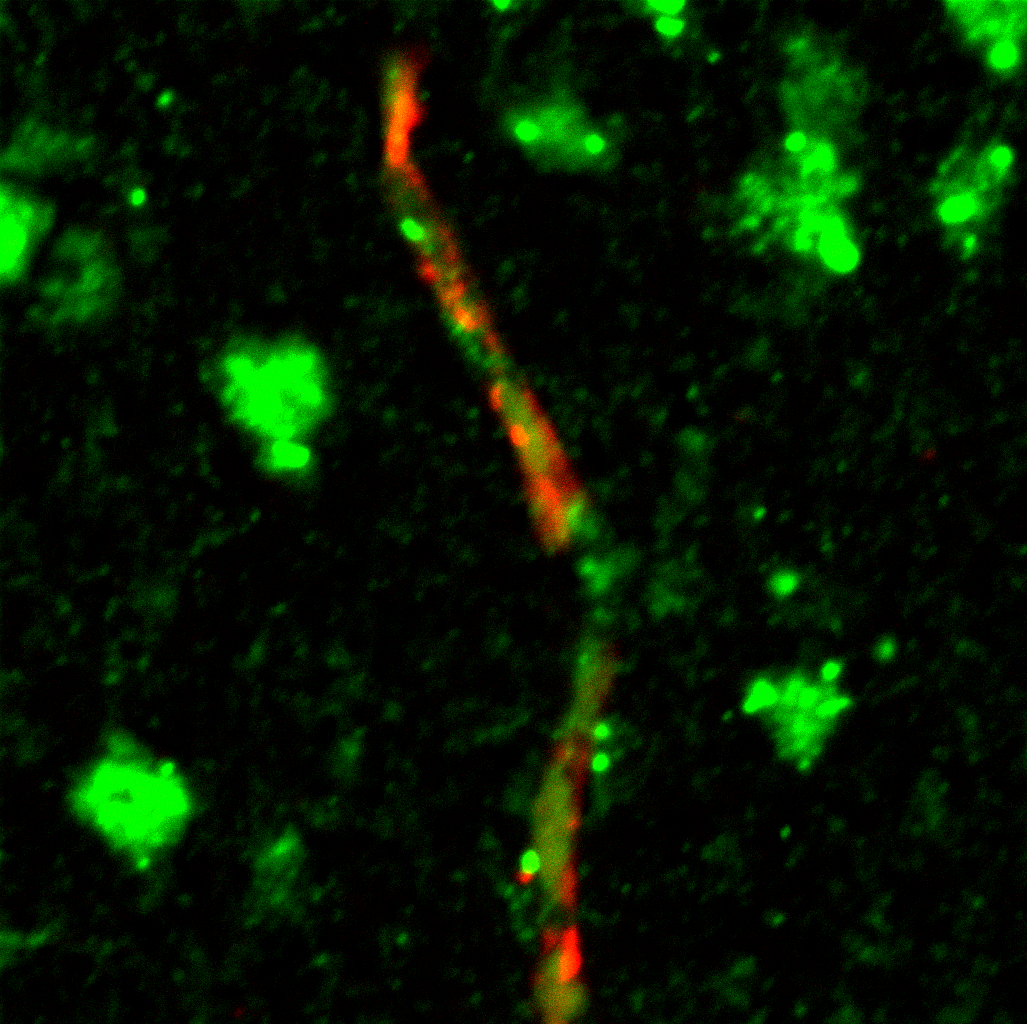

Supplement: Supplementary file 5 [file DataSheet9.ZIP › Immunofluorescence (Figure 6G, part4)/17/1/2s_c1+2.tif]

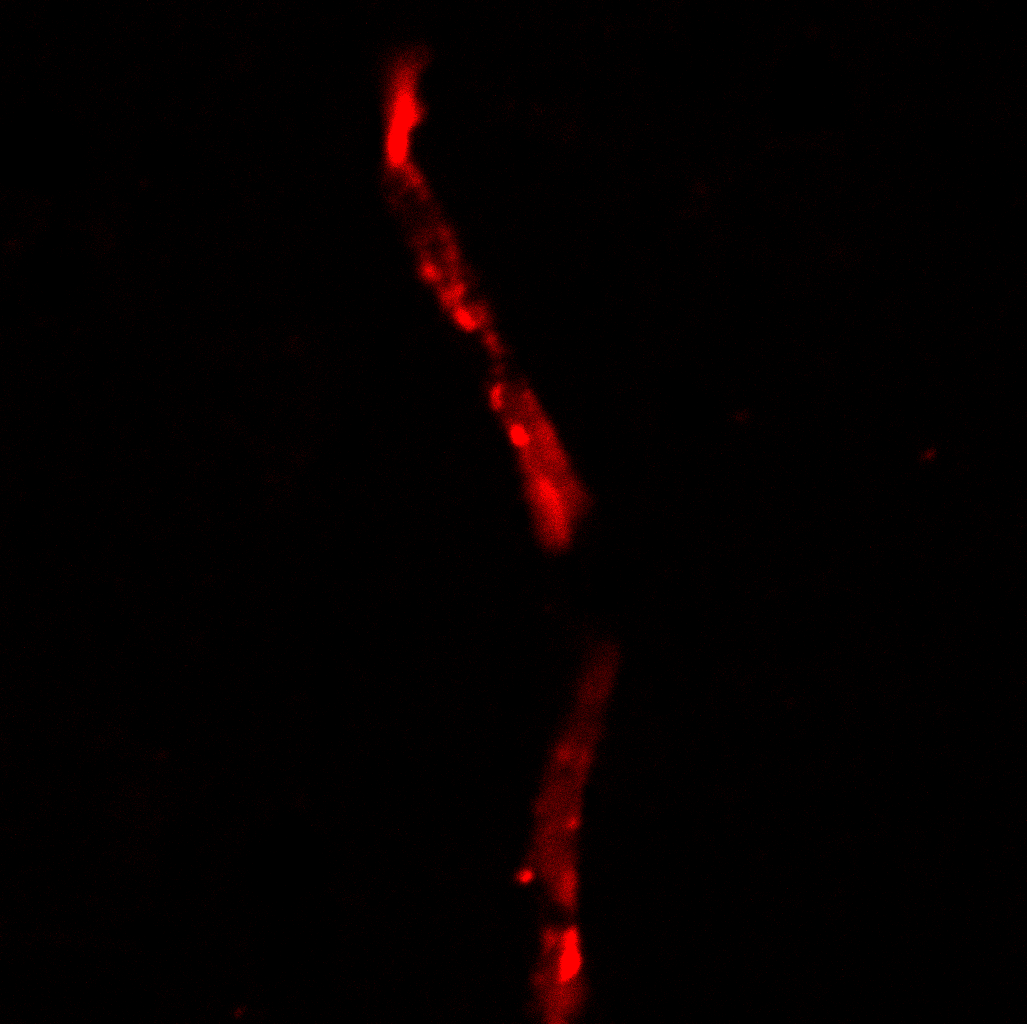

Supplement: Supplementary file 5 [file DataSheet9.ZIP › Immunofluorescence (Figure 6G, part4)/17/1/2s_c1.tif]

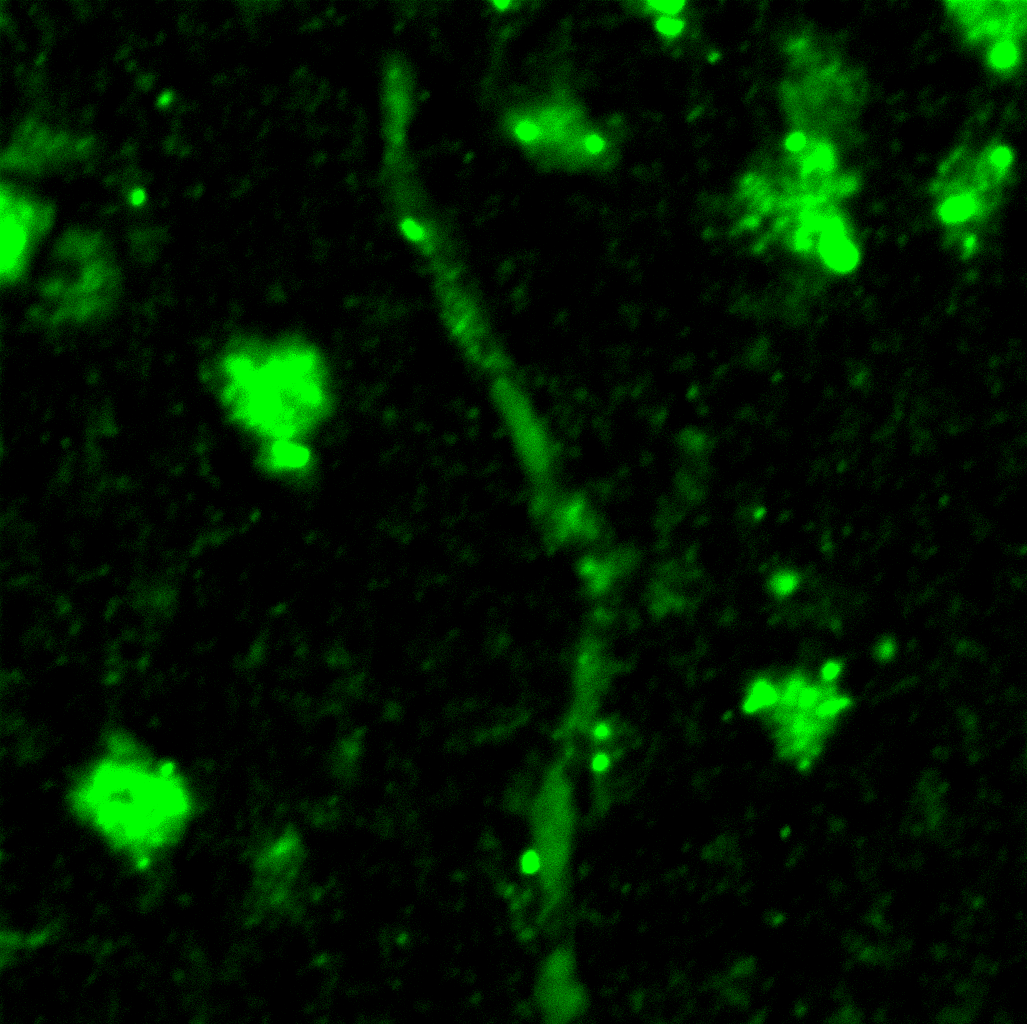

Supplement: Supplementary file 5 [file DataSheet9.ZIP › Immunofluorescence (Figure 6G, part4)/17/1/2s_c2.tif]

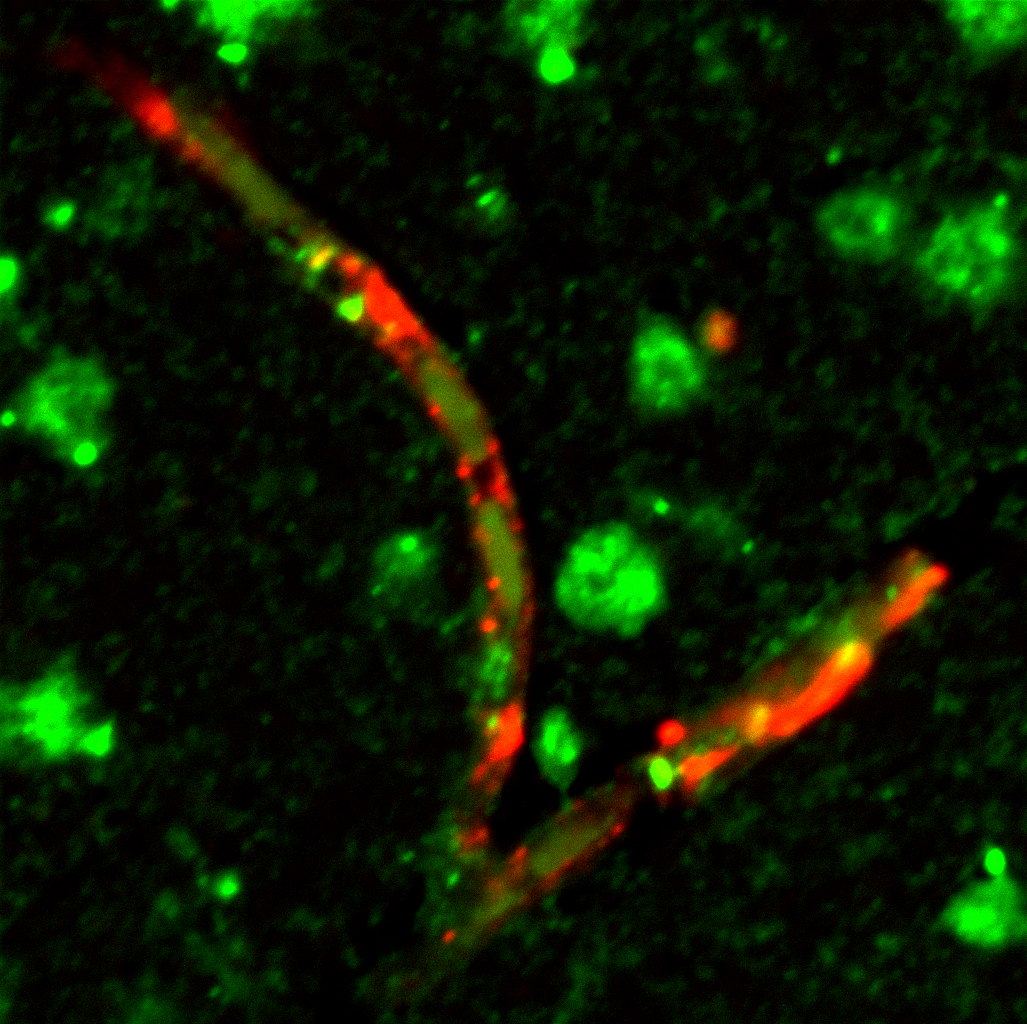

Supplement: Supplementary file 5 [file DataSheet9.ZIP › Immunofluorescence (Figure 6G, part4)/17/2/5s_c1+2.tif]

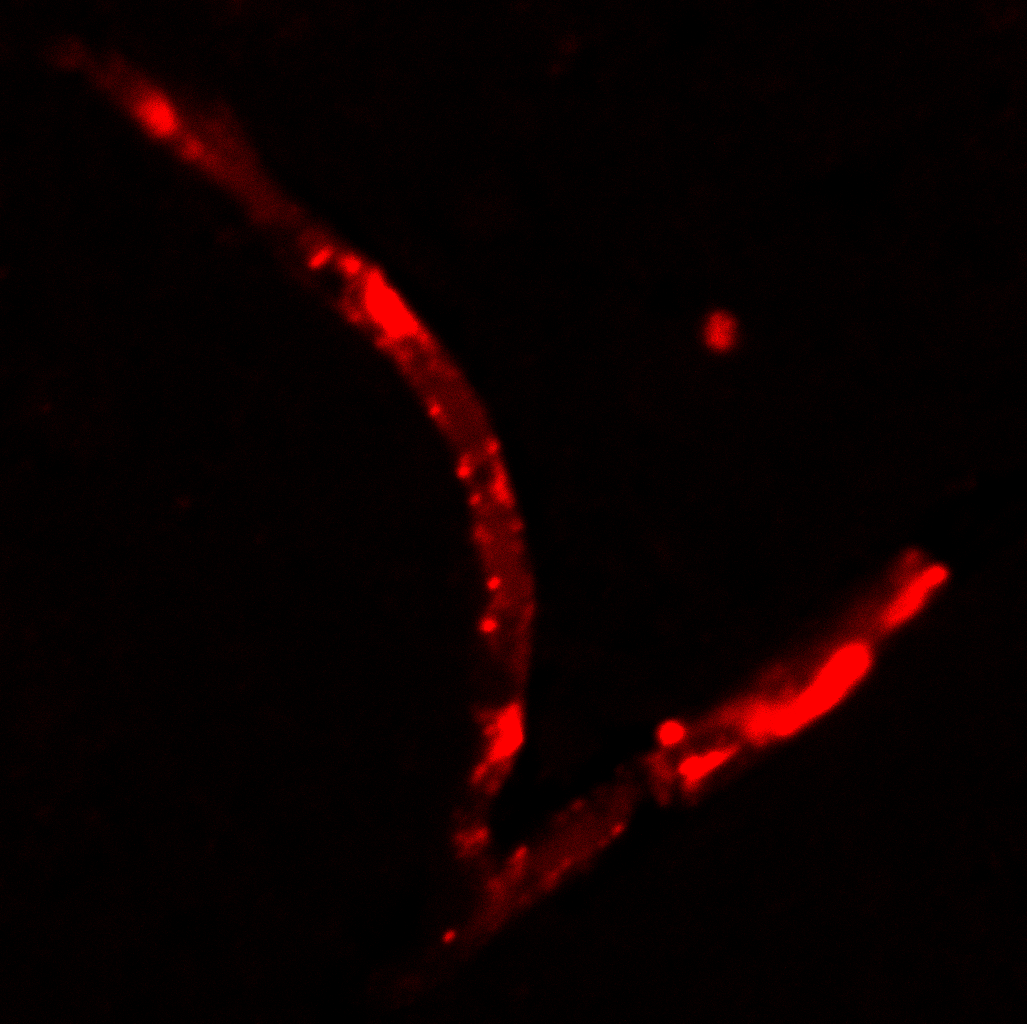

Supplement: Supplementary file 5 [file DataSheet9.ZIP › Immunofluorescence (Figure 6G, part4)/17/2/5s_c1.tif]

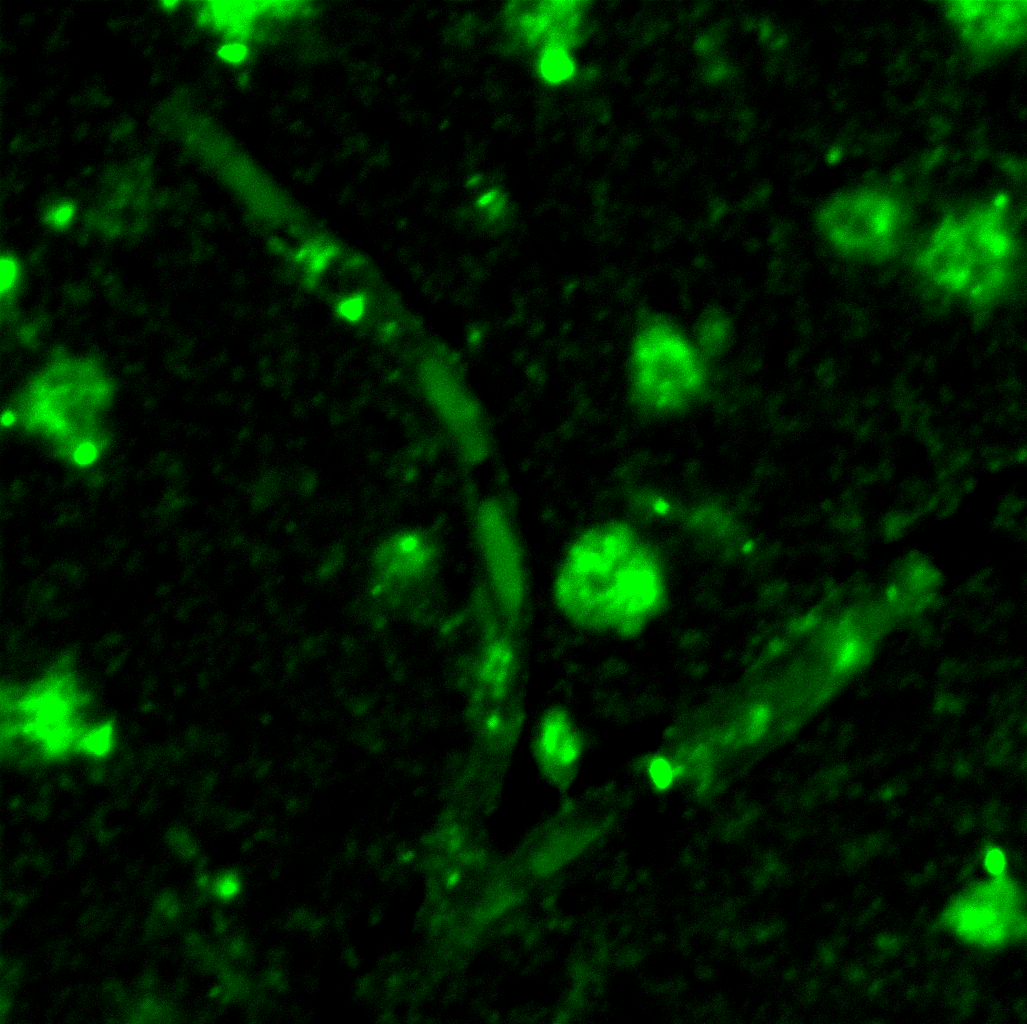

Supplement: Supplementary file 5 [file DataSheet9.ZIP › Immunofluorescence (Figure 6G, part4)/17/2/5s_c2.tif]

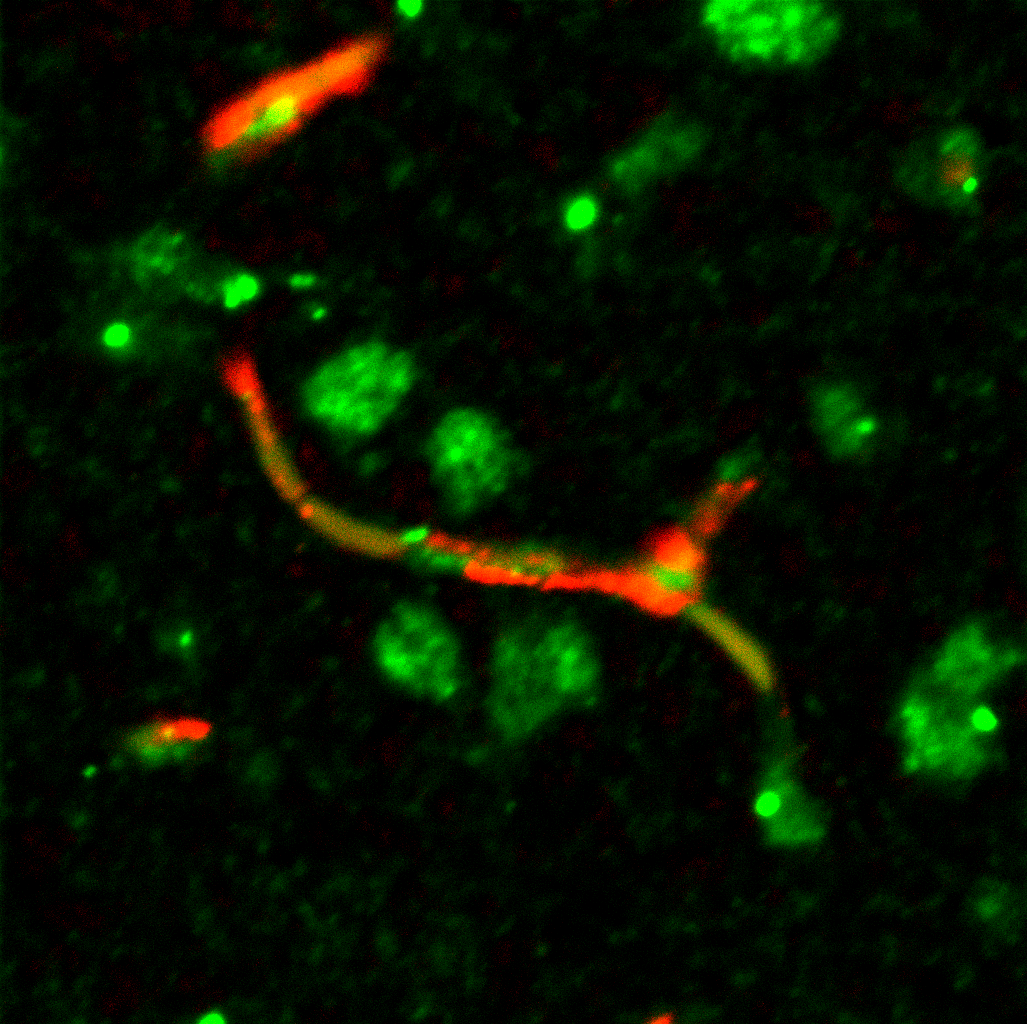

Supplement: Supplementary file 5 [file DataSheet9.ZIP › Immunofluorescence (Figure 6G, part4)/17/3/7s_c1+2.tif]

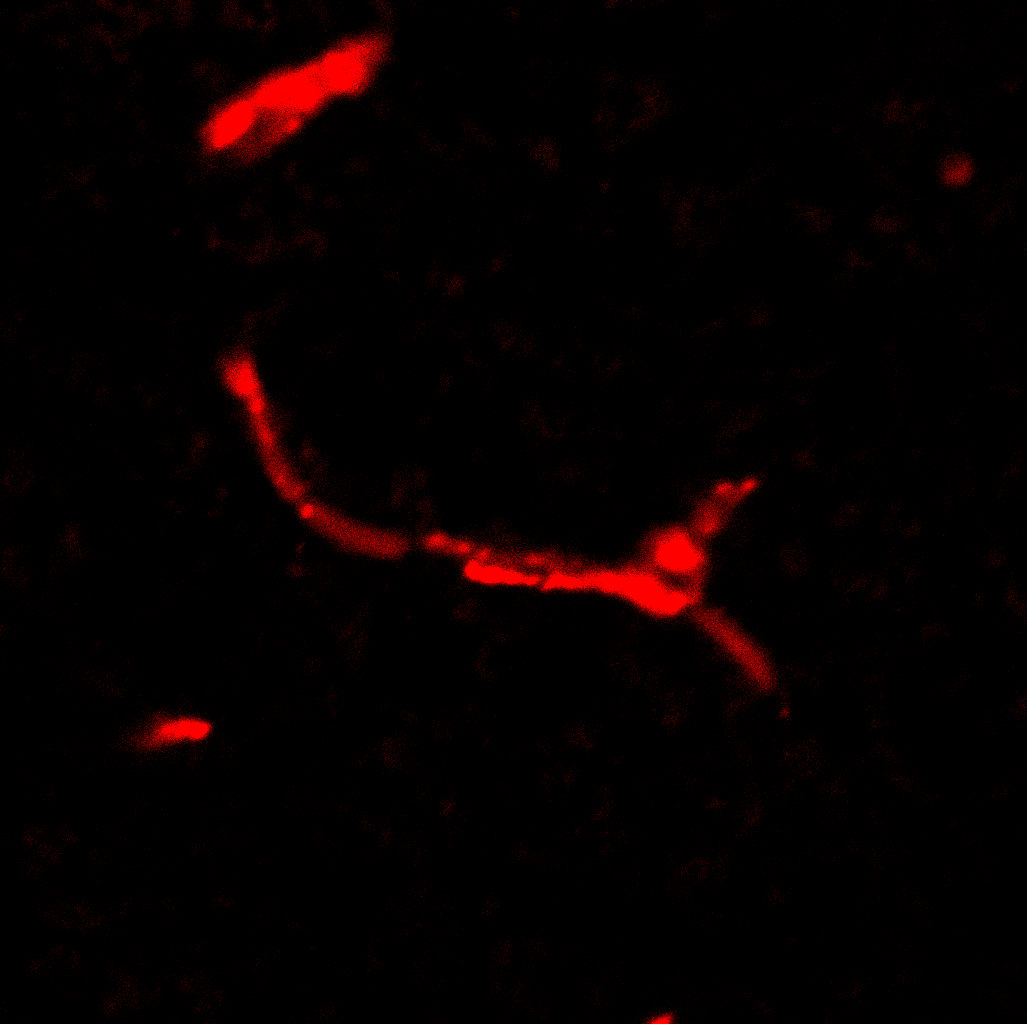

Supplement: Supplementary file 5 [file DataSheet9.ZIP › Immunofluorescence (Figure 6G, part4)/17/3/7s_c1.tif]

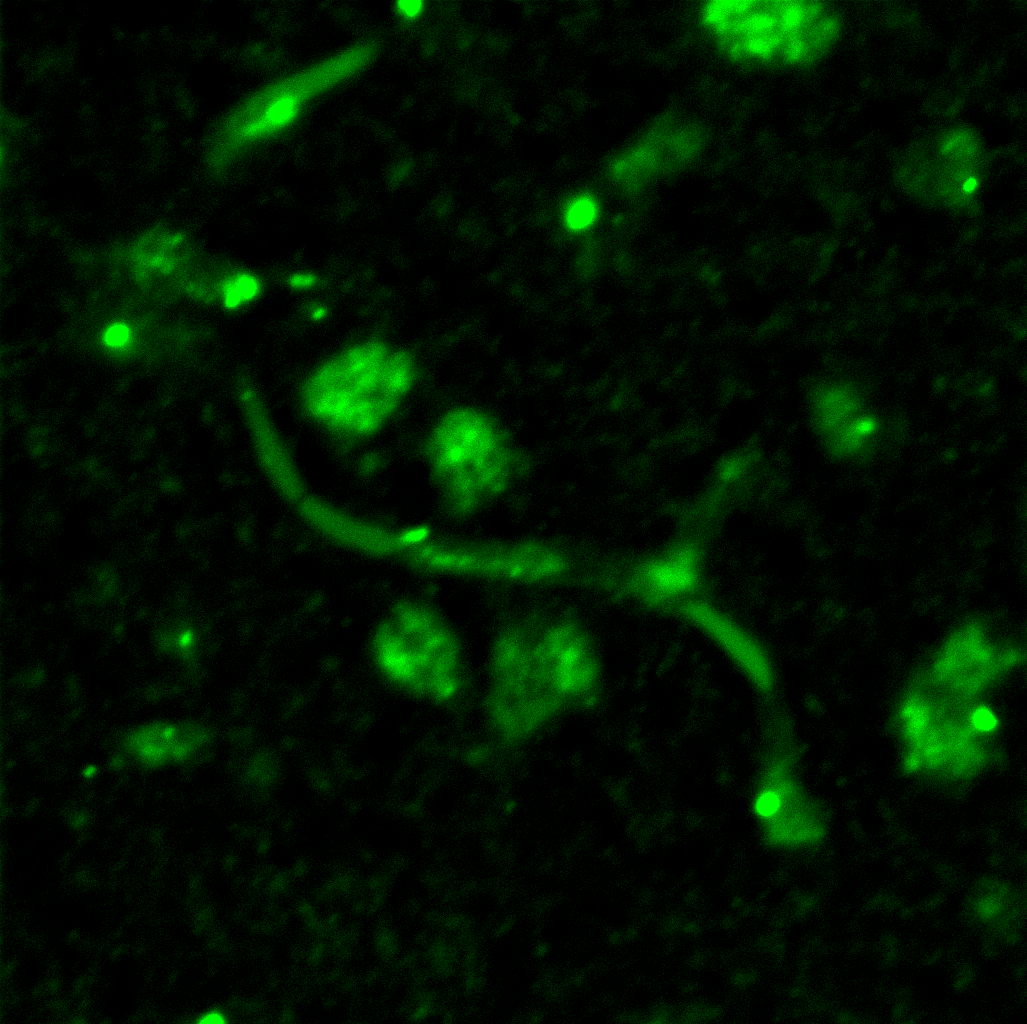

Supplement: Supplementary file 5 [file DataSheet9.ZIP › Immunofluorescence (Figure 6G, part4)/17/3/7s_c2.tif]

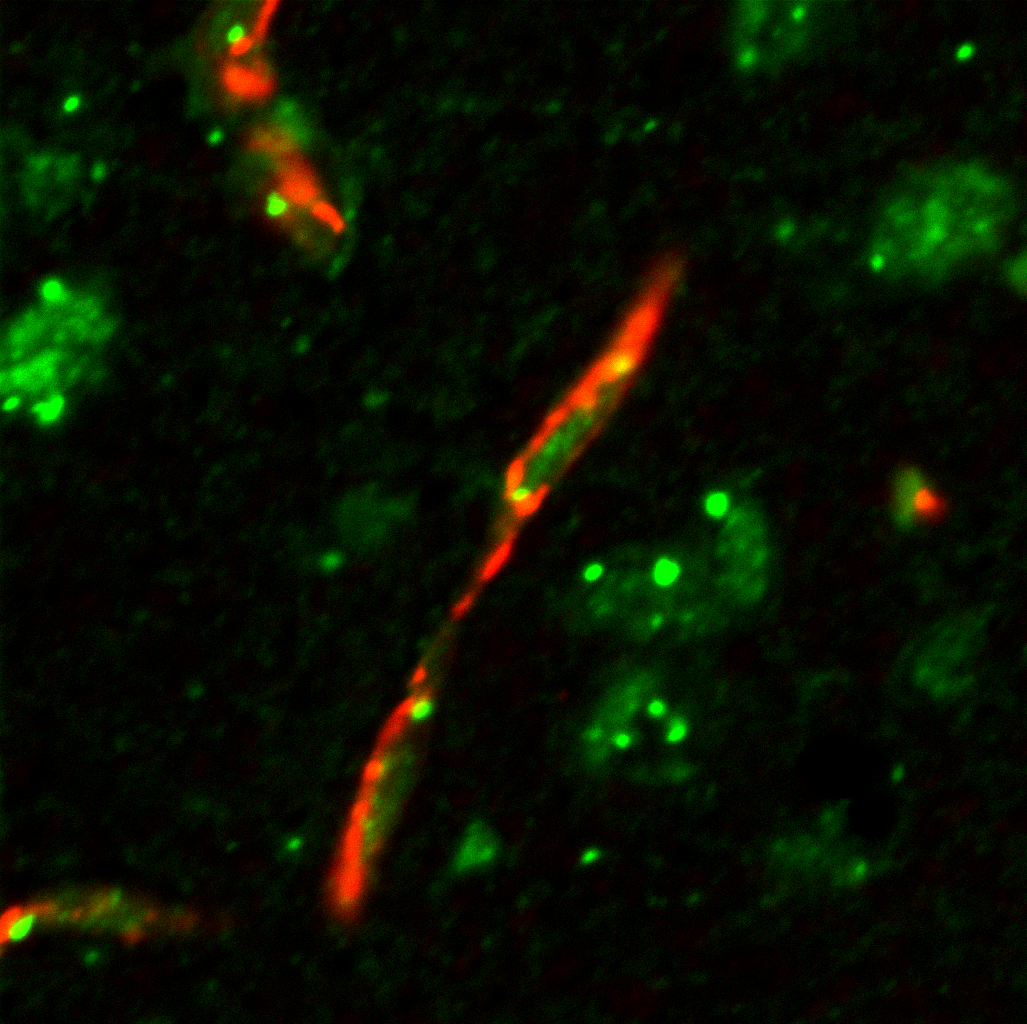

Supplement: Supplementary file 5 [file DataSheet9.ZIP › Immunofluorescence (Figure 6G, part4)/18/1/1s_c1+2.tif]

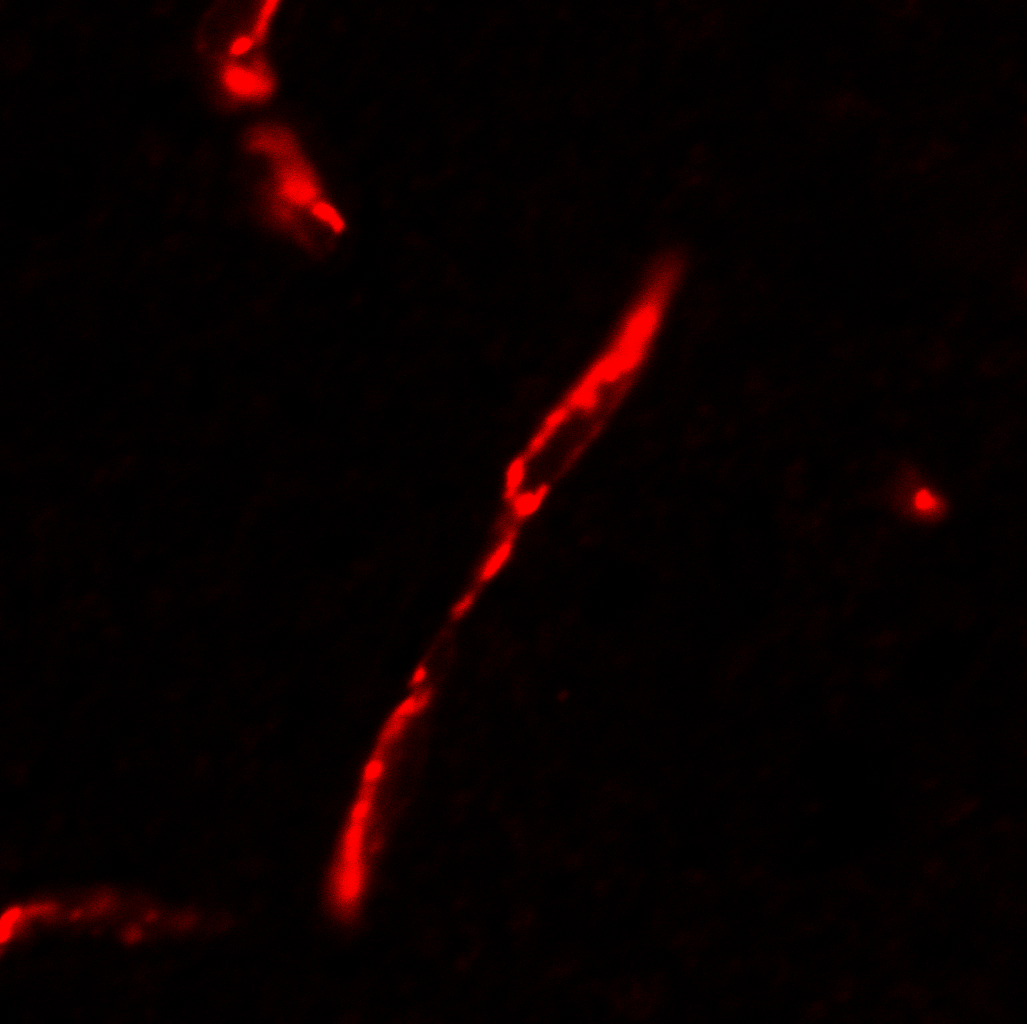

Supplement: Supplementary file 5 [file DataSheet9.ZIP › Immunofluorescence (Figure 6G, part4)/18/1/1s_c1.tif]

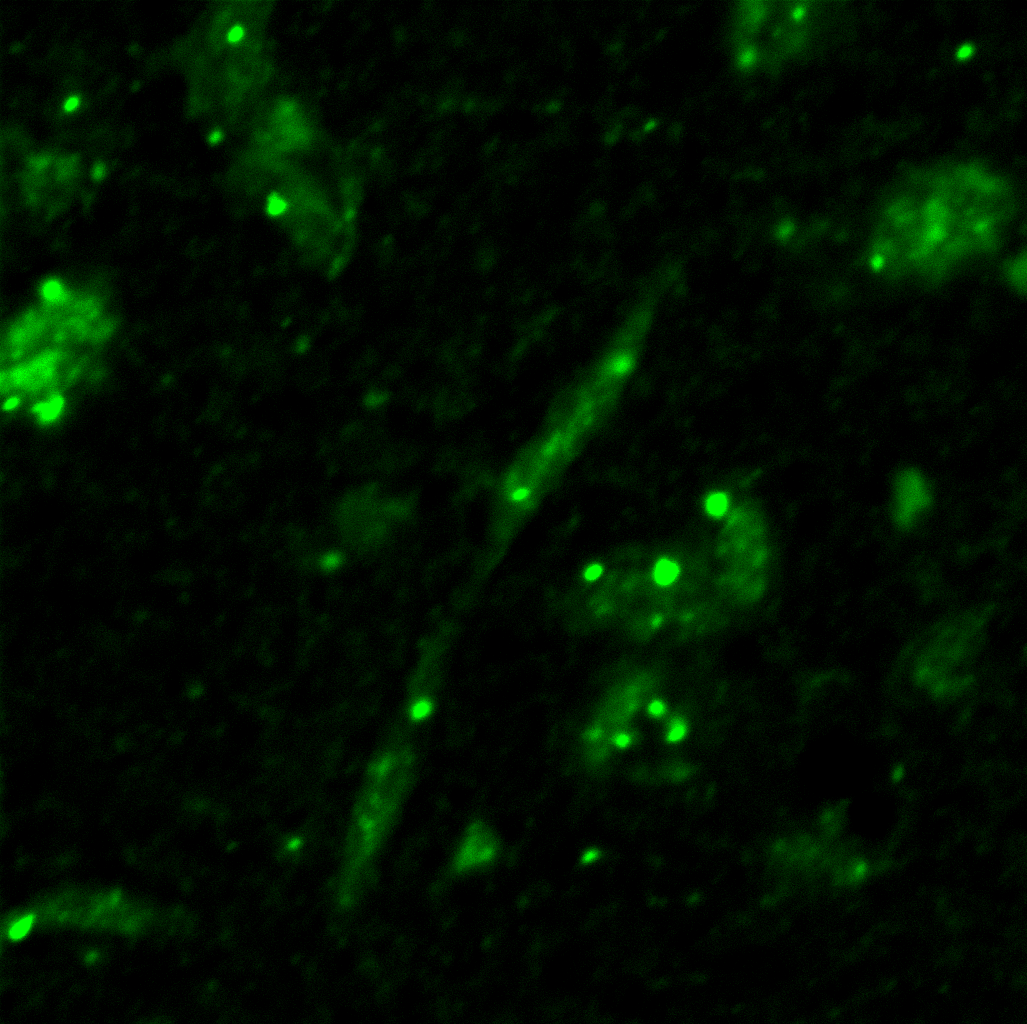

Supplement: Supplementary file 5 [file DataSheet9.ZIP › Immunofluorescence (Figure 6G, part4)/18/1/1s_c2.tif]

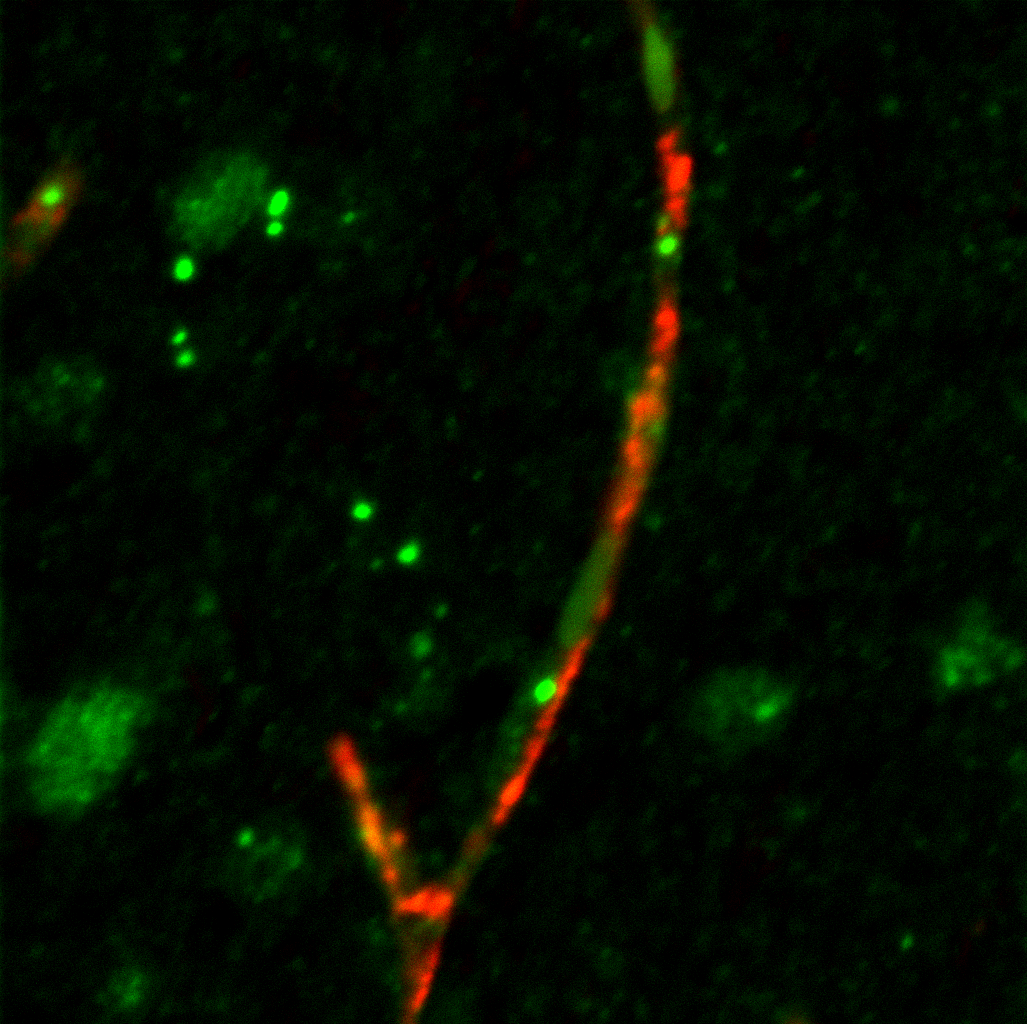

Supplement: Supplementary file 5 [file DataSheet9.ZIP › Immunofluorescence (Figure 6G, part4)/18/2/2s_c1+2.tif]

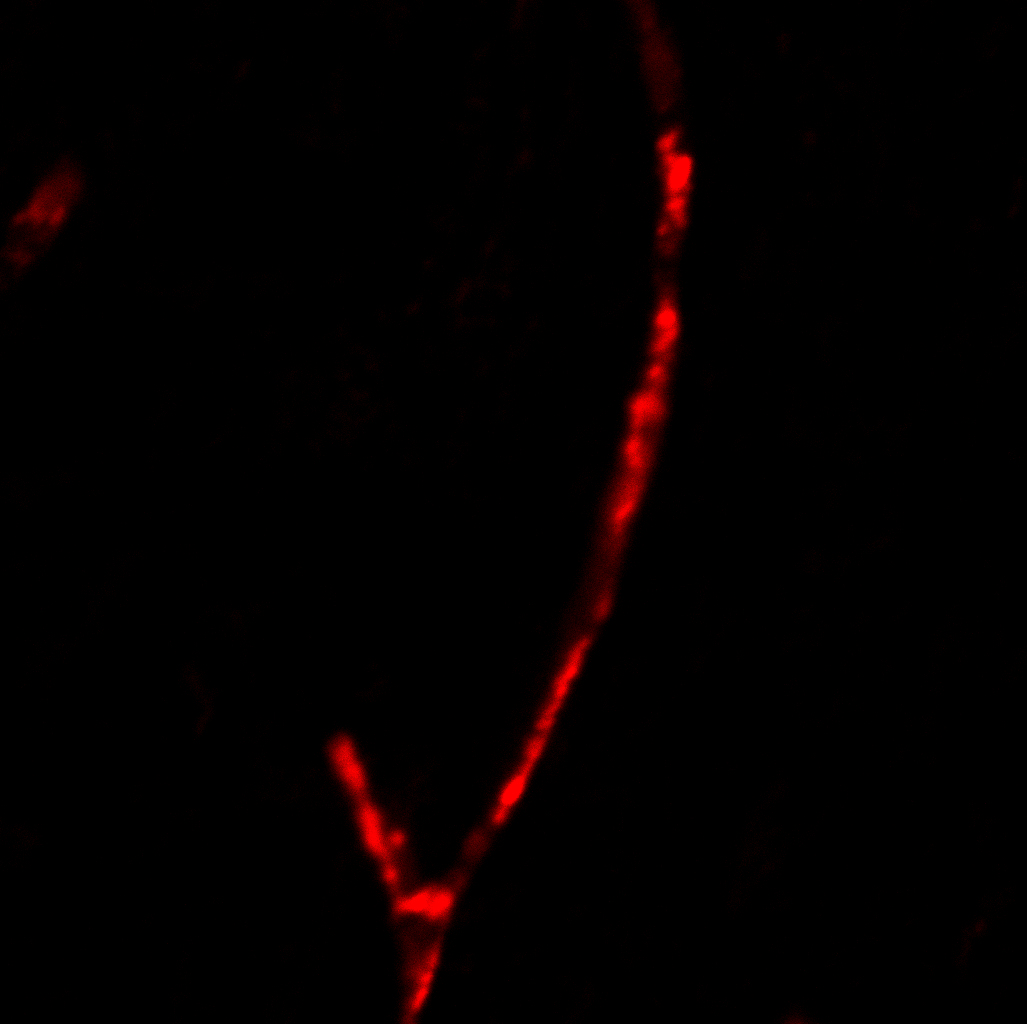

Supplement: Supplementary file 5 [file DataSheet9.ZIP › Immunofluorescence (Figure 6G, part4)/18/2/2s_c1.tif]

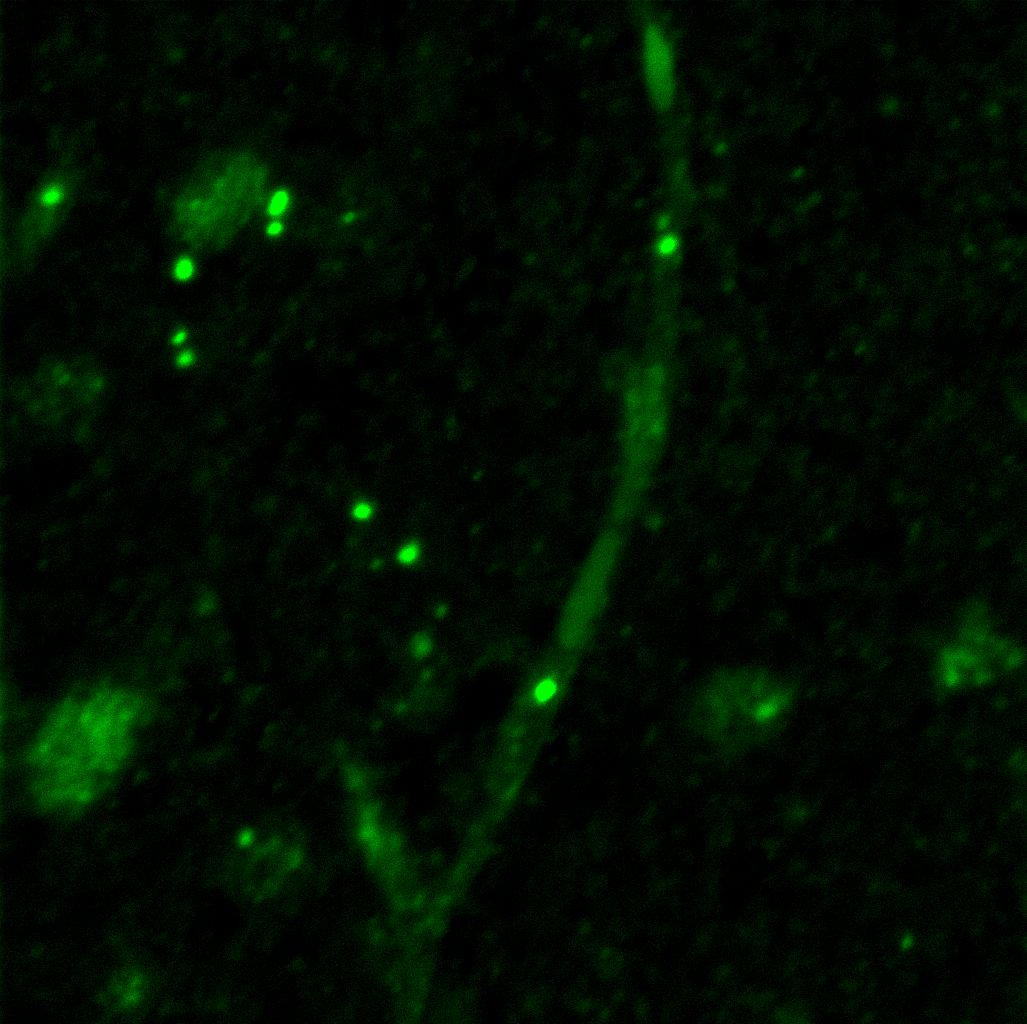

Supplement: Supplementary file 5 [file DataSheet9.ZIP › Immunofluorescence (Figure 6G, part4)/18/2/2s_c2.tif]

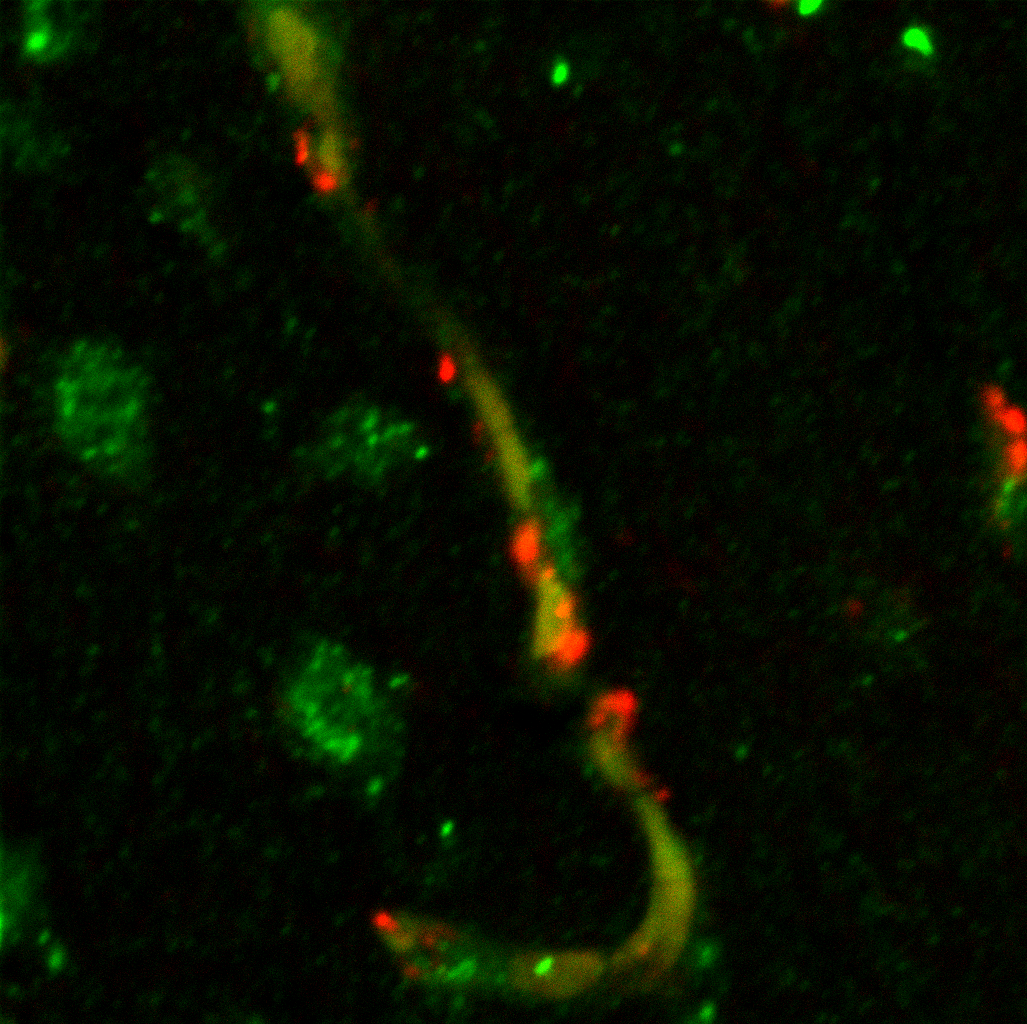

Supplement: Supplementary file 5 [file DataSheet9.ZIP › Immunofluorescence (Figure 6G, part4)/18/3/3s_c1+2.tif]

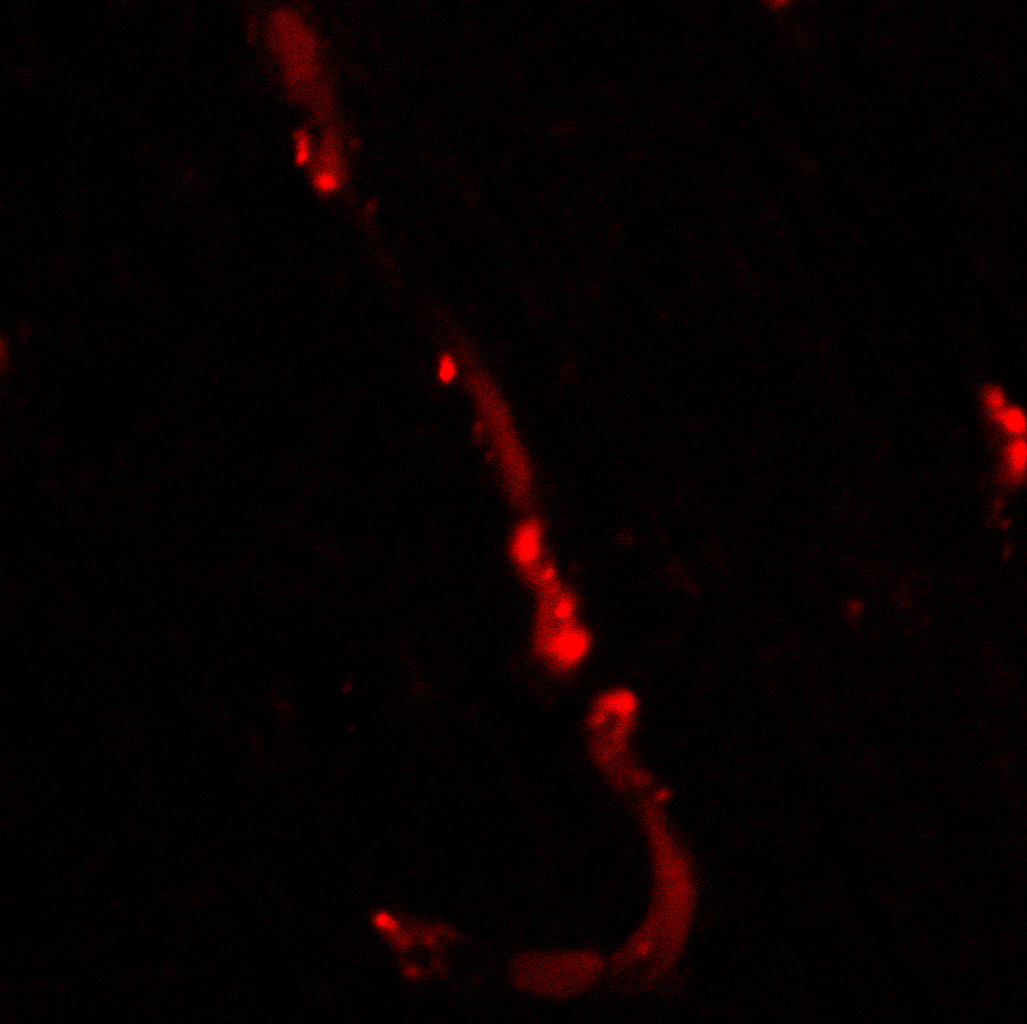

Supplement: Supplementary file 5 [file DataSheet9.ZIP › Immunofluorescence (Figure 6G, part4)/18/3/3s_c1.tif]

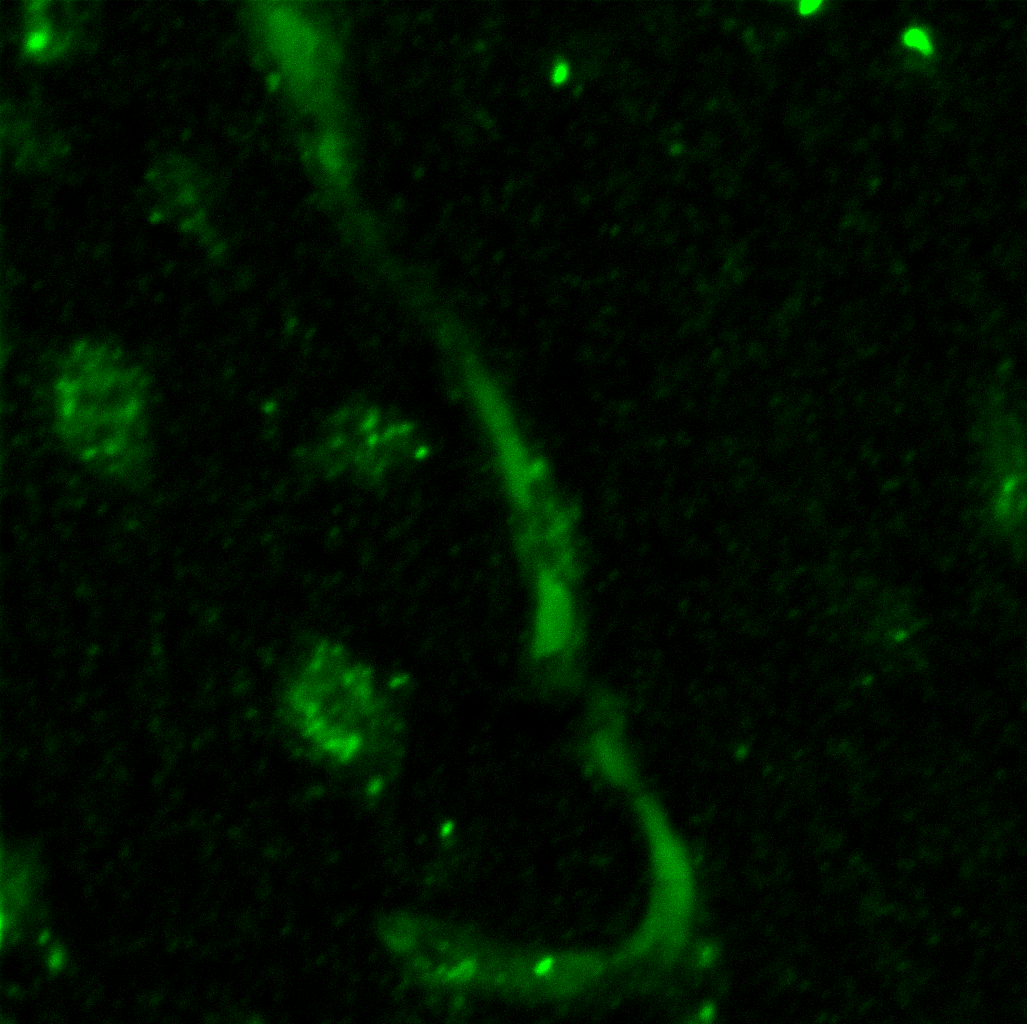

Supplement: Supplementary file 5 [file DataSheet9.ZIP › Immunofluorescence (Figure 6G, part4)/18/3/3s_c2.tif]

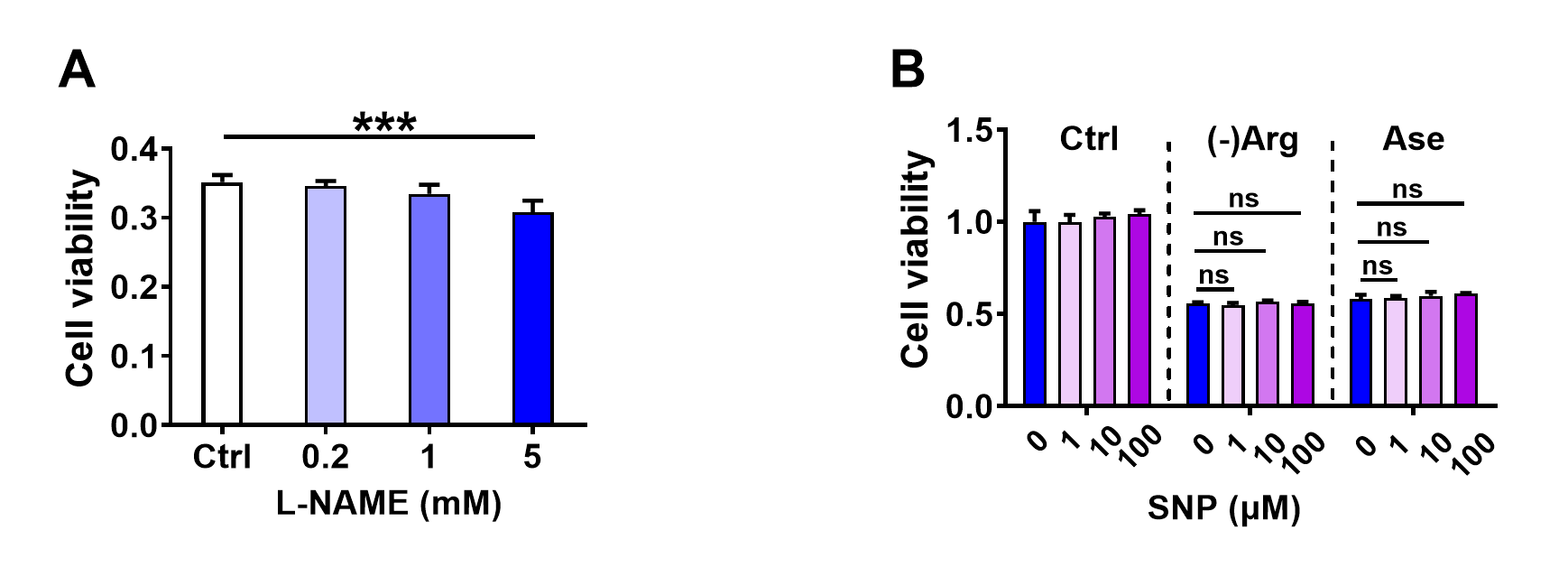

Supplement: Supplementary file 8 [file Image2.TIF]

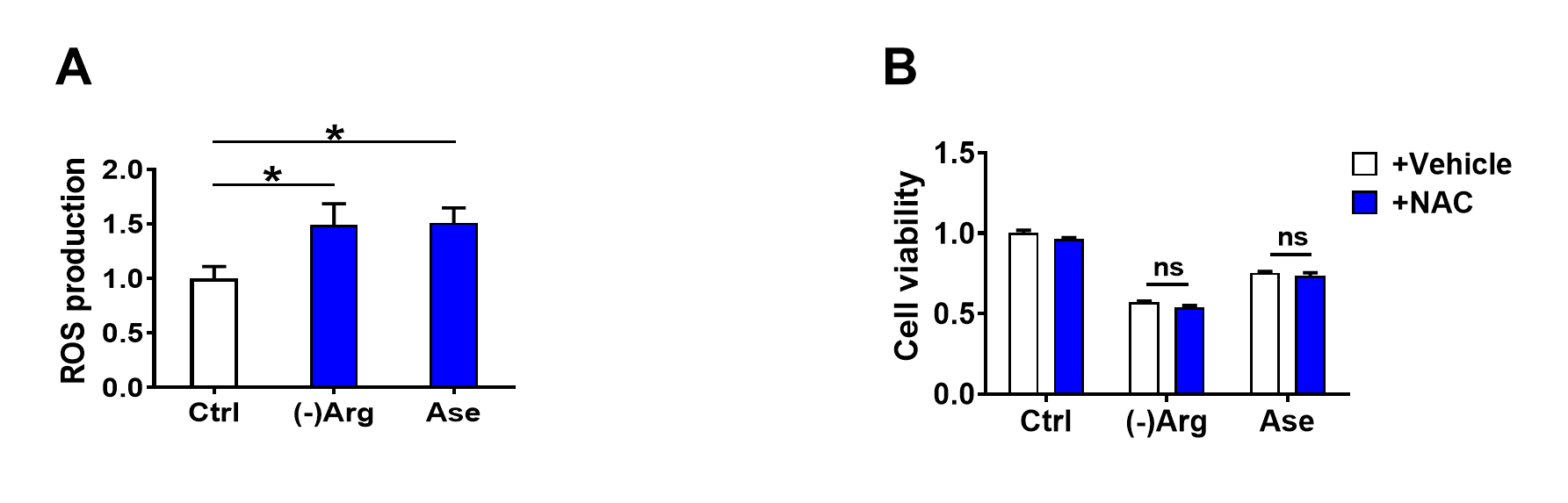

Supplement: Supplementary file 10 [file Image1.TIF]

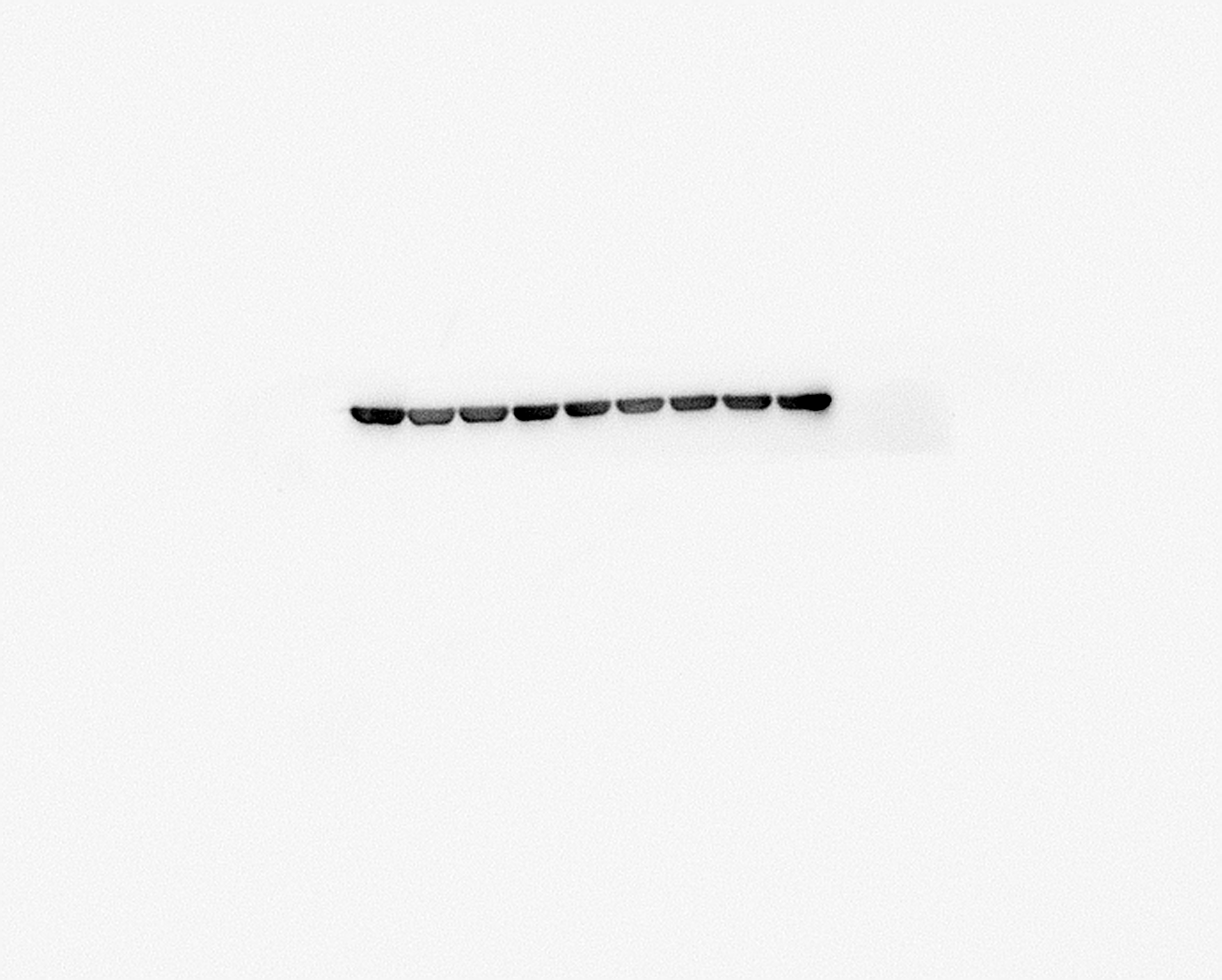

Supplement: Supplementary file 11 [file DataSheet10.ZIP › Western blot/Figure 4N/1 (presented in manuscript)/1-actin(ZO1).tif]

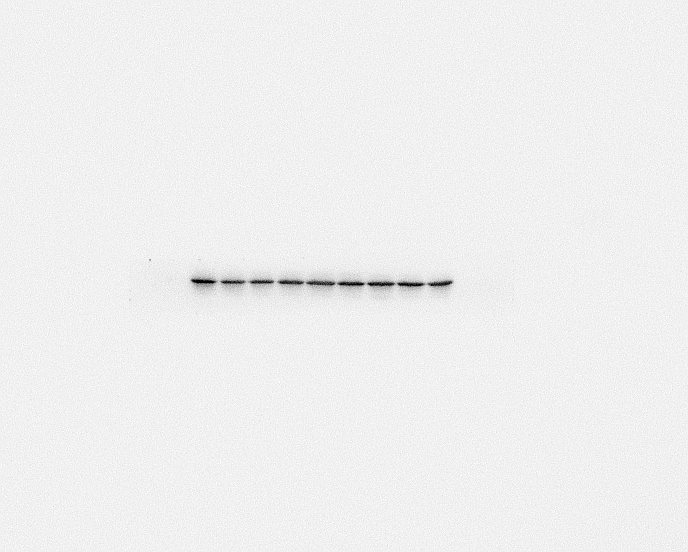

Supplement: Supplementary file 11 [file DataSheet10.ZIP › Western blot/Figure 4N/1 (presented in manuscript)/1-actin.tif]

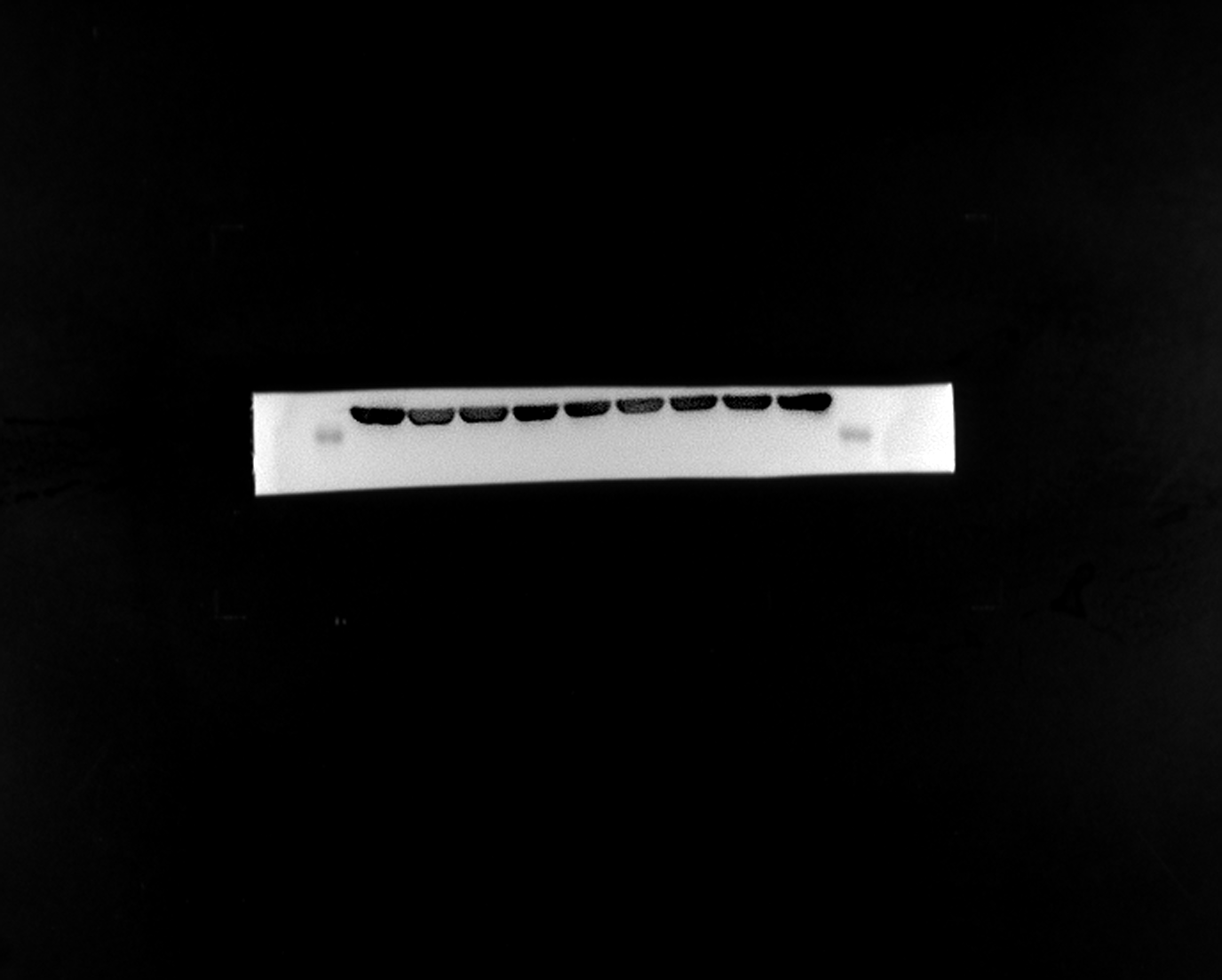

Supplement: Supplementary file 11 [file DataSheet10.ZIP › Western blot/Figure 4N/1 (presented in manuscript)/1-actins(ZO1).tif]

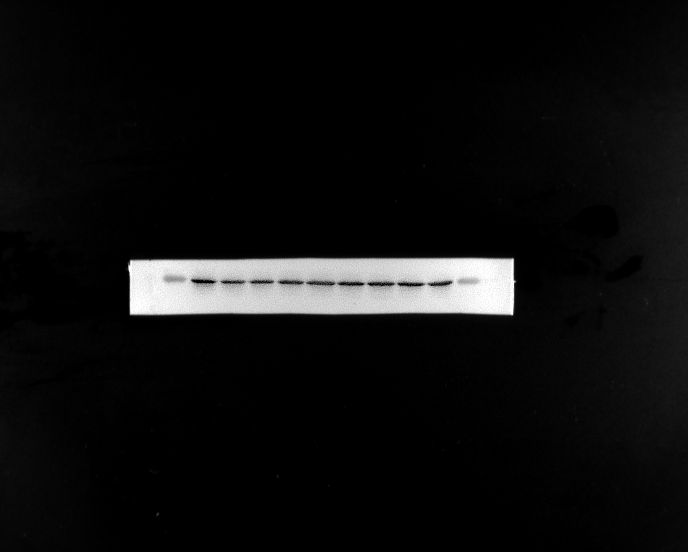

Supplement: Supplementary file 11 [file DataSheet10.ZIP › Western blot/Figure 4N/1 (presented in manuscript)/1-actins.tif]

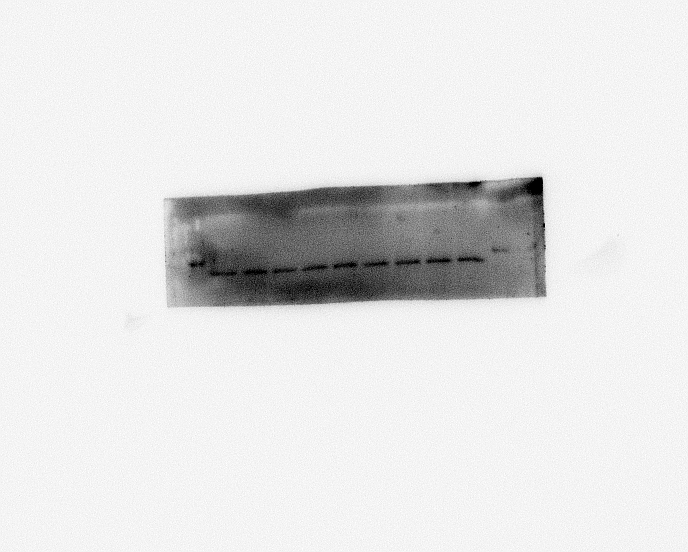

Supplement: Supplementary file 11 [file DataSheet10.ZIP › Western blot/Figure 4N/1 (presented in manuscript)/1-claudin.tif]

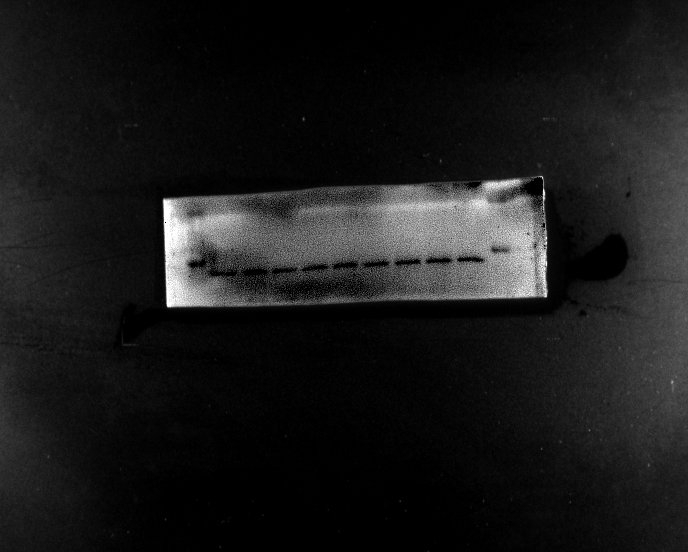

Supplement: Supplementary file 11 [file DataSheet10.ZIP › Western blot/Figure 4N/1 (presented in manuscript)/1-claudins.tif]

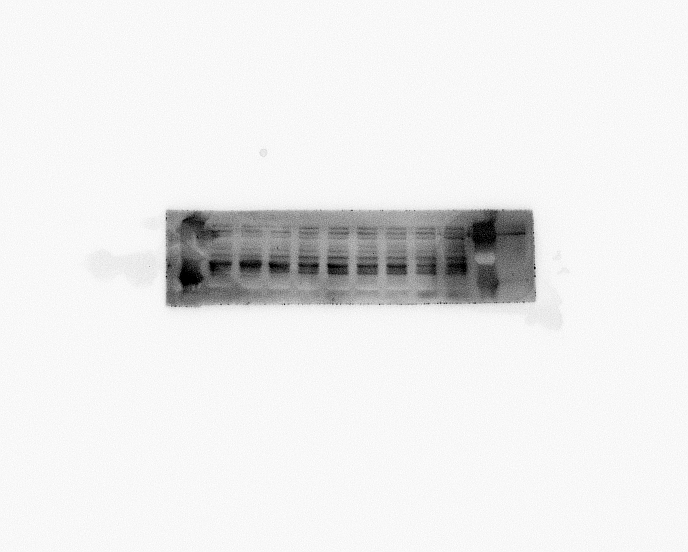

Supplement: Supplementary file 11 [file DataSheet10.ZIP › Western blot/Figure 4N/1 (presented in manuscript)/1-OCCLUDIN.tif]

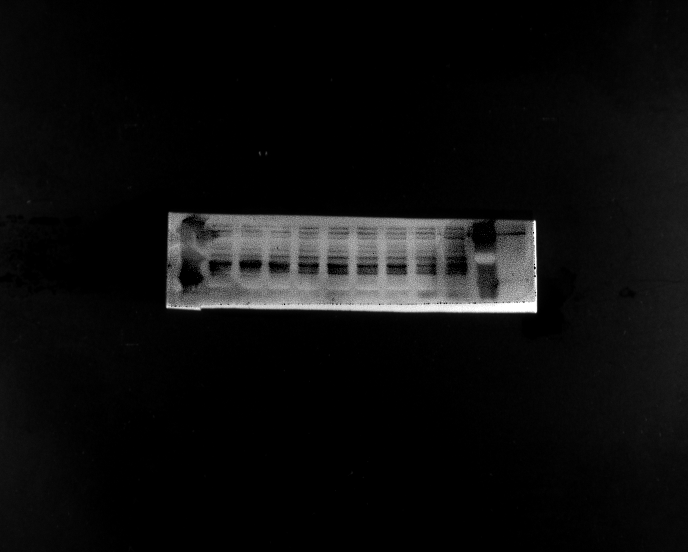

Supplement: Supplementary file 11 [file DataSheet10.ZIP › Western blot/Figure 4N/1 (presented in manuscript)/1-OCCLUDINS.tif]

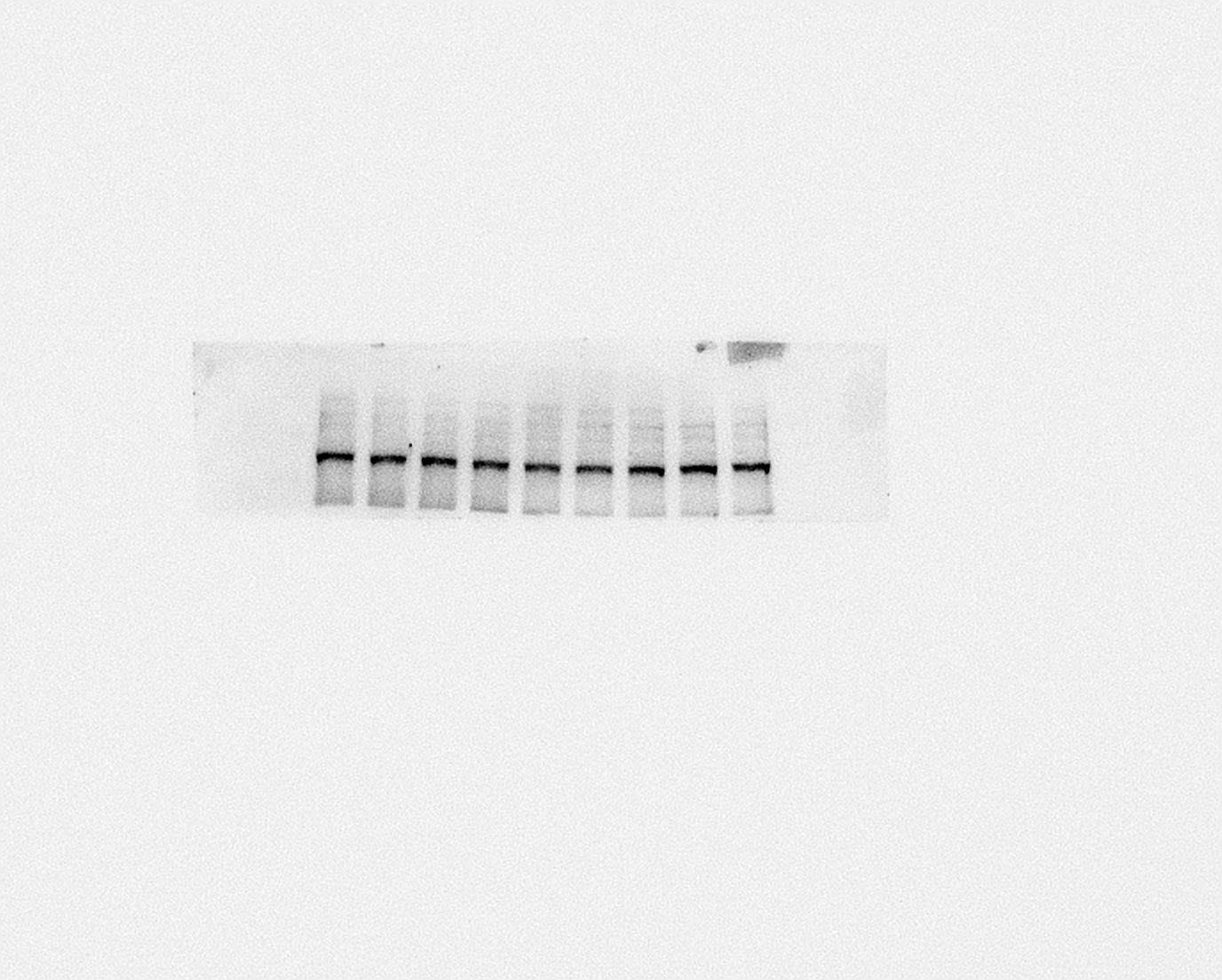

Supplement: Supplementary file 11 [file DataSheet10.ZIP › Western blot/Figure 4N/1 (presented in manuscript)/1-ZO1.tif]

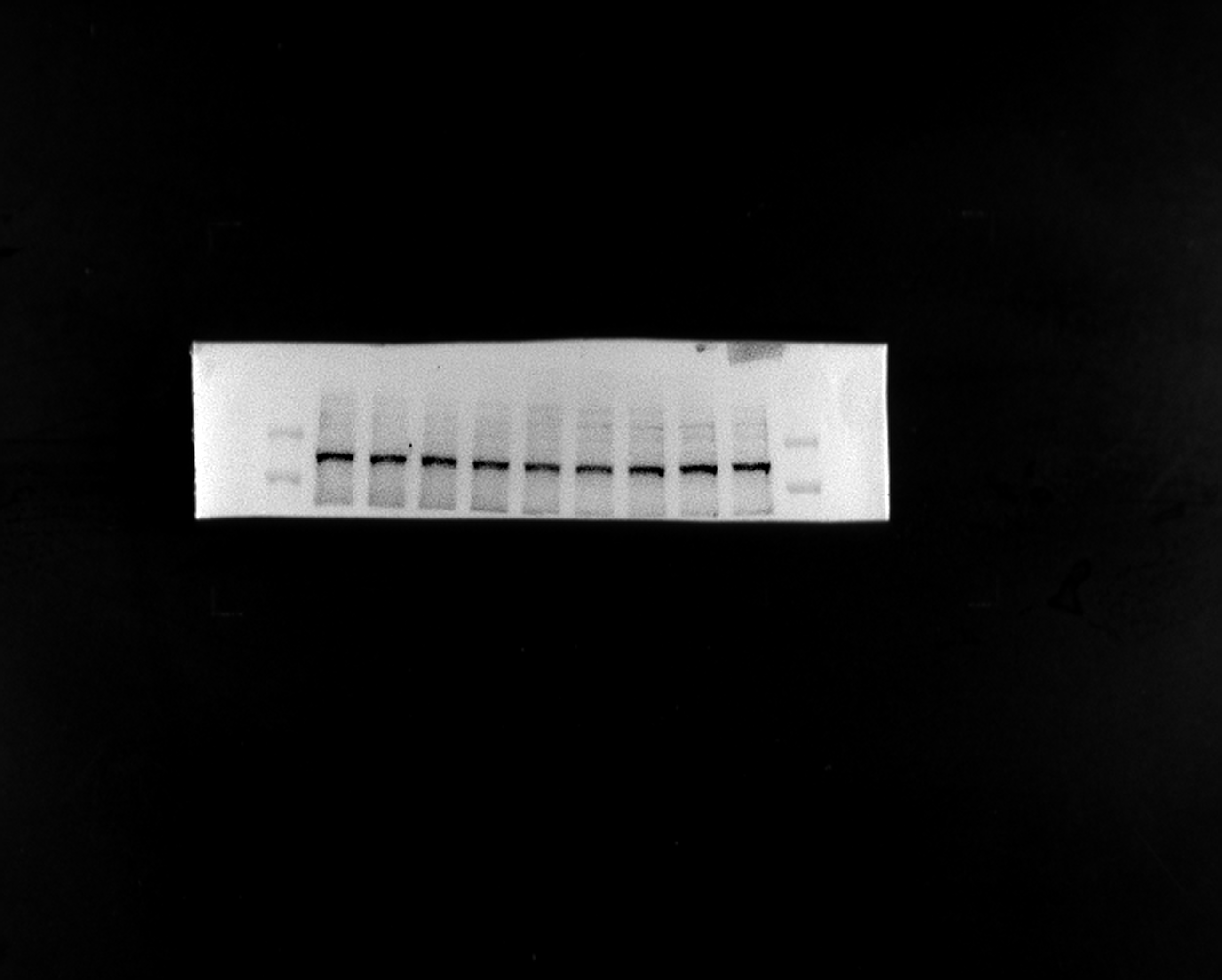

Supplement: Supplementary file 11 [file DataSheet10.ZIP › Western blot/Figure 4N/1 (presented in manuscript)/1-ZO1s.tif]

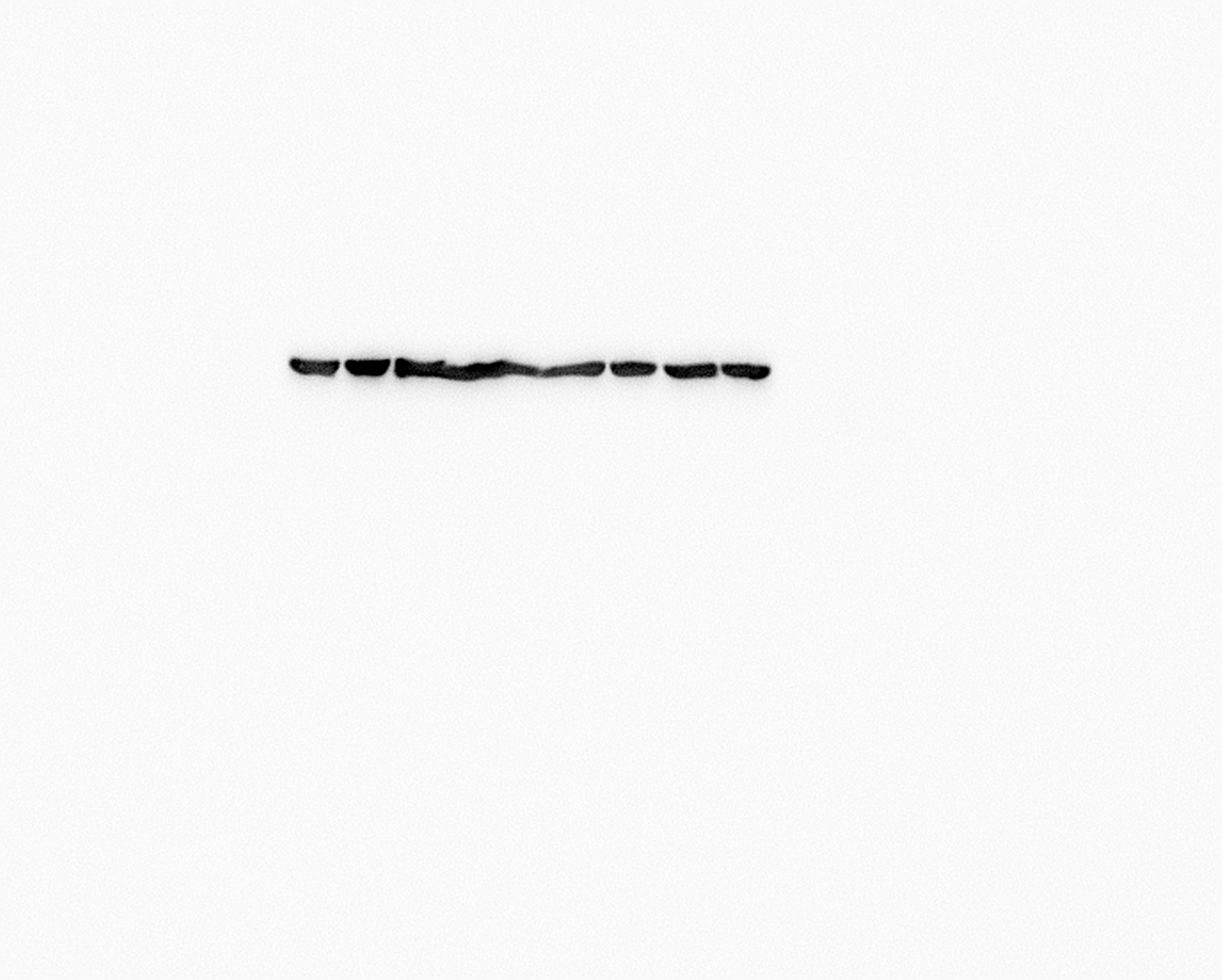

Supplement: Supplementary file 11 [file DataSheet10.ZIP › Western blot/Figure 4N/2/2-actin (ZO1).tif]

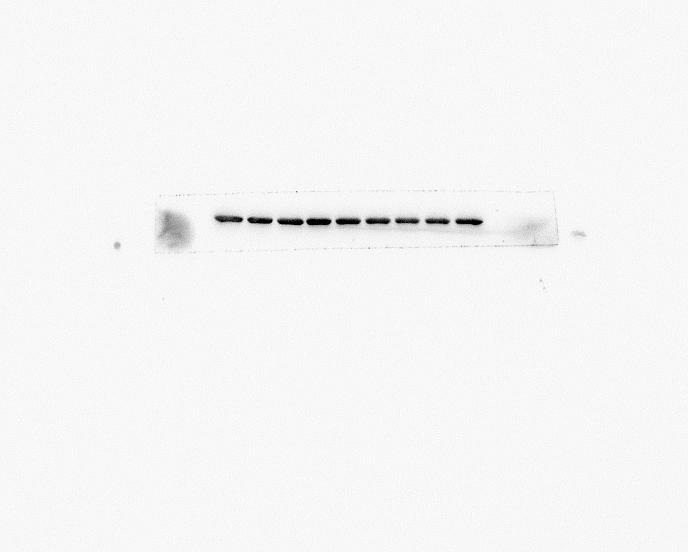

Supplement: Supplementary file 11 [file DataSheet10.ZIP › Western blot/Figure 4N/2/2-actin.tif]

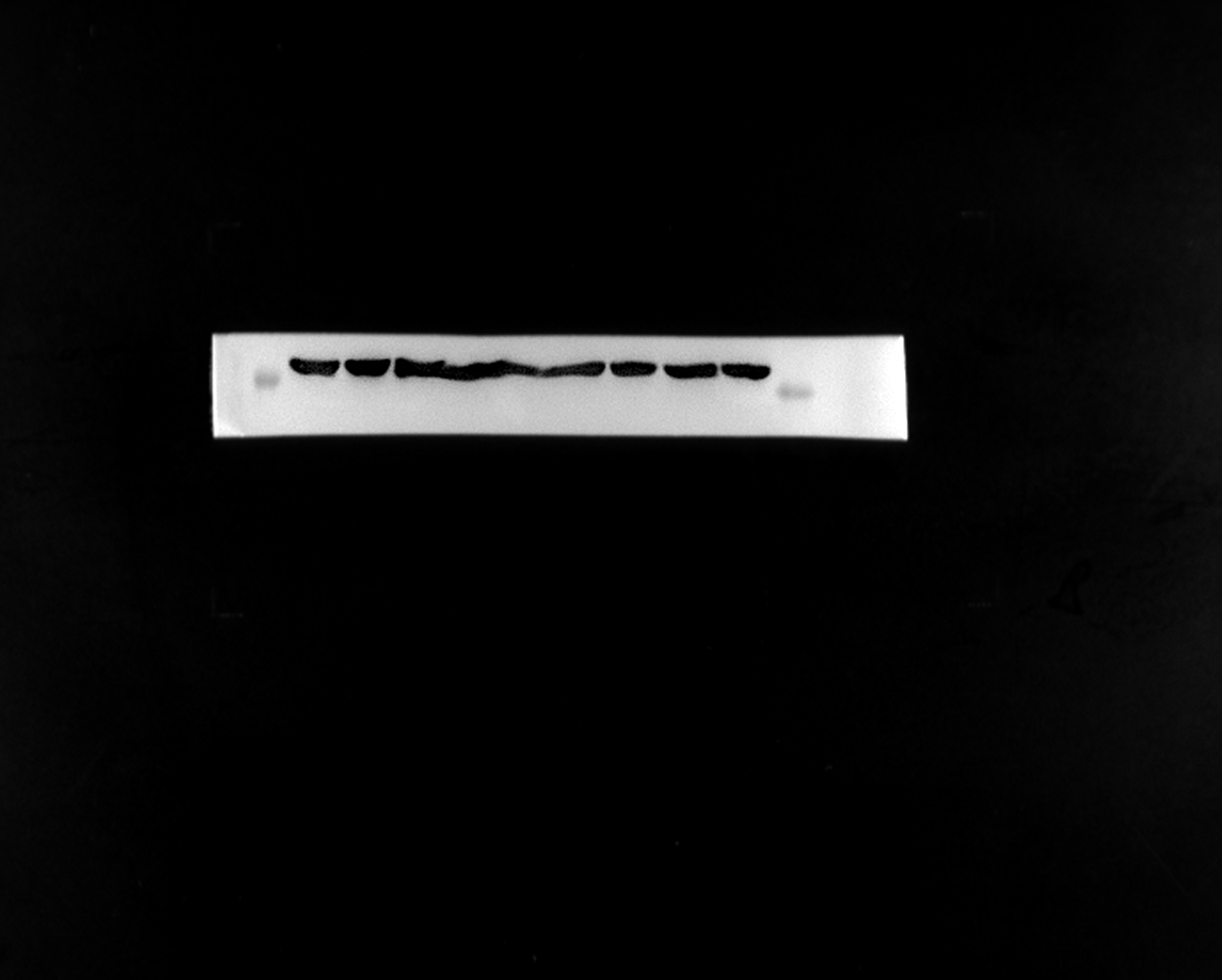

Supplement: Supplementary file 11 [file DataSheet10.ZIP › Western blot/Figure 4N/2/2-actins (ZO1).tif]

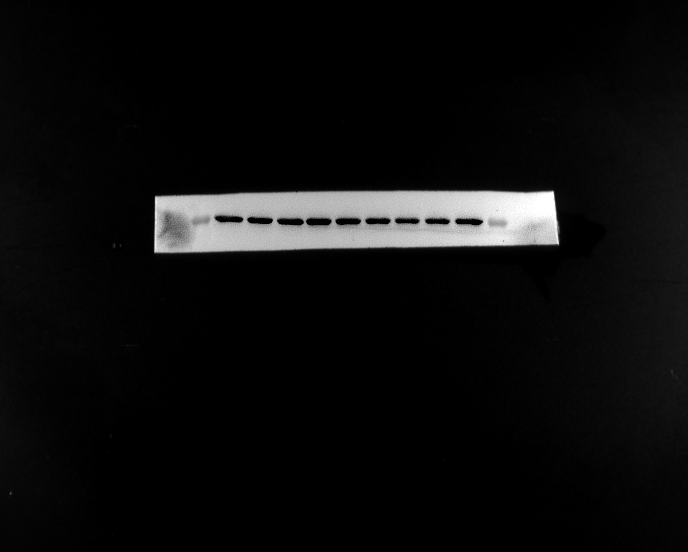

Supplement: Supplementary file 11 [file DataSheet10.ZIP › Western blot/Figure 4N/2/2-actins.tif]

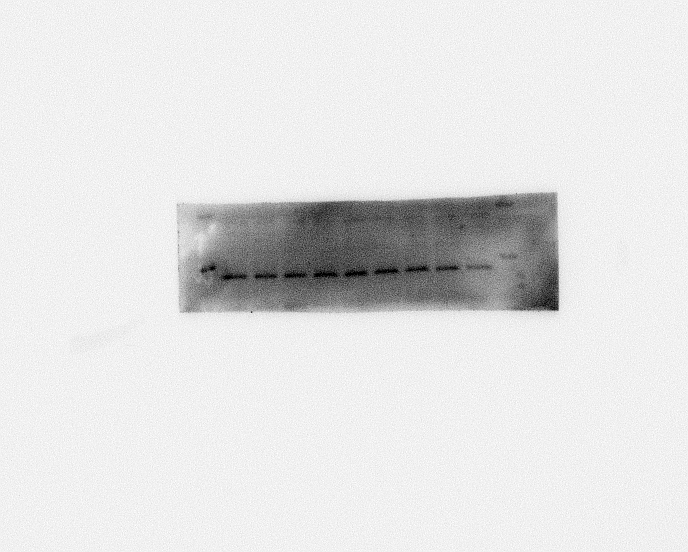

Supplement: Supplementary file 11 [file DataSheet10.ZIP › Western blot/Figure 4N/2/2-claudin.tif]

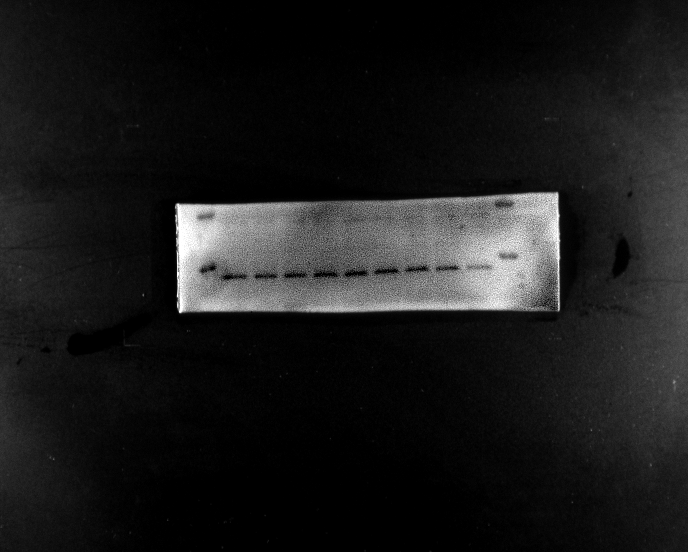

Supplement: Supplementary file 11 [file DataSheet10.ZIP › Western blot/Figure 4N/2/2-claudins.tif]

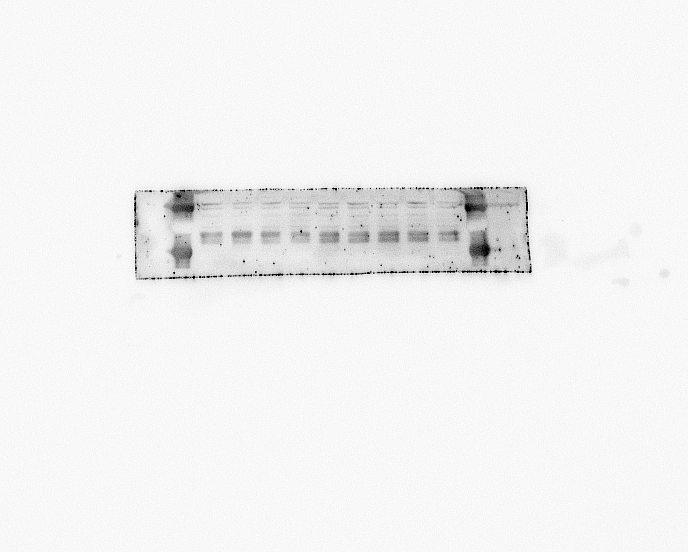

Supplement: Supplementary file 11 [file DataSheet10.ZIP › Western blot/Figure 4N/2/2-occludin.tif]

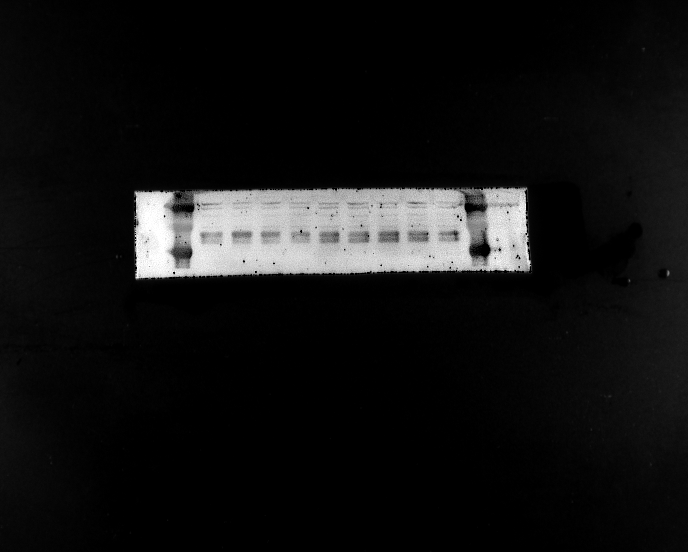

Supplement: Supplementary file 11 [file DataSheet10.ZIP › Western blot/Figure 4N/2/2-occludins.tif]

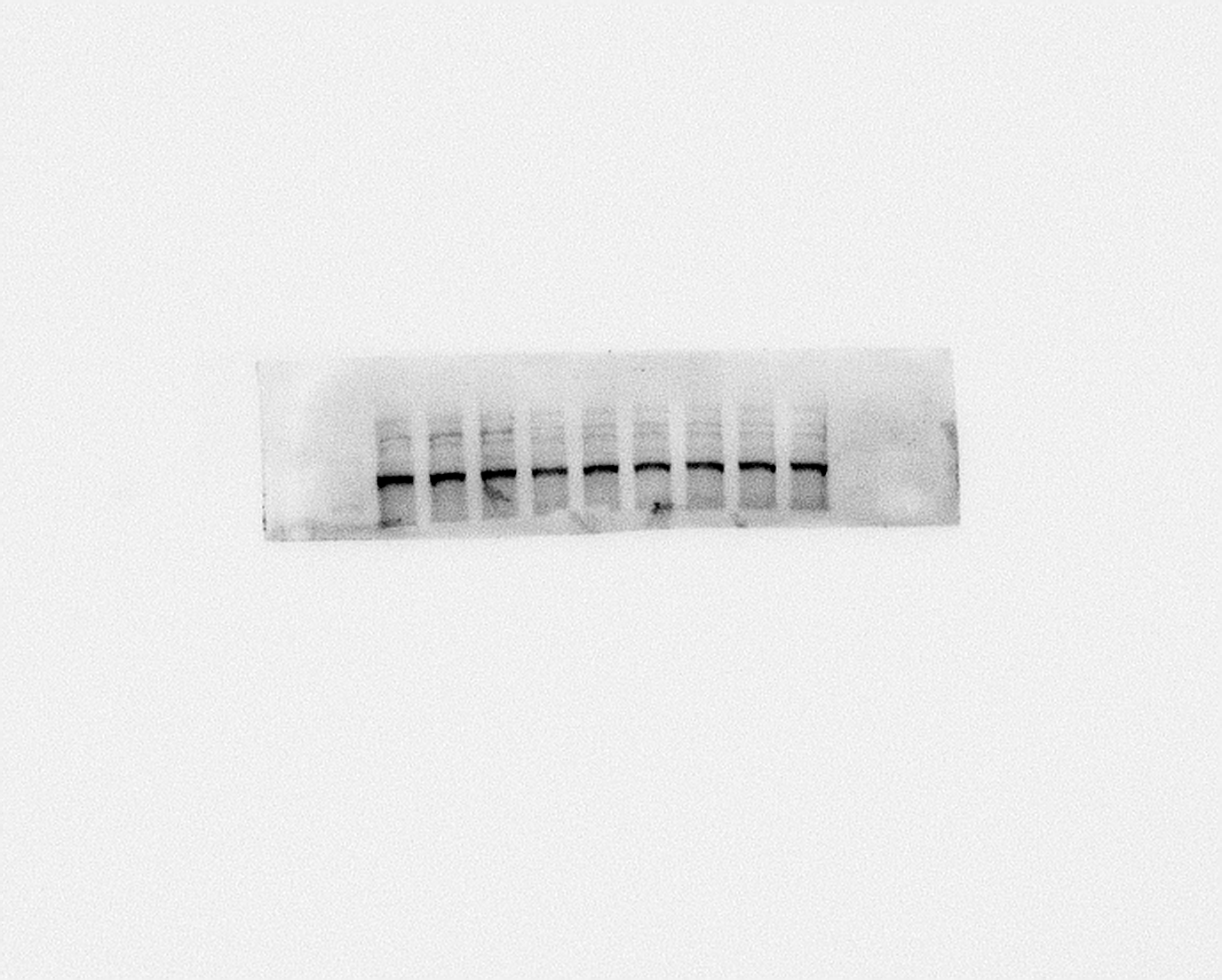

Supplement: Supplementary file 11 [file DataSheet10.ZIP › Western blot/Figure 4N/2/2-ZO1.tif]

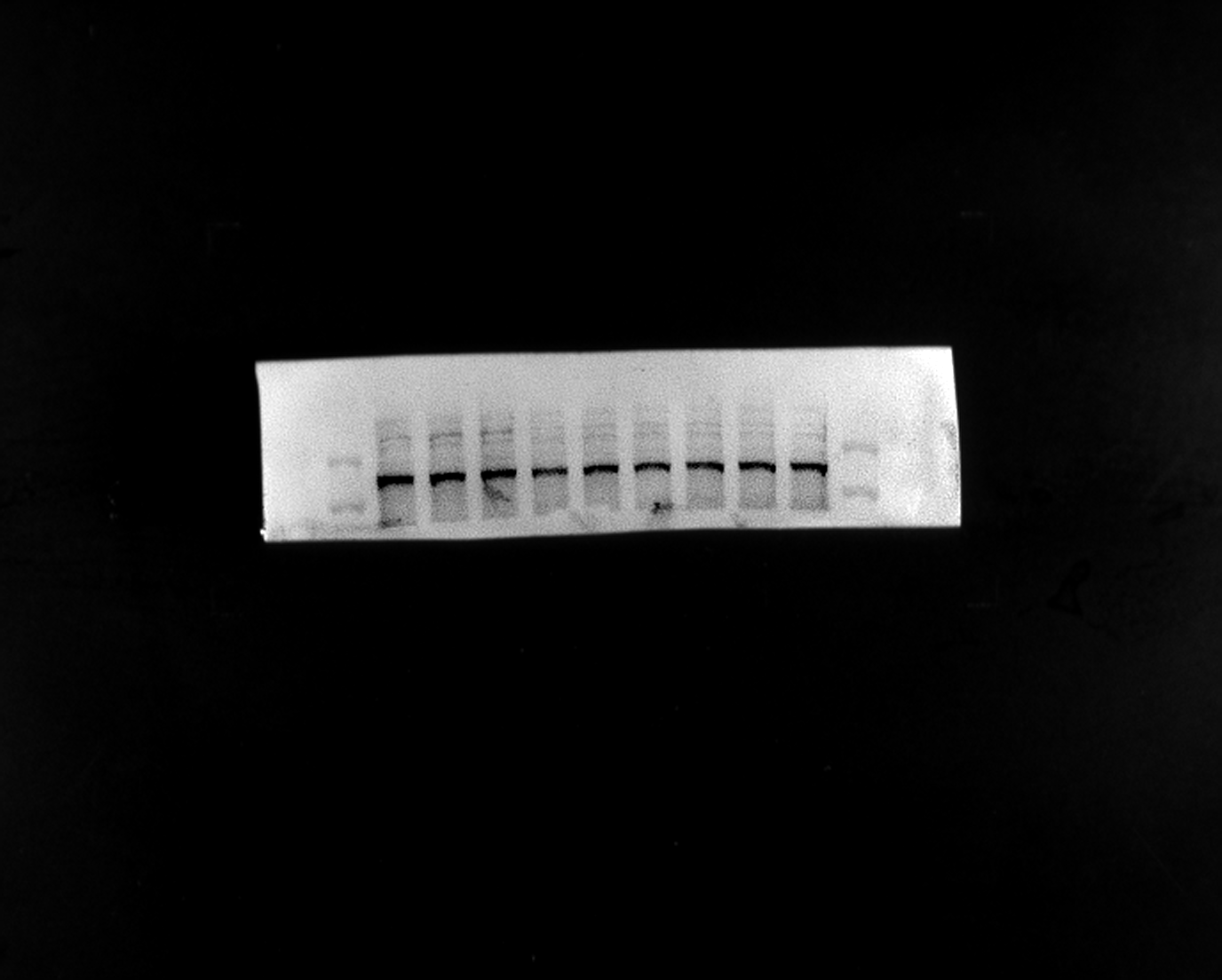

Supplement: Supplementary file 11 [file DataSheet10.ZIP › Western blot/Figure 4N/2/2-ZO1s.tif]

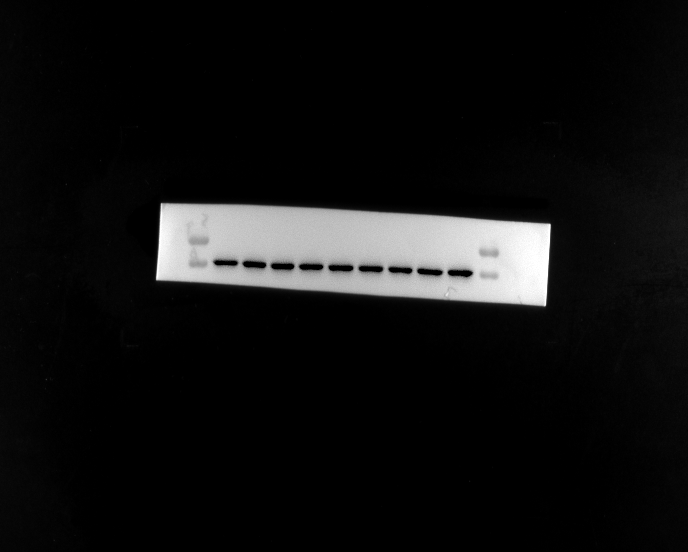

Supplement: Supplementary file 11 [file DataSheet10.ZIP › Western blot/Figure 5G/1 (presented in manuscript)/cyclin A, CDK2/1-1-actin-s.tif]

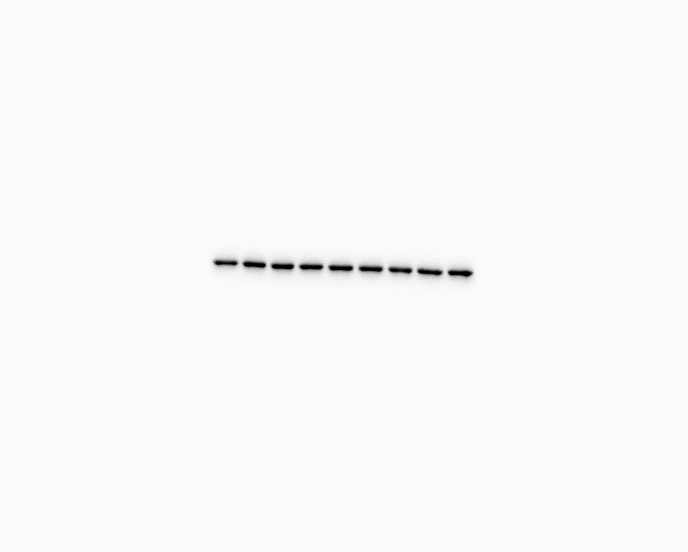

Supplement: Supplementary file 11 [file DataSheet10.ZIP › Western blot/Figure 5G/1 (presented in manuscript)/cyclin A, CDK2/1-1-actin.tif]

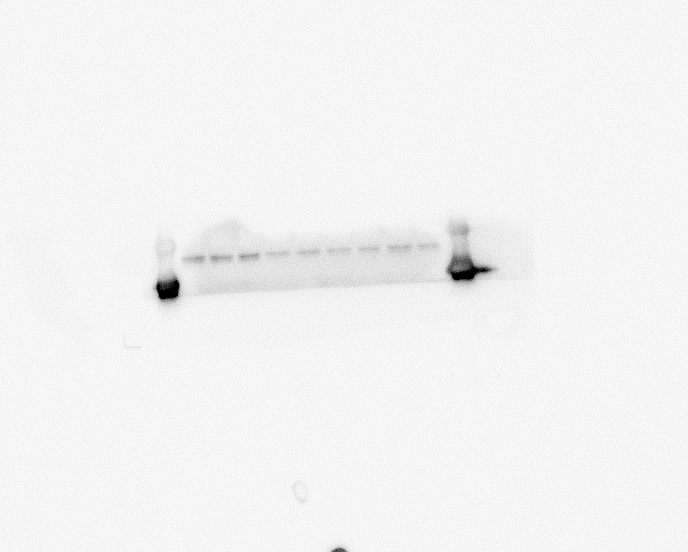

Supplement: Supplementary file 11 [file DataSheet10.ZIP › Western blot/Figure 5G/1 (presented in manuscript)/cyclin A, CDK2/1-CDK2.tif]

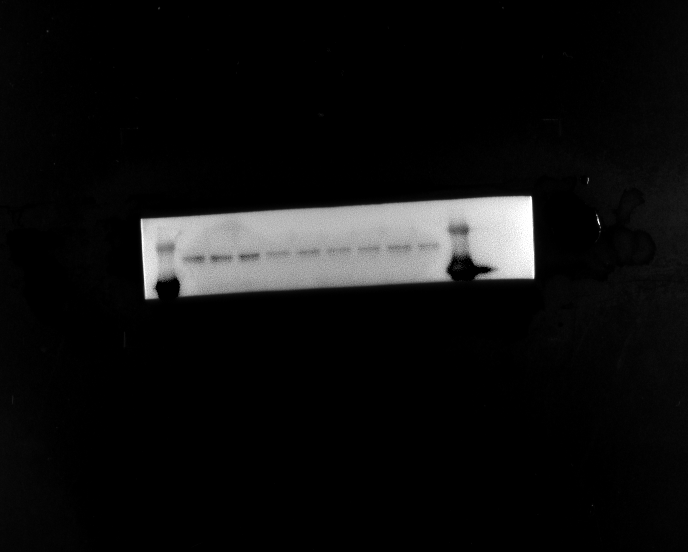

Supplement: Supplementary file 11 [file DataSheet10.ZIP › Western blot/Figure 5G/1 (presented in manuscript)/cyclin A, CDK2/1-CDK2s.tif]

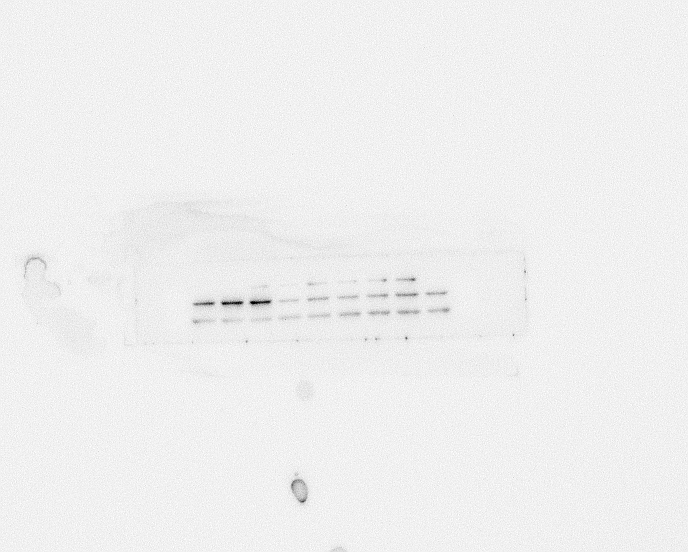

Supplement: Supplementary file 11 [file DataSheet10.ZIP › Western blot/Figure 5G/1 (presented in manuscript)/cyclin A, CDK2/1-cyclinA.tif]

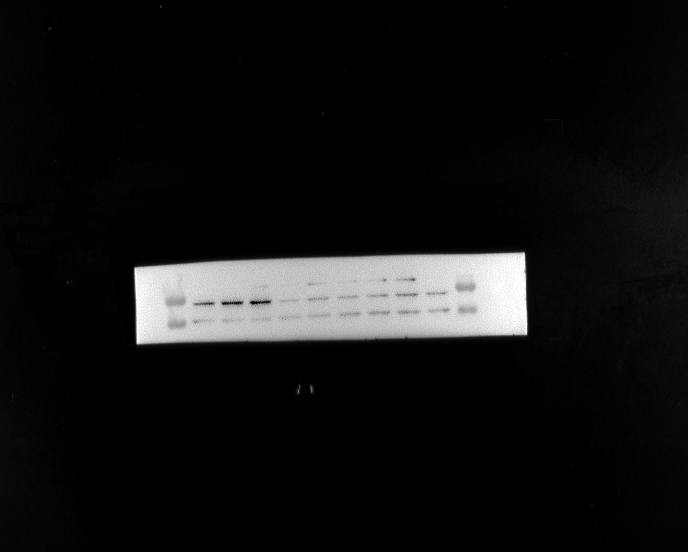

Supplement: Supplementary file 11 [file DataSheet10.ZIP › Western blot/Figure 5G/1 (presented in manuscript)/cyclin A, CDK2/1-cyclinAs.tif]

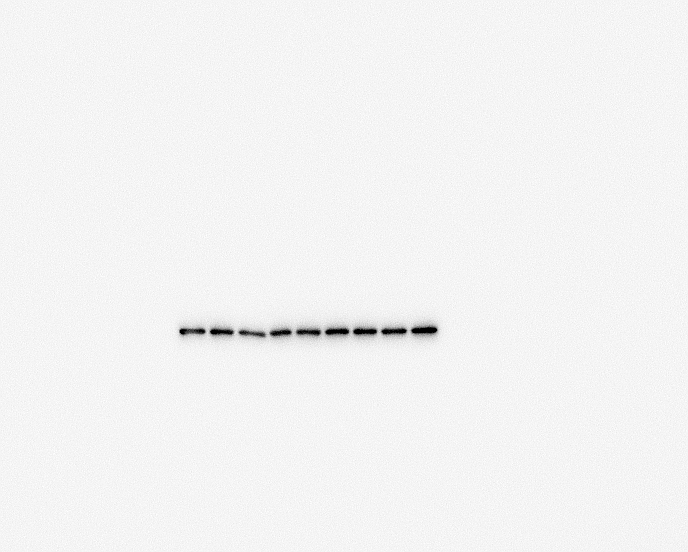

Supplement: Supplementary file 11 [file DataSheet10.ZIP › Western blot/Figure 5G/1 (presented in manuscript)/cyclin D, CDK4/1-2-actin.tif]

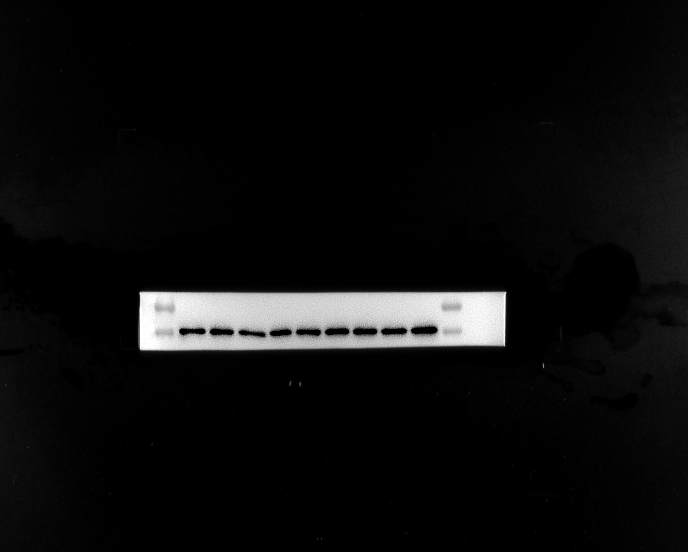

Supplement: Supplementary file 11 [file DataSheet10.ZIP › Western blot/Figure 5G/1 (presented in manuscript)/cyclin D, CDK4/1-2-actins.tif]

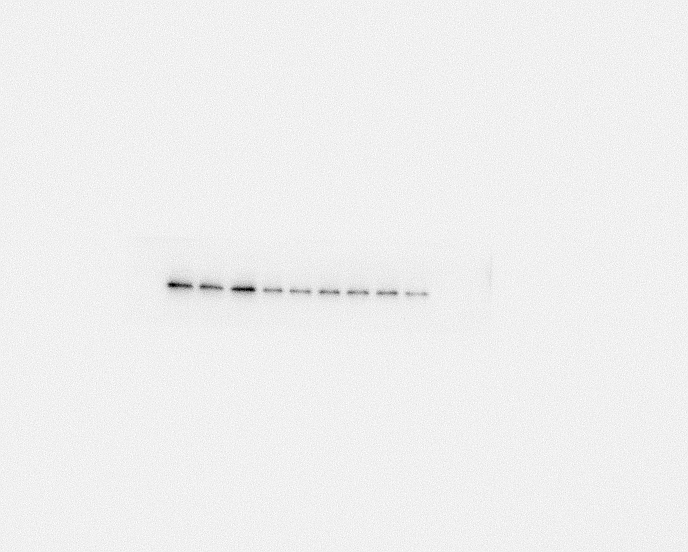

Supplement: Supplementary file 11 [file DataSheet10.ZIP › Western blot/Figure 5G/1 (presented in manuscript)/cyclin D, CDK4/1-CDK4.tif]

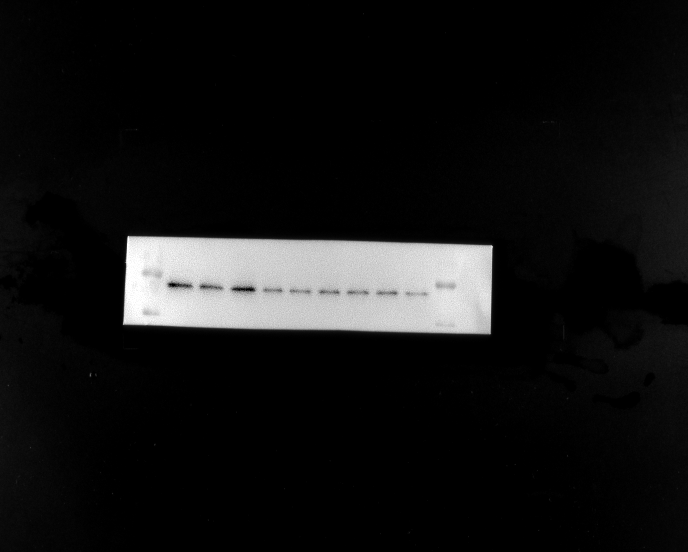

Supplement: Supplementary file 11 [file DataSheet10.ZIP › Western blot/Figure 5G/1 (presented in manuscript)/cyclin D, CDK4/1-CDK4s.tif]

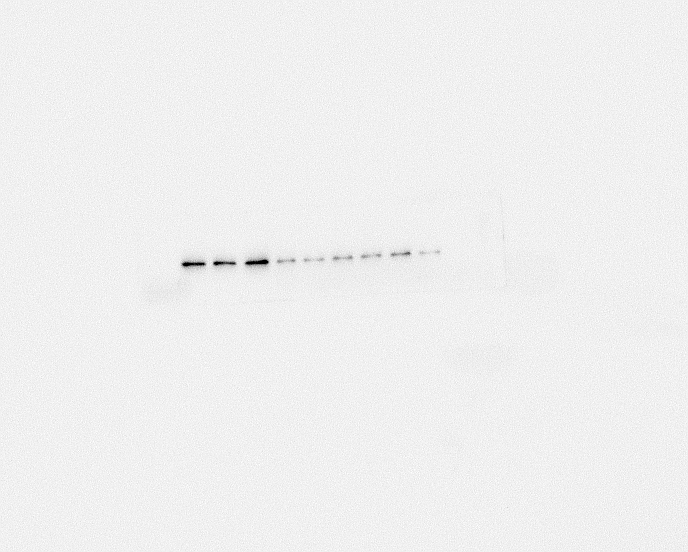

Supplement: Supplementary file 11 [file DataSheet10.ZIP › Western blot/Figure 5G/1 (presented in manuscript)/cyclin D, CDK4/1-cyclinD.tif]

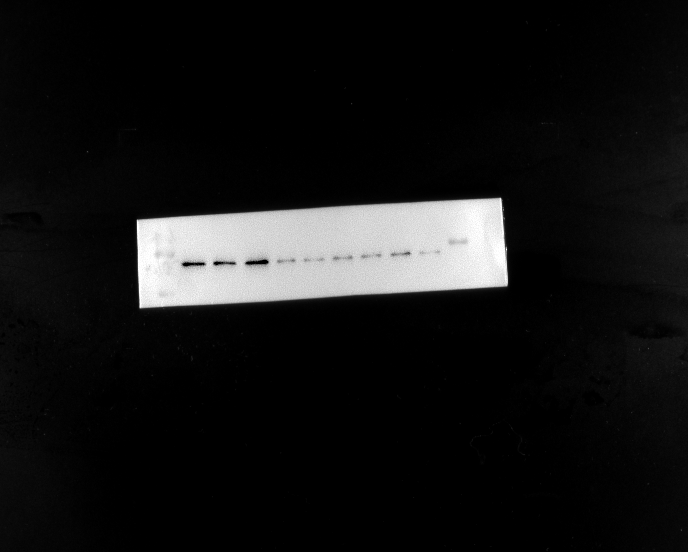

Supplement: Supplementary file 11 [file DataSheet10.ZIP › Western blot/Figure 5G/1 (presented in manuscript)/cyclin D, CDK4/1-cyclinDs.tif]

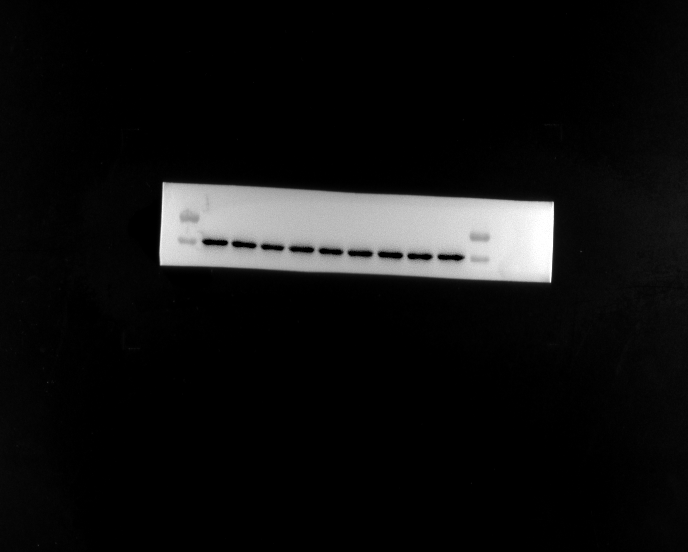

Supplement: Supplementary file 11 [file DataSheet10.ZIP › Western blot/Figure 5G/2/CDK4/2-3-actin-s.tif]

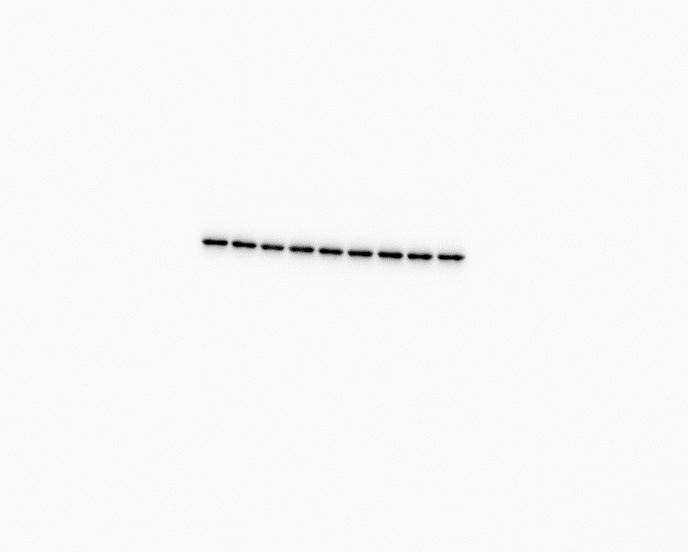

Supplement: Supplementary file 11 [file DataSheet10.ZIP › Western blot/Figure 5G/2/CDK4/2-3-actin.tif]

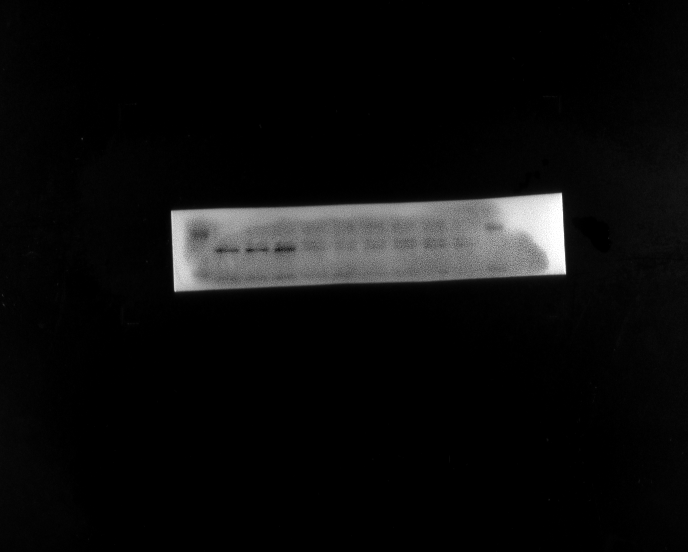

Supplement: Supplementary file 11 [file DataSheet10.ZIP › Western blot/Figure 5G/2/CDK4/2-CDK4-s.tif]

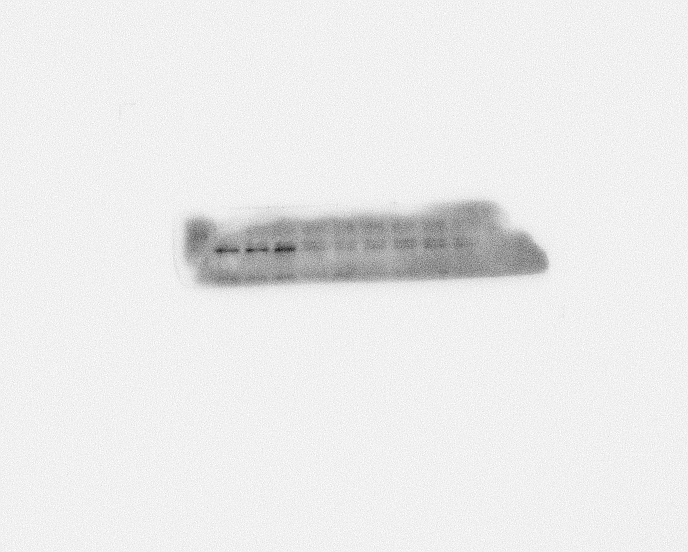

Supplement: Supplementary file 11 [file DataSheet10.ZIP › Western blot/Figure 5G/2/CDK4/2-CDK4.tif]

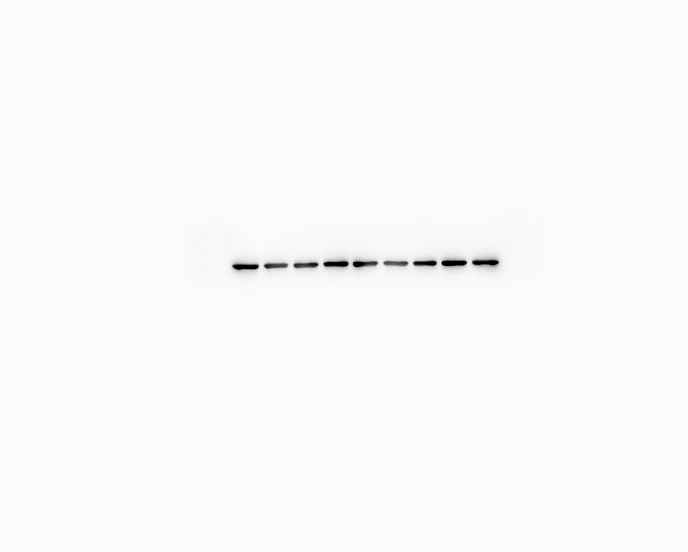

Supplement: Supplementary file 11 [file DataSheet10.ZIP › Western blot/Figure 5G/2/cyclin A, CDK2/2-1-actin.tif]

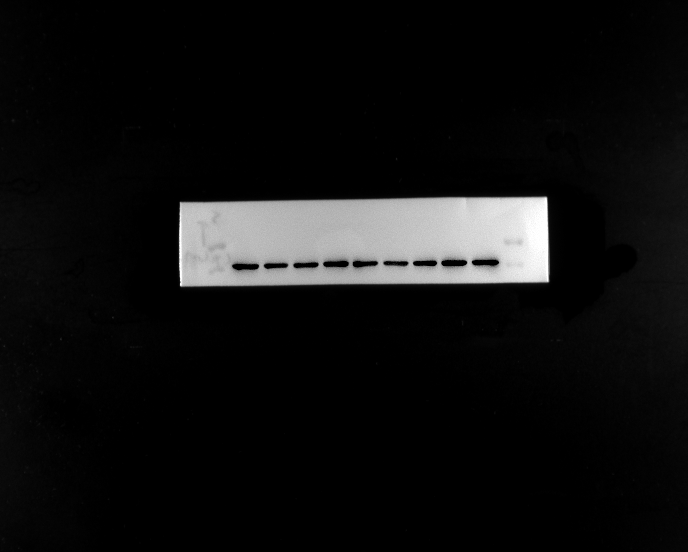

Supplement: Supplementary file 11 [file DataSheet10.ZIP › Western blot/Figure 5G/2/cyclin A, CDK2/2-1-actins.tif]

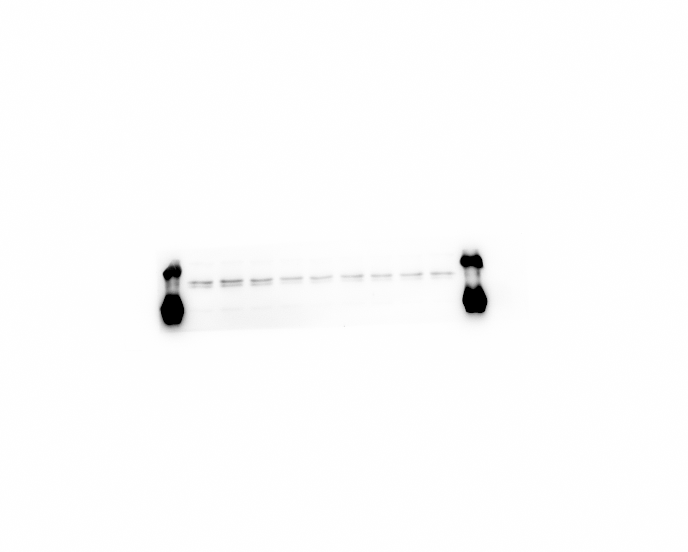

Supplement: Supplementary file 11 [file DataSheet10.ZIP › Western blot/Figure 5G/2/cyclin A, CDK2/2-CDK2.tif]

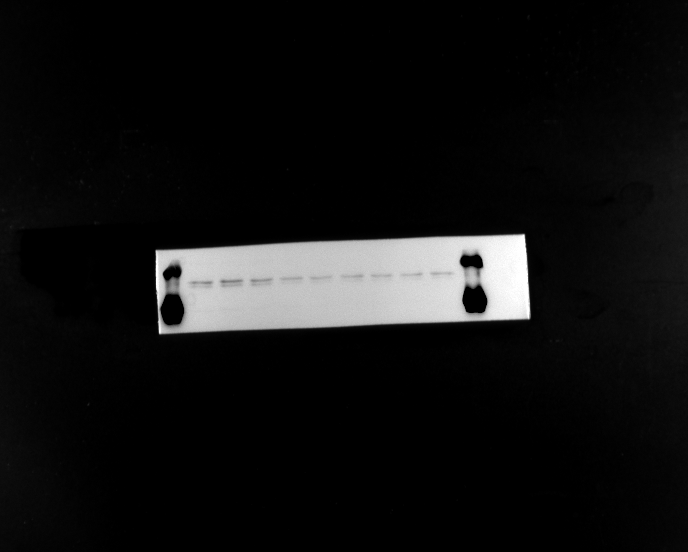

Supplement: Supplementary file 11 [file DataSheet10.ZIP › Western blot/Figure 5G/2/cyclin A, CDK2/2-CDK2s.tif]

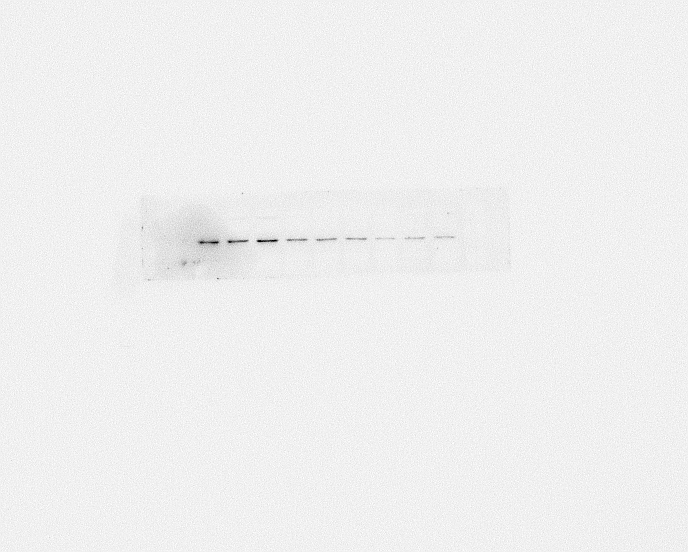

Supplement: Supplementary file 11 [file DataSheet10.ZIP › Western blot/Figure 5G/2/cyclin A, CDK2/2-cyclinA.tif]

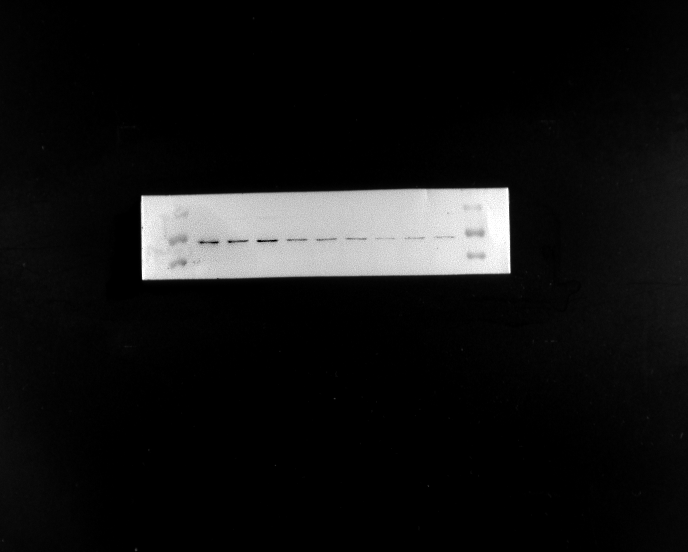

Supplement: Supplementary file 11 [file DataSheet10.ZIP › Western blot/Figure 5G/2/cyclin A, CDK2/2-cyclinAs.tif]

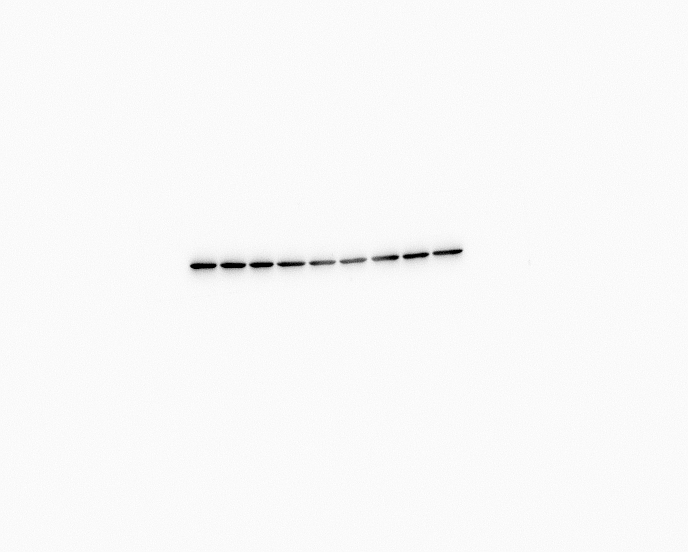

Supplement: Supplementary file 11 [file DataSheet10.ZIP › Western blot/Figure 5G/2/cyclin D/2-2-actin.tif]

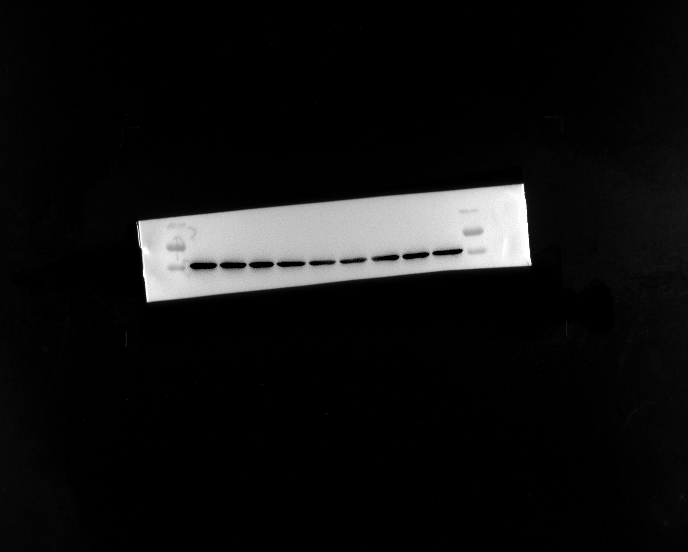

Supplement: Supplementary file 11 [file DataSheet10.ZIP › Western blot/Figure 5G/2/cyclin D/2-2-actins.tif]
