# Supplementary material for: Hepatic Ischemia-Reperfusion Impairs Blood-Brain Barrier Partly Due to Release of Arginase From Injured Liver
Source: Front Pharmacol. 2021 Oct 13;12:724471. doi: 10.3389/fphar.2021.724471 (PMC8548691; doi:10.3389/fphar.2021.724471)
Supplement: Supplementary file 9 [file Table7.DOCX]

**Supplementary Figure S1.** Role of oxidative stress in arginase induced hCMEC/D3 cells damage. (A) Reactive oxide species (ROS) assay in hCMEC/D3 cells treated with arginine-free medium ((-)Arg) or 20 μg/mL arginase (Ase) (n=4, **p*<0.05 vs control cells (Ctrl)). (B) Effect of free radical scavenger N-Acetyl-L-cysteine (NAC) (8 mM) on damaged cell viability caused by (-)Arg or Ase (n=4, ns, no significance vs cells treated with vehicle).

**Supplementary Figure S2.** Involvement of NO in arginase induced hCMEC/D3 cells damage. (A) Effect of eNOS inhibitor (L-NAME) on hCMEC/D3 cells viability (n=4, ****p*<0.001 vs control cells (Ctrl)). (B) Effect of NO donor sodium nitroprusside (SNP) on reduced cell viability caused by arginine-free medium ((-)Arg) or 20 μg/mL arginase (Ase) (n=4, ns, no significance vs cells without SNP treatment).
