# Supplementary figures and images for: Hepatic Ischemia-Reperfusion Impairs Blood-Brain Barrier Partly Due to Release of Arginase From Injured Liver (part 2 of 3)
Source: Front Pharmacol. 2021 Oct 13;12:724471. doi: 10.3389/fphar.2021.724471 (PMC8548691; doi:10.3389/fphar.2021.724471)

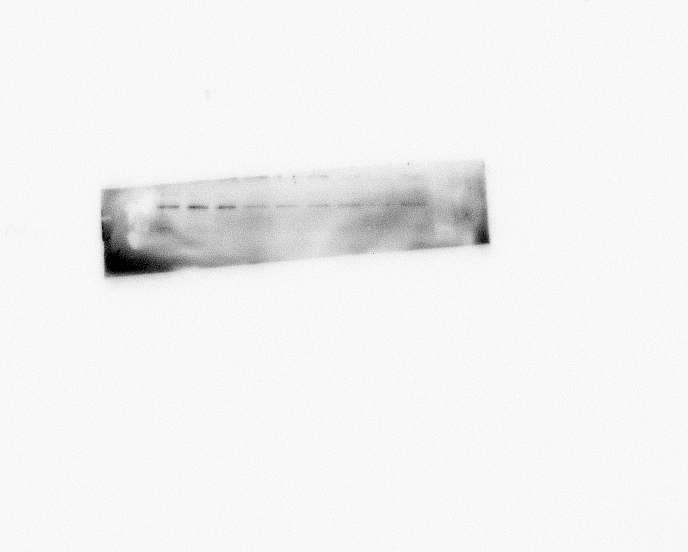

Supplement: Supplementary file 11 [file DataSheet10.ZIP › Western blot/Figure 5G/2/cyclin D/2-cyclinD.tif]

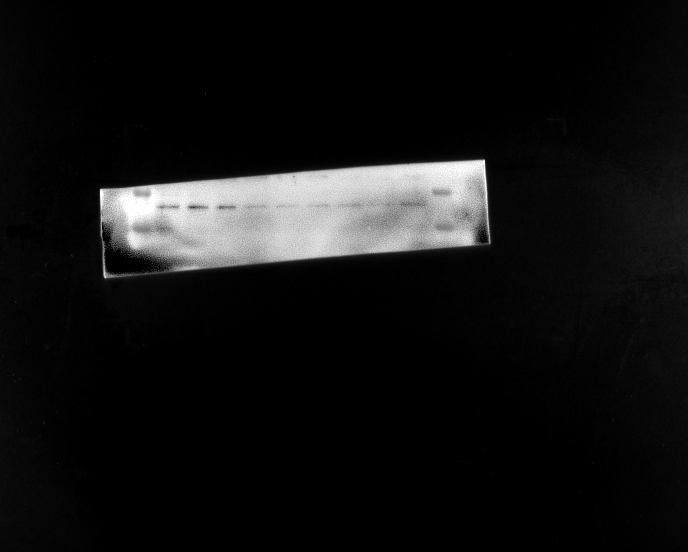

Supplement: Supplementary file 11 [file DataSheet10.ZIP › Western blot/Figure 5G/2/cyclin D/2-cyclinDs.tif]

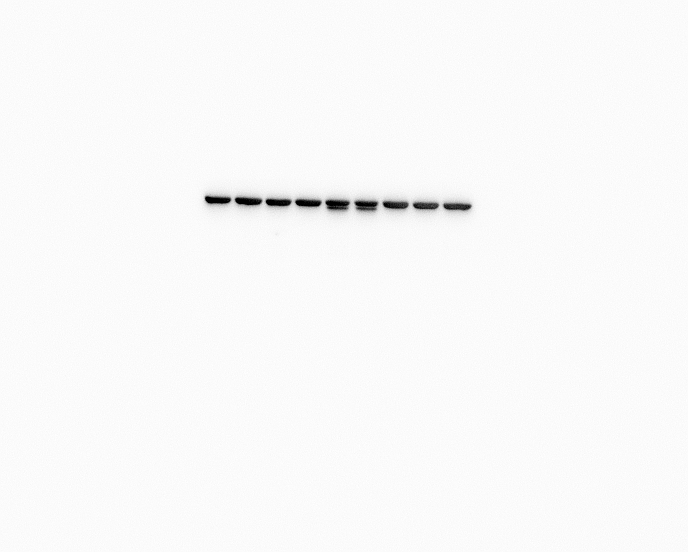

Supplement: Supplementary file 11 [file DataSheet10.ZIP › Western blot/Figure 5H/1 (presented in manuscript)/CDK1, CDK6/1-2-actin.tif]

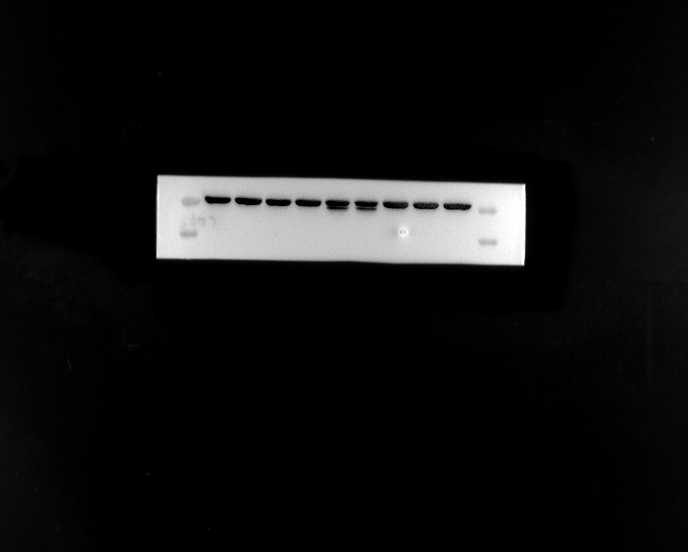

Supplement: Supplementary file 11 [file DataSheet10.ZIP › Western blot/Figure 5H/1 (presented in manuscript)/CDK1, CDK6/1-2-actins.tif]

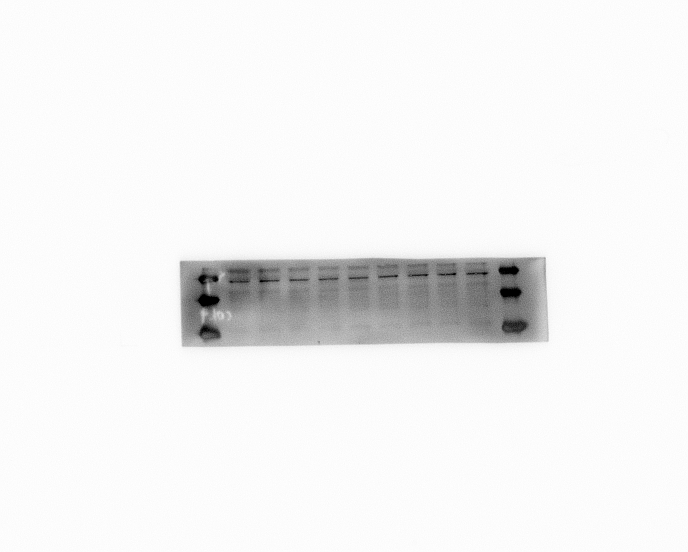

Supplement: Supplementary file 11 [file DataSheet10.ZIP › Western blot/Figure 5H/1 (presented in manuscript)/CDK1, CDK6/1-CDK1.tif]

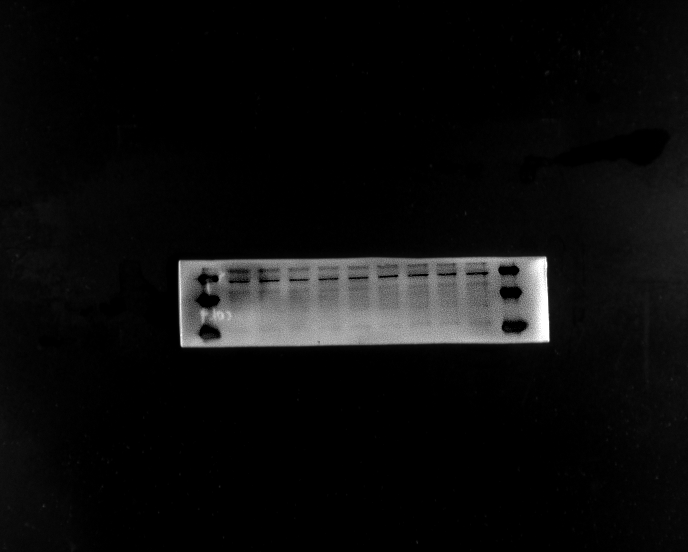

Supplement: Supplementary file 11 [file DataSheet10.ZIP › Western blot/Figure 5H/1 (presented in manuscript)/CDK1, CDK6/1-CDK1s.tif]

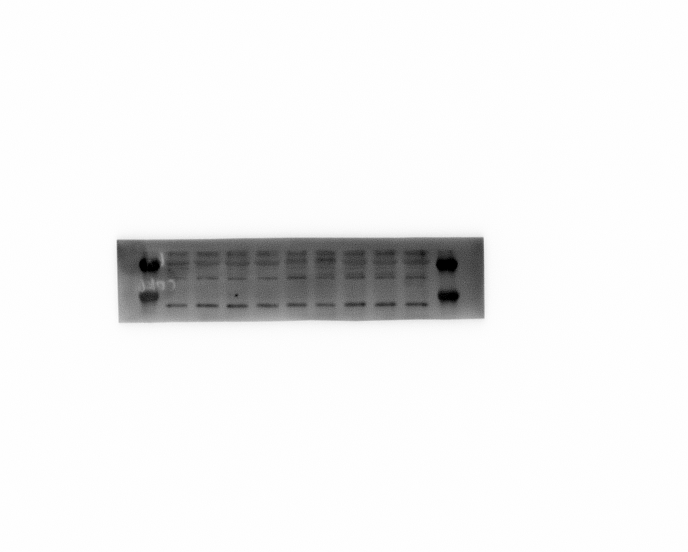

Supplement: Supplementary file 11 [file DataSheet10.ZIP › Western blot/Figure 5H/1 (presented in manuscript)/CDK1, CDK6/1-CDK6.tif]

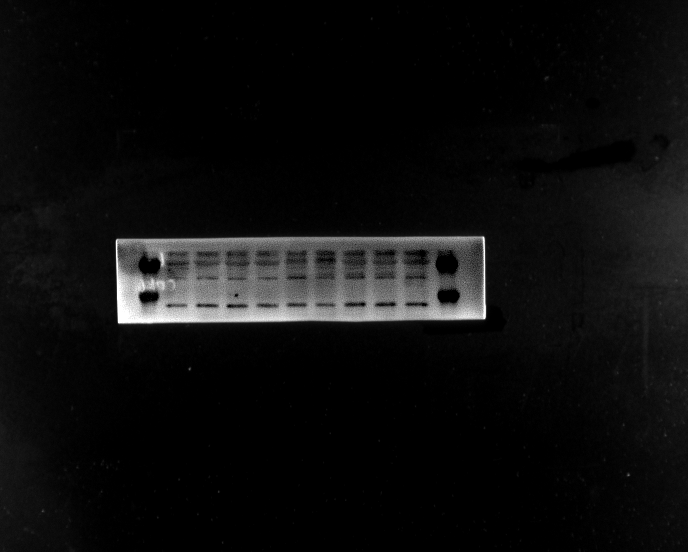

Supplement: Supplementary file 11 [file DataSheet10.ZIP › Western blot/Figure 5H/1 (presented in manuscript)/CDK1, CDK6/1-CDK6s.tif]

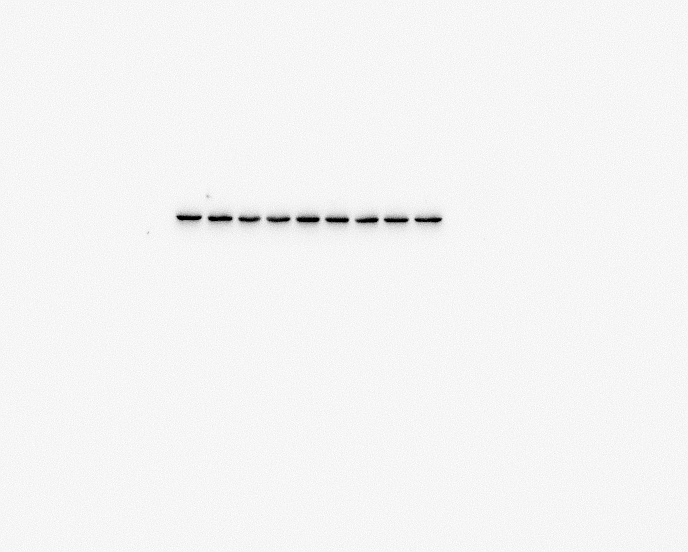

Supplement: Supplementary file 11 [file DataSheet10.ZIP › Western blot/Figure 5H/1 (presented in manuscript)/cyclin B, cyclin E/1-1-actin.tif]

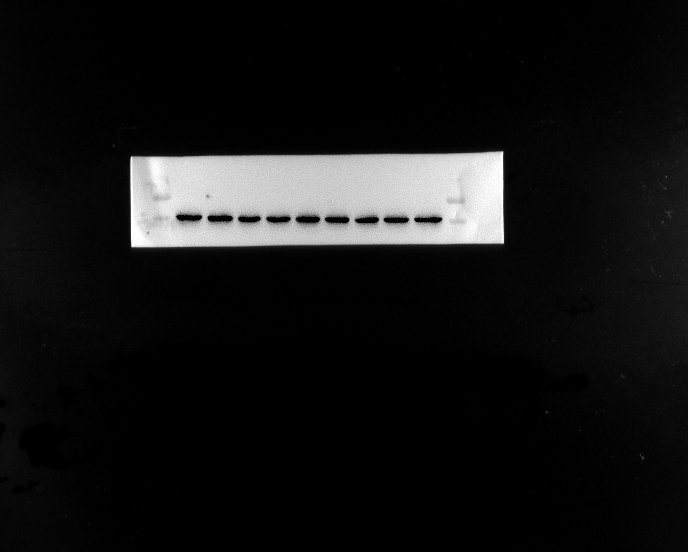

Supplement: Supplementary file 11 [file DataSheet10.ZIP › Western blot/Figure 5H/1 (presented in manuscript)/cyclin B, cyclin E/1-1-actins.tif]

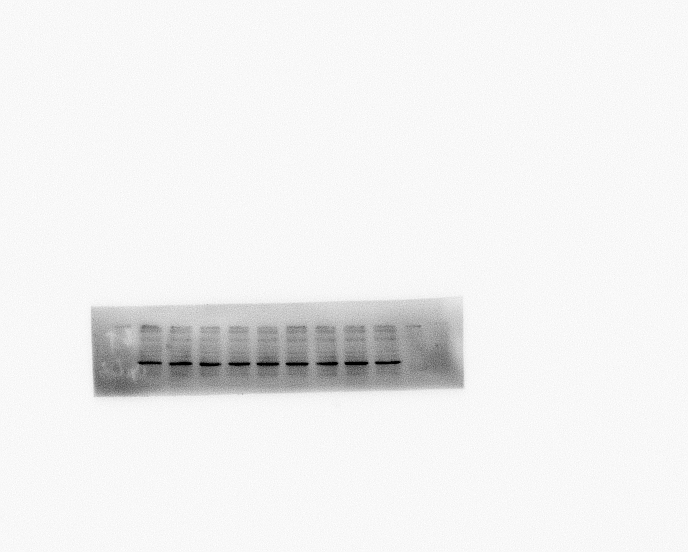

Supplement: Supplementary file 11 [file DataSheet10.ZIP › Western blot/Figure 5H/1 (presented in manuscript)/cyclin B, cyclin E/1-cyclinB.tif]

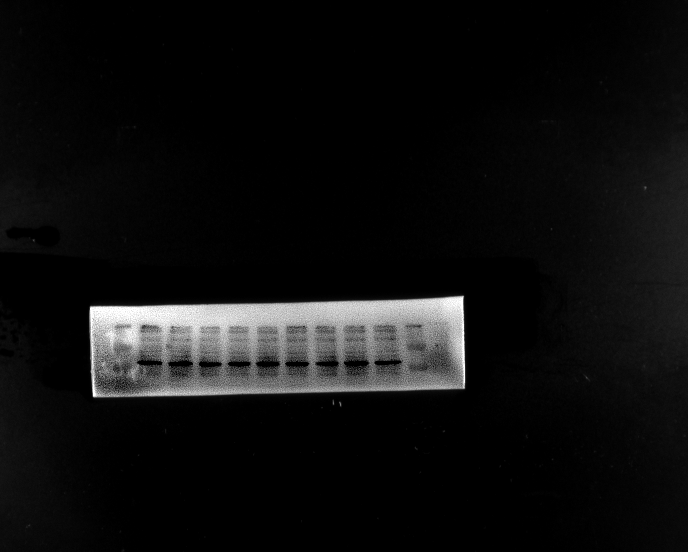

Supplement: Supplementary file 11 [file DataSheet10.ZIP › Western blot/Figure 5H/1 (presented in manuscript)/cyclin B, cyclin E/1-cyclinBs.tif]

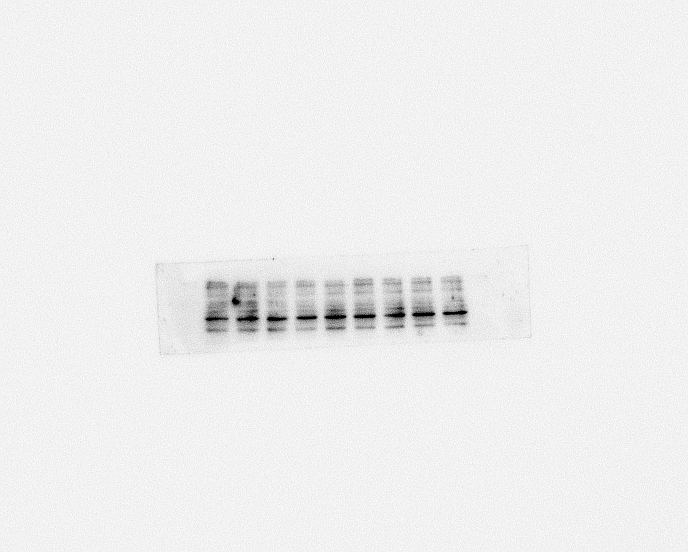

Supplement: Supplementary file 11 [file DataSheet10.ZIP › Western blot/Figure 5H/1 (presented in manuscript)/cyclin B, cyclin E/1-cyclinE.tif]

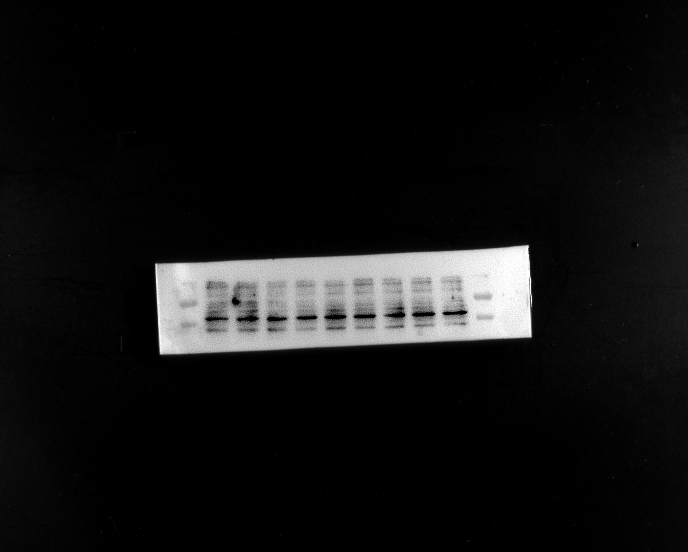

Supplement: Supplementary file 11 [file DataSheet10.ZIP › Western blot/Figure 5H/1 (presented in manuscript)/cyclin B, cyclin E/1-cyclinEs.tif]

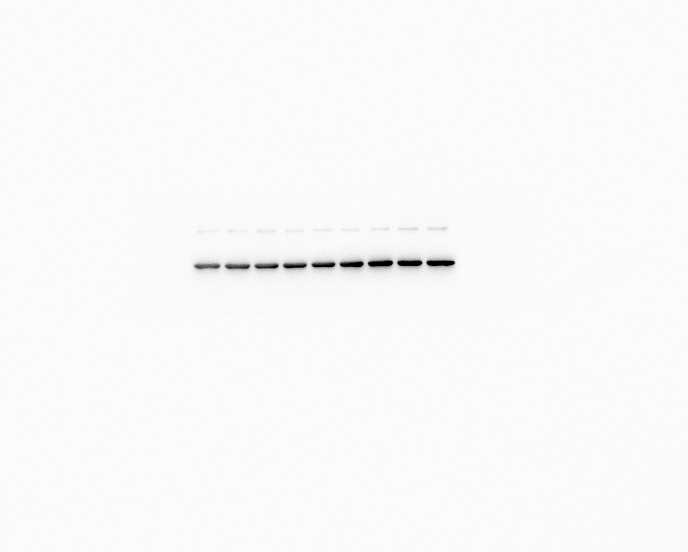

Supplement: Supplementary file 11 [file DataSheet10.ZIP › Western blot/Figure 5H/2/CDK1/2-3-actin.tif]

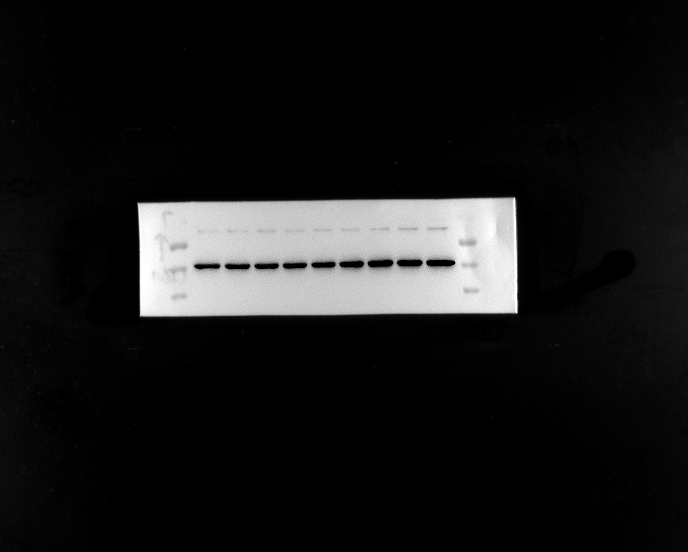

Supplement: Supplementary file 11 [file DataSheet10.ZIP › Western blot/Figure 5H/2/CDK1/2-3-actins.tif]

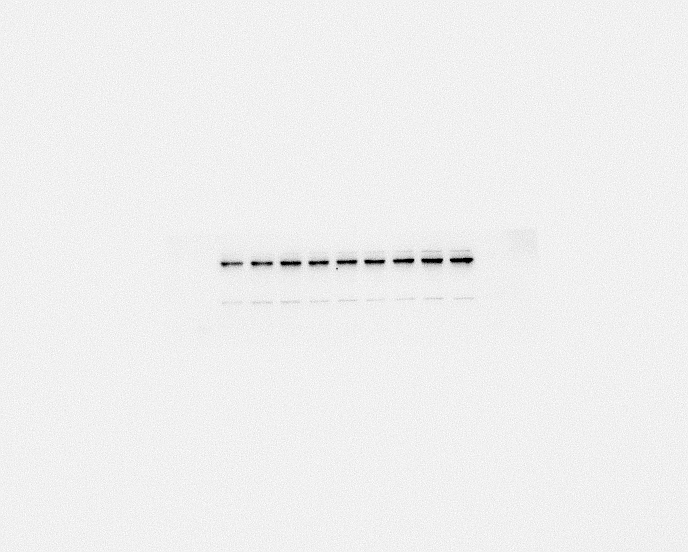

Supplement: Supplementary file 11 [file DataSheet10.ZIP › Western blot/Figure 5H/2/CDK1/2-CDK1.tif]

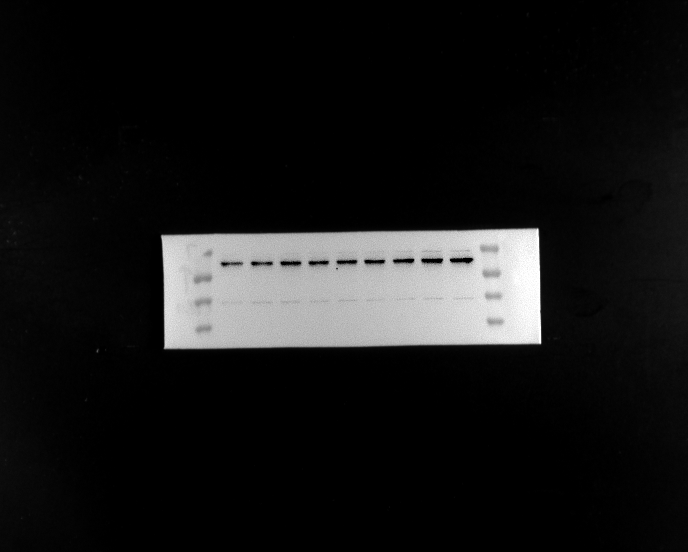

Supplement: Supplementary file 11 [file DataSheet10.ZIP › Western blot/Figure 5H/2/CDK1/2-CDK1s.tif]

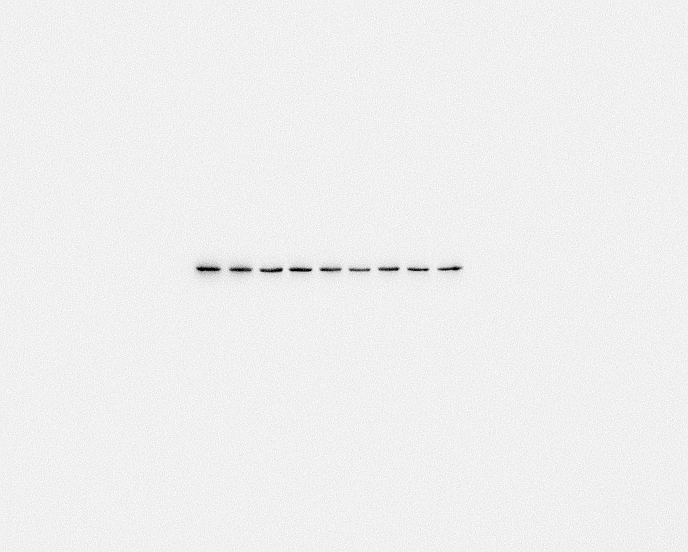

Supplement: Supplementary file 11 [file DataSheet10.ZIP › Western blot/Figure 5H/2/CDK6/2-4-actin.tif]

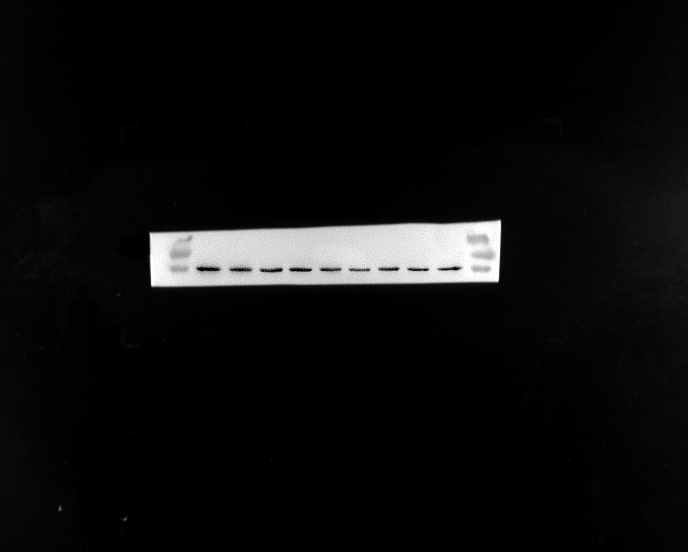

Supplement: Supplementary file 11 [file DataSheet10.ZIP › Western blot/Figure 5H/2/CDK6/2-4-actins.tif]

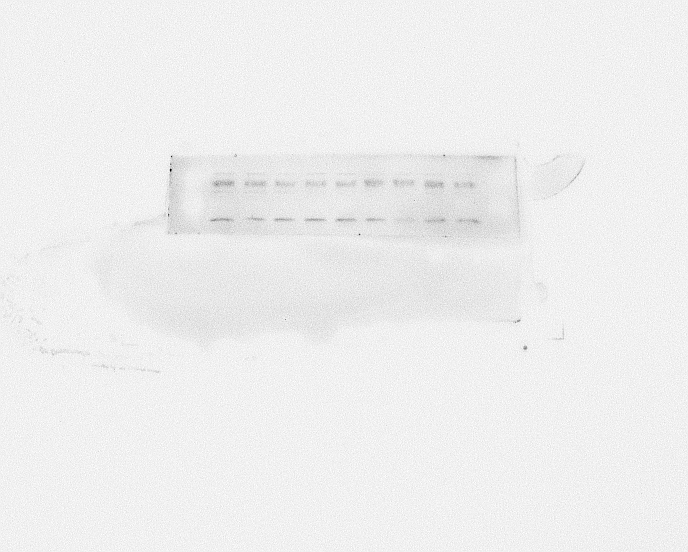

Supplement: Supplementary file 11 [file DataSheet10.ZIP › Western blot/Figure 5H/2/CDK6/2-CDK6.tif]

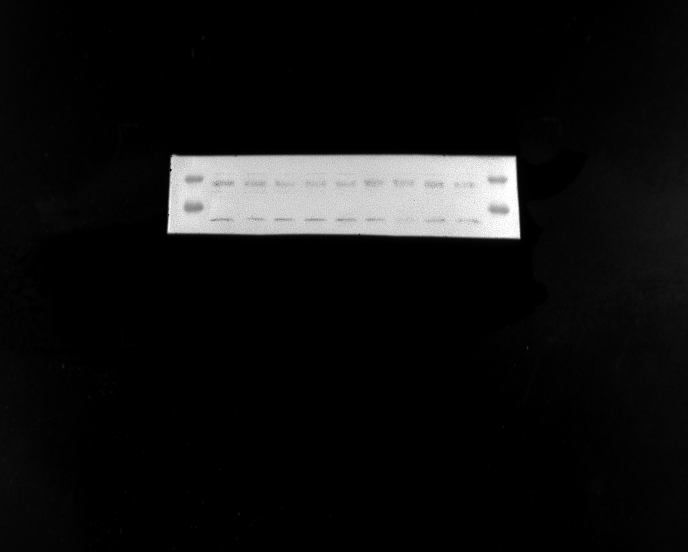

Supplement: Supplementary file 11 [file DataSheet10.ZIP › Western blot/Figure 5H/2/CDK6/2-CDK6s.tif]

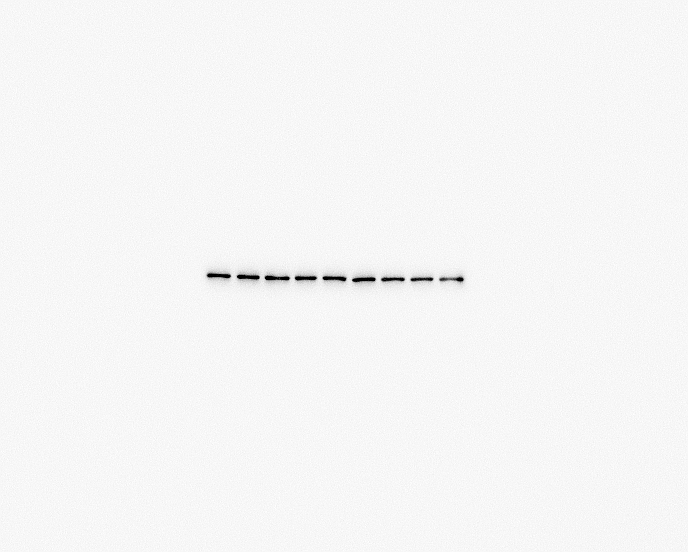

Supplement: Supplementary file 11 [file DataSheet10.ZIP › Western blot/Figure 5H/2/cyclin B/2-1-actin.tif]

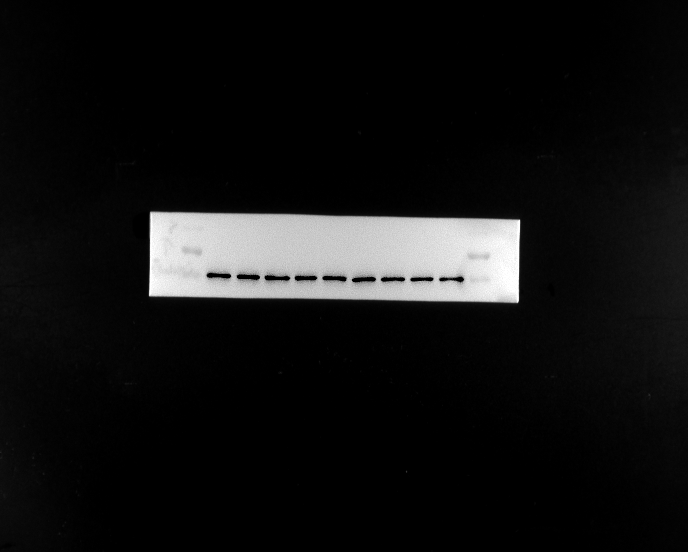

Supplement: Supplementary file 11 [file DataSheet10.ZIP › Western blot/Figure 5H/2/cyclin B/2-1-actins.tif]

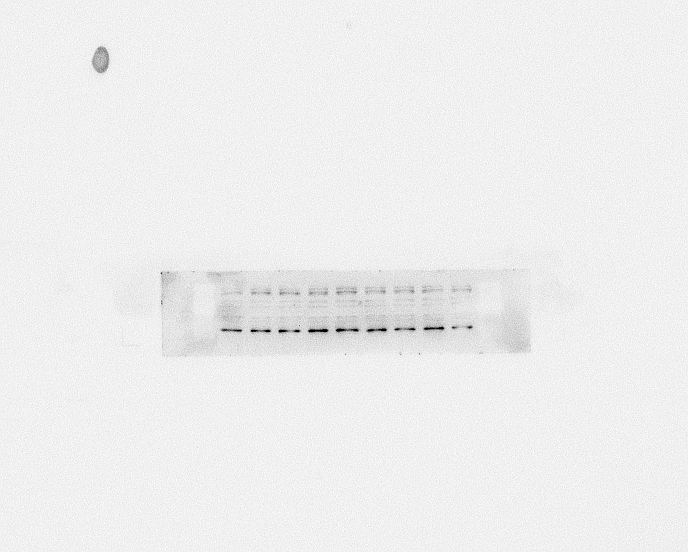

Supplement: Supplementary file 11 [file DataSheet10.ZIP › Western blot/Figure 5H/2/cyclin B/2-cyclin B.tif]

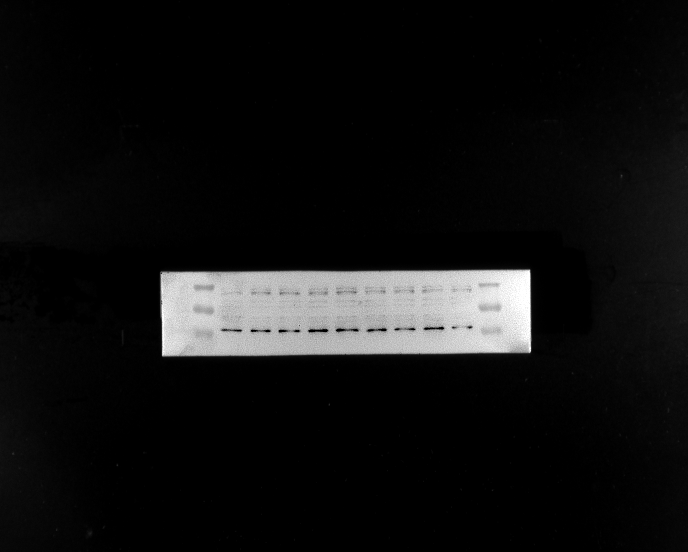

Supplement: Supplementary file 11 [file DataSheet10.ZIP › Western blot/Figure 5H/2/cyclin B/2-cyclin Bs.tif]

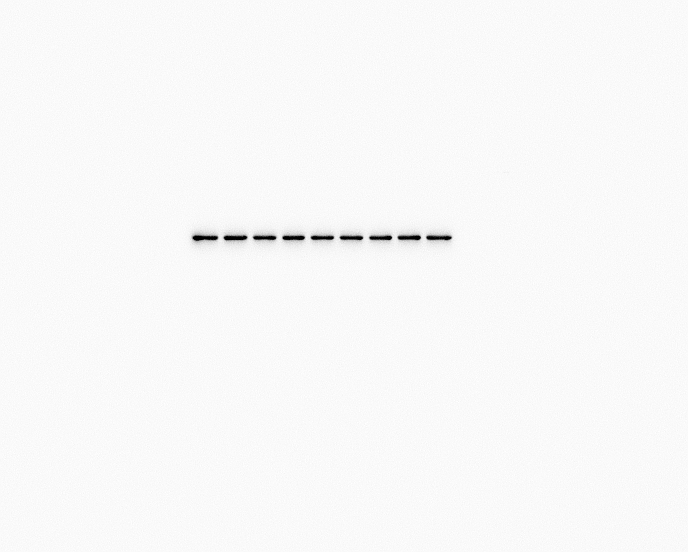

Supplement: Supplementary file 11 [file DataSheet10.ZIP › Western blot/Figure 5H/2/cyclin E/2-1-actin.tif]

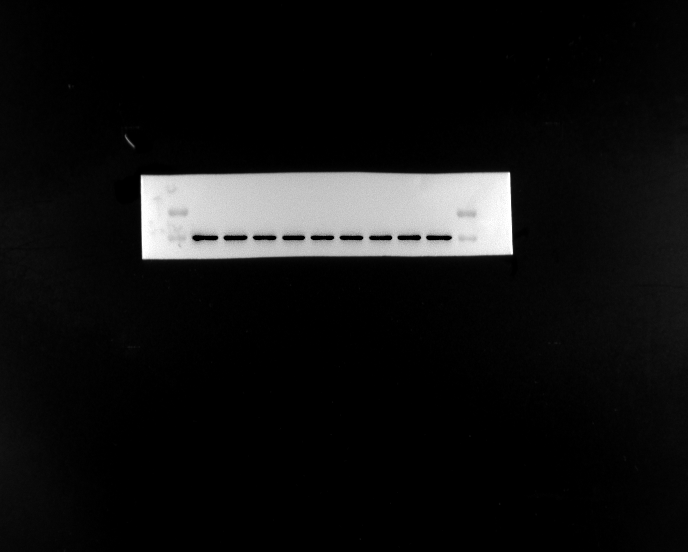

Supplement: Supplementary file 11 [file DataSheet10.ZIP › Western blot/Figure 5H/2/cyclin E/2-1-actins.tif]

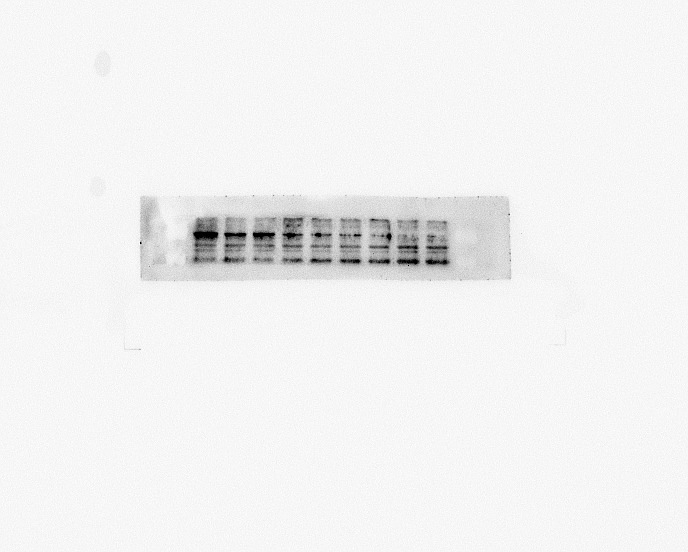

Supplement: Supplementary file 11 [file DataSheet10.ZIP › Western blot/Figure 5H/2/cyclin E/2-cyclin E.tif]

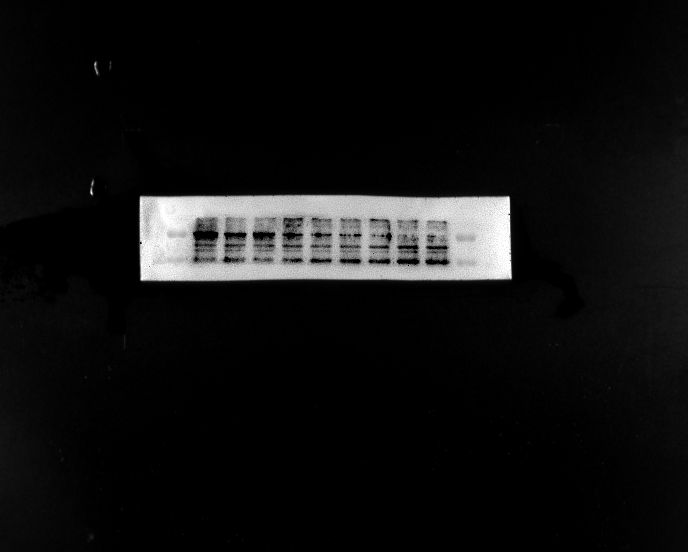

Supplement: Supplementary file 11 [file DataSheet10.ZIP › Western blot/Figure 5H/2/cyclin E/2-cyclin Es.tif]

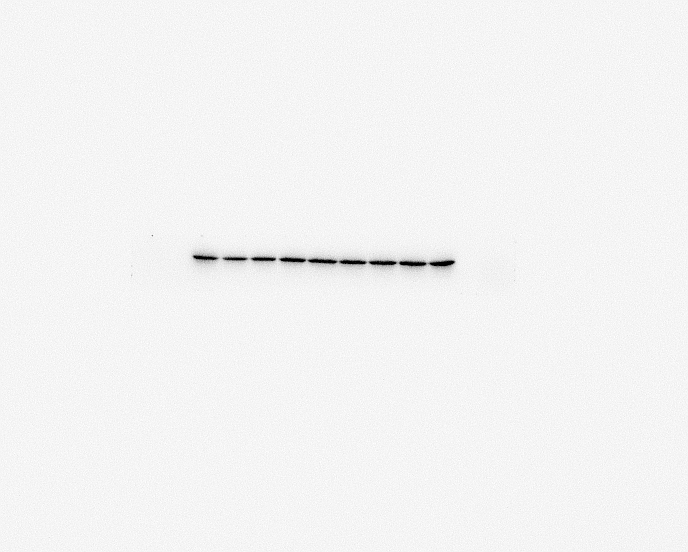

Supplement: Supplementary file 11 [file DataSheet10.ZIP › Western blot/Figure 6E, F/1 (presented in manuscript)/claudin5, occludin/1-actin.tif]

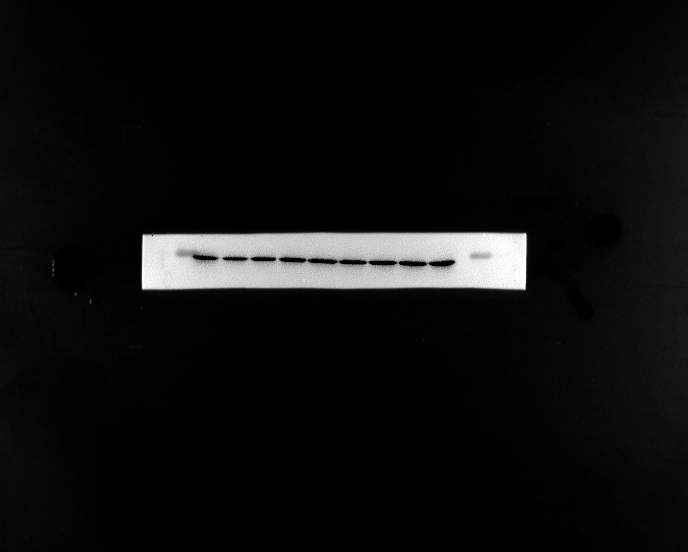

Supplement: Supplementary file 11 [file DataSheet10.ZIP › Western blot/Figure 6E, F/1 (presented in manuscript)/claudin5, occludin/1-actins.tif]

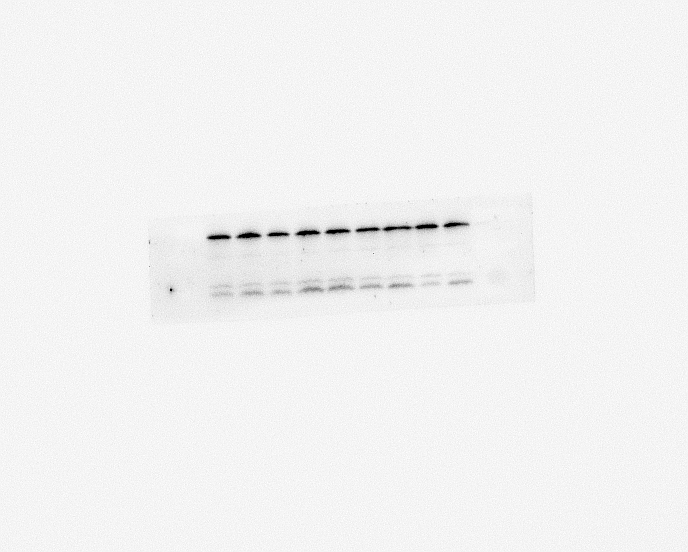

Supplement: Supplementary file 11 [file DataSheet10.ZIP › Western blot/Figure 6E, F/1 (presented in manuscript)/claudin5, occludin/1-claudin.tif]

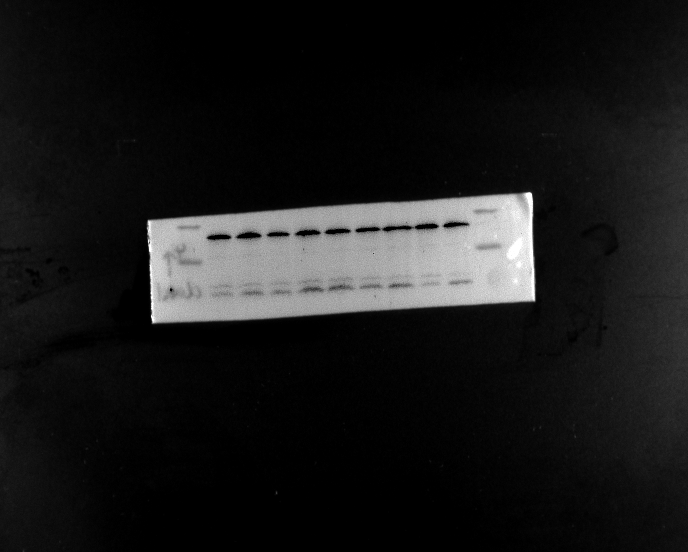

Supplement: Supplementary file 11 [file DataSheet10.ZIP › Western blot/Figure 6E, F/1 (presented in manuscript)/claudin5, occludin/1-claudins.tif]

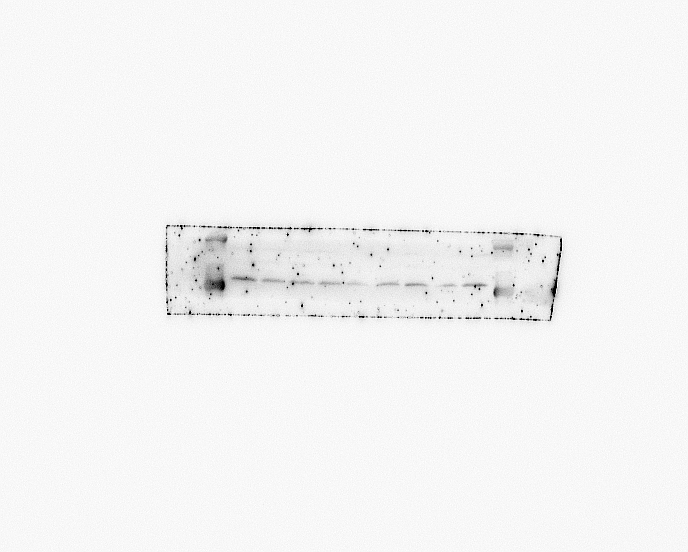

Supplement: Supplementary file 11 [file DataSheet10.ZIP › Western blot/Figure 6E, F/1 (presented in manuscript)/claudin5, occludin/1-occludin.tif]

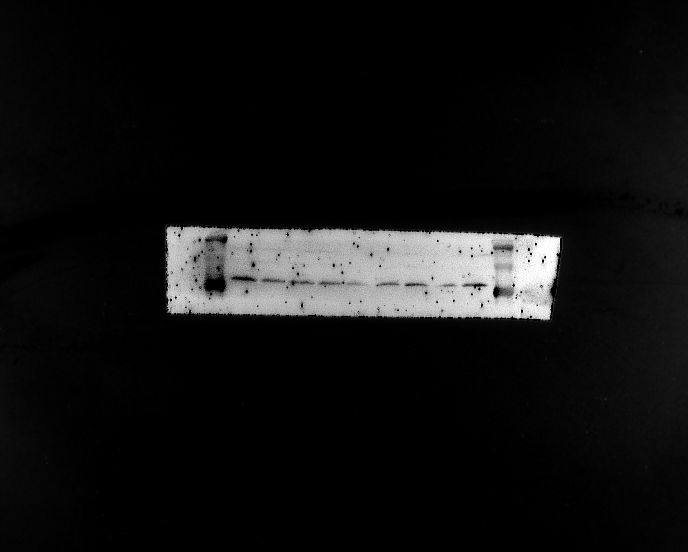

Supplement: Supplementary file 11 [file DataSheet10.ZIP › Western blot/Figure 6E, F/1 (presented in manuscript)/claudin5, occludin/1-occludins.tif]

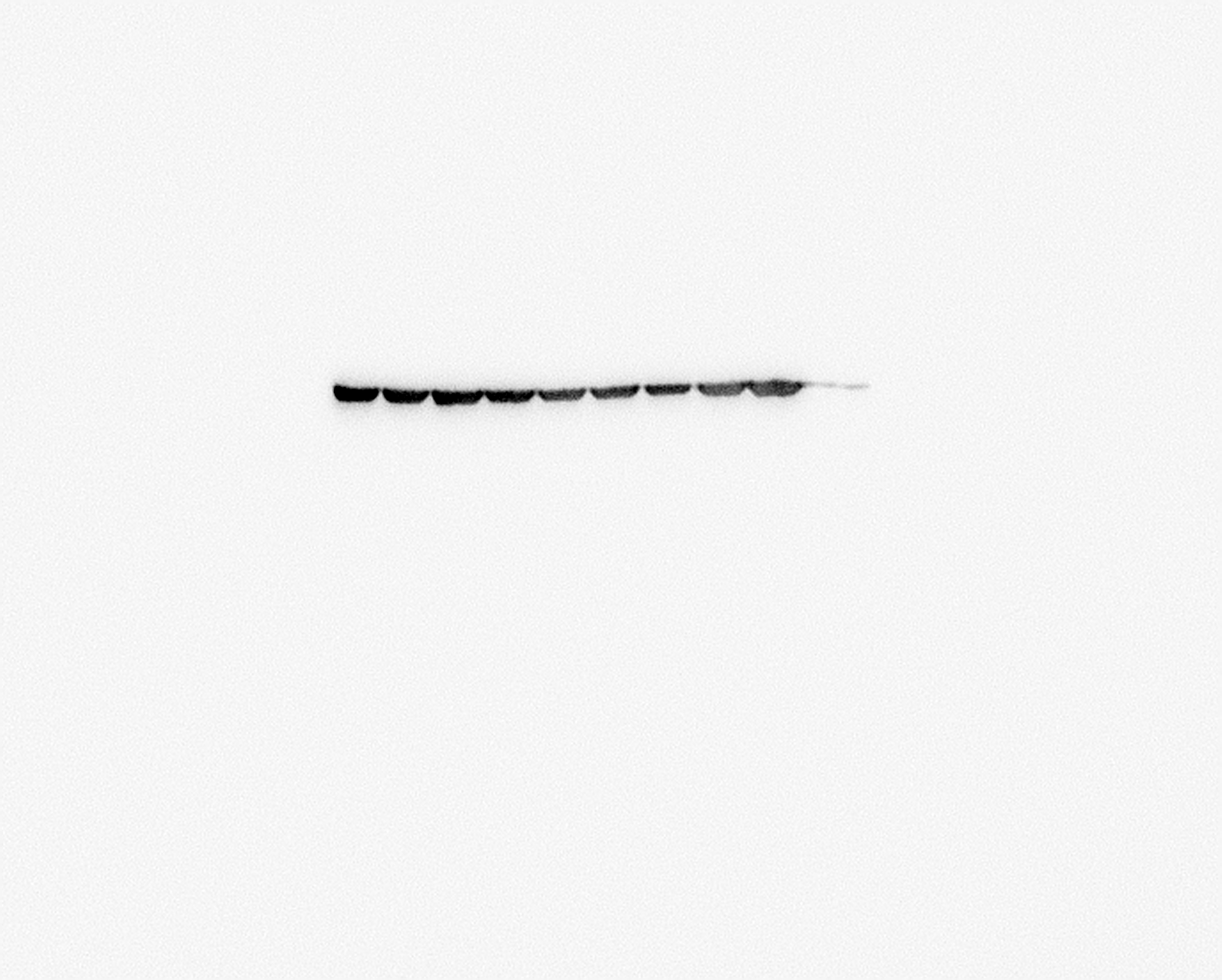

Supplement: Supplementary file 11 [file DataSheet10.ZIP › Western blot/Figure 6E, F/1 (presented in manuscript)/ZO1/1-actin.tif]

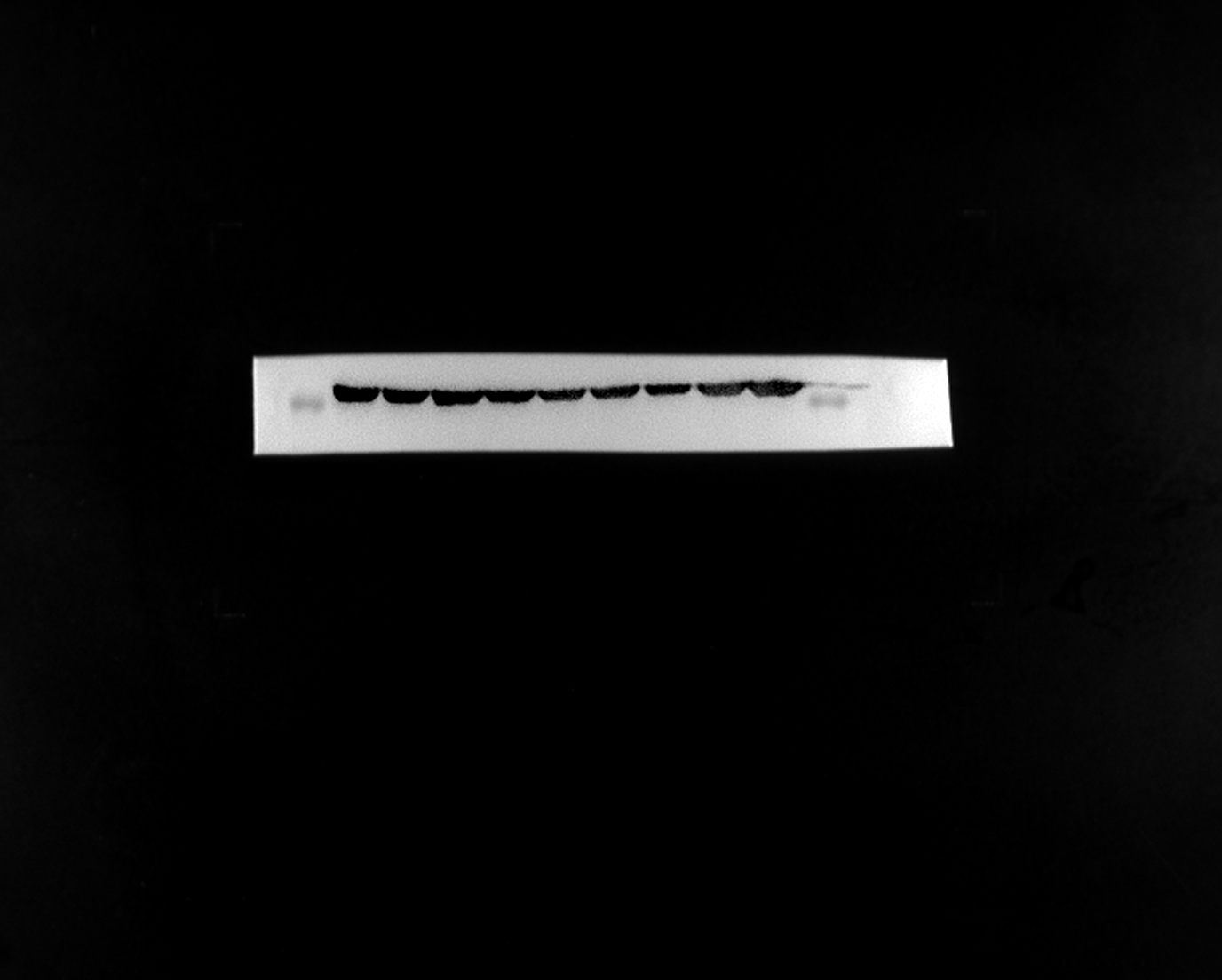

Supplement: Supplementary file 11 [file DataSheet10.ZIP › Western blot/Figure 6E, F/1 (presented in manuscript)/ZO1/1-actins.tif]

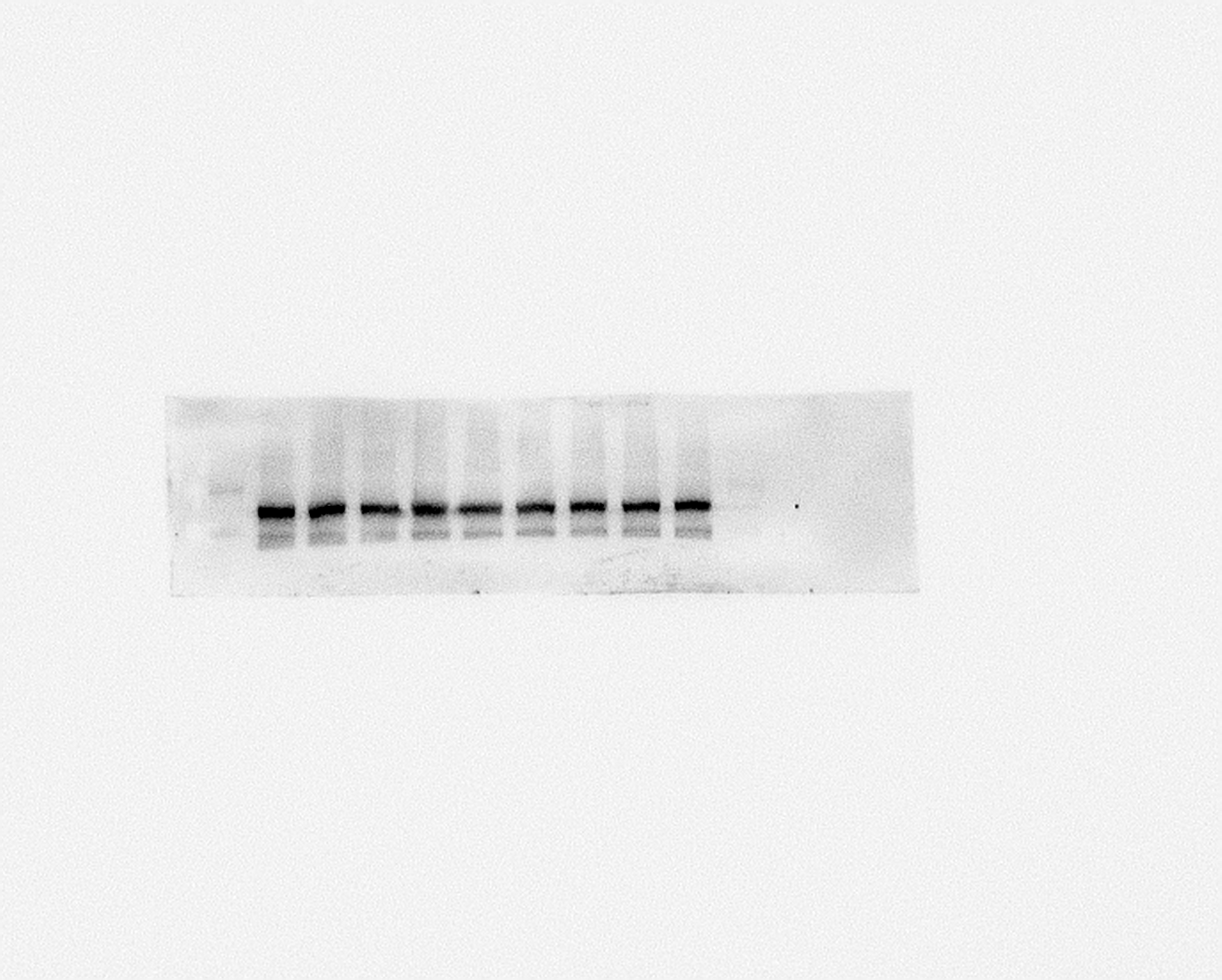

Supplement: Supplementary file 11 [file DataSheet10.ZIP › Western blot/Figure 6E, F/1 (presented in manuscript)/ZO1/1-ZO1.tif]

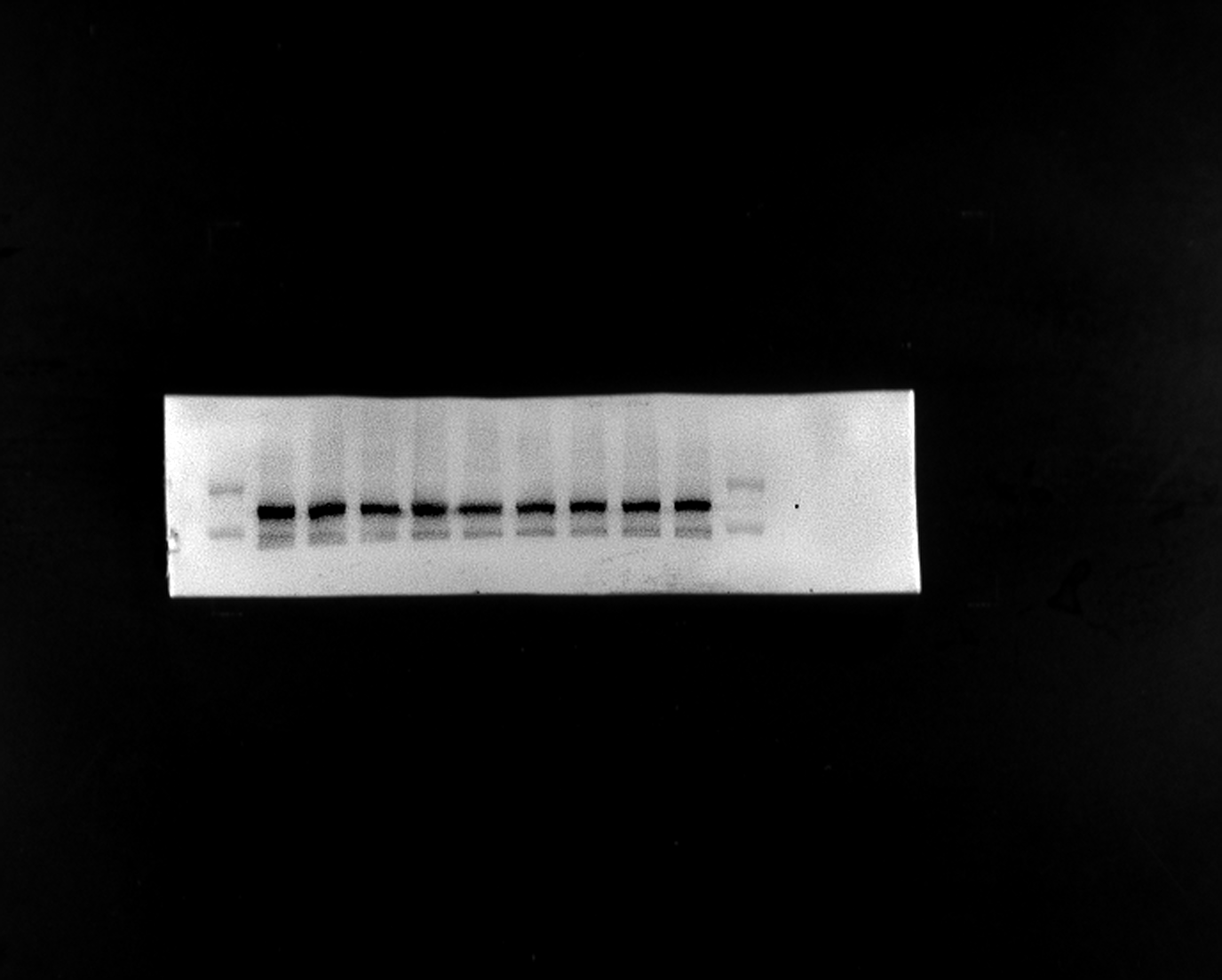

Supplement: Supplementary file 11 [file DataSheet10.ZIP › Western blot/Figure 6E, F/1 (presented in manuscript)/ZO1/1-ZO1s.tif]

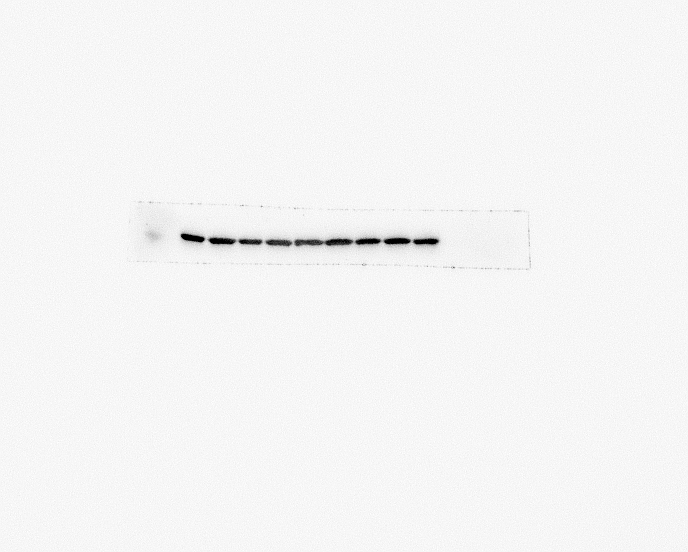

Supplement: Supplementary file 11 [file DataSheet10.ZIP › Western blot/Figure 6E, F/2/claudin5, occludin/2 actin.tif]

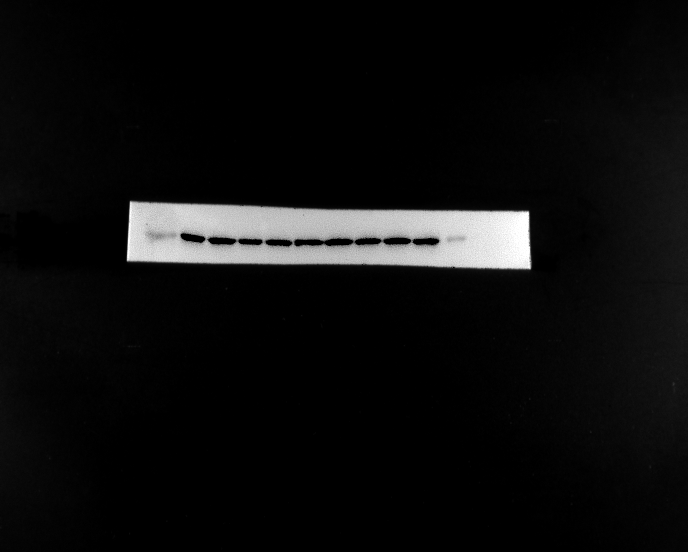

Supplement: Supplementary file 11 [file DataSheet10.ZIP › Western blot/Figure 6E, F/2/claudin5, occludin/2 actins.tif]

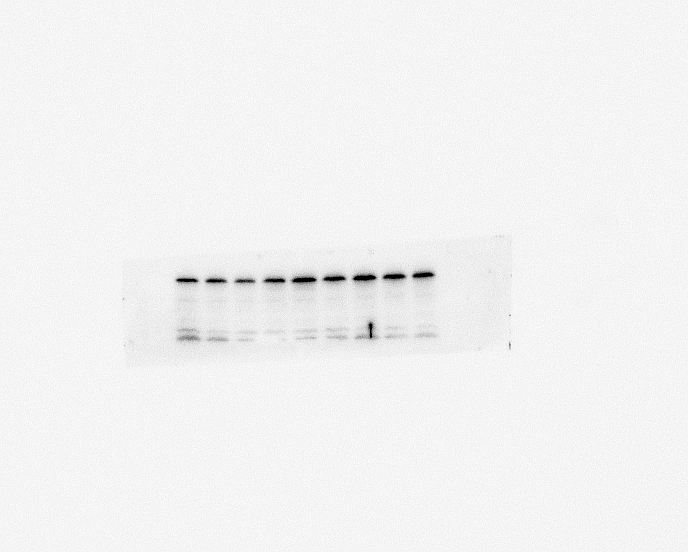

Supplement: Supplementary file 11 [file DataSheet10.ZIP › Western blot/Figure 6E, F/2/claudin5, occludin/2 claudin.tif]

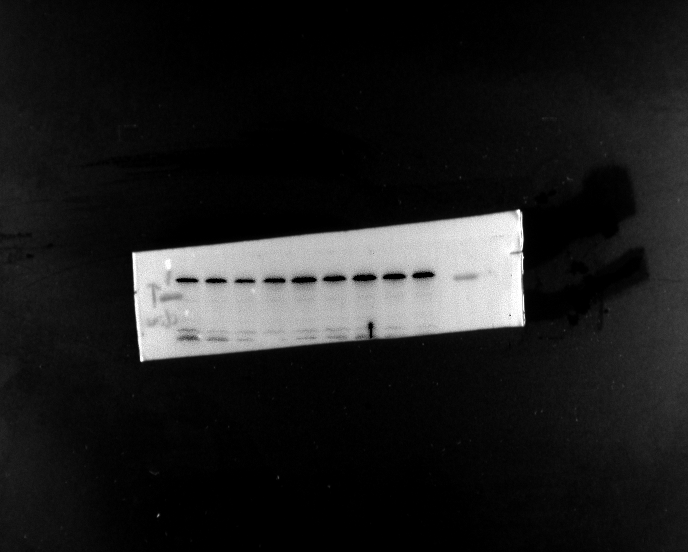

Supplement: Supplementary file 11 [file DataSheet10.ZIP › Western blot/Figure 6E, F/2/claudin5, occludin/2 claudins.tif]

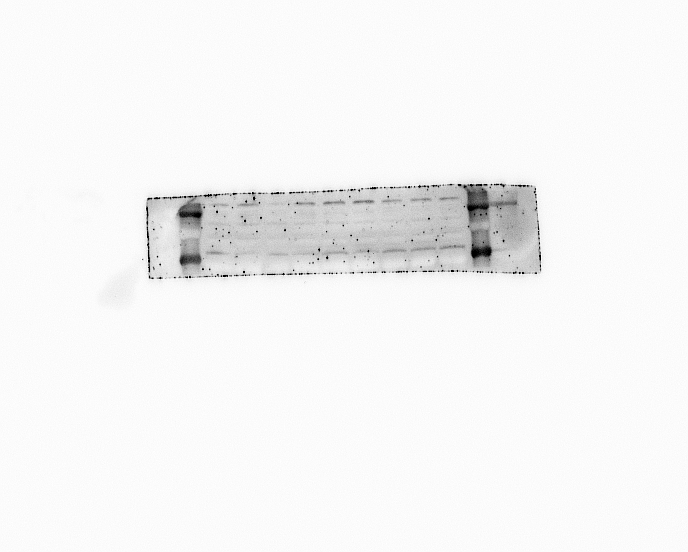

Supplement: Supplementary file 11 [file DataSheet10.ZIP › Western blot/Figure 6E, F/2/claudin5, occludin/2 occludin.tif]

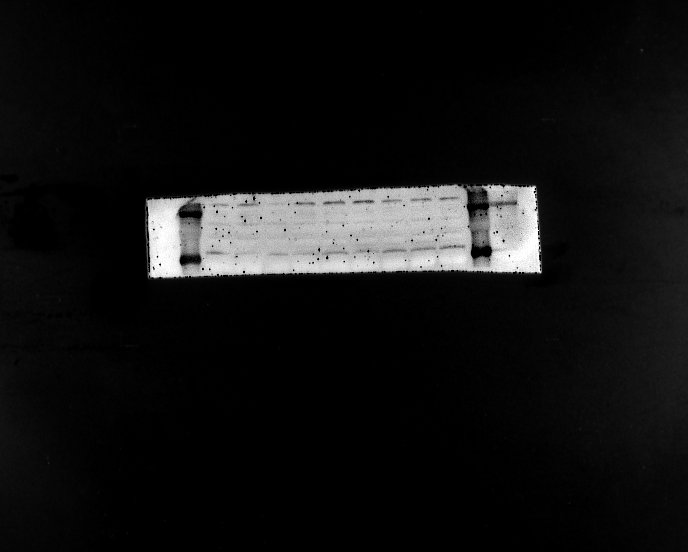

Supplement: Supplementary file 11 [file DataSheet10.ZIP › Western blot/Figure 6E, F/2/claudin5, occludin/2 occludins.tif]

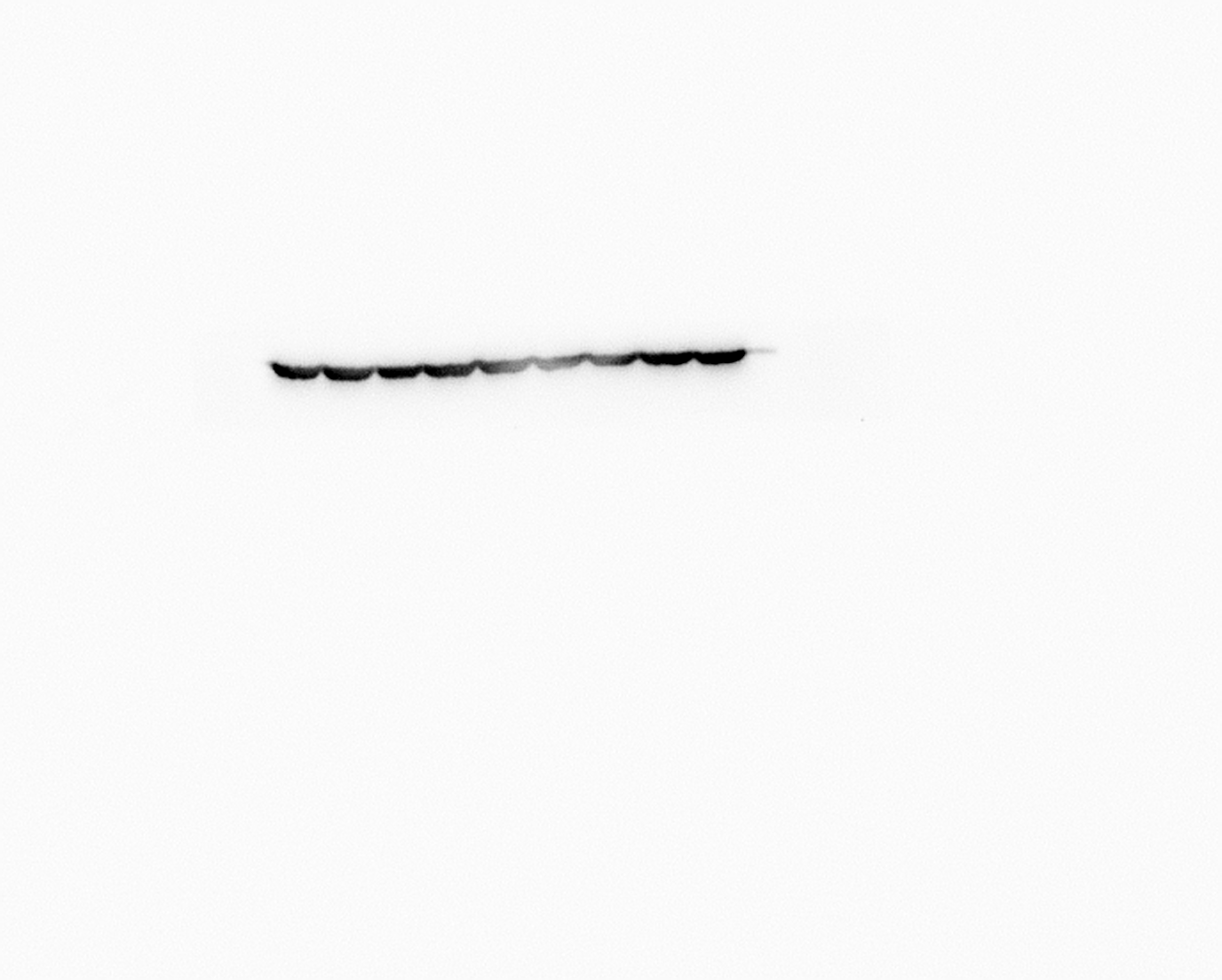

Supplement: Supplementary file 11 [file DataSheet10.ZIP › Western blot/Figure 6E, F/2/ZO1/2-actin.tif]

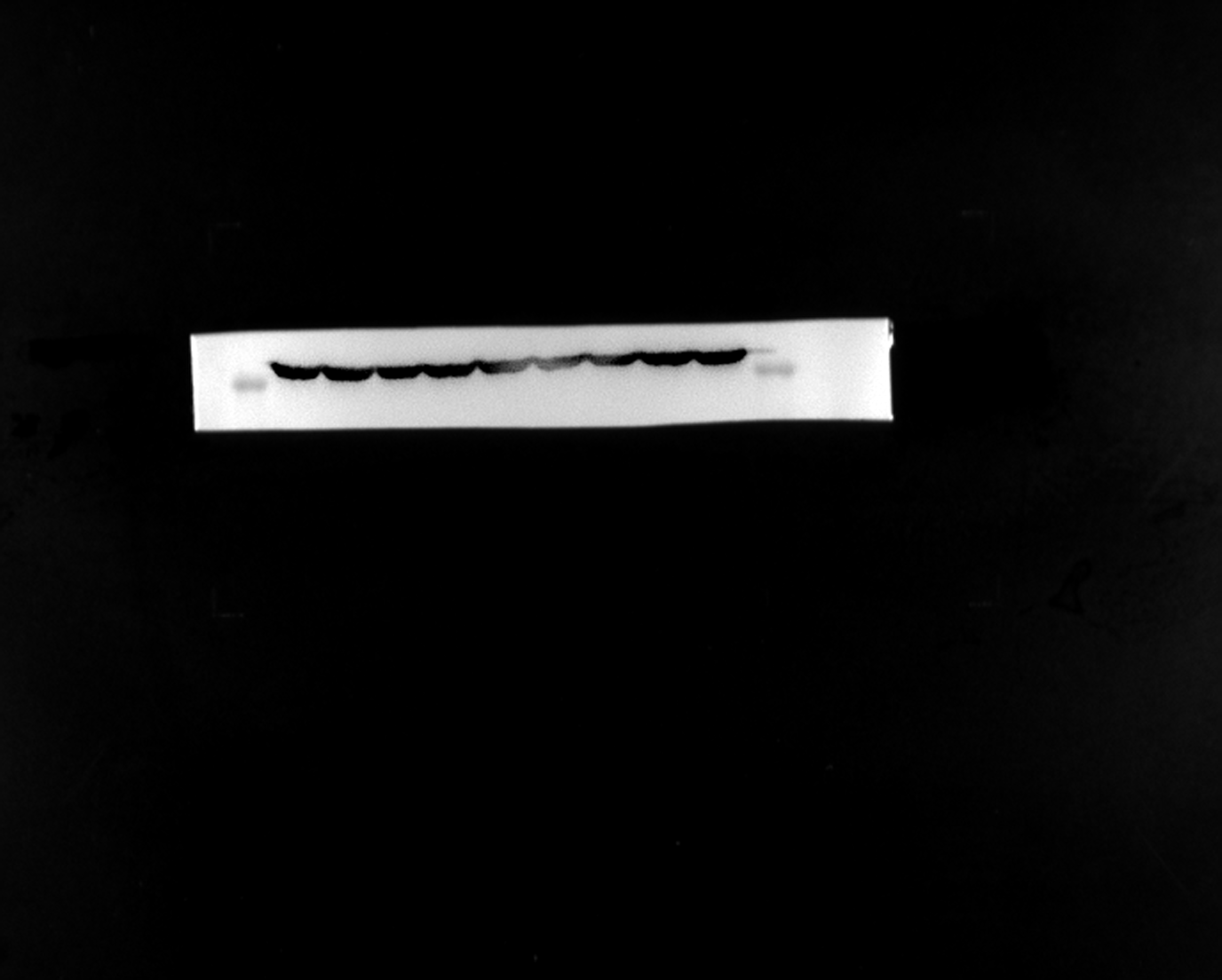

Supplement: Supplementary file 11 [file DataSheet10.ZIP › Western blot/Figure 6E, F/2/ZO1/2-actins.tif]

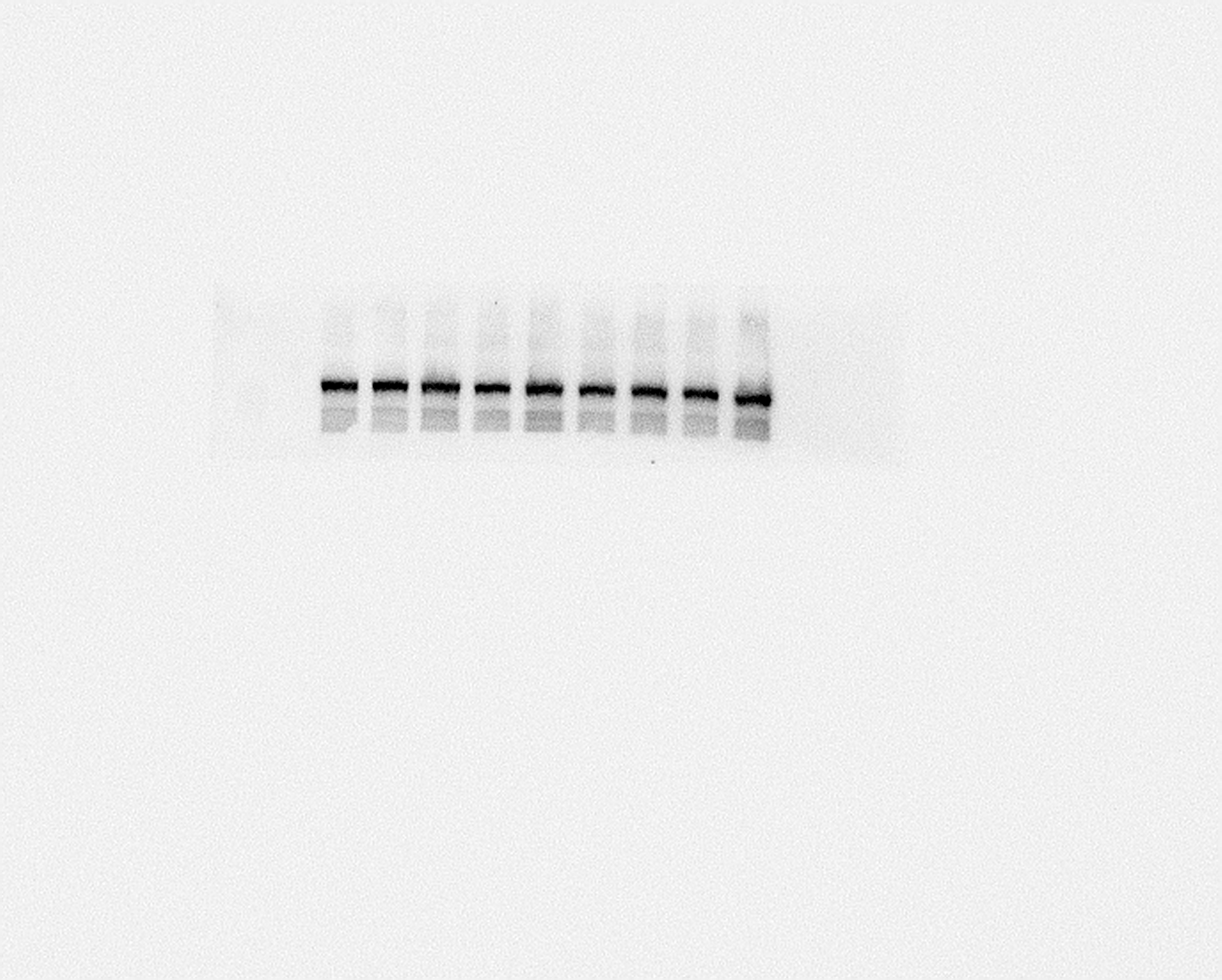

Supplement: Supplementary file 11 [file DataSheet10.ZIP › Western blot/Figure 6E, F/2/ZO1/2-ZO1.tif]

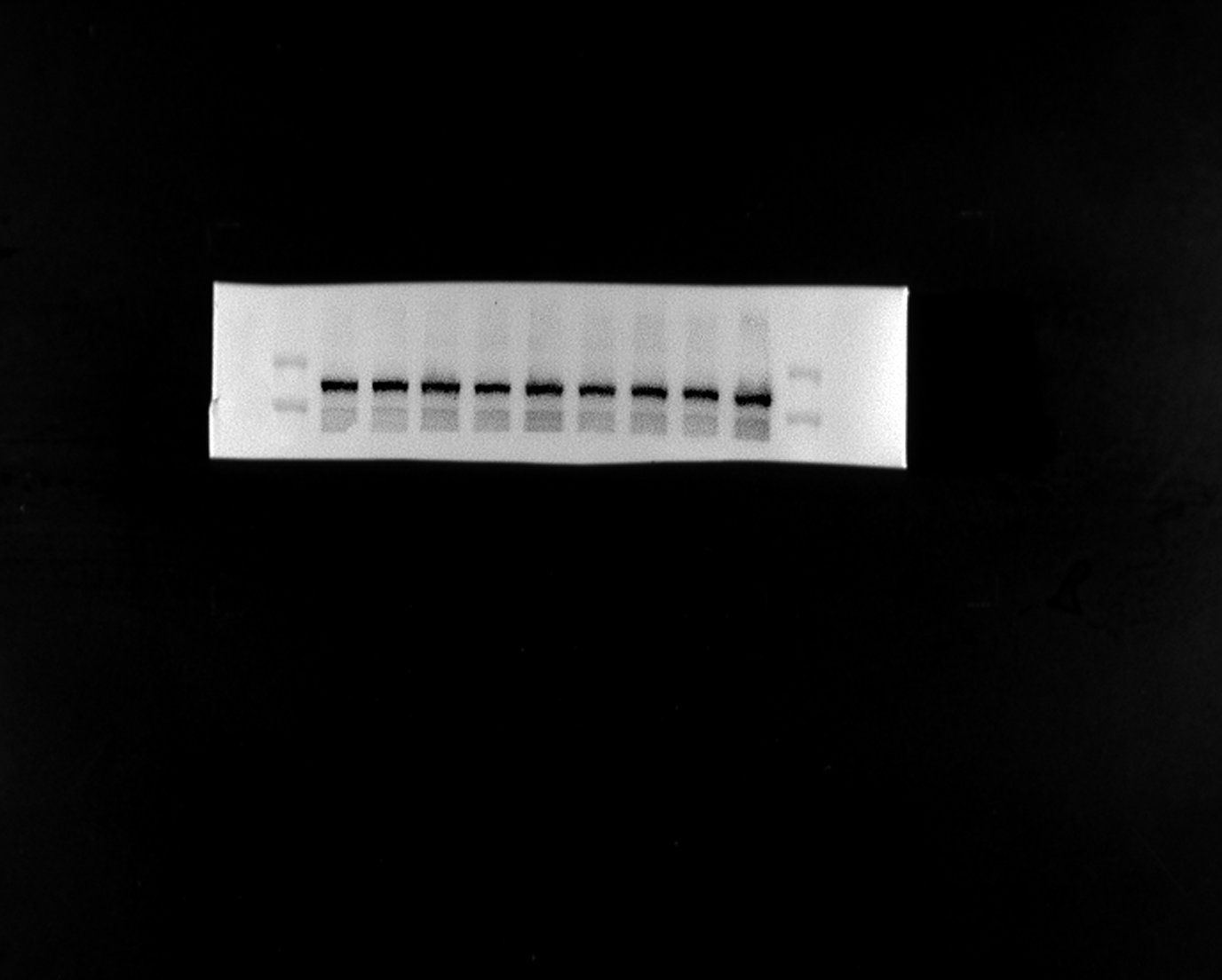

Supplement: Supplementary file 11 [file DataSheet10.ZIP › Western blot/Figure 6E, F/2/ZO1/2-ZO1s.tif]

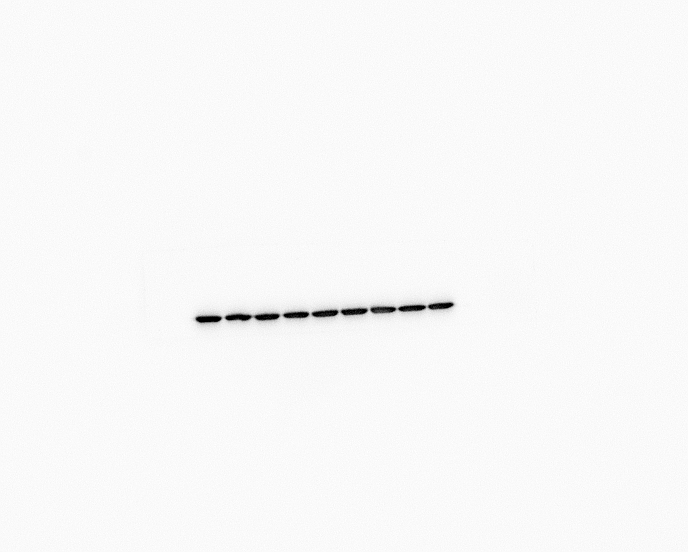

Supplement: Supplementary file 11 [file DataSheet10.ZIP › Western blot/Figure 6I, J/1 (presented in manuscript)/CDK2/1-2-actin.tif]

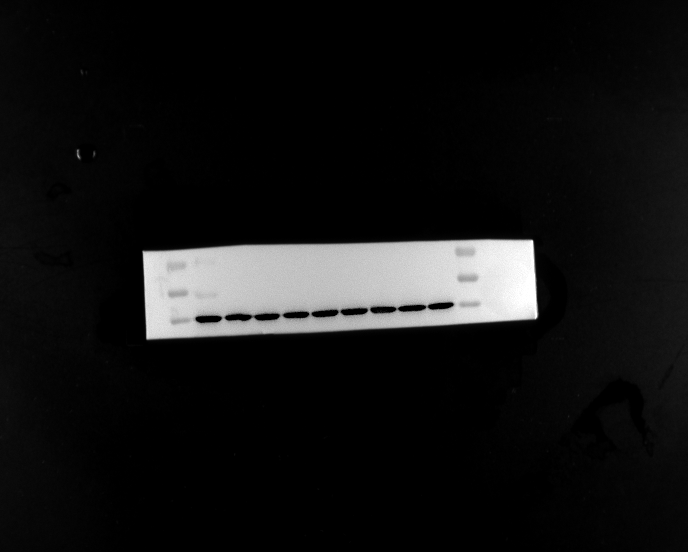

Supplement: Supplementary file 11 [file DataSheet10.ZIP › Western blot/Figure 6I, J/1 (presented in manuscript)/CDK2/1-2-actins.tif]

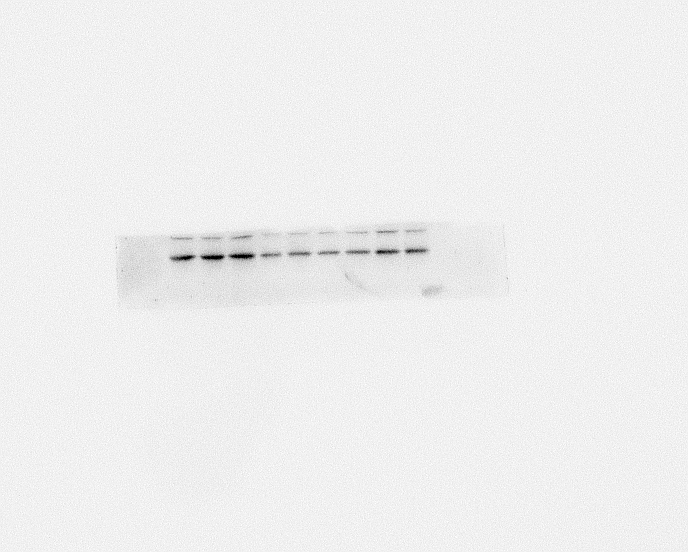

Supplement: Supplementary file 11 [file DataSheet10.ZIP › Western blot/Figure 6I, J/1 (presented in manuscript)/CDK2/1-CDK2.tif]

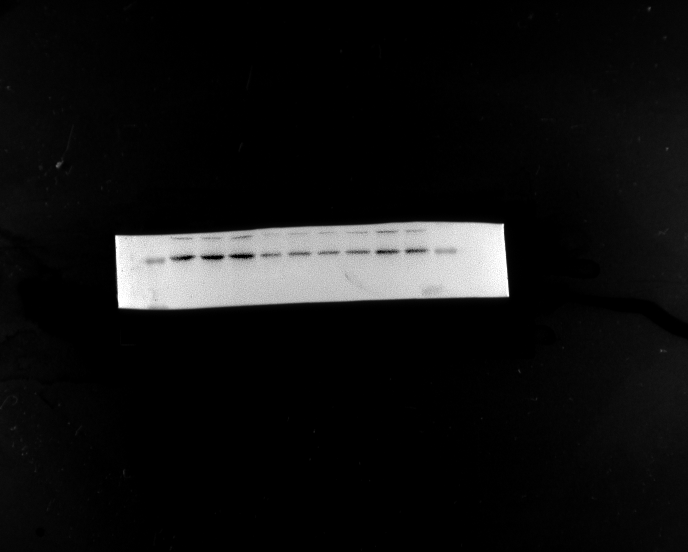

Supplement: Supplementary file 11 [file DataSheet10.ZIP › Western blot/Figure 6I, J/1 (presented in manuscript)/CDK2/1-CDK2s.tif]

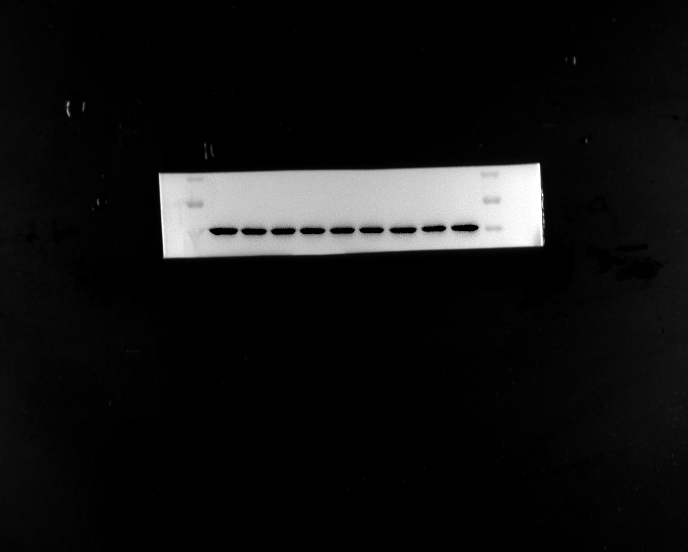

Supplement: Supplementary file 11 [file DataSheet10.ZIP › Western blot/Figure 6I, J/1 (presented in manuscript)/cyclin A/1-1-actin-s.tif]

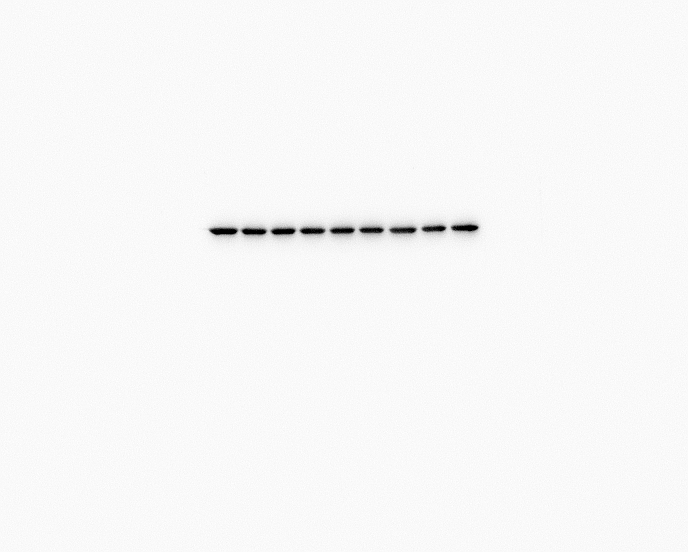

Supplement: Supplementary file 11 [file DataSheet10.ZIP › Western blot/Figure 6I, J/1 (presented in manuscript)/cyclin A/1-1-actin.tif]

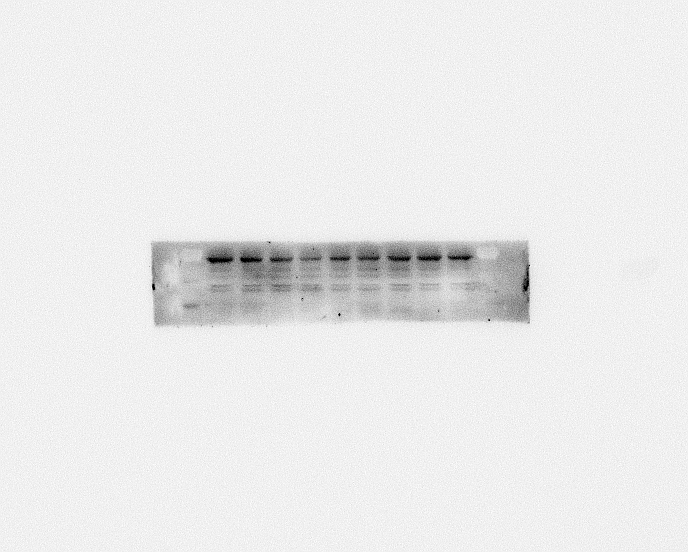

Supplement: Supplementary file 11 [file DataSheet10.ZIP › Western blot/Figure 6I, J/1 (presented in manuscript)/cyclin A/1-cyclinA.tif]

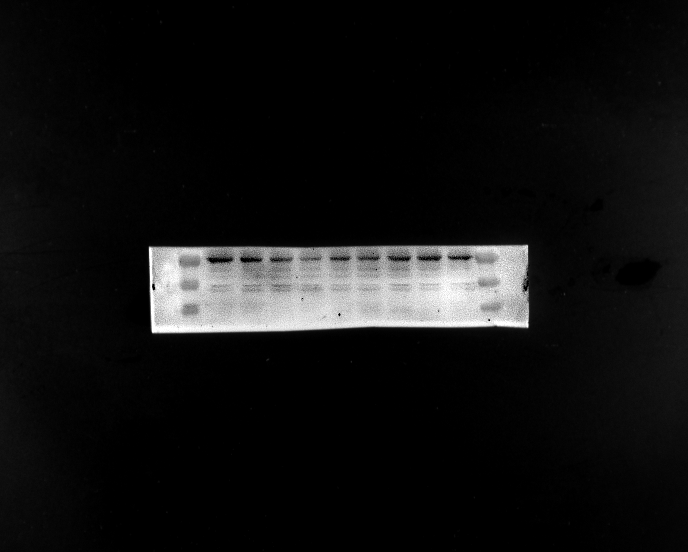

Supplement: Supplementary file 11 [file DataSheet10.ZIP › Western blot/Figure 6I, J/1 (presented in manuscript)/cyclin A/1-cyclinAs.tif]

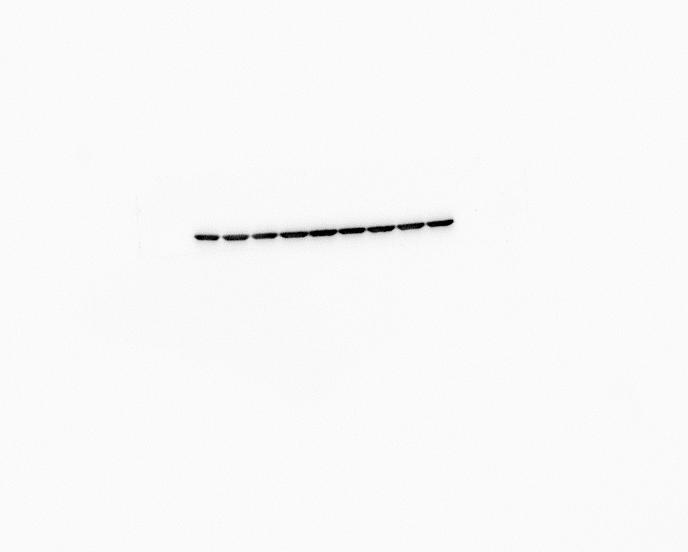

Supplement: Supplementary file 11 [file DataSheet10.ZIP › Western blot/Figure 6I, J/1 (presented in manuscript)/cyclin D, CDK4/1-3-actin.tif]

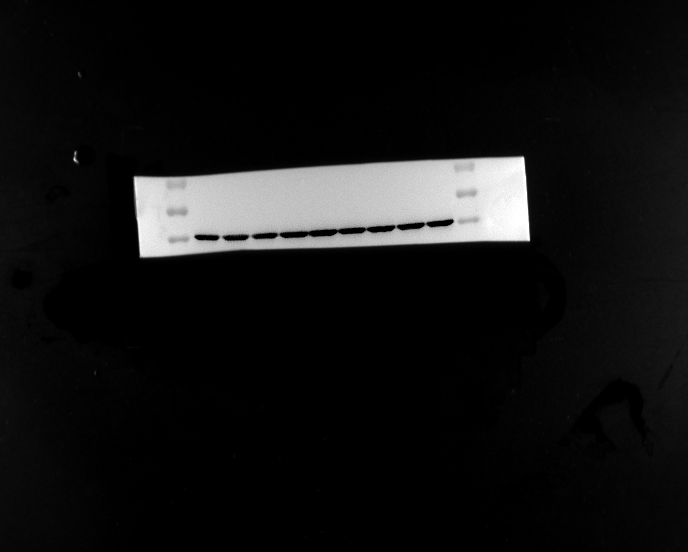

Supplement: Supplementary file 11 [file DataSheet10.ZIP › Western blot/Figure 6I, J/1 (presented in manuscript)/cyclin D, CDK4/1-3-actins.tif]

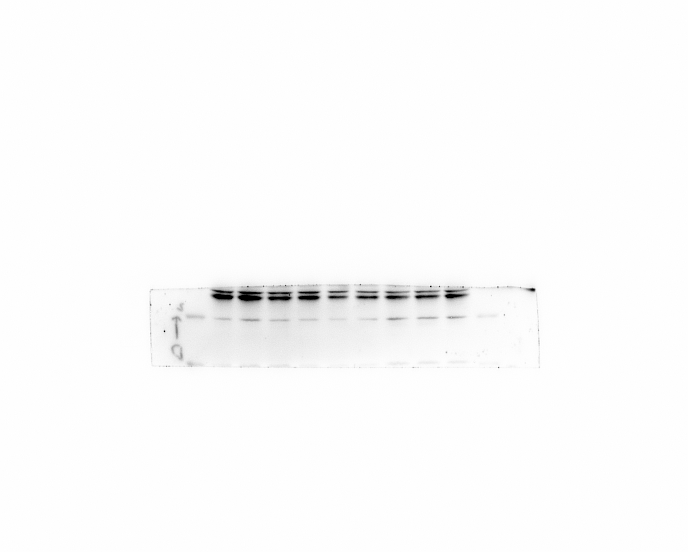

Supplement: Supplementary file 11 [file DataSheet10.ZIP › Western blot/Figure 6I, J/1 (presented in manuscript)/cyclin D, CDK4/1-CDK4.tif]

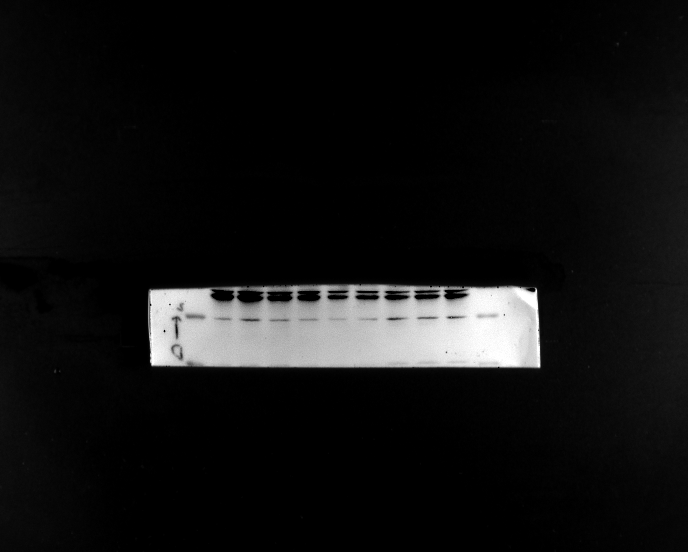

Supplement: Supplementary file 11 [file DataSheet10.ZIP › Western blot/Figure 6I, J/1 (presented in manuscript)/cyclin D, CDK4/1-CDK4s.tif]

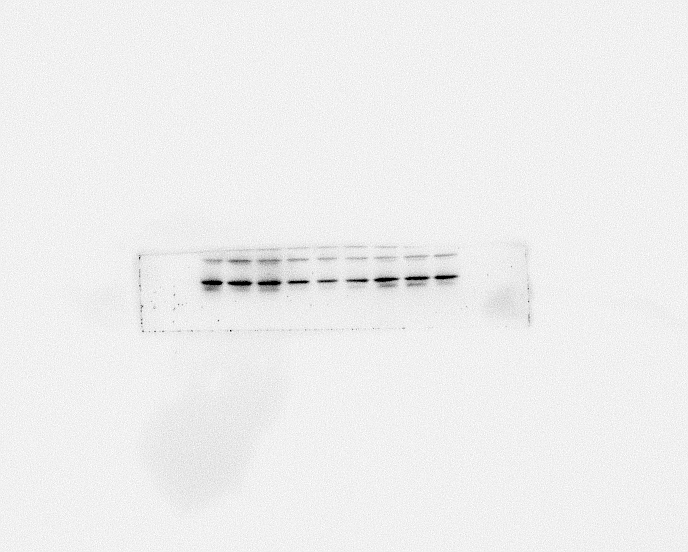

Supplement: Supplementary file 11 [file DataSheet10.ZIP › Western blot/Figure 6I, J/1 (presented in manuscript)/cyclin D, CDK4/1-cyclinD.tif]

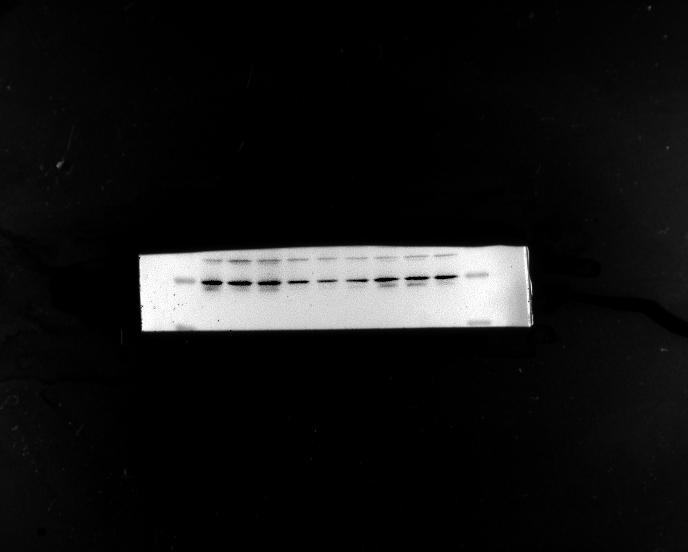

Supplement: Supplementary file 11 [file DataSheet10.ZIP › Western blot/Figure 6I, J/1 (presented in manuscript)/cyclin D, CDK4/1-cyclinDs.tif]

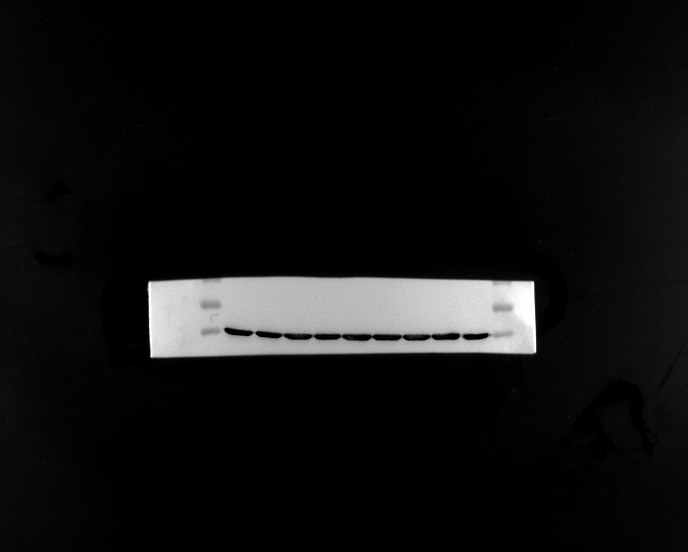

Supplement: Supplementary file 11 [file DataSheet10.ZIP › Western blot/Figure 6I, J/2/CDK2/3-actin-s.tif]

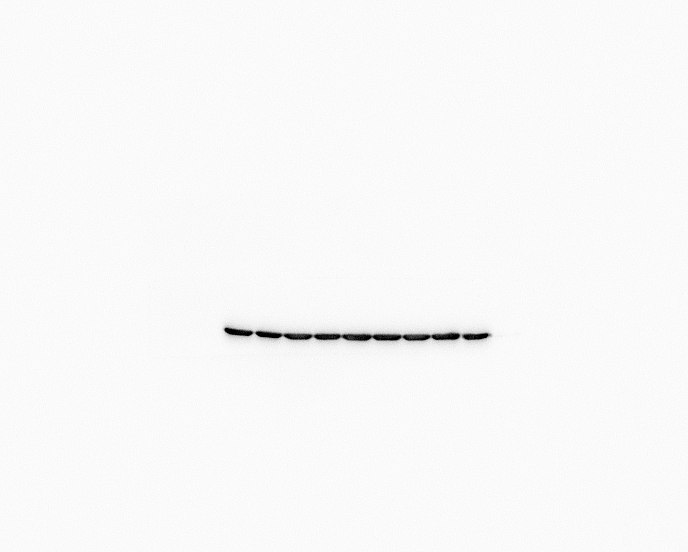

Supplement: Supplementary file 11 [file DataSheet10.ZIP › Western blot/Figure 6I, J/2/CDK2/3-actin.tif]

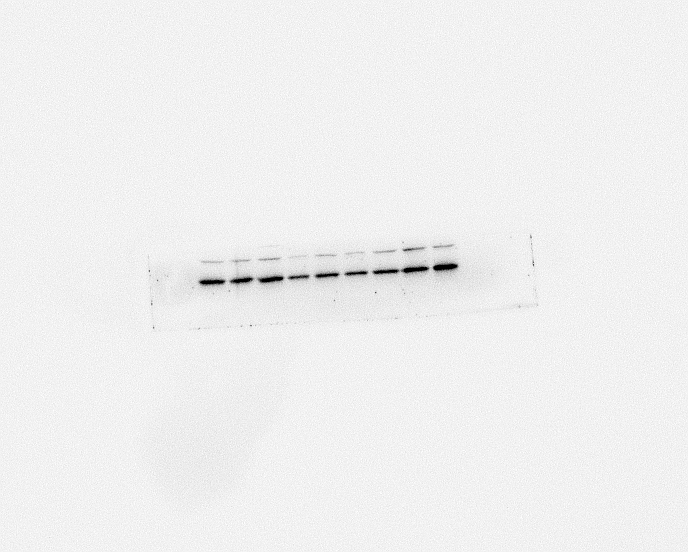

Supplement: Supplementary file 11 [file DataSheet10.ZIP › Western blot/Figure 6I, J/2/CDK2/3-CDK2.tif]

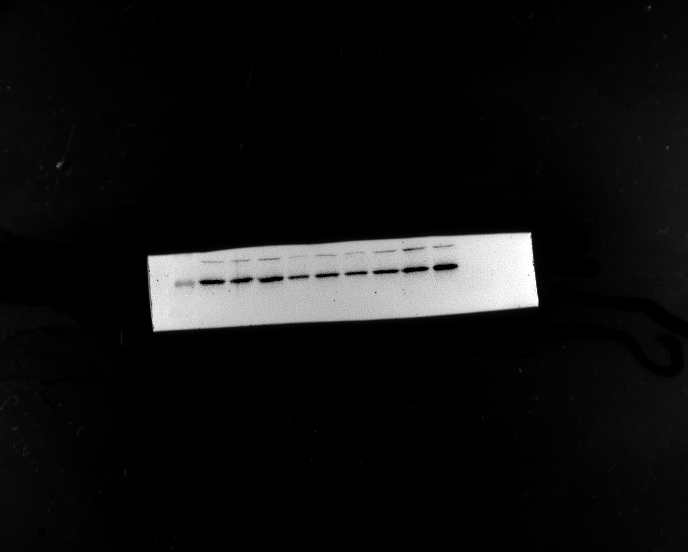

Supplement: Supplementary file 11 [file DataSheet10.ZIP › Western blot/Figure 6I, J/2/CDK2/3-CDK2s.tif]

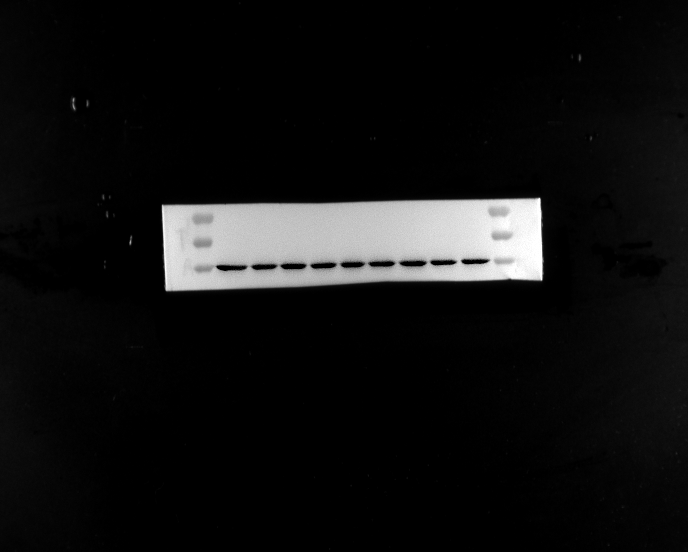

Supplement: Supplementary file 11 [file DataSheet10.ZIP › Western blot/Figure 6I, J/2/cyclin A/2-1-actin-s.tif]

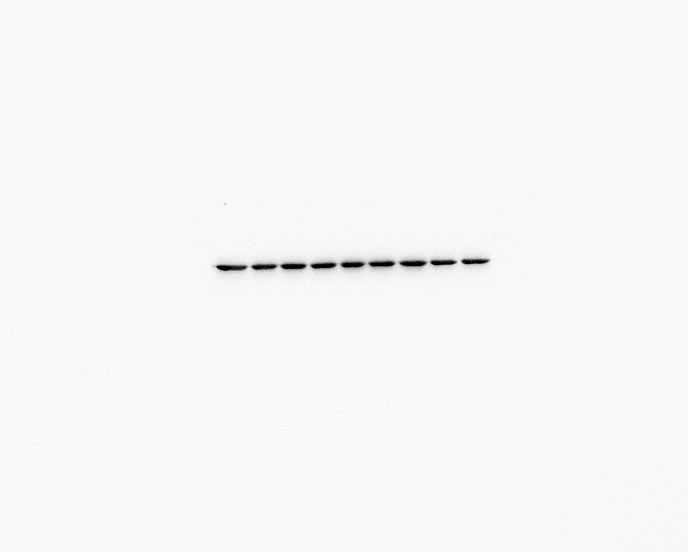

Supplement: Supplementary file 11 [file DataSheet10.ZIP › Western blot/Figure 6I, J/2/cyclin A/2-1-actin.tif]

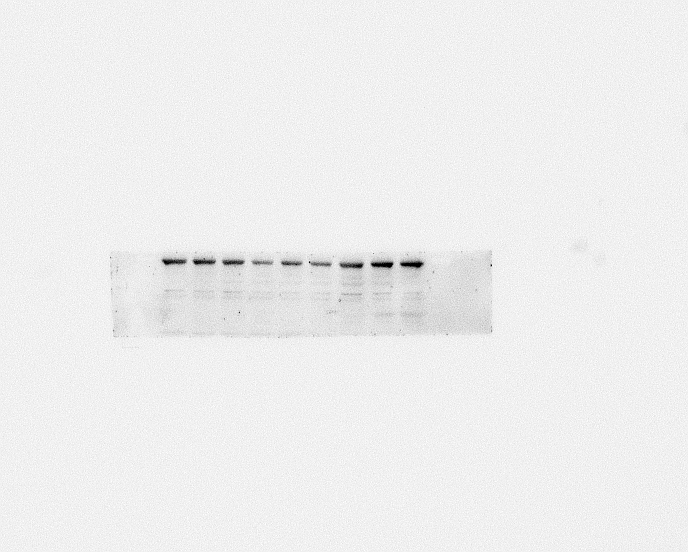

Supplement: Supplementary file 11 [file DataSheet10.ZIP › Western blot/Figure 6I, J/2/cyclin A/2-cyclinA.tif]

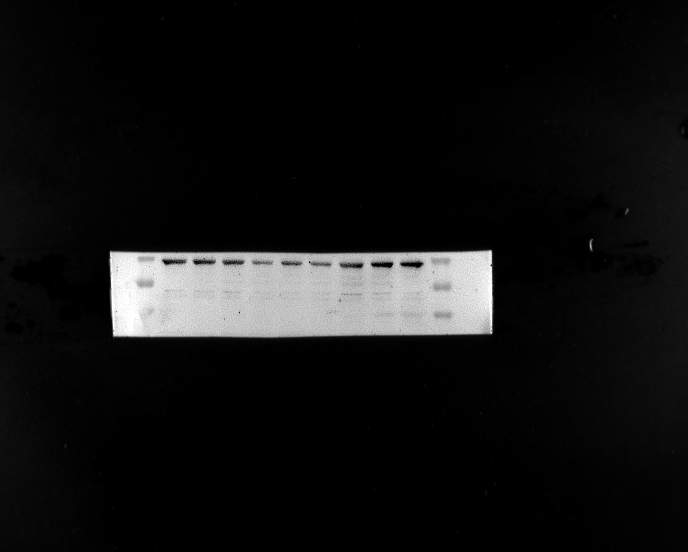

Supplement: Supplementary file 11 [file DataSheet10.ZIP › Western blot/Figure 6I, J/2/cyclin A/2-cyclinAs.tif]

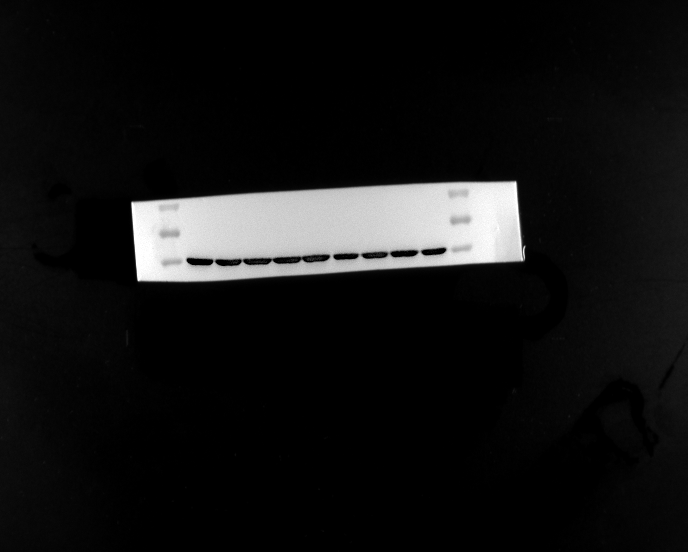

Supplement: Supplementary file 11 [file DataSheet10.ZIP › Western blot/Figure 6I, J/2/cyclin D, CDK4/2-3-actin-s.tif]

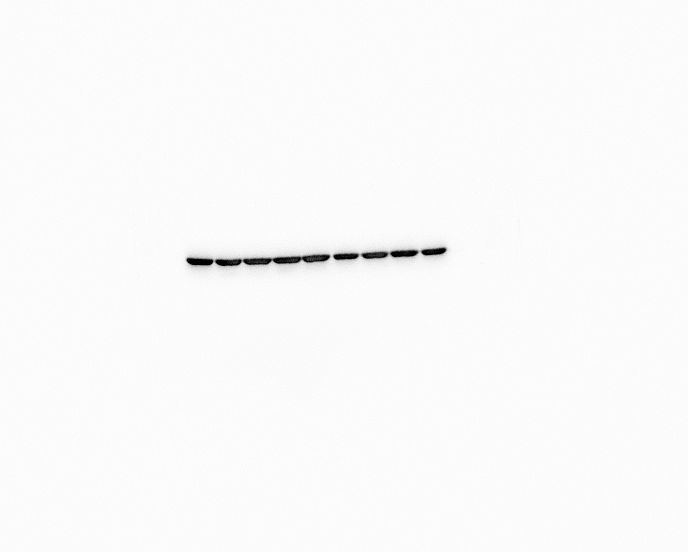

Supplement: Supplementary file 11 [file DataSheet10.ZIP › Western blot/Figure 6I, J/2/cyclin D, CDK4/2-3-actin.tif]

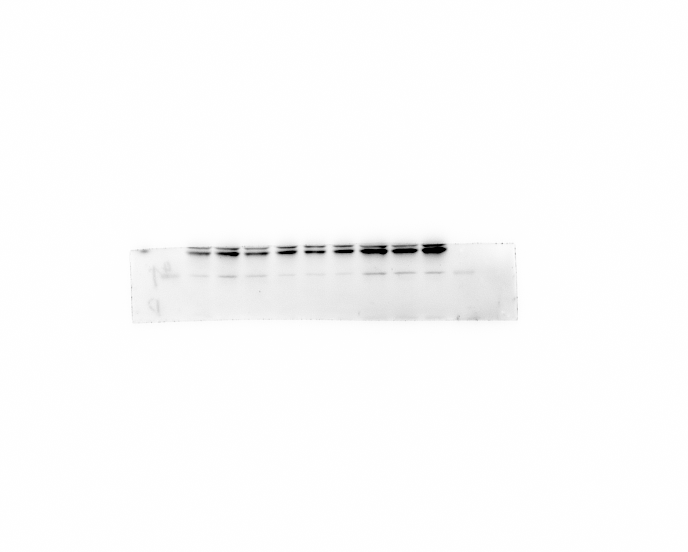

Supplement: Supplementary file 11 [file DataSheet10.ZIP › Western blot/Figure 6I, J/2/cyclin D, CDK4/2-CDK4.tif]

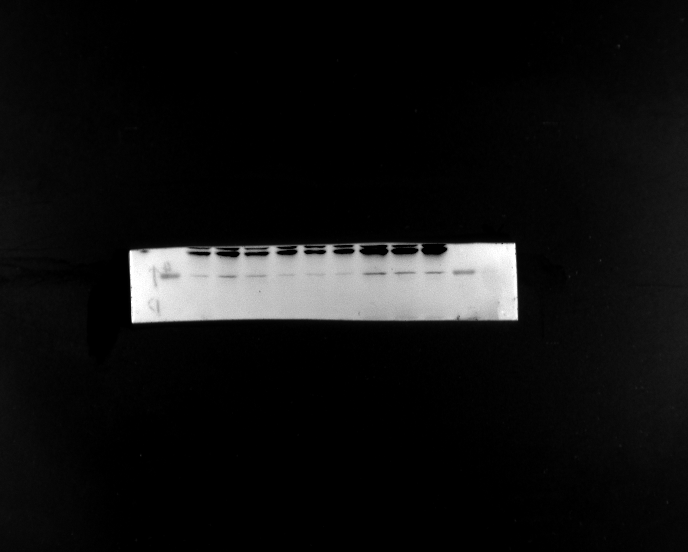

Supplement: Supplementary file 11 [file DataSheet10.ZIP › Western blot/Figure 6I, J/2/cyclin D, CDK4/2-CDK4s.tif]

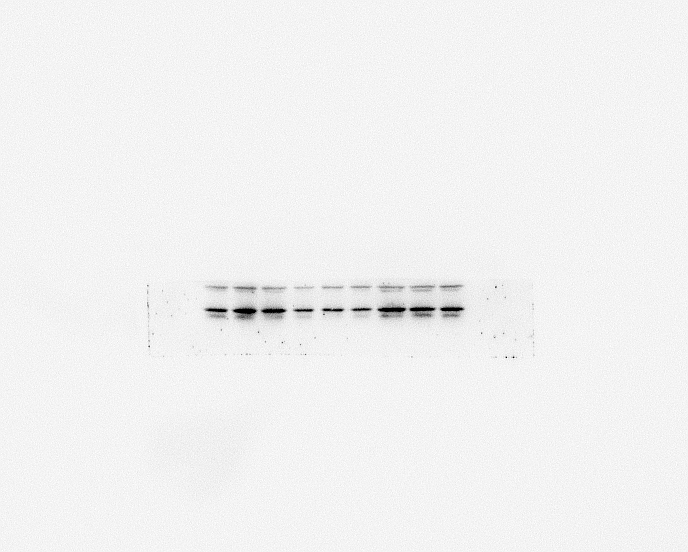

Supplement: Supplementary file 11 [file DataSheet10.ZIP › Western blot/Figure 6I, J/2/cyclin D, CDK4/2-cyclinD.tif]

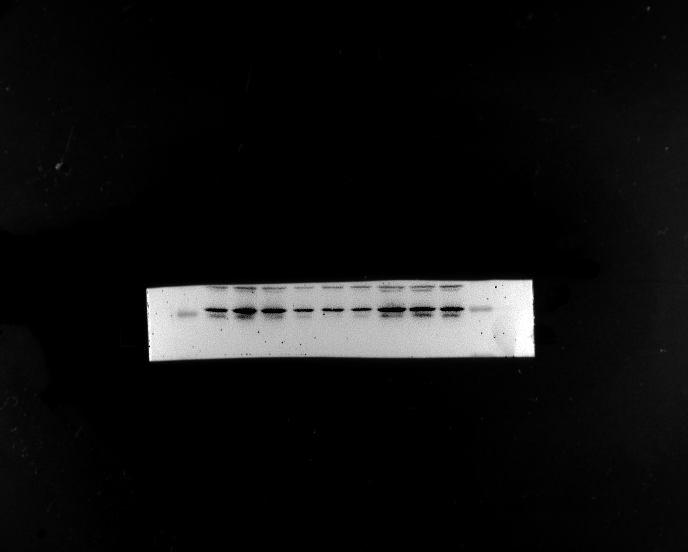

Supplement: Supplementary file 11 [file DataSheet10.ZIP › Western blot/Figure 6I, J/2/cyclin D, CDK4/2-cyclinDs.tif]

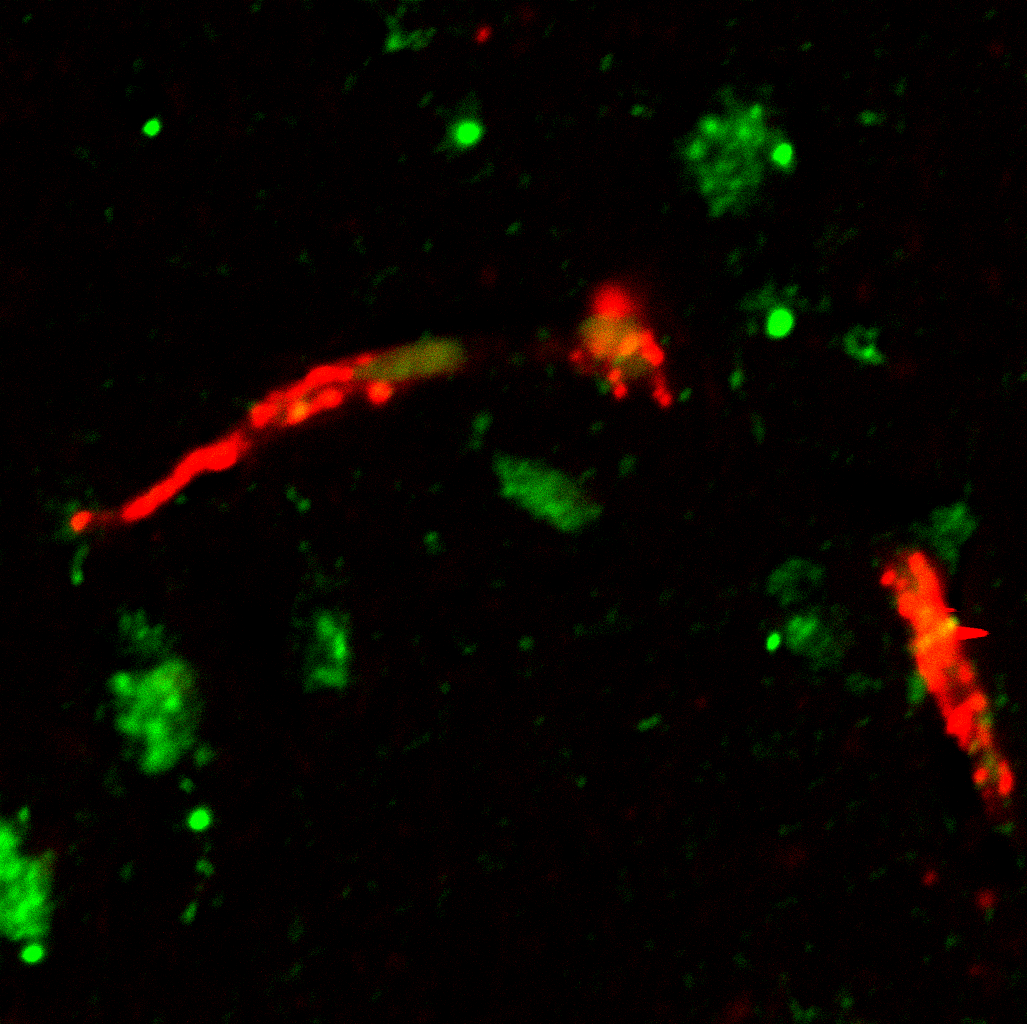

Supplement: Supplementary file 12 [file DataSheet6.ZIP › Immunofluorescence (Figure 6G, part1)/1/1/1s_c1+2.tif]

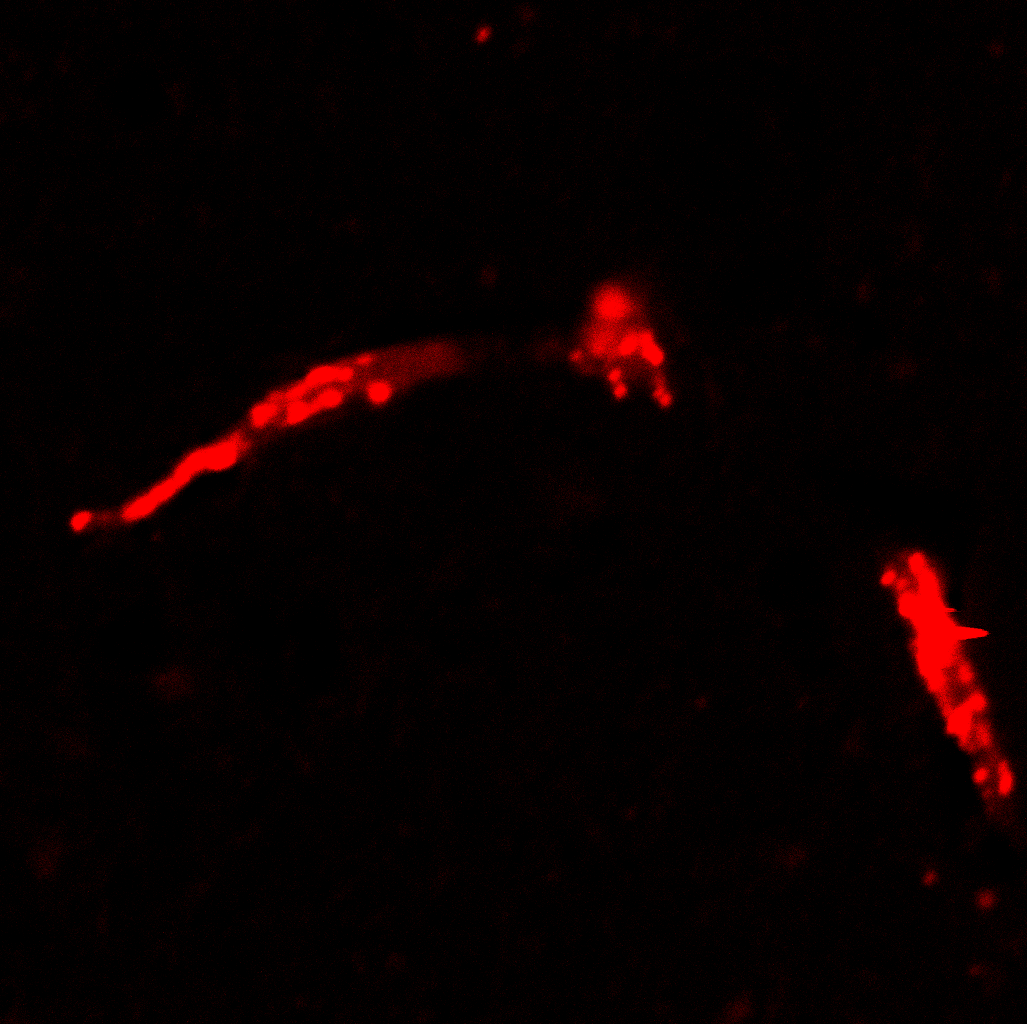

Supplement: Supplementary file 12 [file DataSheet6.ZIP › Immunofluorescence (Figure 6G, part1)/1/1/1s_c1.tif]

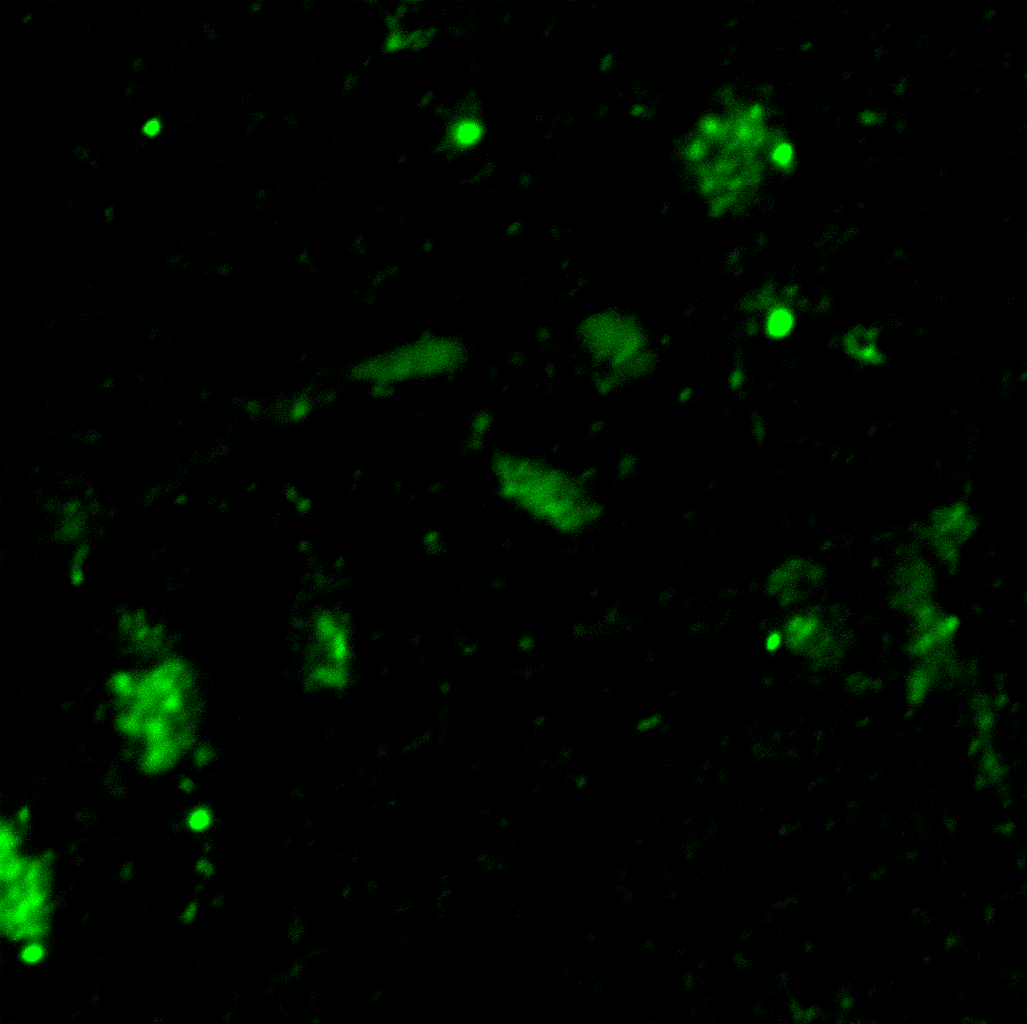

Supplement: Supplementary file 12 [file DataSheet6.ZIP › Immunofluorescence (Figure 6G, part1)/1/1/1s_c2.tif]

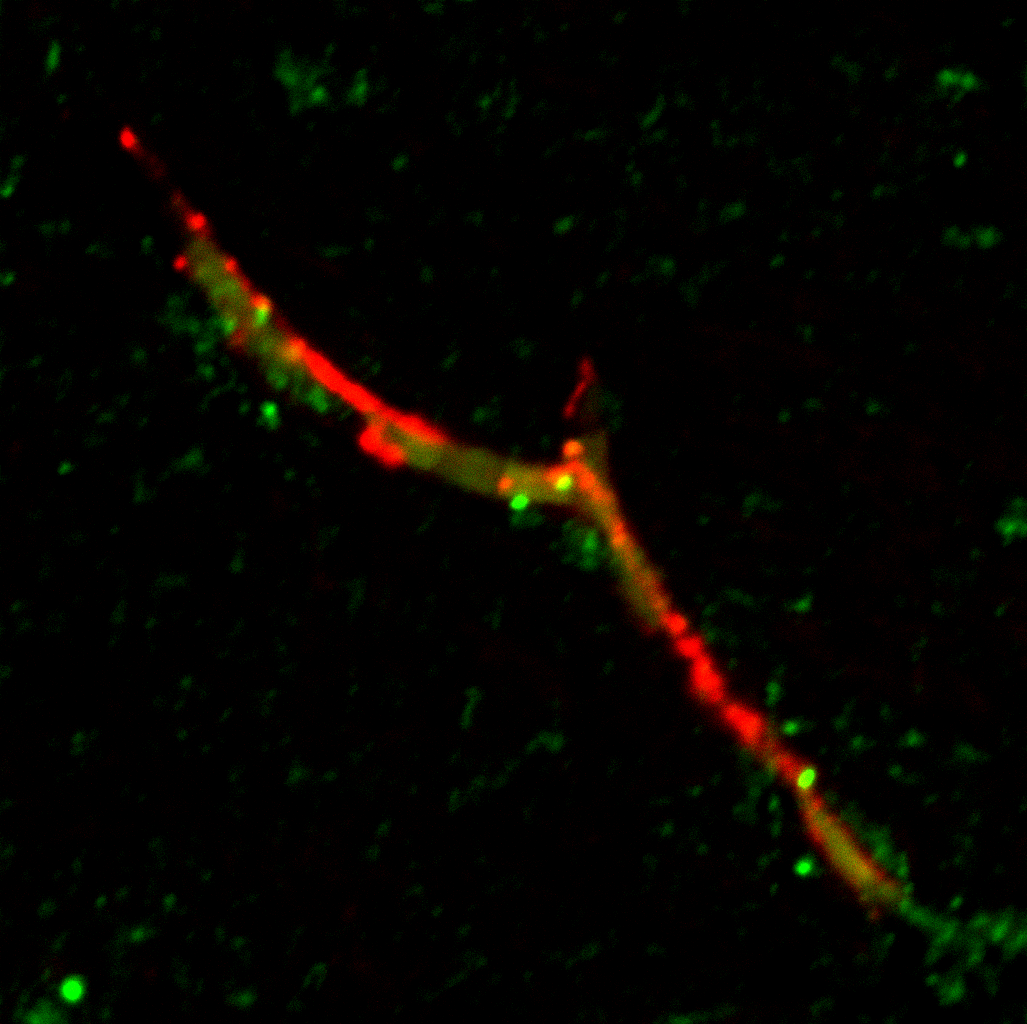

Supplement: Supplementary file 12 [file DataSheet6.ZIP › Immunofluorescence (Figure 6G, part1)/1/2/3s_c1+2.tif]

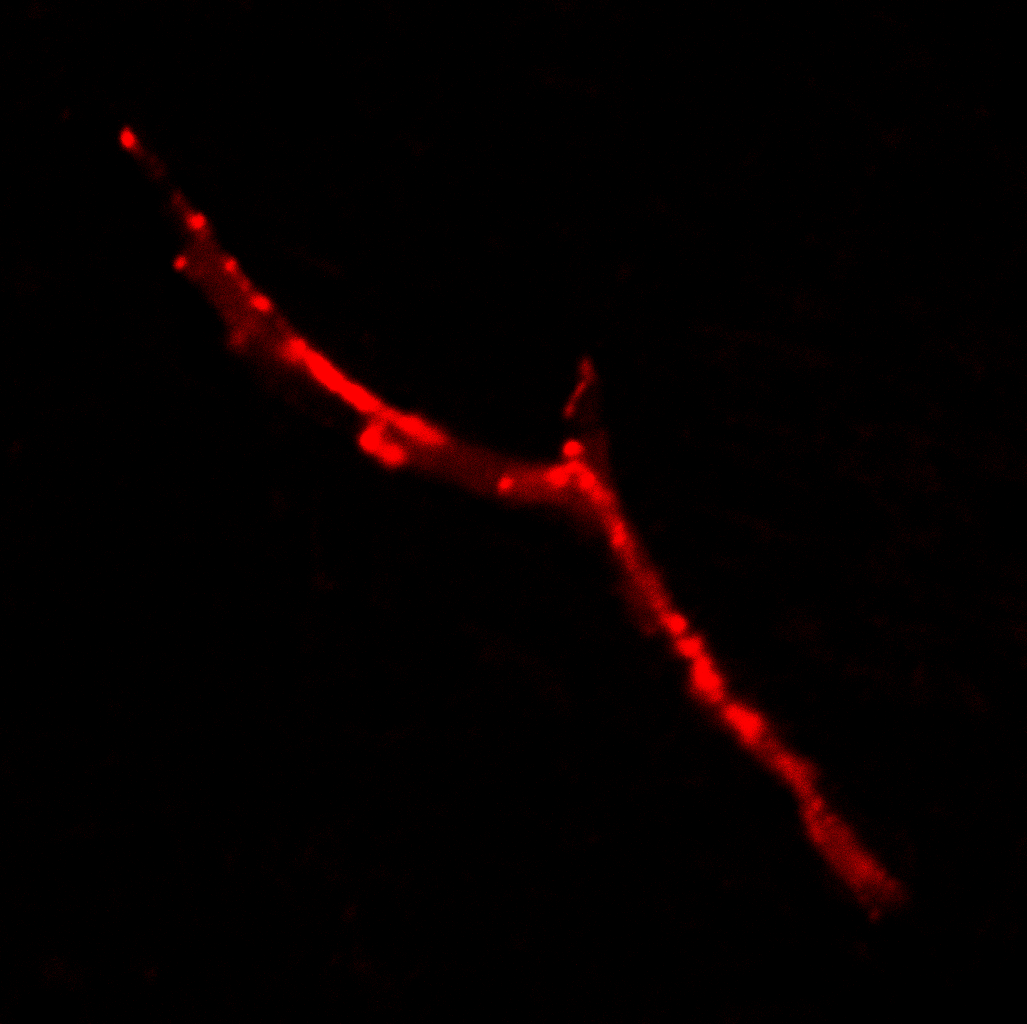

Supplement: Supplementary file 12 [file DataSheet6.ZIP › Immunofluorescence (Figure 6G, part1)/1/2/3s_c1.tif]

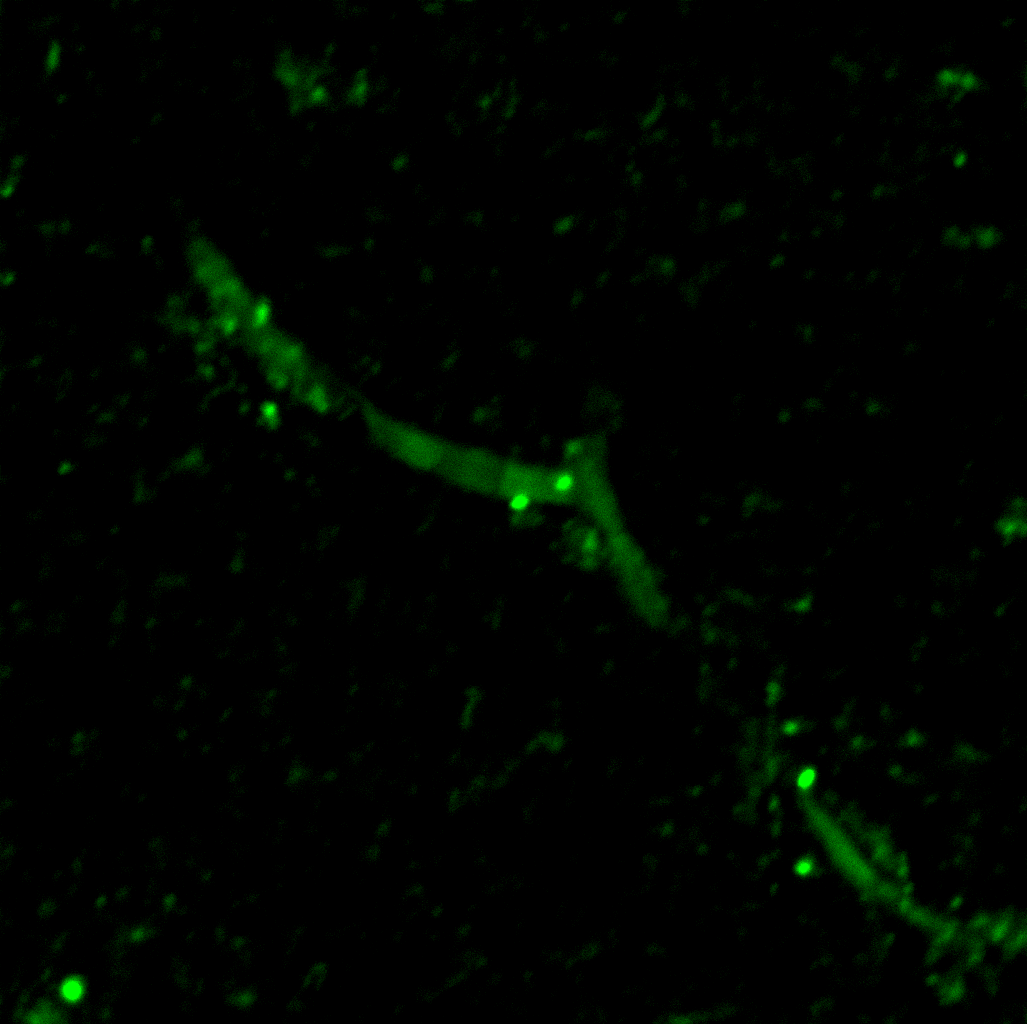

Supplement: Supplementary file 12 [file DataSheet6.ZIP › Immunofluorescence (Figure 6G, part1)/1/2/3s_c2.tif]

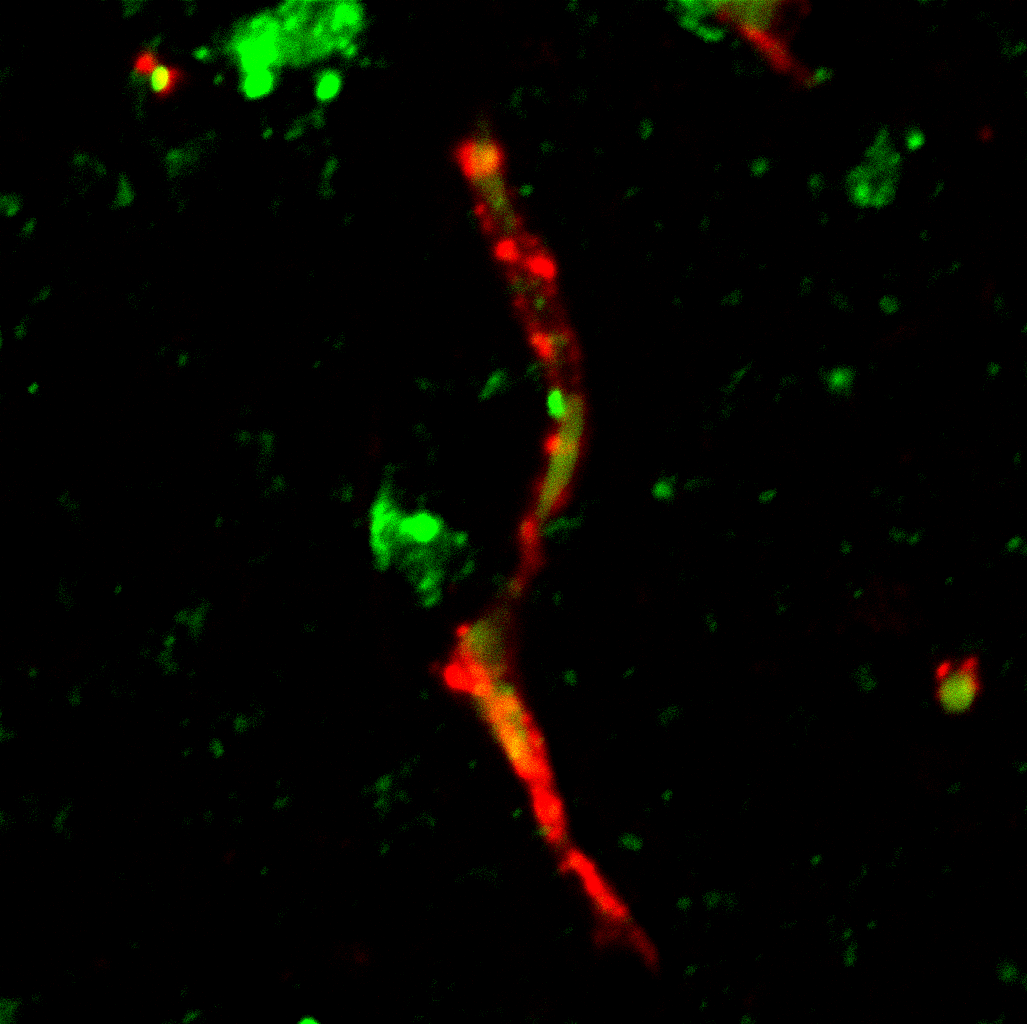

Supplement: Supplementary file 12 [file DataSheet6.ZIP › Immunofluorescence (Figure 6G, part1)/1/3/5s_c1+2.tif]

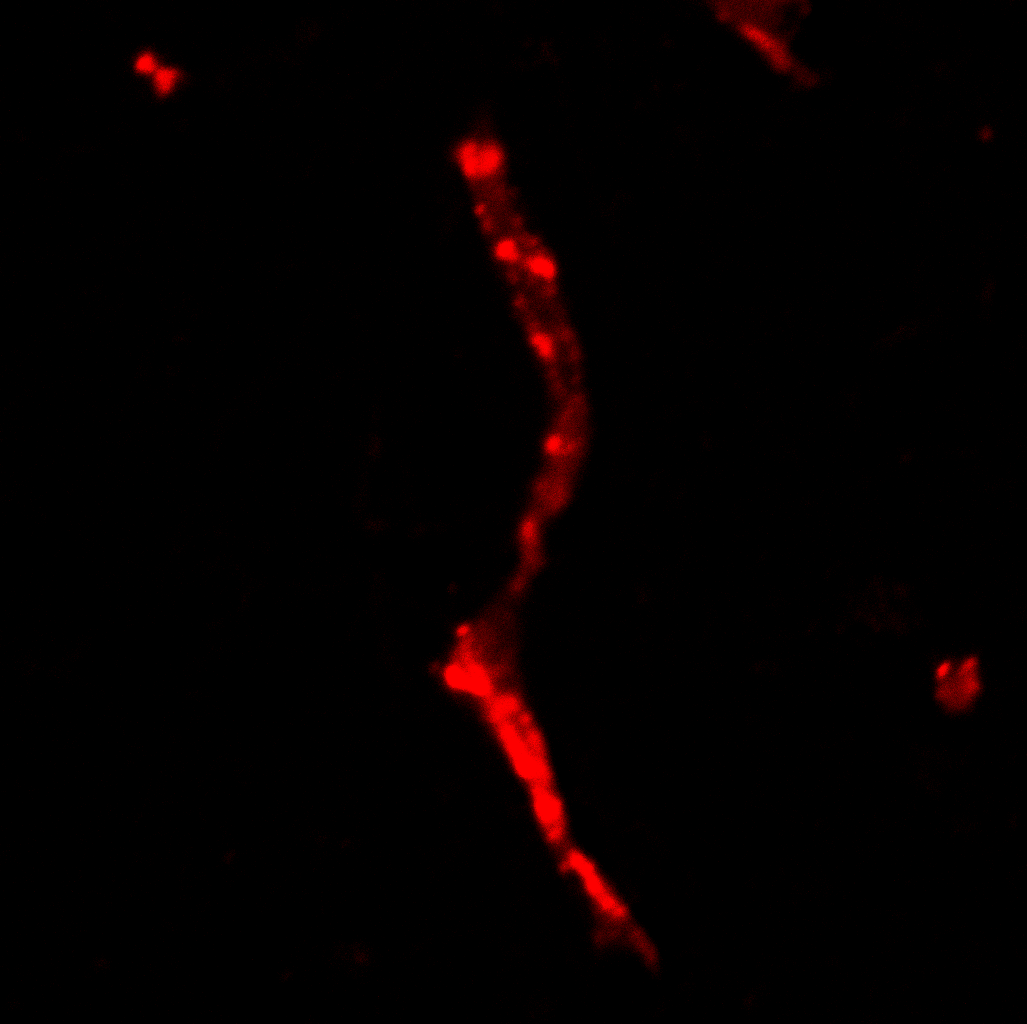

Supplement: Supplementary file 12 [file DataSheet6.ZIP › Immunofluorescence (Figure 6G, part1)/1/3/5s_c1.tif]

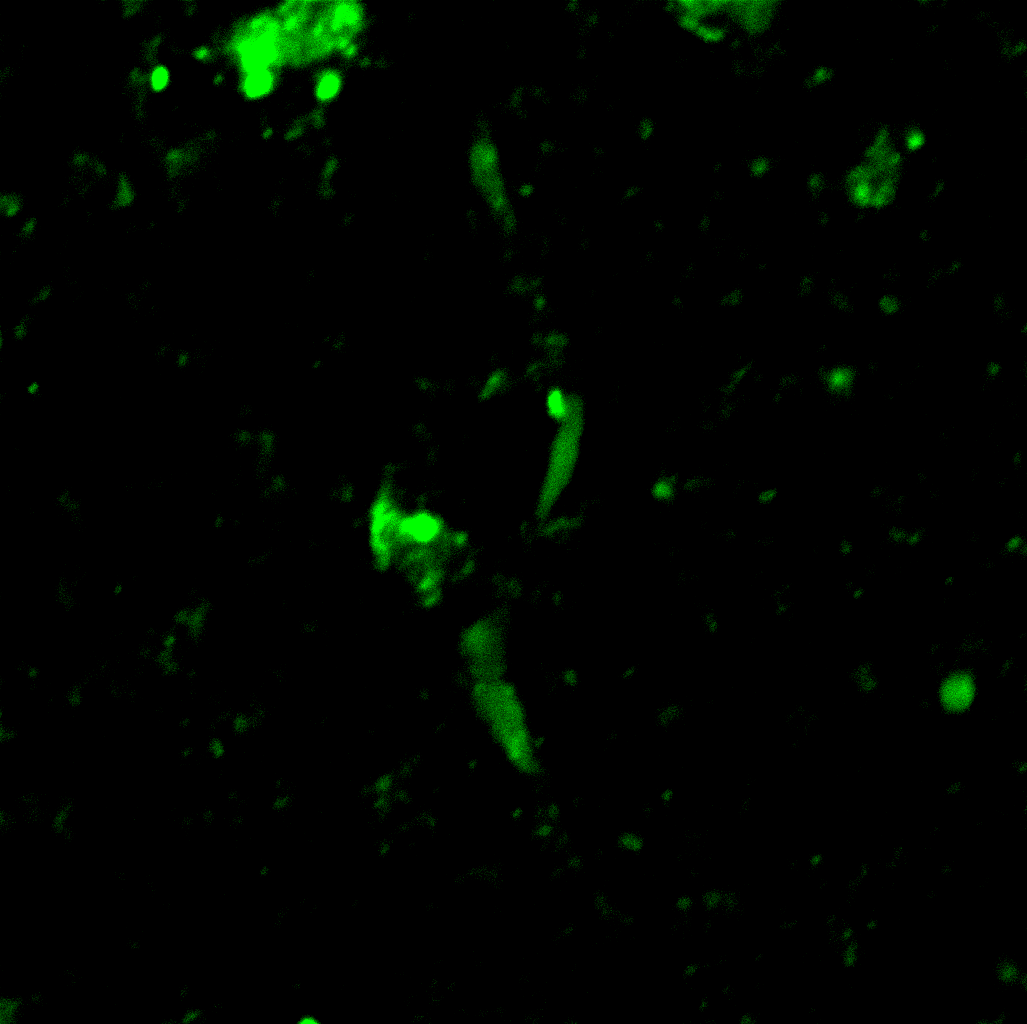

Supplement: Supplementary file 12 [file DataSheet6.ZIP › Immunofluorescence (Figure 6G, part1)/1/3/5s_c2.tif]

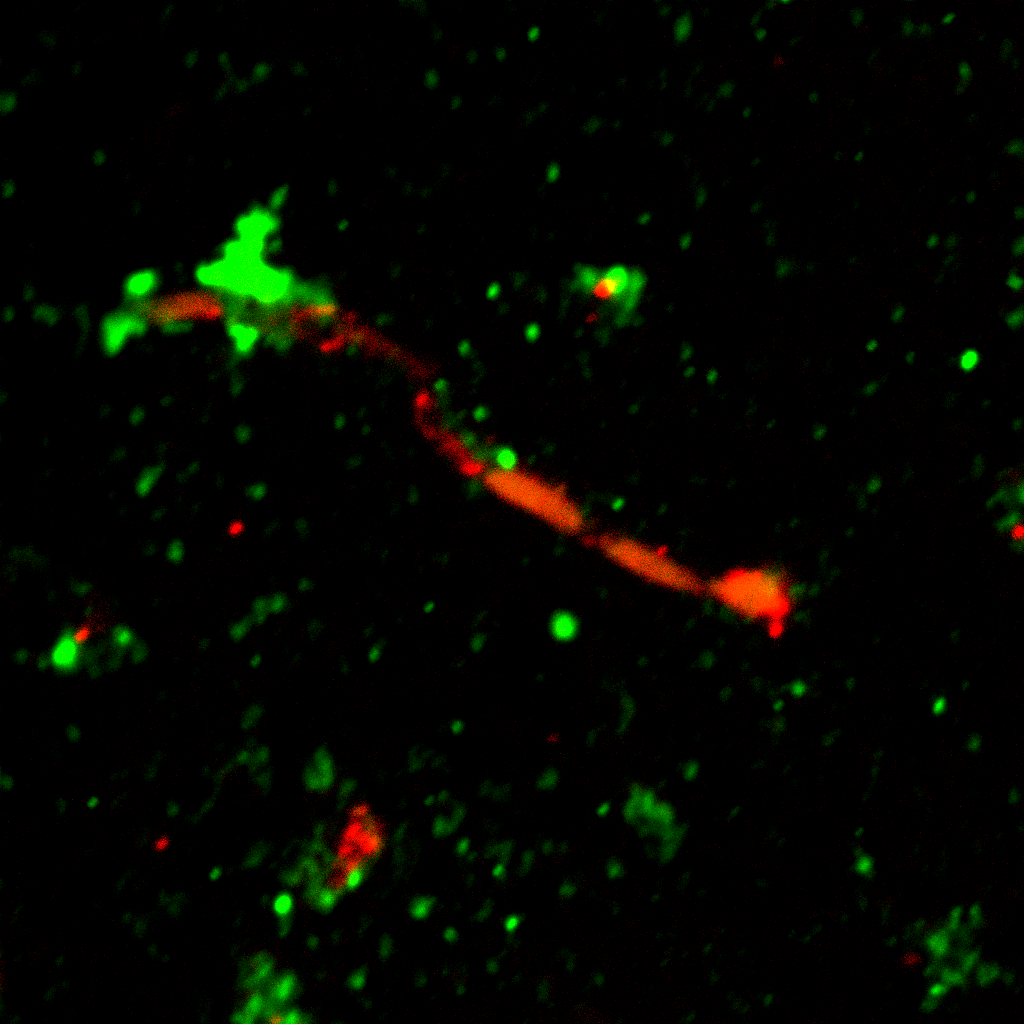

Supplement: Supplementary file 12 [file DataSheet6.ZIP › Immunofluorescence (Figure 6G, part1)/2/1/1s_c1+2.tif]

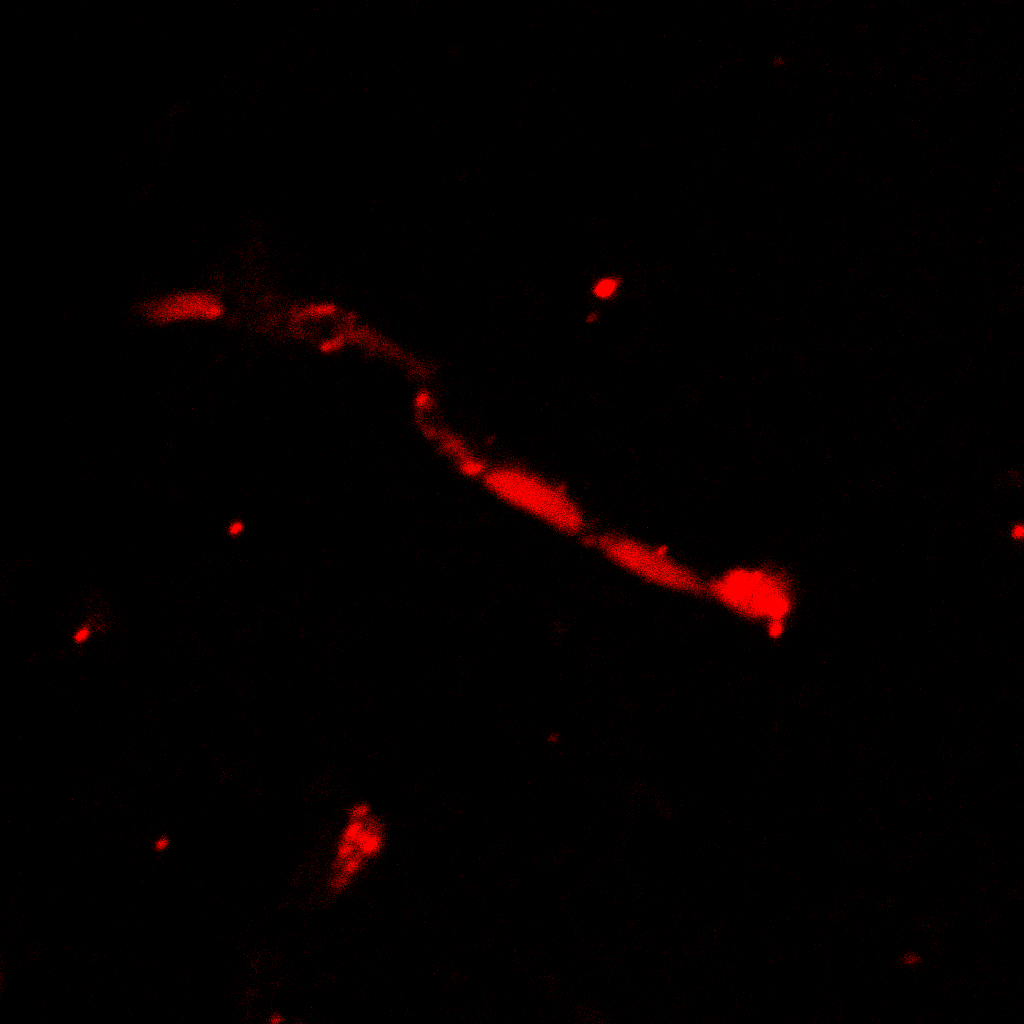

Supplement: Supplementary file 12 [file DataSheet6.ZIP › Immunofluorescence (Figure 6G, part1)/2/1/1s_c1.tif]

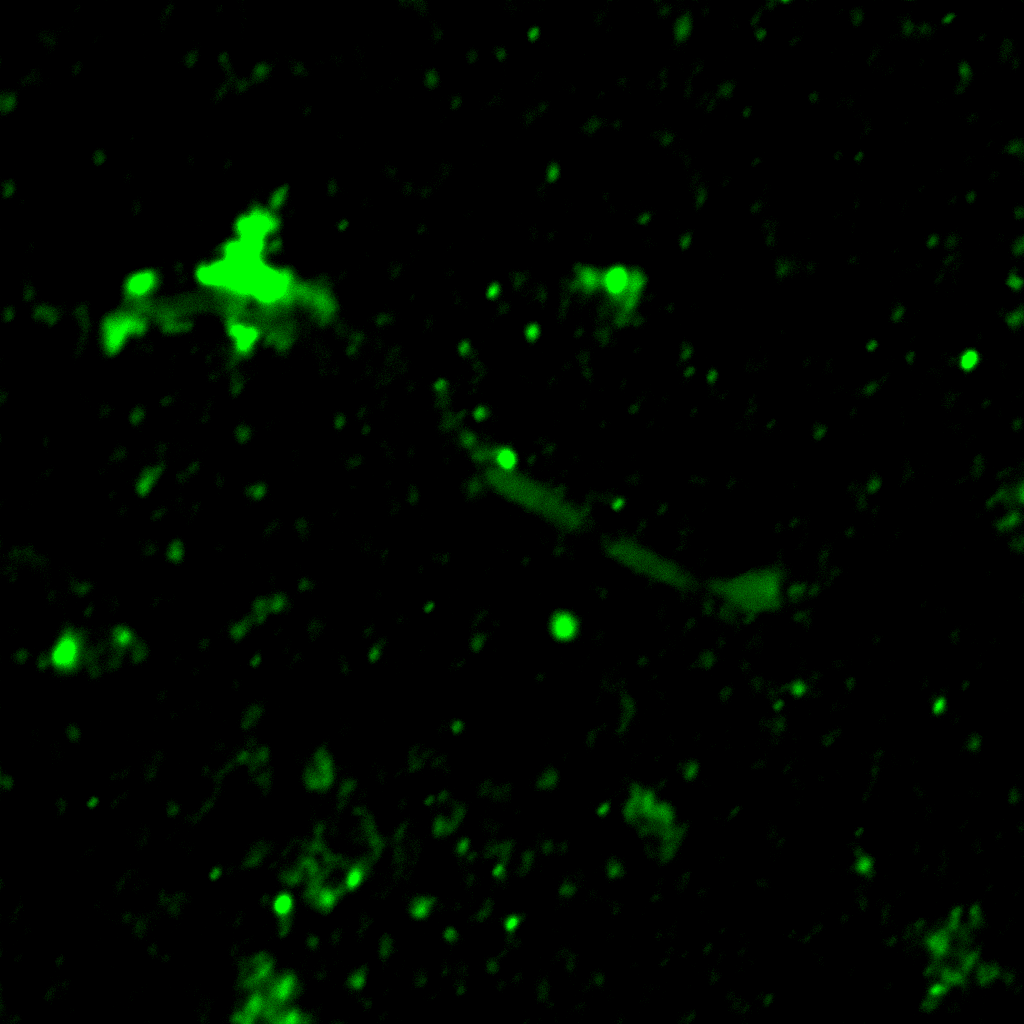

Supplement: Supplementary file 12 [file DataSheet6.ZIP › Immunofluorescence (Figure 6G, part1)/2/1/1s_c2.tif]

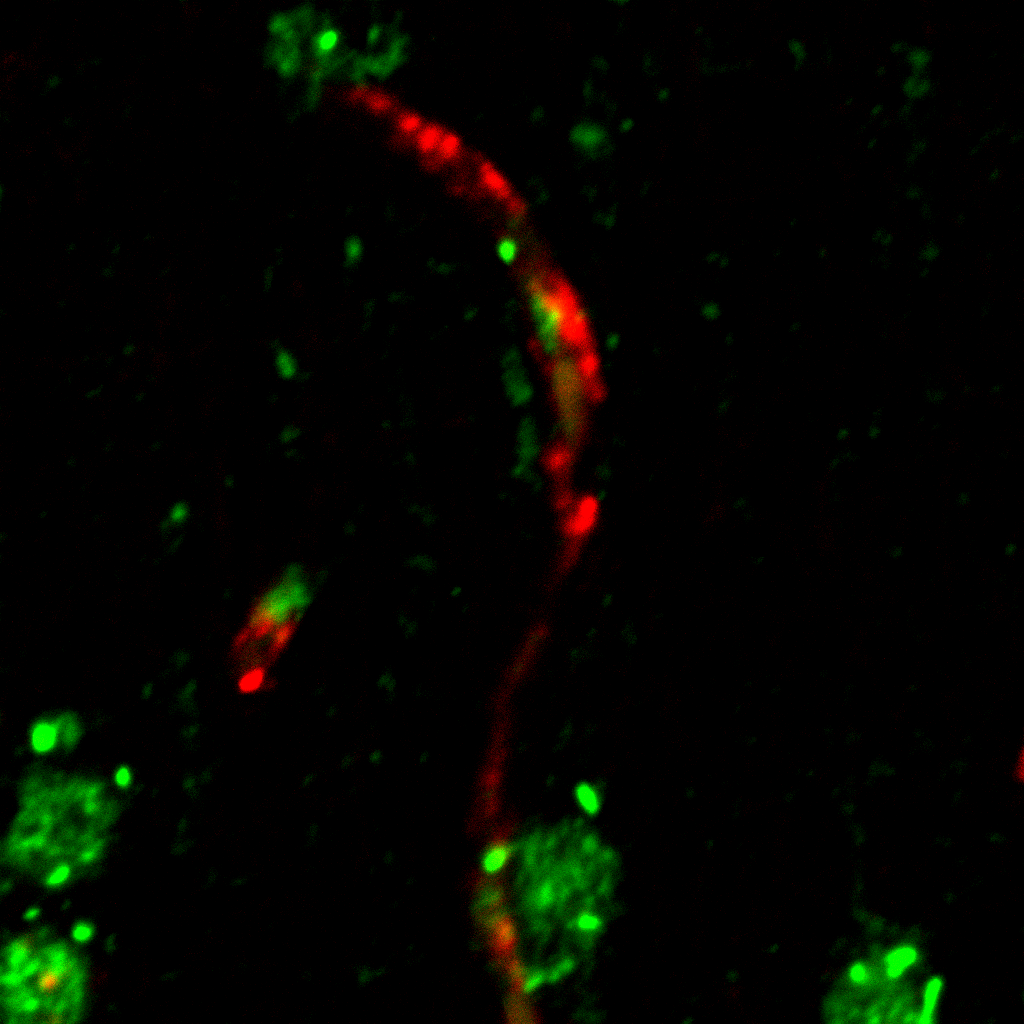

Supplement: Supplementary file 12 [file DataSheet6.ZIP › Immunofluorescence (Figure 6G, part1)/2/2/3s_c1+2.tif]

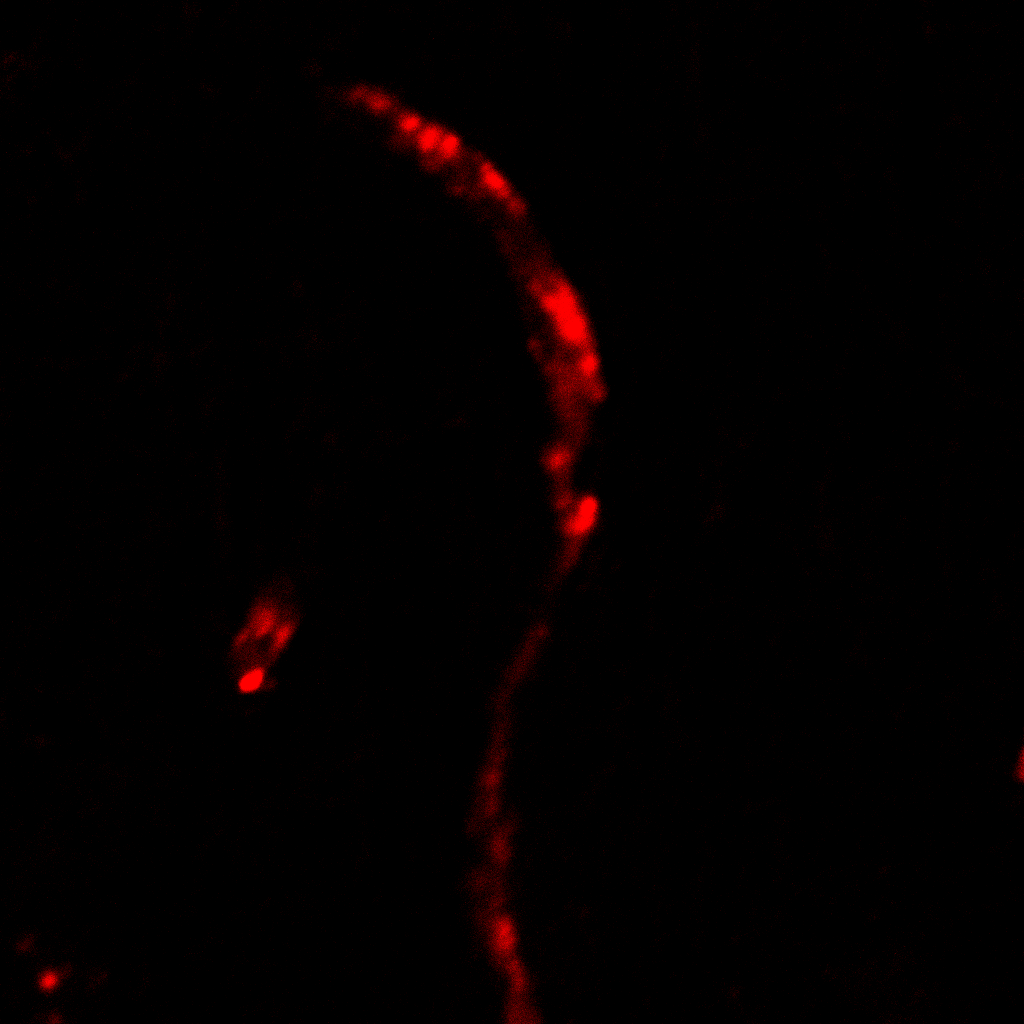

Supplement: Supplementary file 12 [file DataSheet6.ZIP › Immunofluorescence (Figure 6G, part1)/2/2/3s_c1.tif]

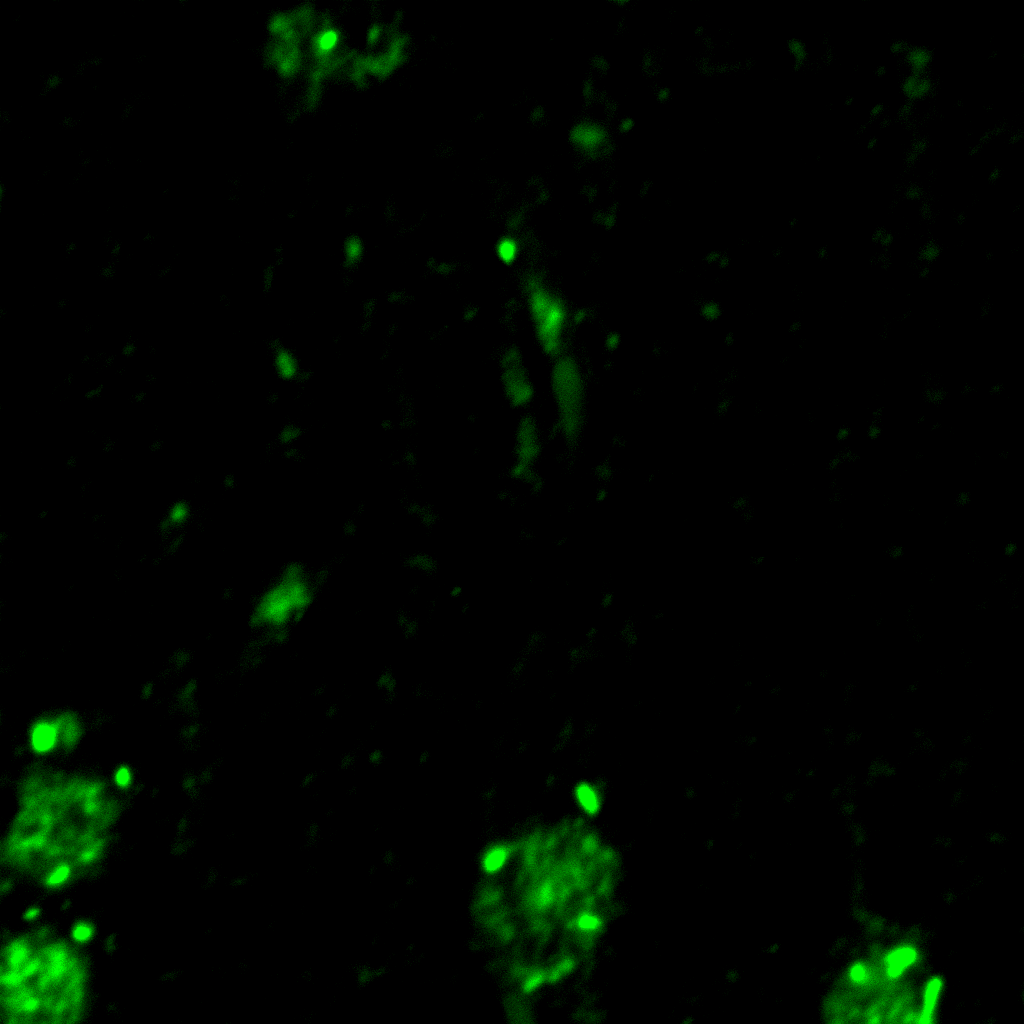

Supplement: Supplementary file 12 [file DataSheet6.ZIP › Immunofluorescence (Figure 6G, part1)/2/2/3s_c2.tif]

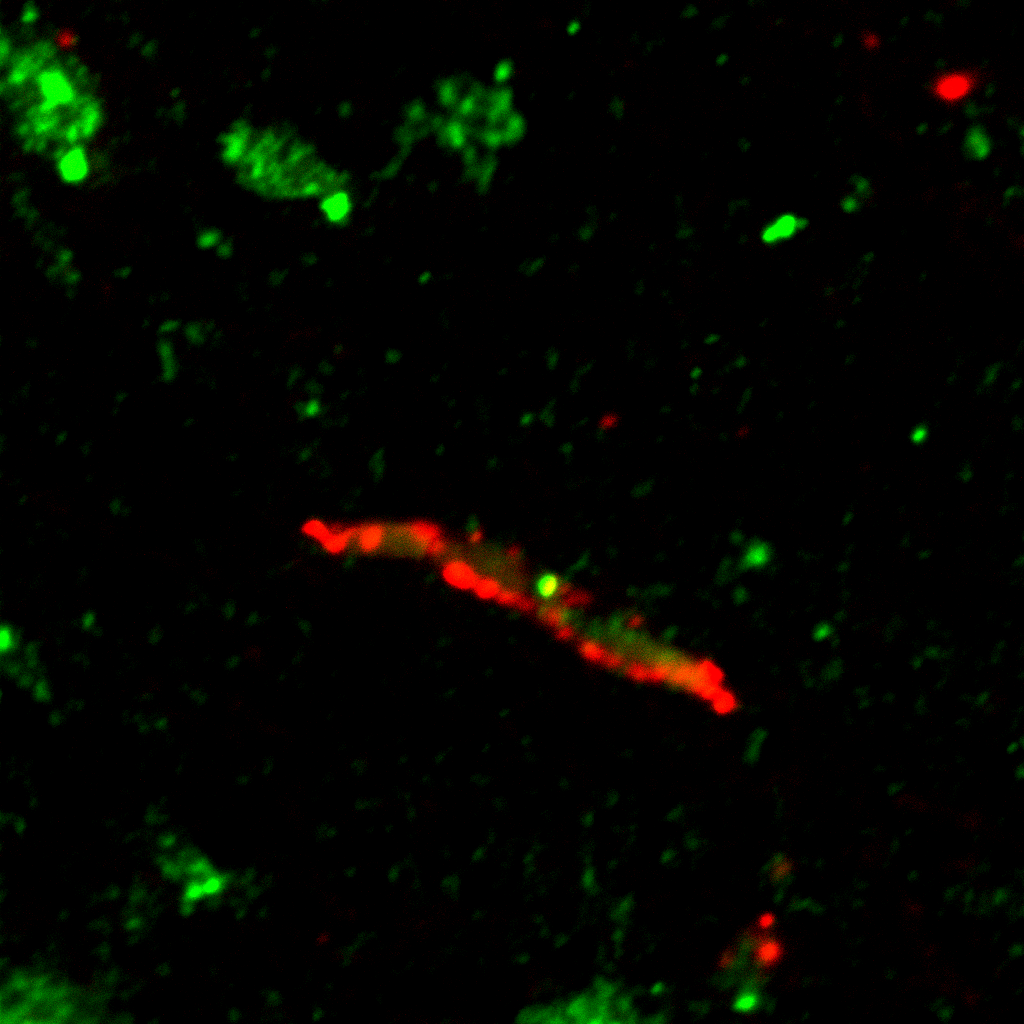

Supplement: Supplementary file 12 [file DataSheet6.ZIP › Immunofluorescence (Figure 6G, part1)/2/3/4s_c1+2.tif]

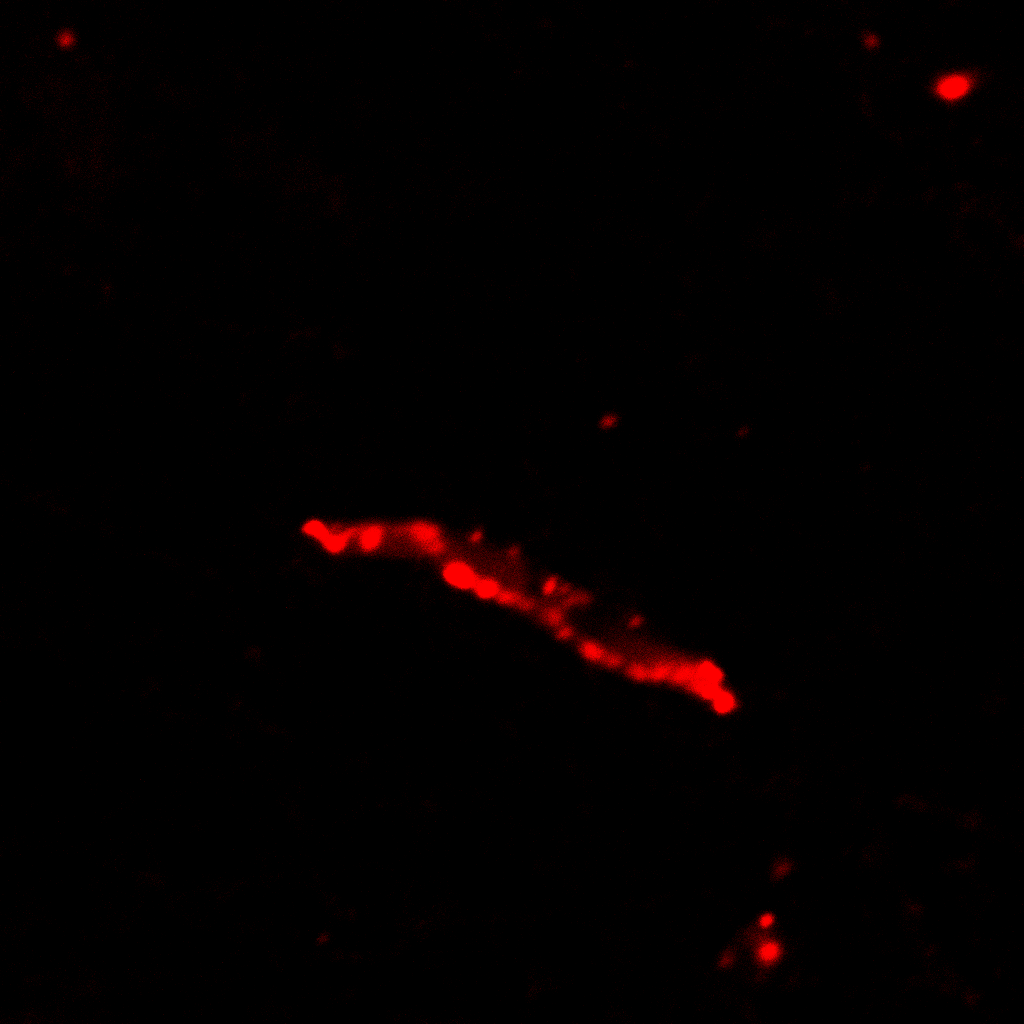

Supplement: Supplementary file 12 [file DataSheet6.ZIP › Immunofluorescence (Figure 6G, part1)/2/3/4s_c1.tif]

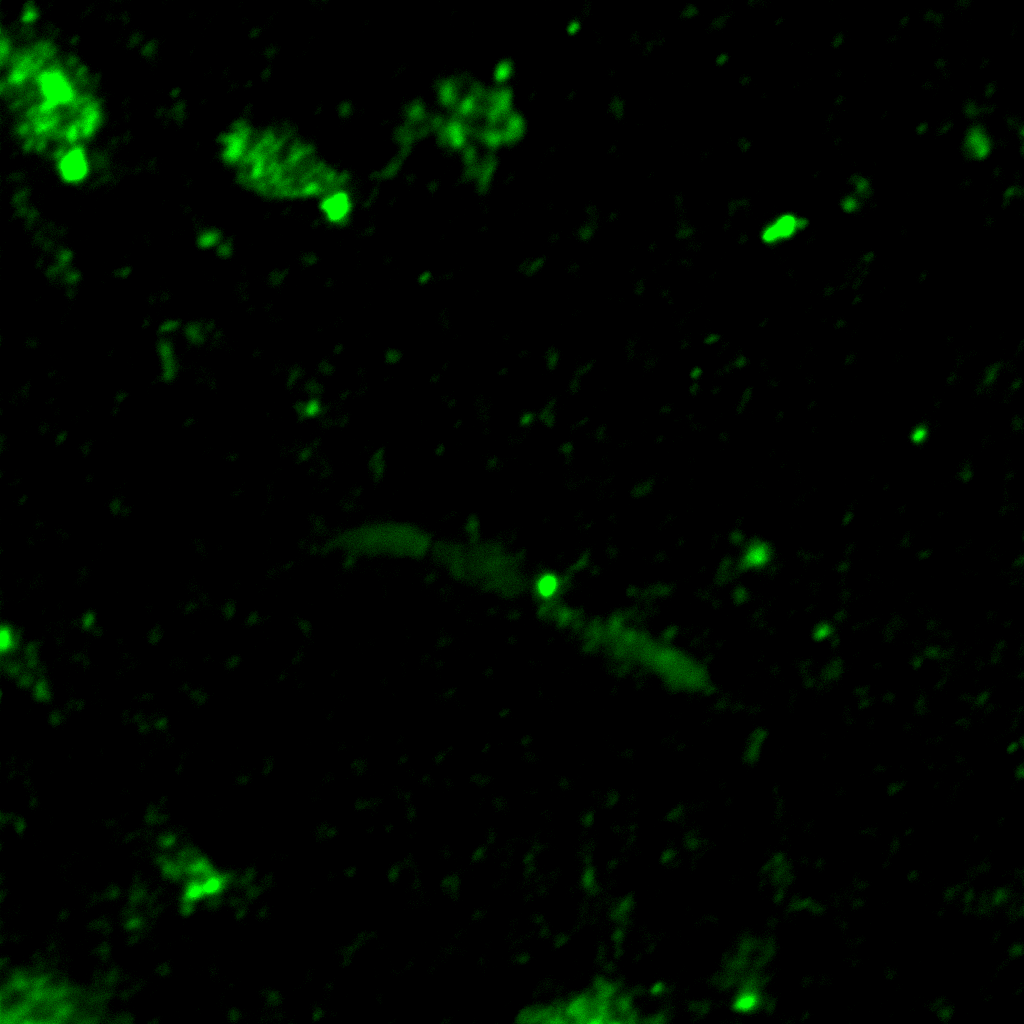

Supplement: Supplementary file 12 [file DataSheet6.ZIP › Immunofluorescence (Figure 6G, part1)/2/3/4s_c2.tif]

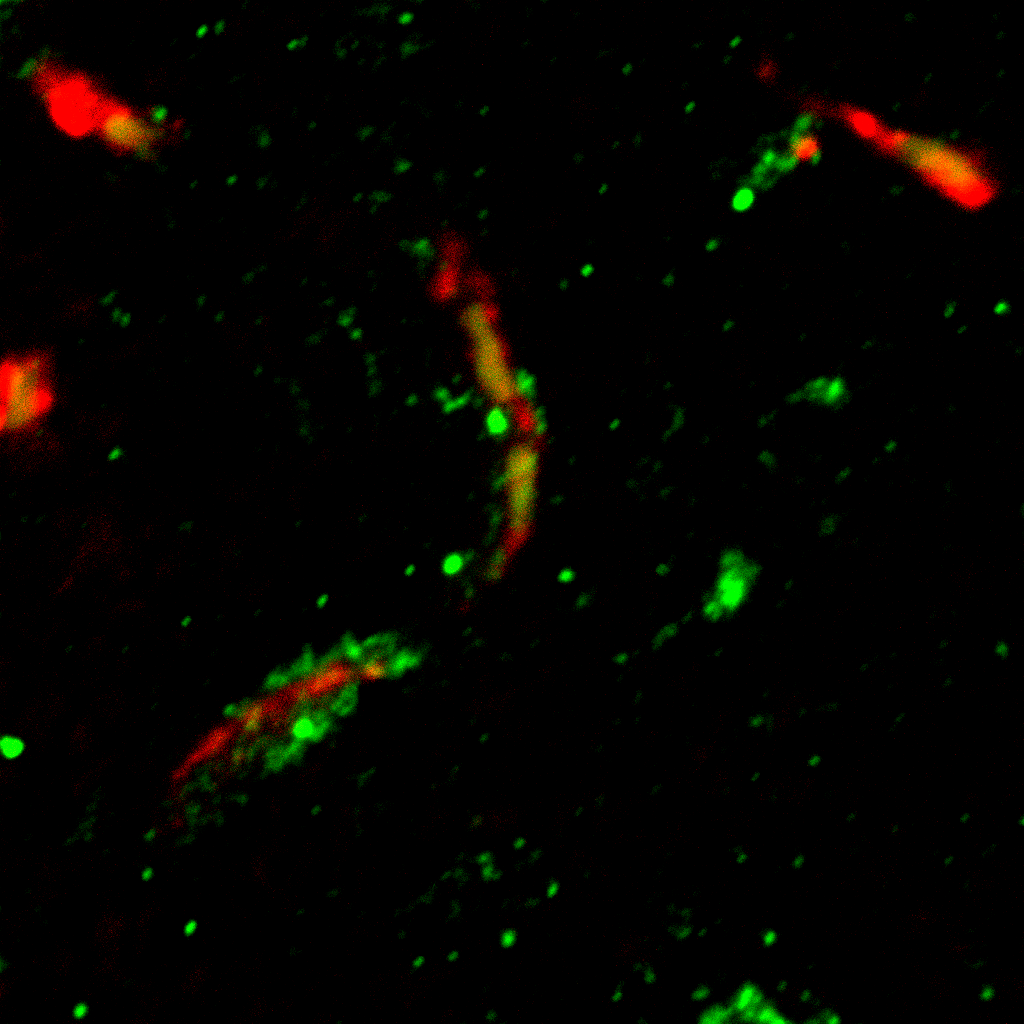

Supplement: Supplementary file 12 [file DataSheet6.ZIP › Immunofluorescence (Figure 6G, part1)/3/1/2s_c1+2.tif]

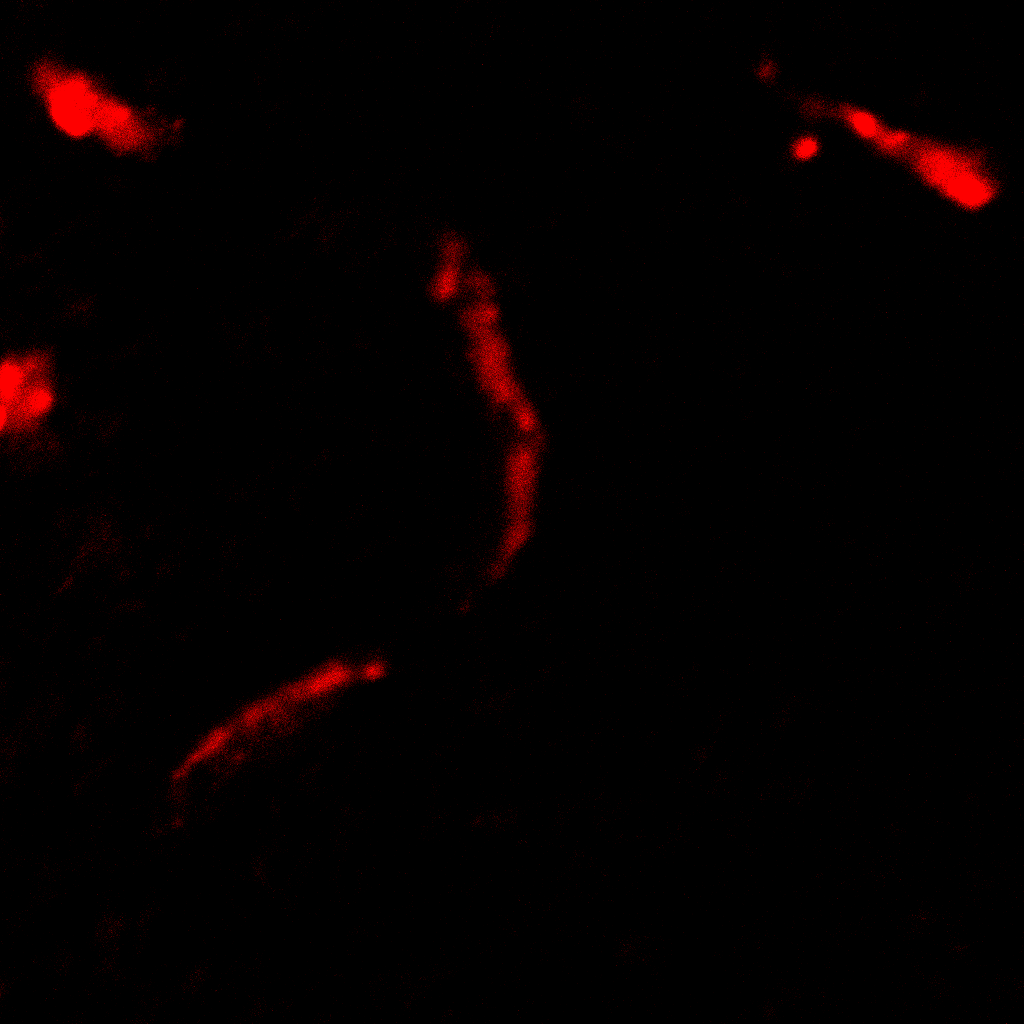

Supplement: Supplementary file 12 [file DataSheet6.ZIP › Immunofluorescence (Figure 6G, part1)/3/1/2s_c1.tif]

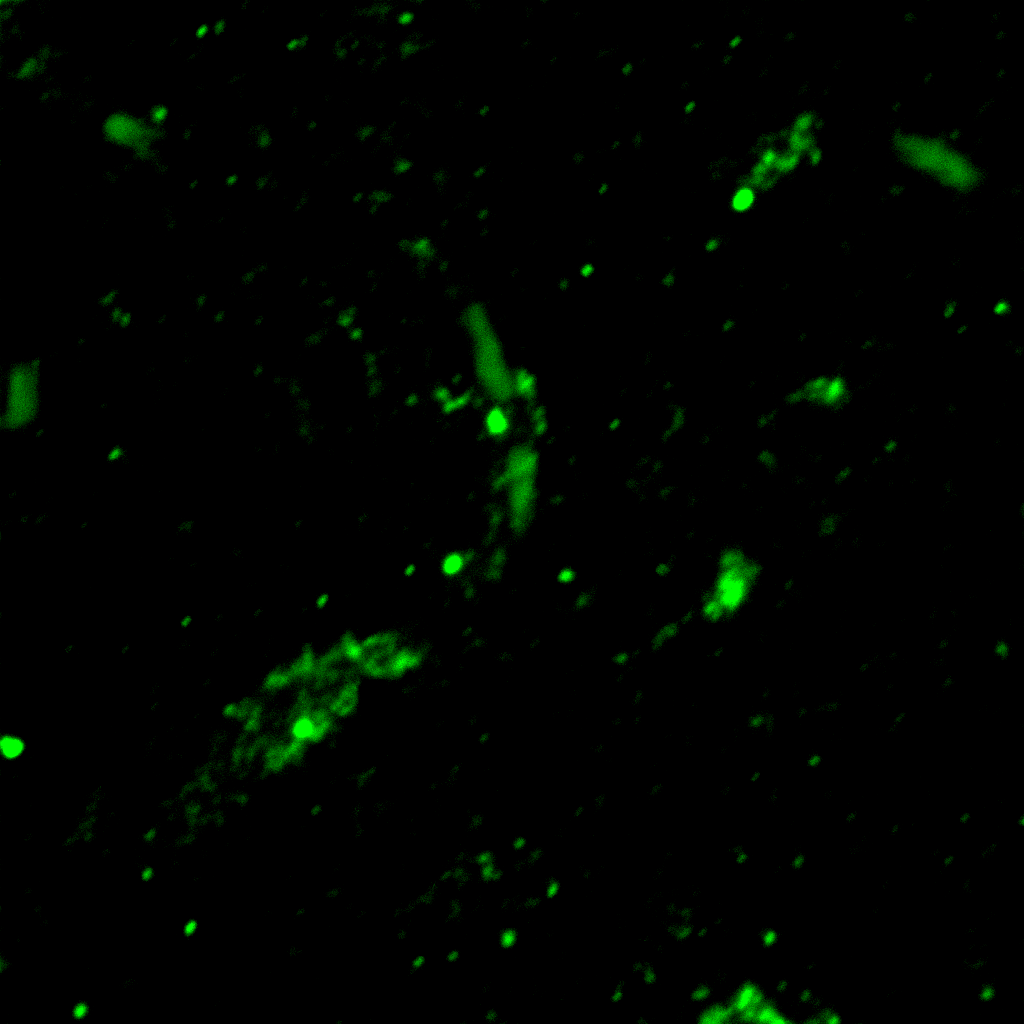

Supplement: Supplementary file 12 [file DataSheet6.ZIP › Immunofluorescence (Figure 6G, part1)/3/1/2s_c2.tif]

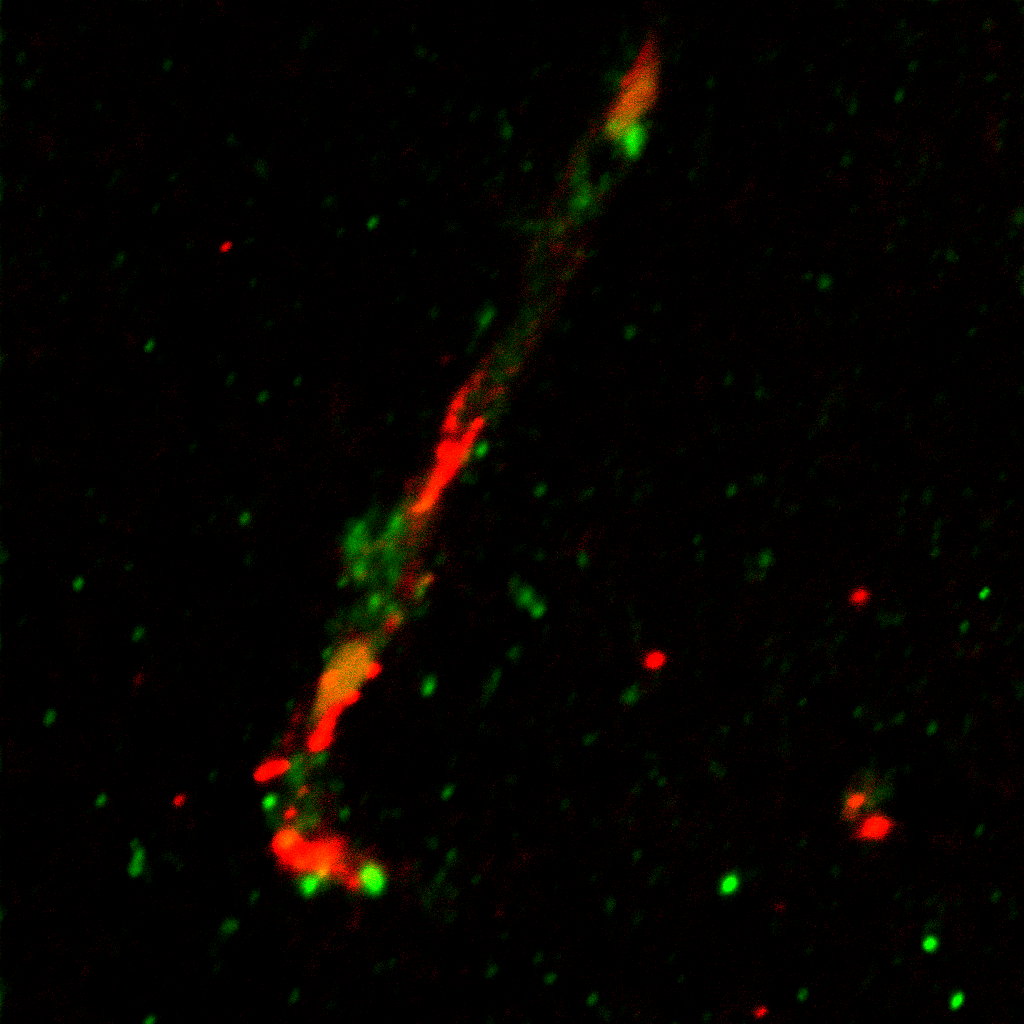

Supplement: Supplementary file 12 [file DataSheet6.ZIP › Immunofluorescence (Figure 6G, part1)/3/2/4s_c1+2.tif]
