# Supplementary figures and images for: Hepatic Ischemia-Reperfusion Impairs Blood-Brain Barrier Partly Due to Release of Arginase From Injured Liver (part 3 of 3)
Source: Front Pharmacol. 2021 Oct 13;12:724471. doi: 10.3389/fphar.2021.724471 (PMC8548691; doi:10.3389/fphar.2021.724471)

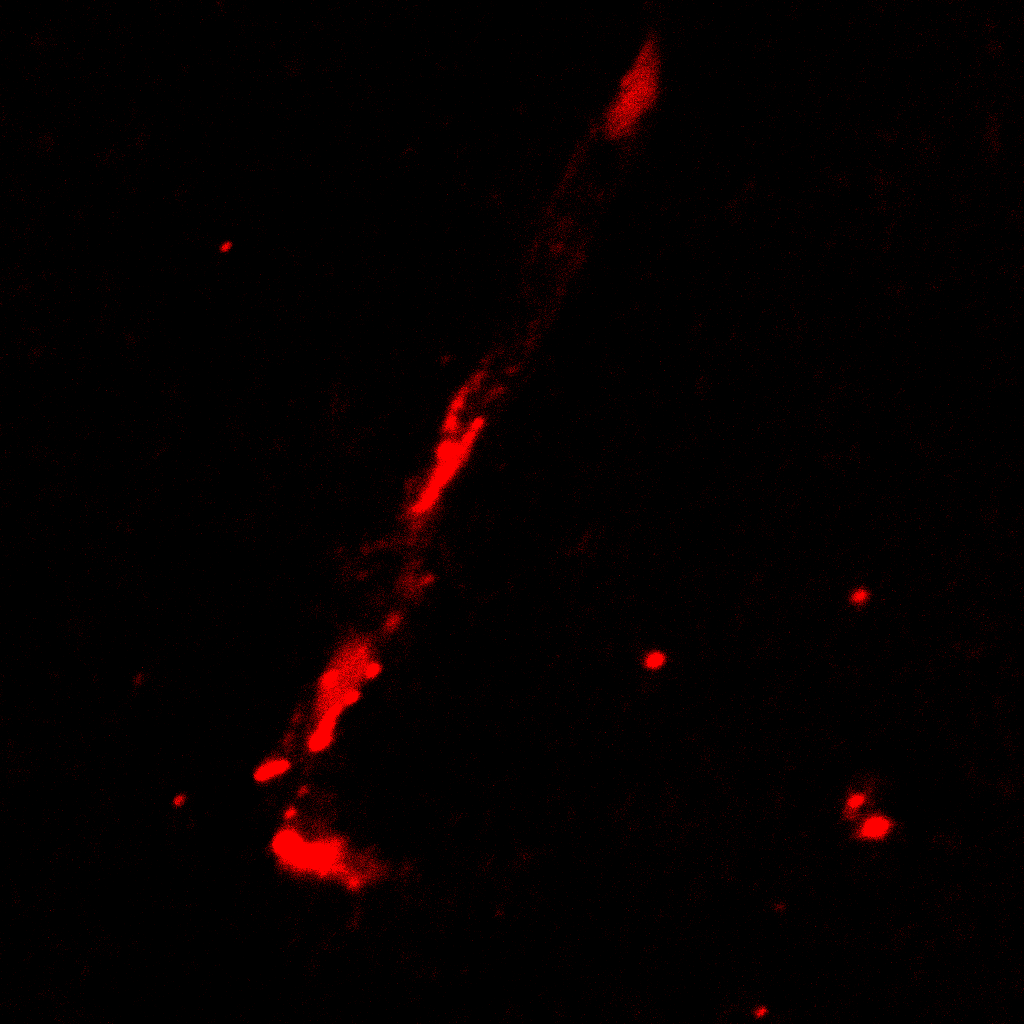

Supplement: Supplementary file 12 [file DataSheet6.ZIP › Immunofluorescence (Figure 6G, part1)/3/2/4s_c1.tif]

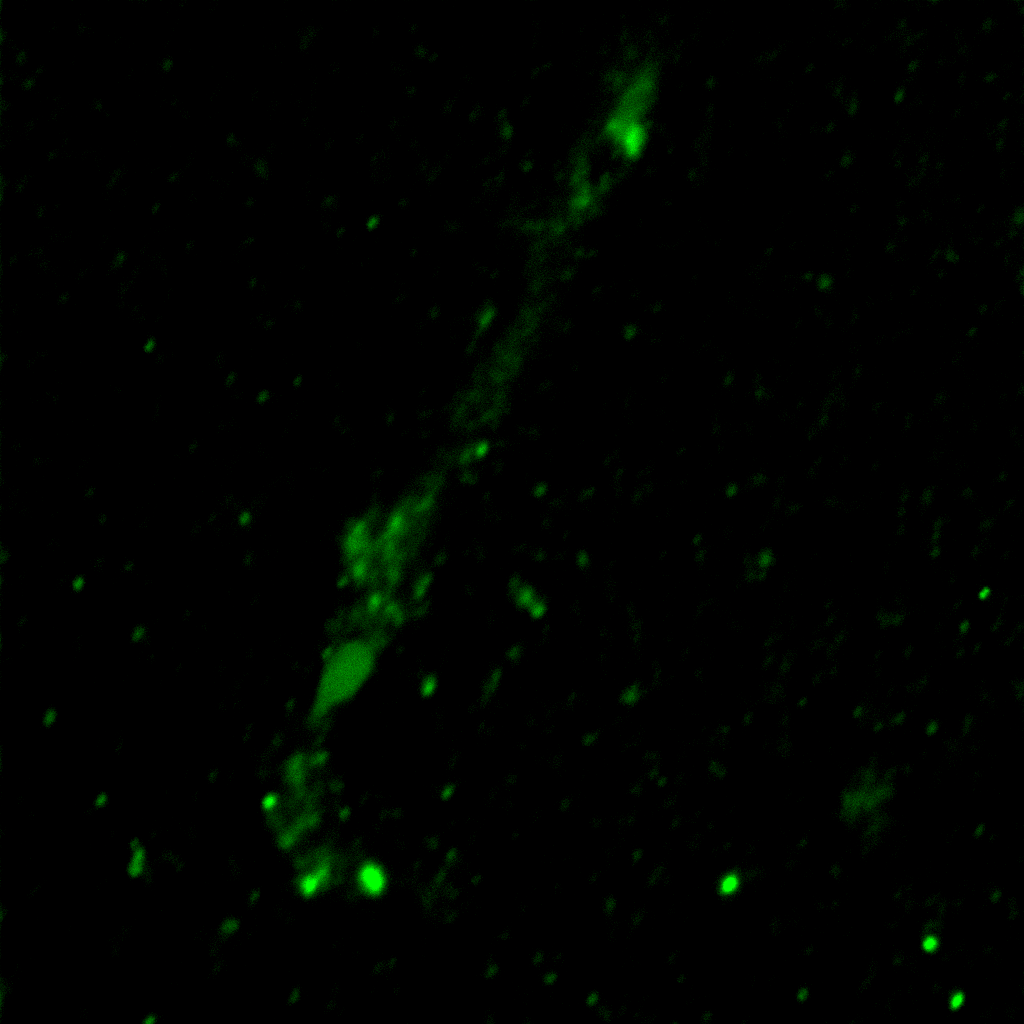

Supplement: Supplementary file 12 [file DataSheet6.ZIP › Immunofluorescence (Figure 6G, part1)/3/2/4s_c2.tif]

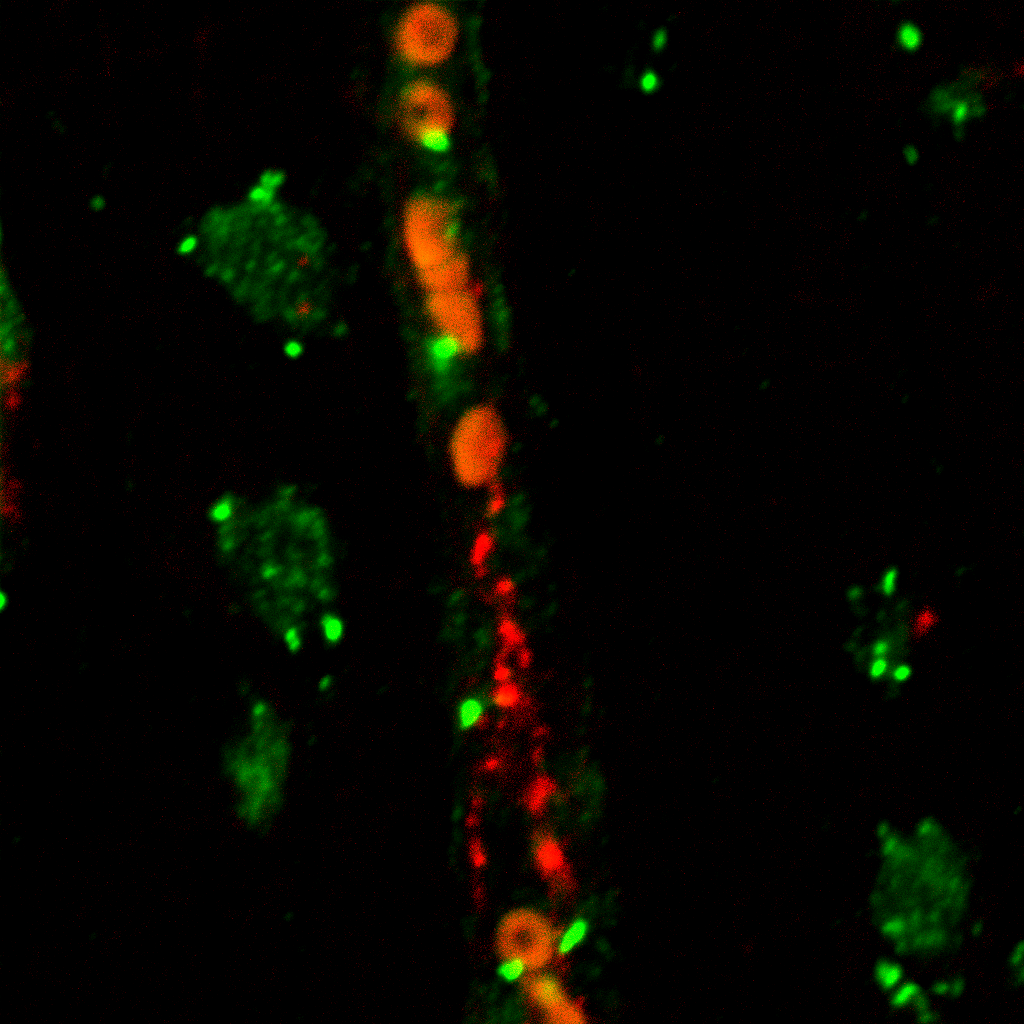

Supplement: Supplementary file 12 [file DataSheet6.ZIP › Immunofluorescence (Figure 6G, part1)/3/3/5s_c1+2.tif]

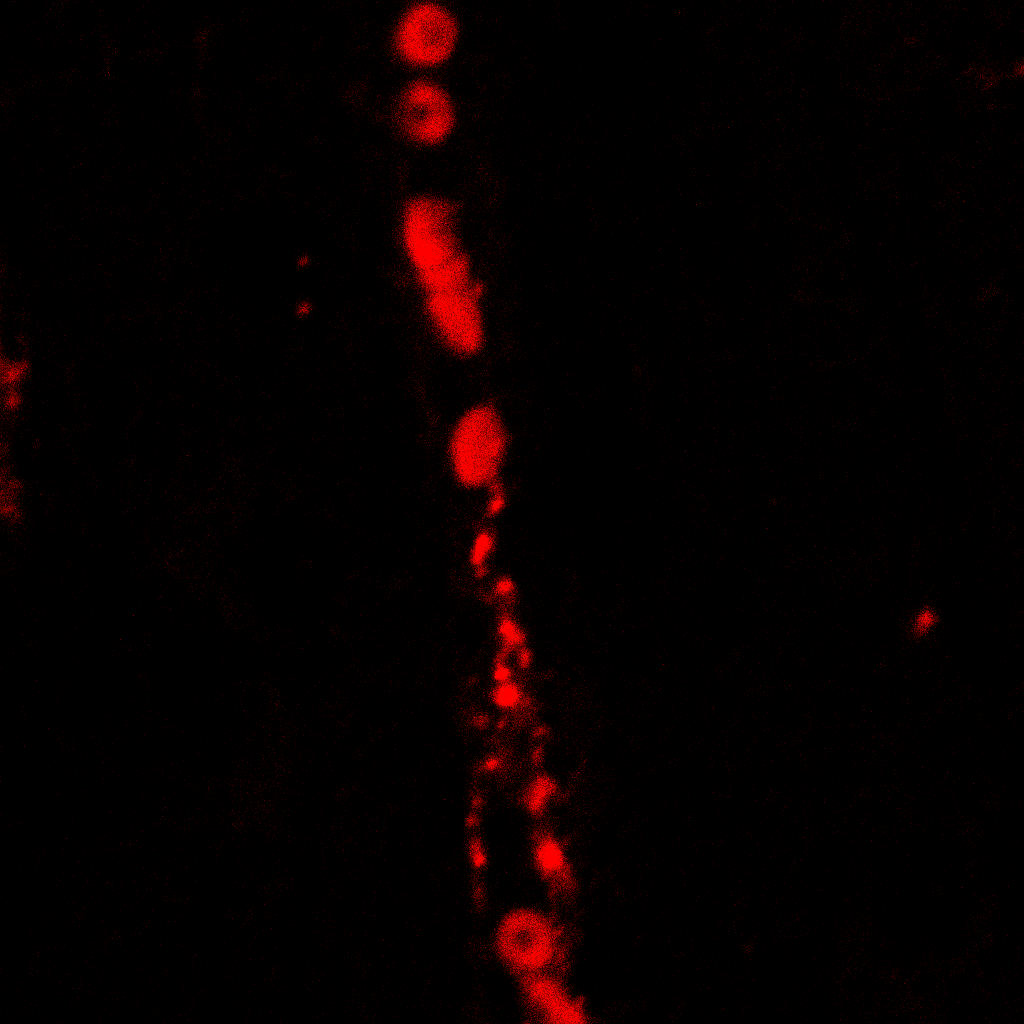

Supplement: Supplementary file 12 [file DataSheet6.ZIP › Immunofluorescence (Figure 6G, part1)/3/3/5s_c1.tif]

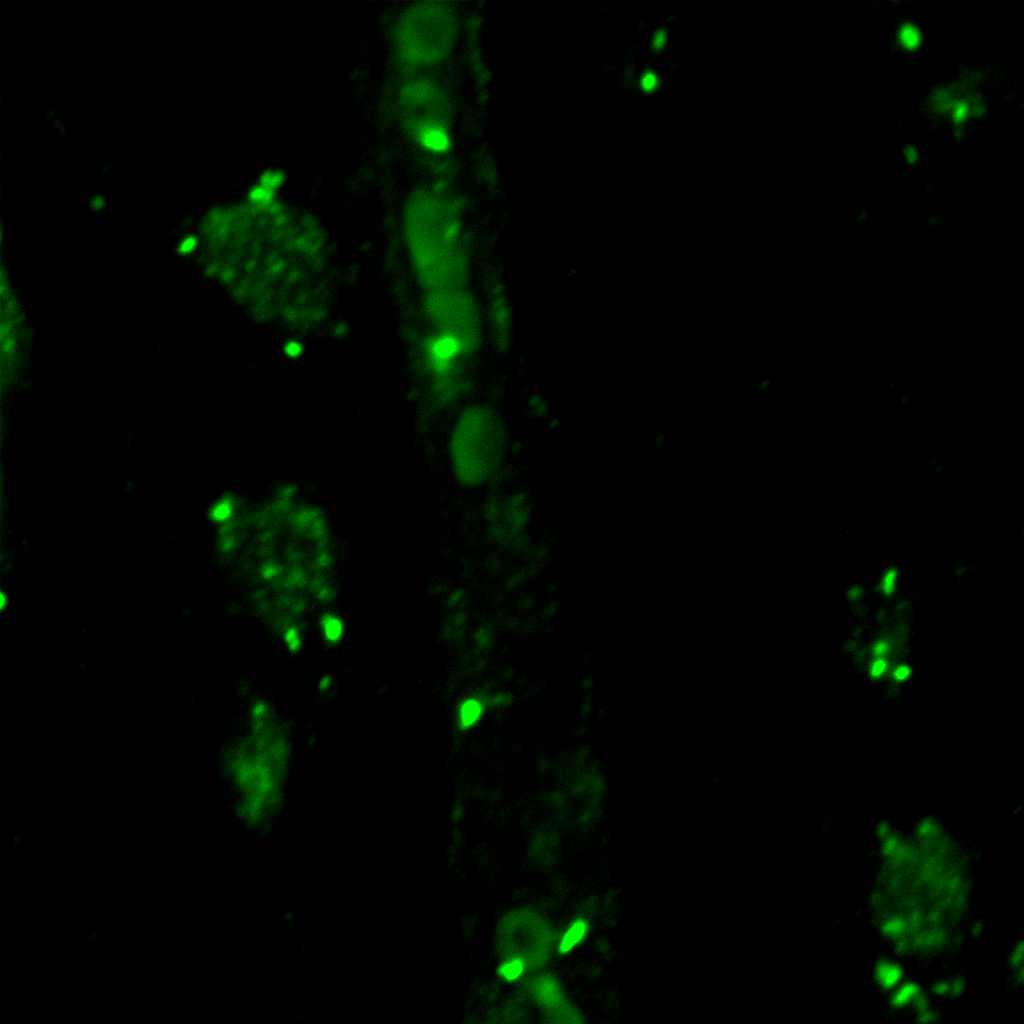

Supplement: Supplementary file 12 [file DataSheet6.ZIP › Immunofluorescence (Figure 6G, part1)/3/3/5s_c2.tif]

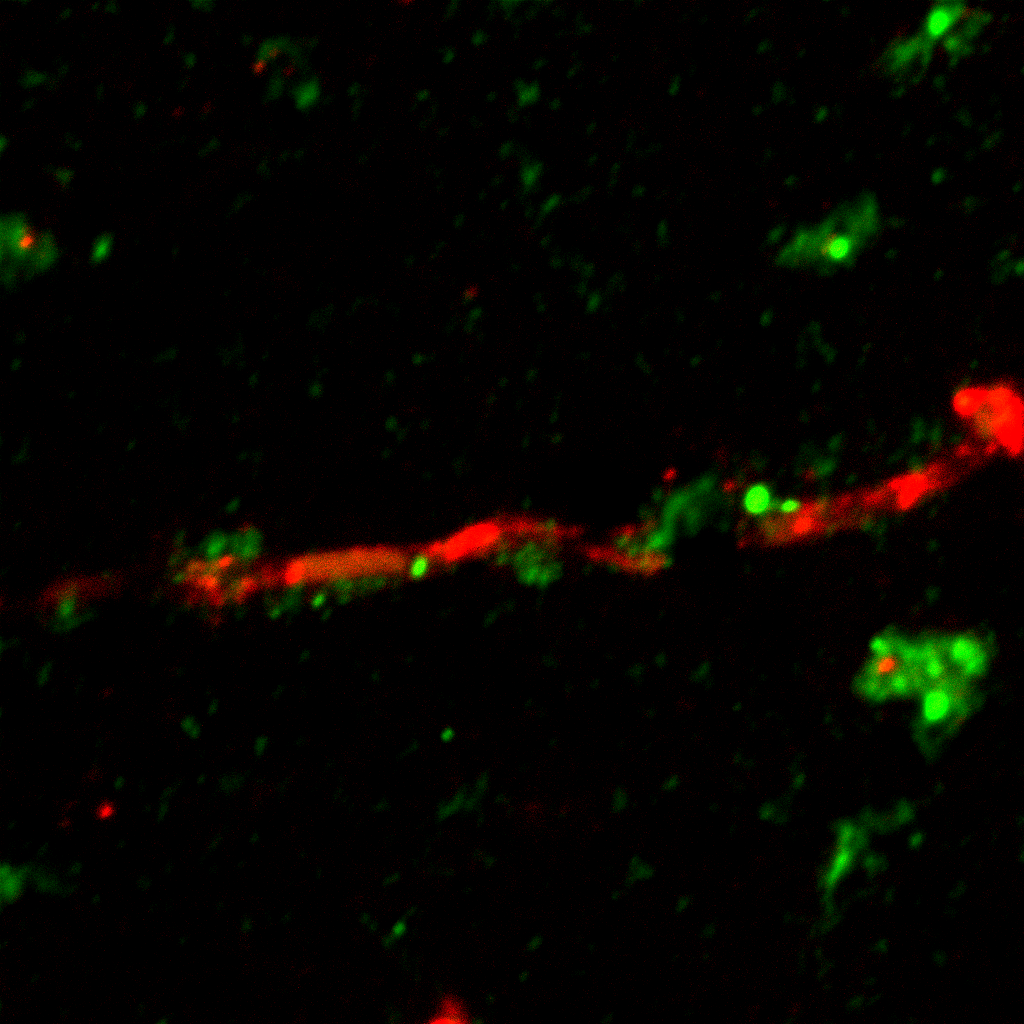

Supplement: Supplementary file 12 [file DataSheet6.ZIP › Immunofluorescence (Figure 6G, part1)/4/1/1s_c1+2.tif]

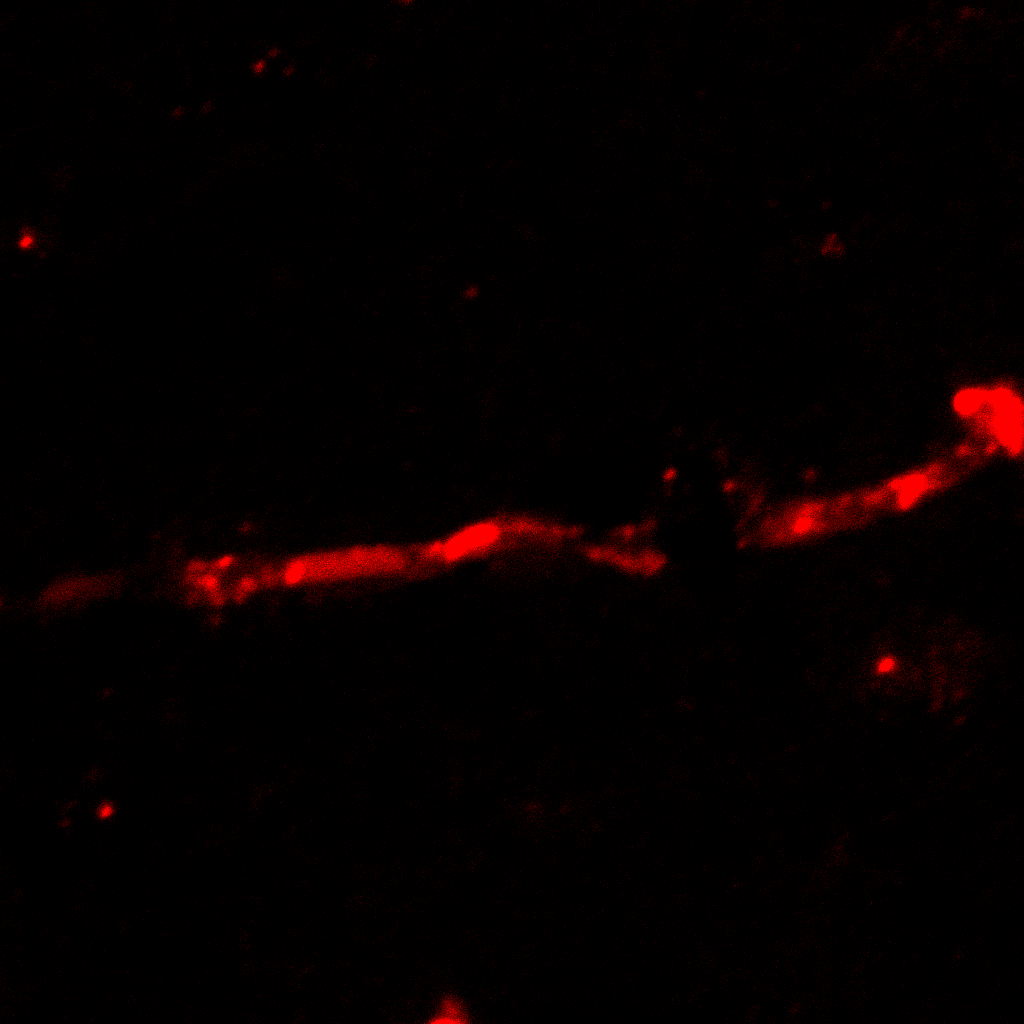

Supplement: Supplementary file 12 [file DataSheet6.ZIP › Immunofluorescence (Figure 6G, part1)/4/1/1s_c1.tif]

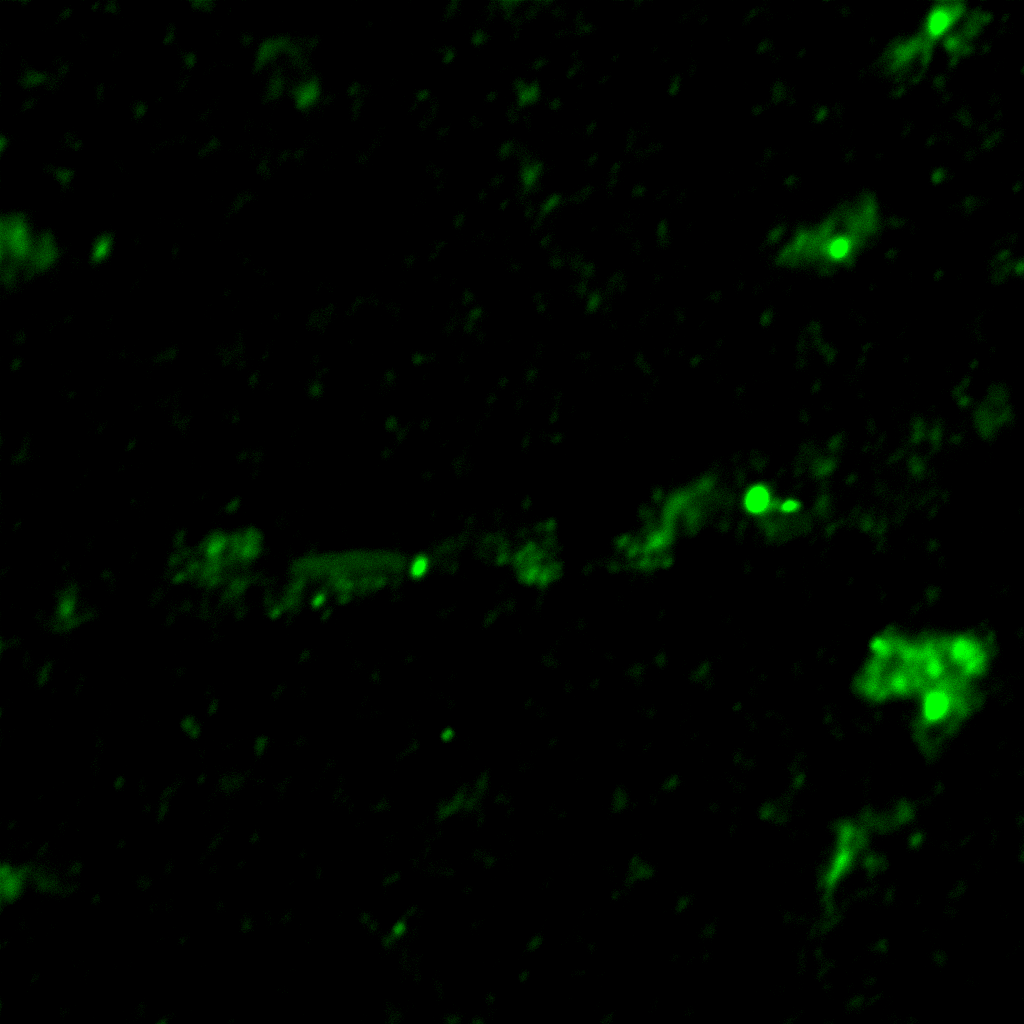

Supplement: Supplementary file 12 [file DataSheet6.ZIP › Immunofluorescence (Figure 6G, part1)/4/1/1s_c2.tif]

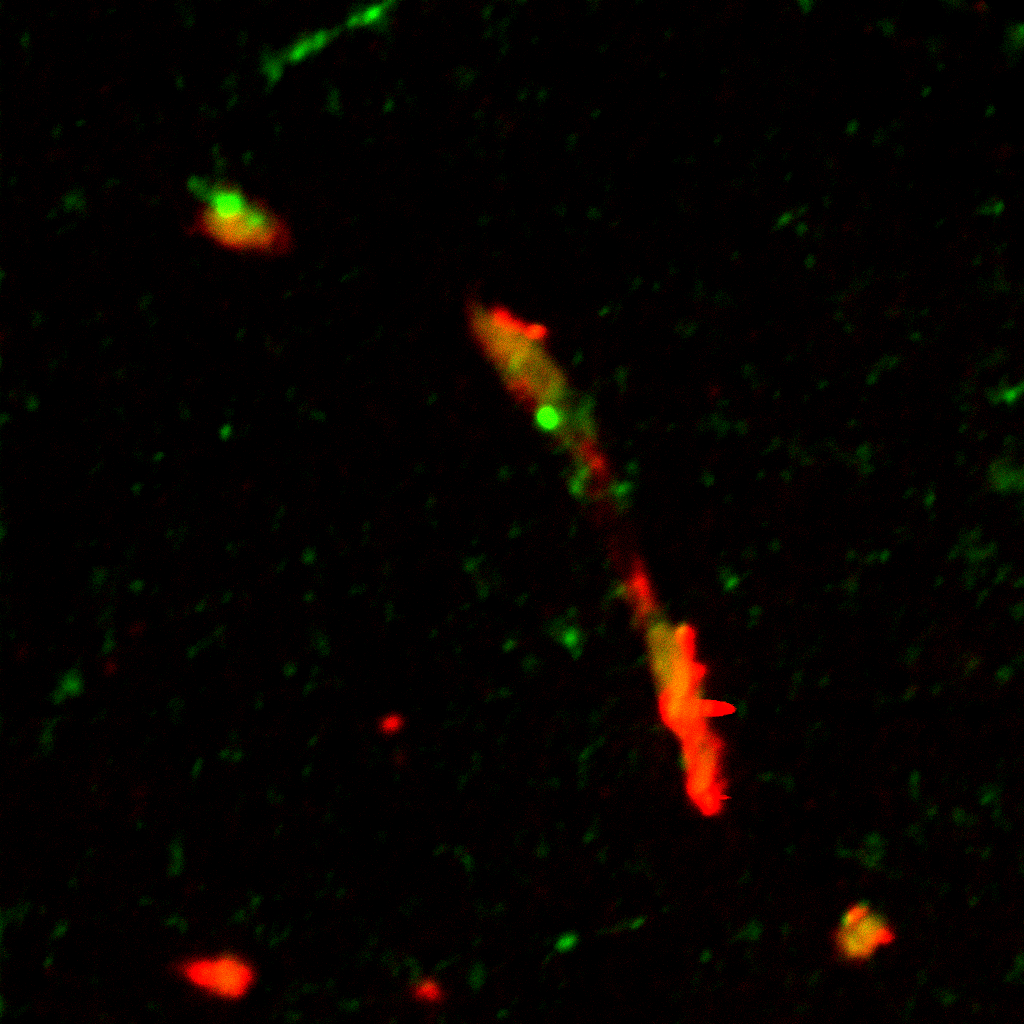

Supplement: Supplementary file 12 [file DataSheet6.ZIP › Immunofluorescence (Figure 6G, part1)/4/2/4s_c1+2.tif]

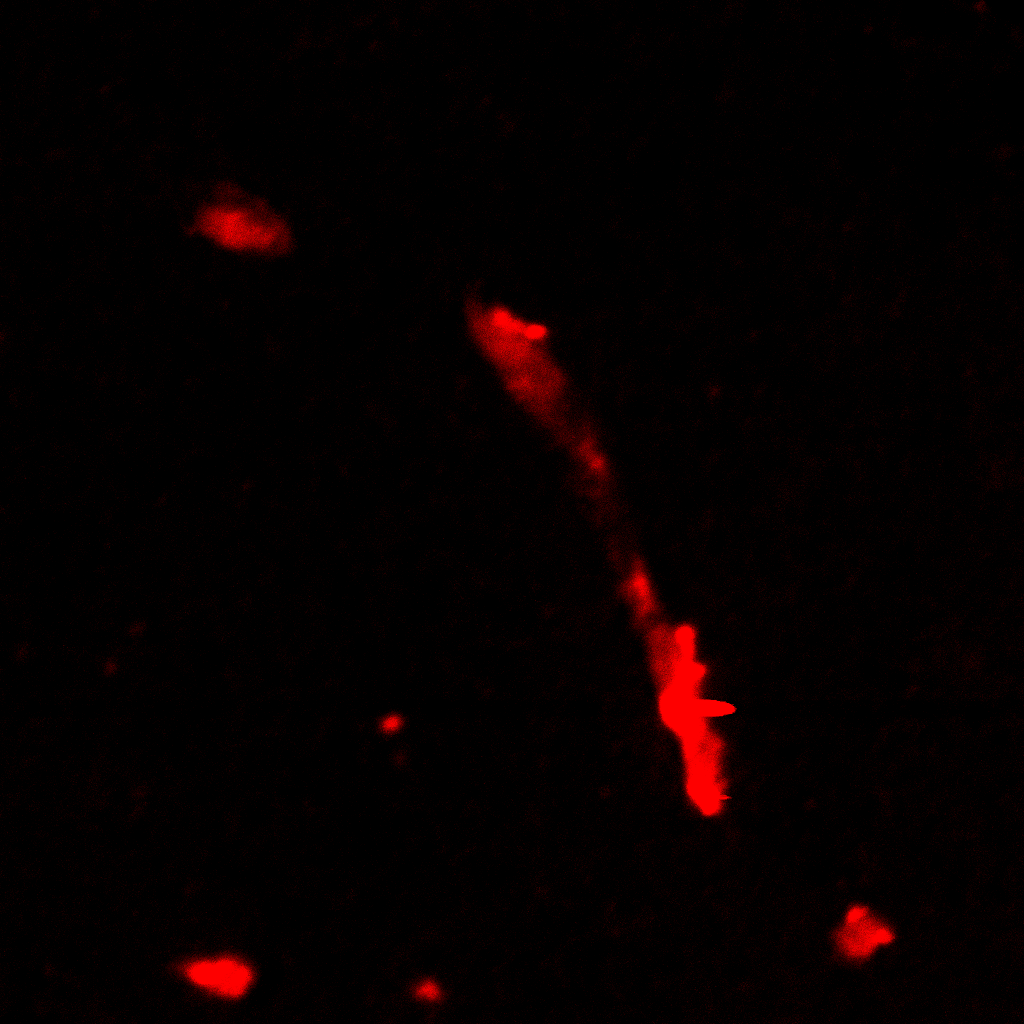

Supplement: Supplementary file 12 [file DataSheet6.ZIP › Immunofluorescence (Figure 6G, part1)/4/2/4s_c1.tif]

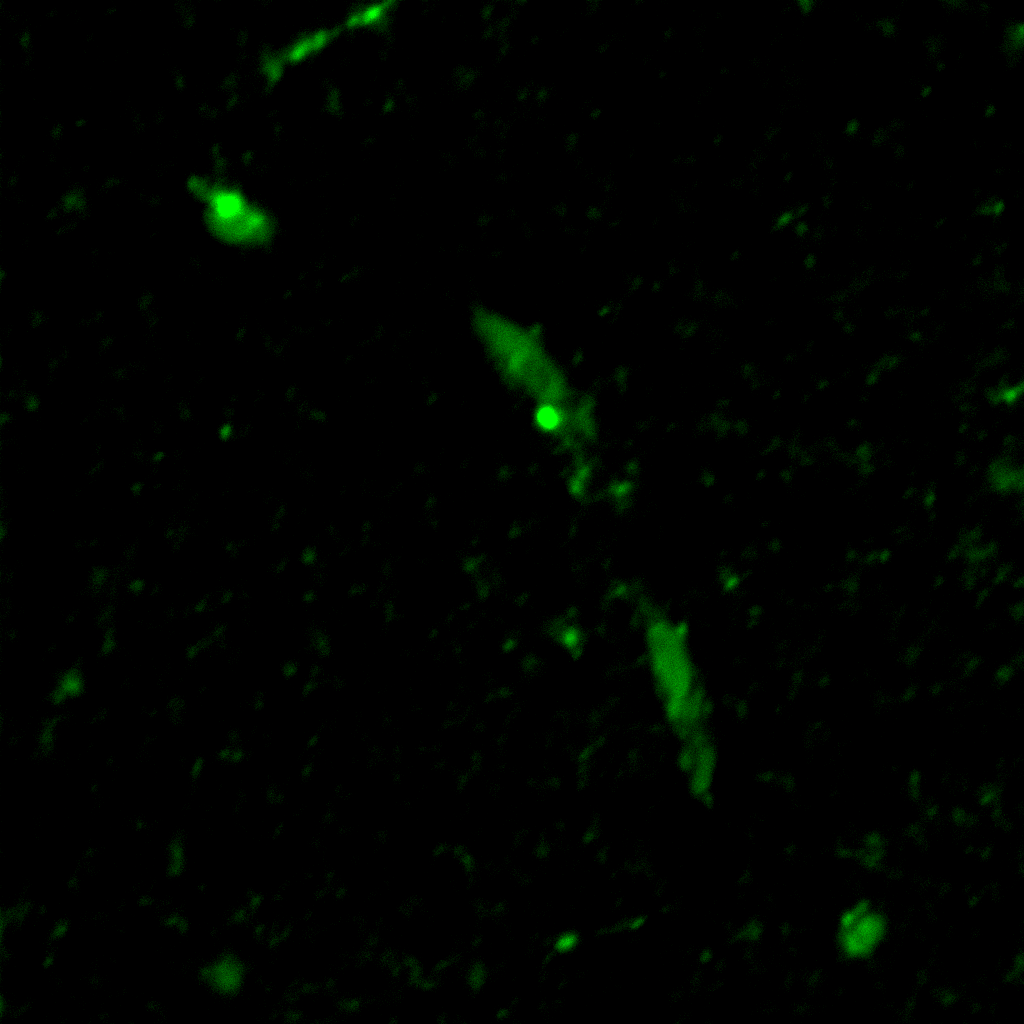

Supplement: Supplementary file 12 [file DataSheet6.ZIP › Immunofluorescence (Figure 6G, part1)/4/2/4s_c2.tif]

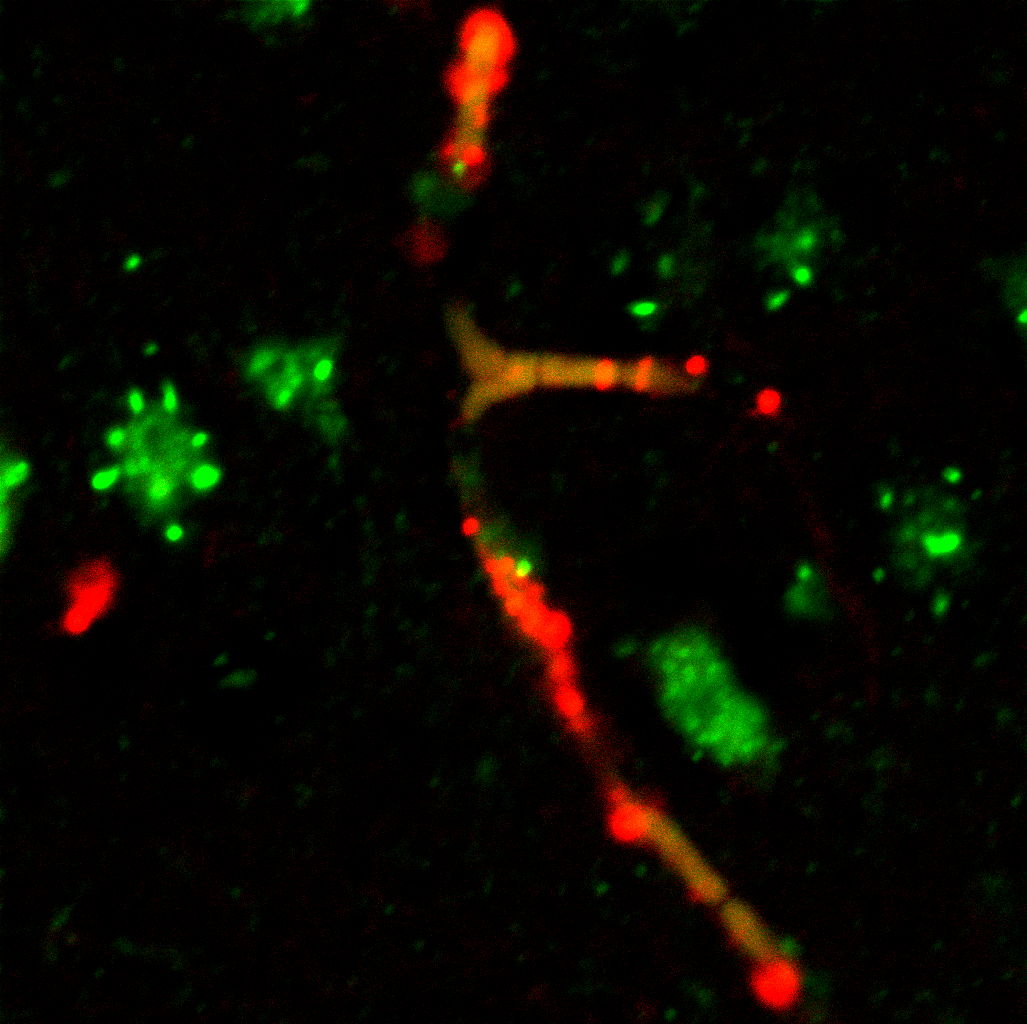

Supplement: Supplementary file 12 [file DataSheet6.ZIP › Immunofluorescence (Figure 6G, part1)/4/3/6s_c1+2.tif]

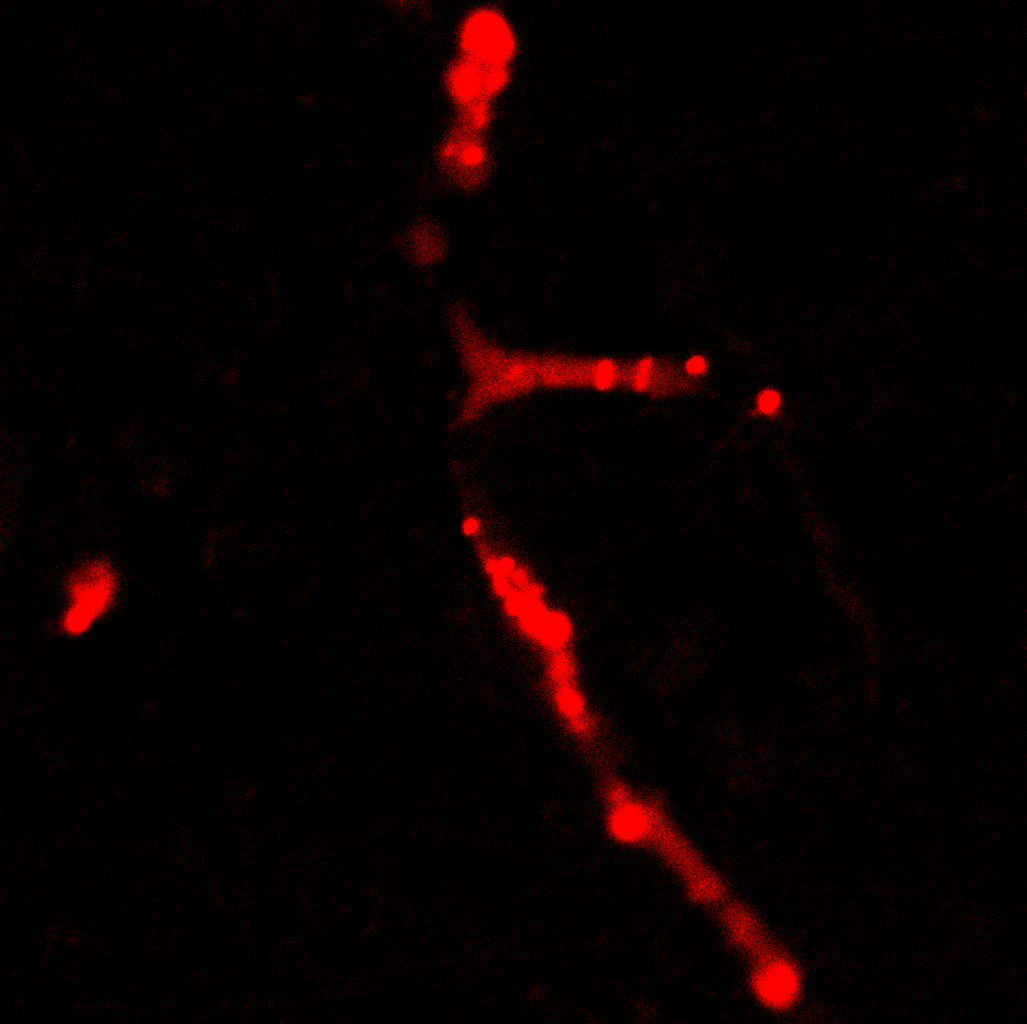

Supplement: Supplementary file 12 [file DataSheet6.ZIP › Immunofluorescence (Figure 6G, part1)/4/3/6s_c1.tif]

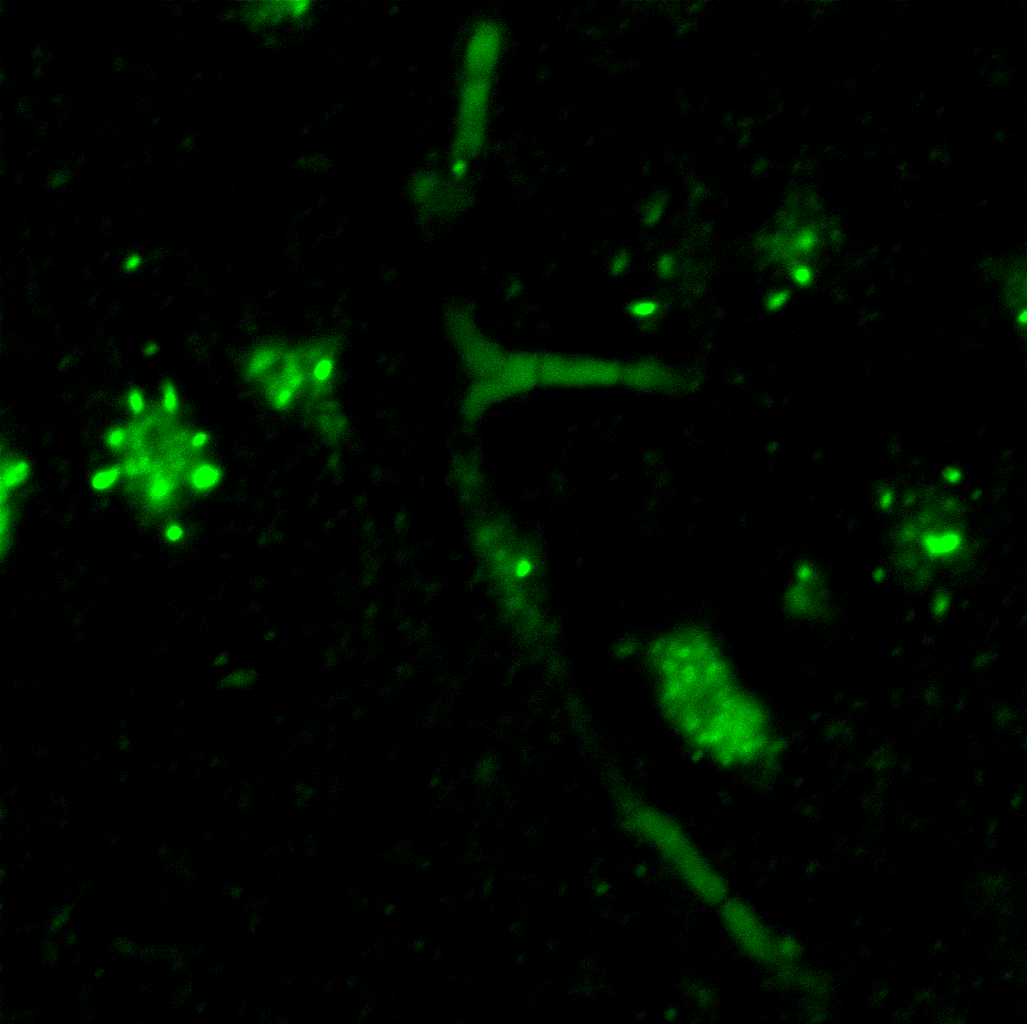

Supplement: Supplementary file 12 [file DataSheet6.ZIP › Immunofluorescence (Figure 6G, part1)/4/3/6s_c2.tif]

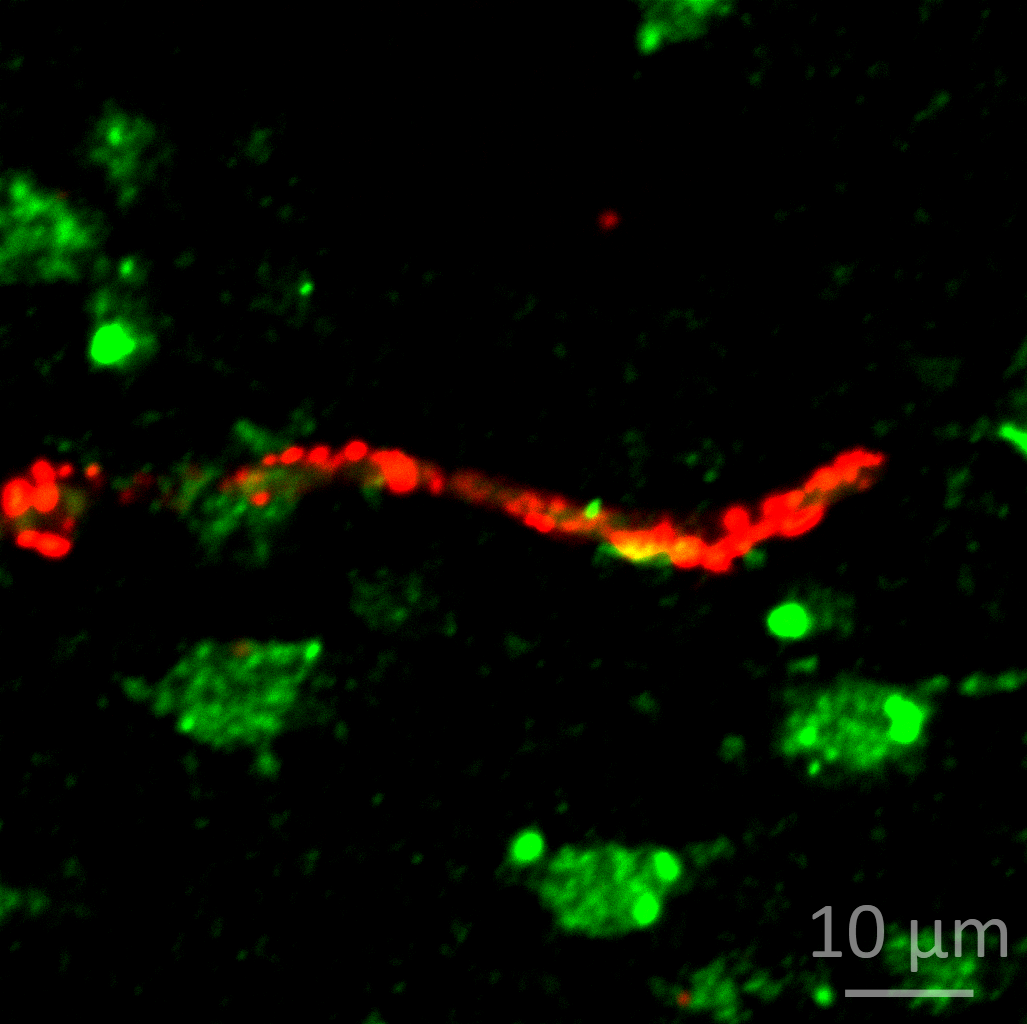

Supplement: Supplementary file 12 [file DataSheet6.ZIP › Immunofluorescence (Figure 6G, part1)/5 (presented in manuscript)/1 (presented in manuscript)/1S_c1+2.tif]

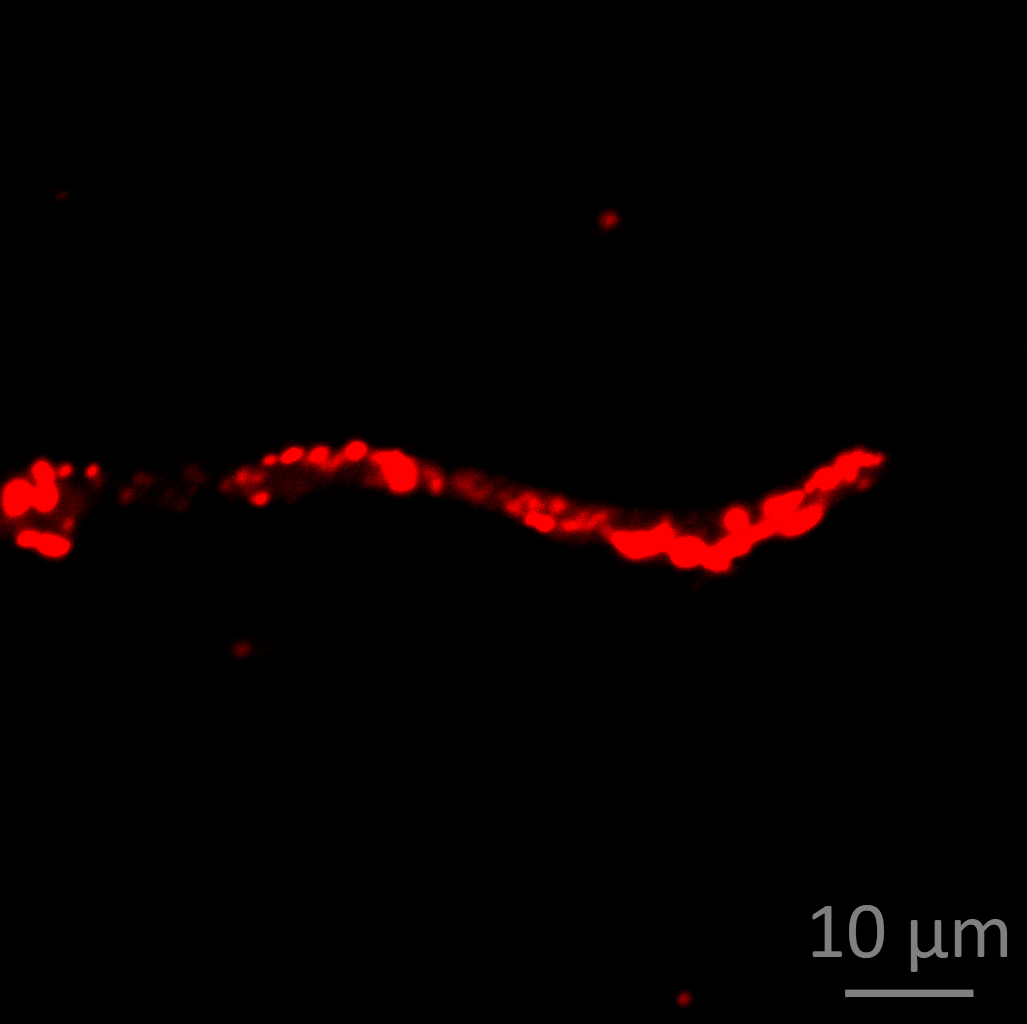

Supplement: Supplementary file 12 [file DataSheet6.ZIP › Immunofluorescence (Figure 6G, part1)/5 (presented in manuscript)/1 (presented in manuscript)/1S_c1.tif]

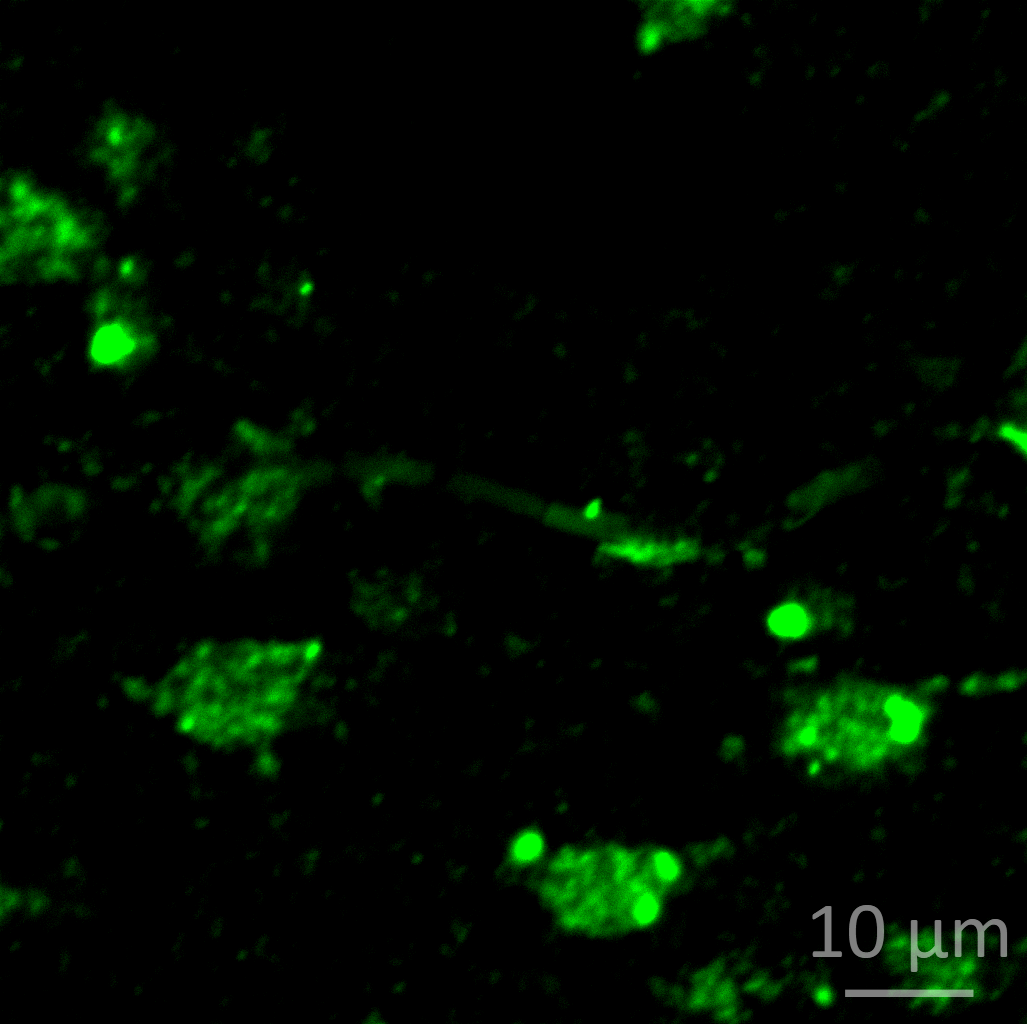

Supplement: Supplementary file 12 [file DataSheet6.ZIP › Immunofluorescence (Figure 6G, part1)/5 (presented in manuscript)/1 (presented in manuscript)/1S_c2.tif]

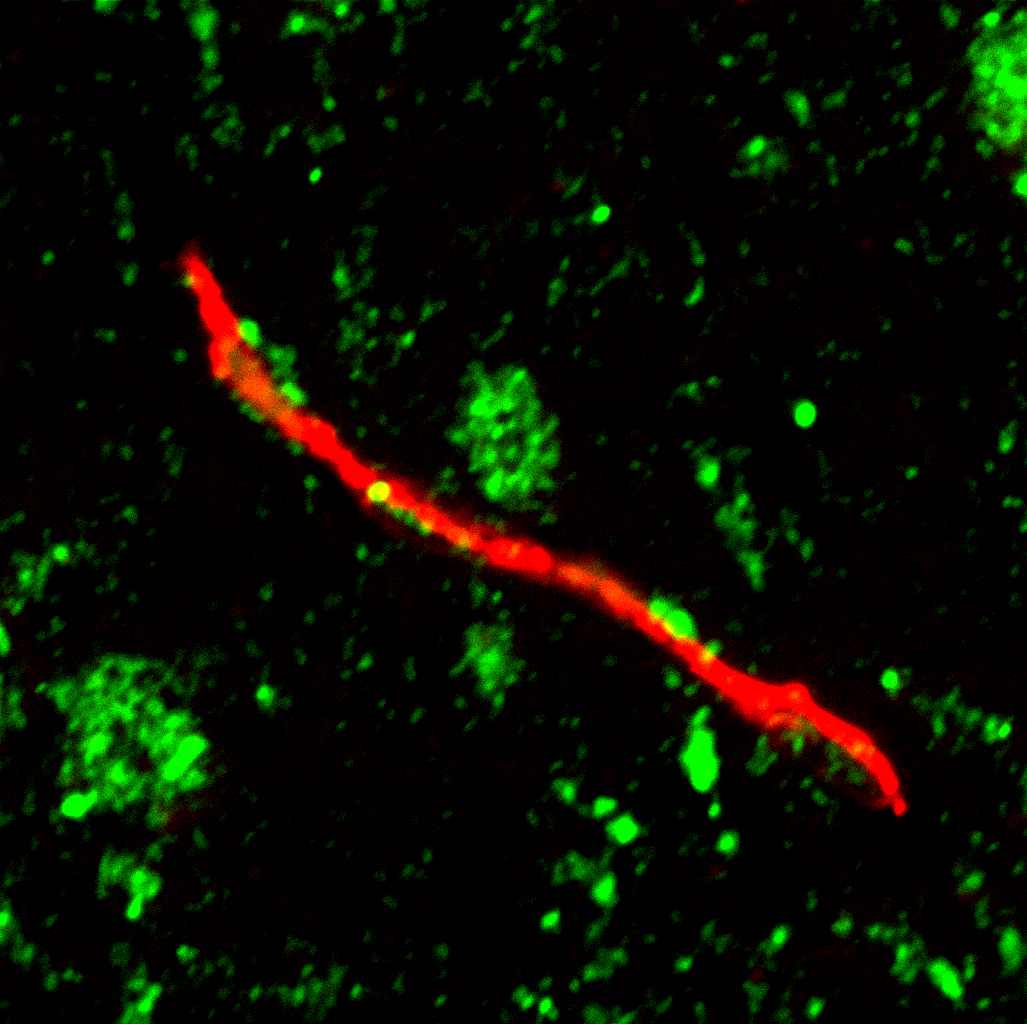

Supplement: Supplementary file 12 [file DataSheet6.ZIP › Immunofluorescence (Figure 6G, part1)/5 (presented in manuscript)/2/4S_c1+2.tif]

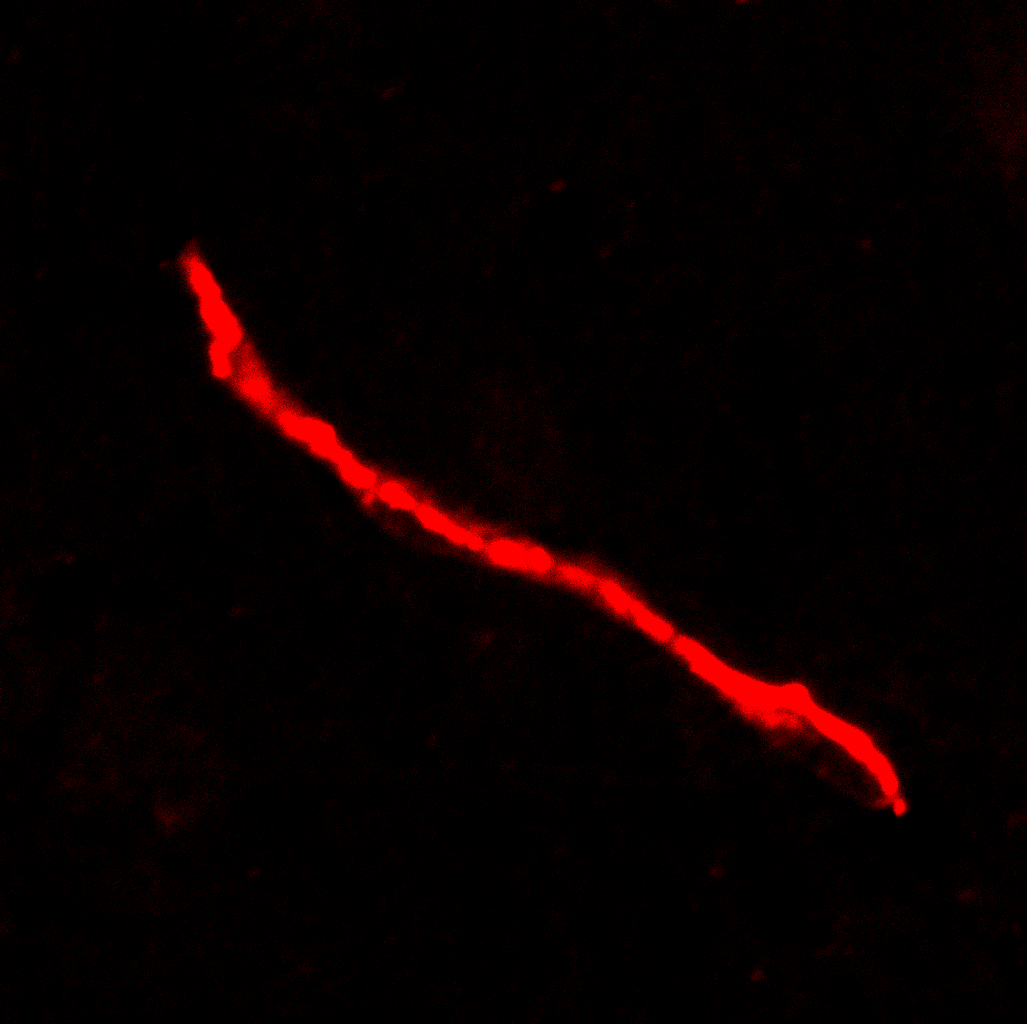

Supplement: Supplementary file 12 [file DataSheet6.ZIP › Immunofluorescence (Figure 6G, part1)/5 (presented in manuscript)/2/4S_c1.tif]

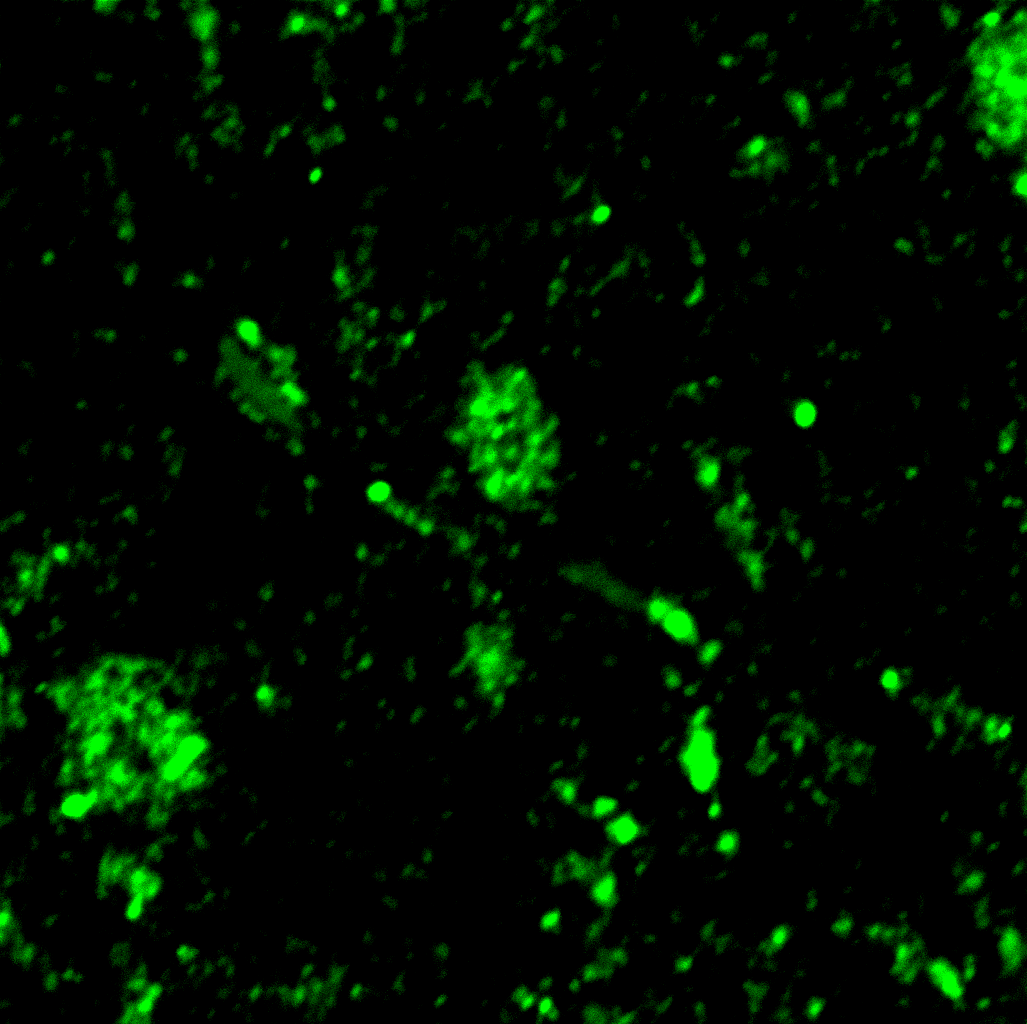

Supplement: Supplementary file 12 [file DataSheet6.ZIP › Immunofluorescence (Figure 6G, part1)/5 (presented in manuscript)/2/4S_c2.tif]

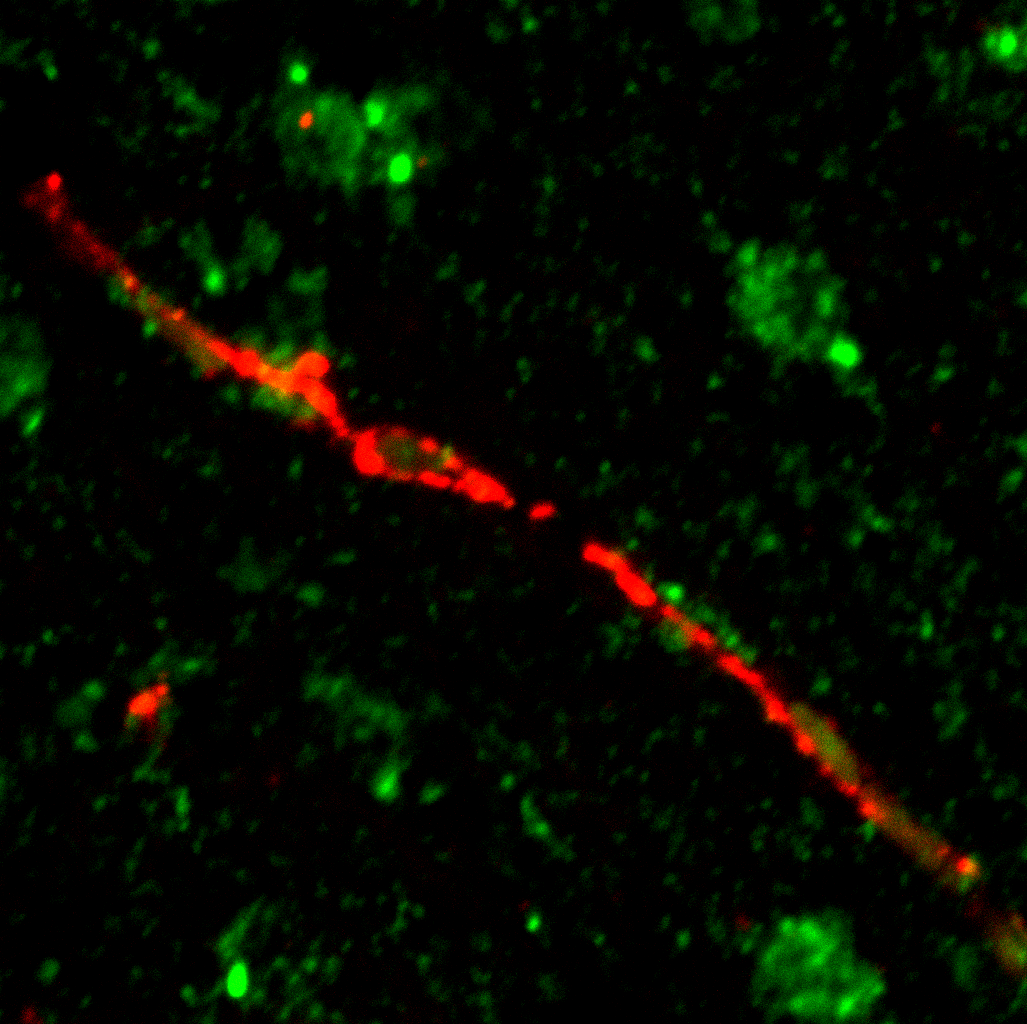

Supplement: Supplementary file 12 [file DataSheet6.ZIP › Immunofluorescence (Figure 6G, part1)/5 (presented in manuscript)/3/5S_c1+2.tif]

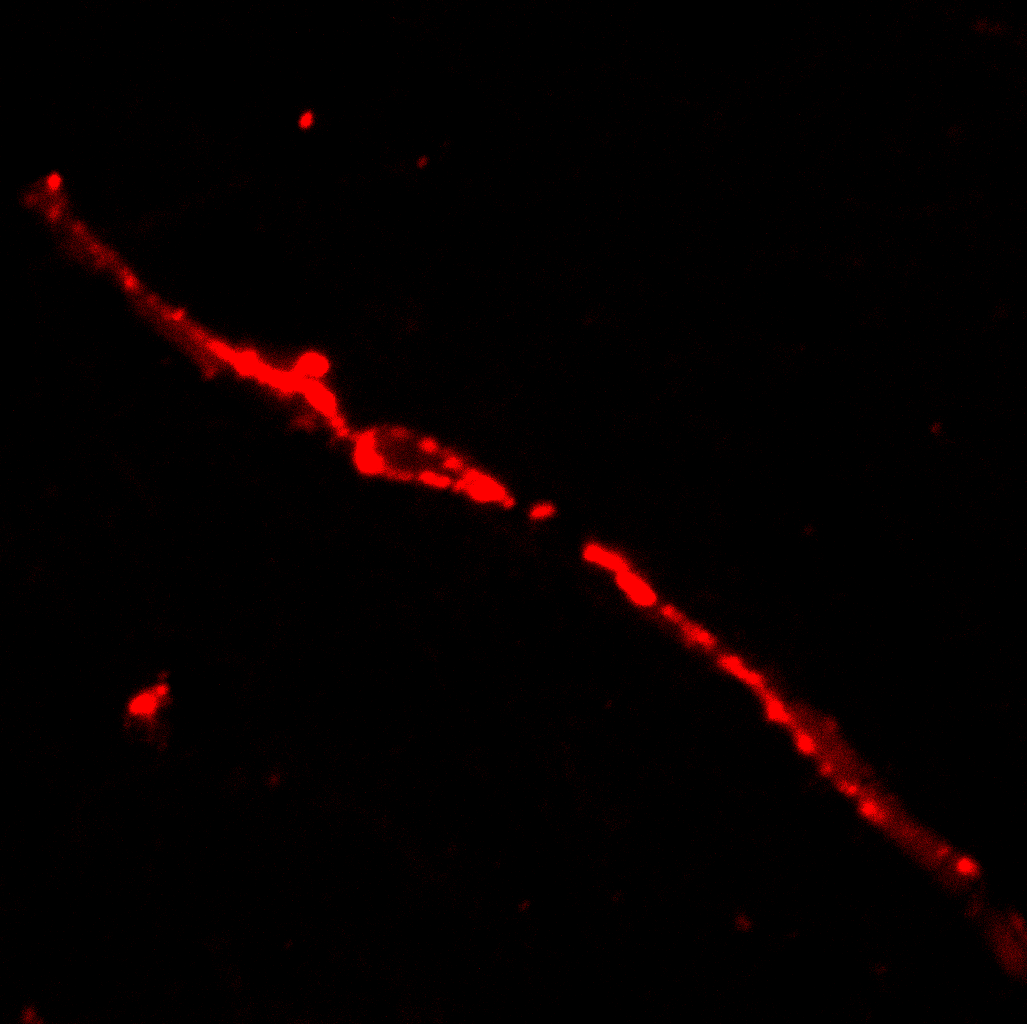

Supplement: Supplementary file 12 [file DataSheet6.ZIP › Immunofluorescence (Figure 6G, part1)/5 (presented in manuscript)/3/5S_c1.tif]

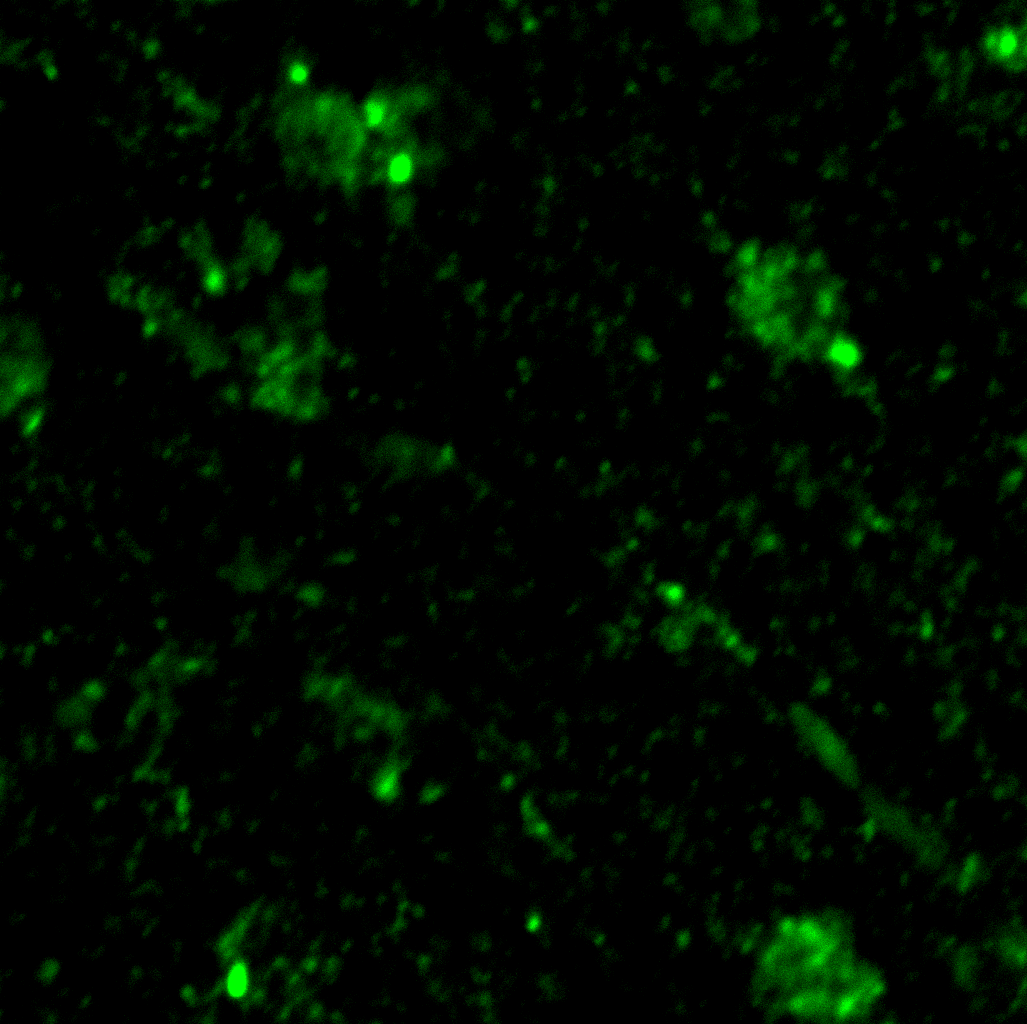

Supplement: Supplementary file 12 [file DataSheet6.ZIP › Immunofluorescence (Figure 6G, part1)/5 (presented in manuscript)/3/5S_c2.tif]

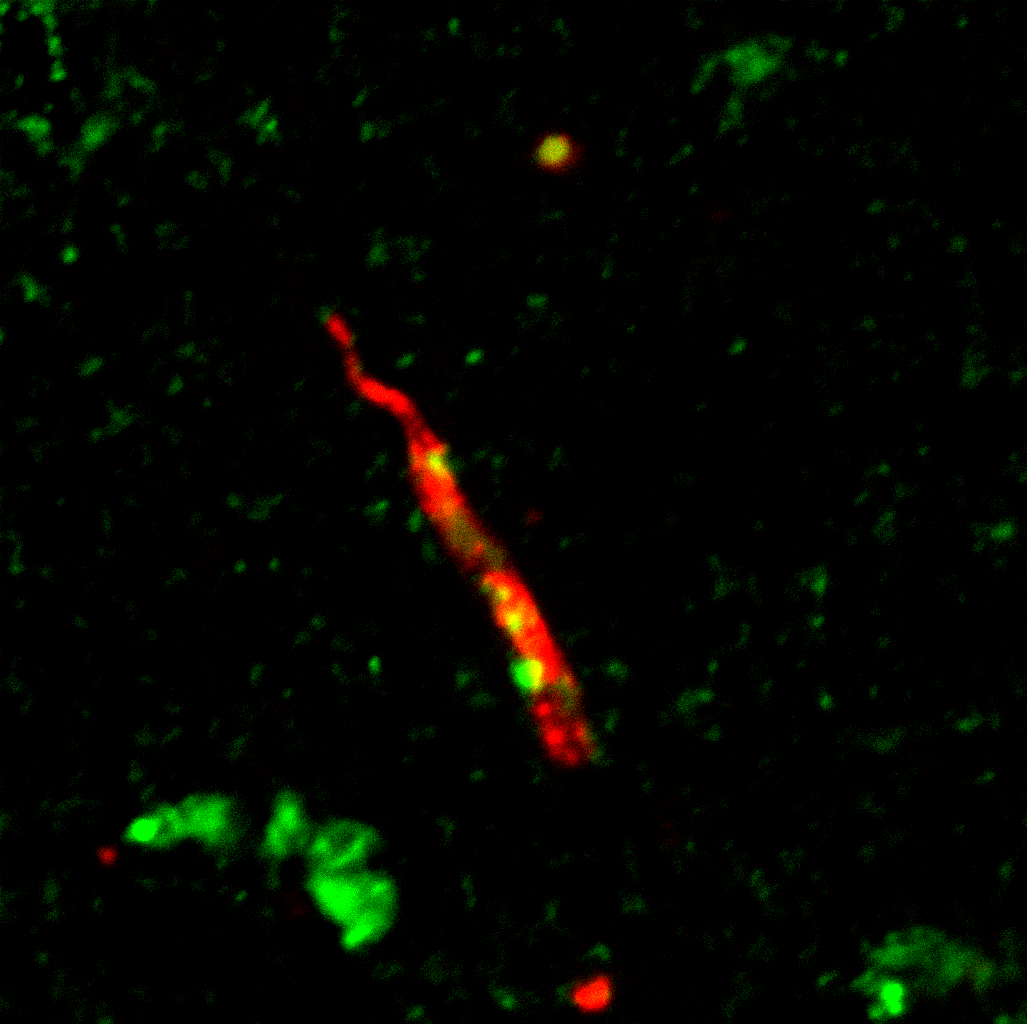

Supplement: Supplementary file 12 [file DataSheet6.ZIP › Immunofluorescence (Figure 6G, part1)/6/1/1S_c1+2.tif]

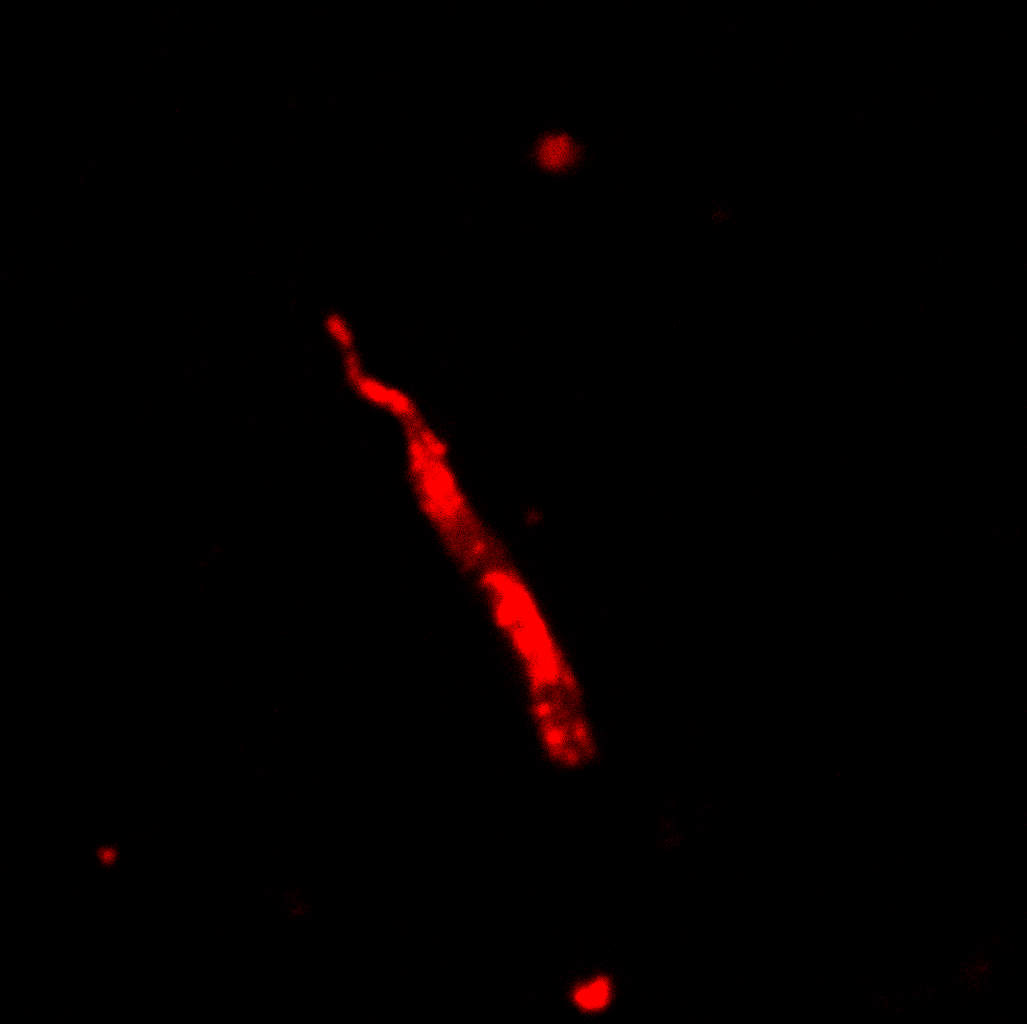

Supplement: Supplementary file 12 [file DataSheet6.ZIP › Immunofluorescence (Figure 6G, part1)/6/1/1S_c1.tif]

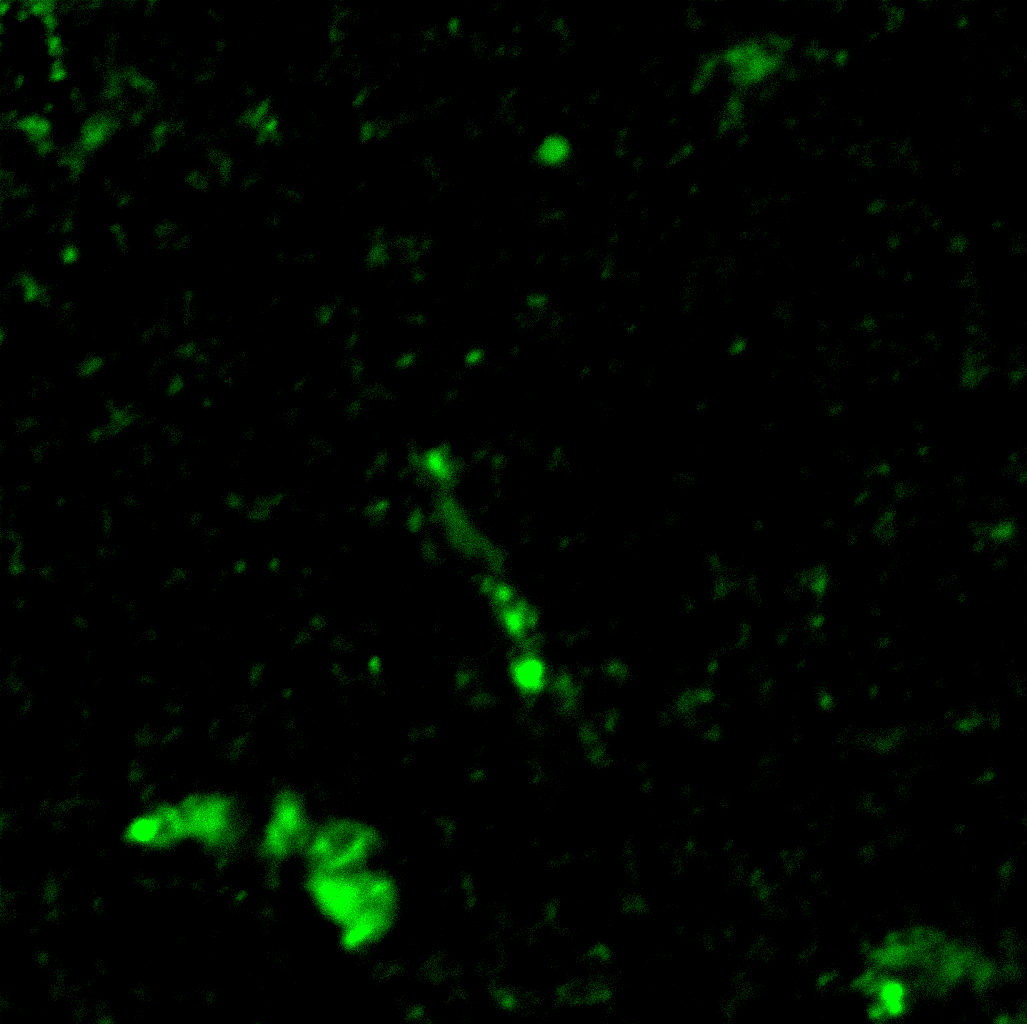

Supplement: Supplementary file 12 [file DataSheet6.ZIP › Immunofluorescence (Figure 6G, part1)/6/1/1S_c2.tif]

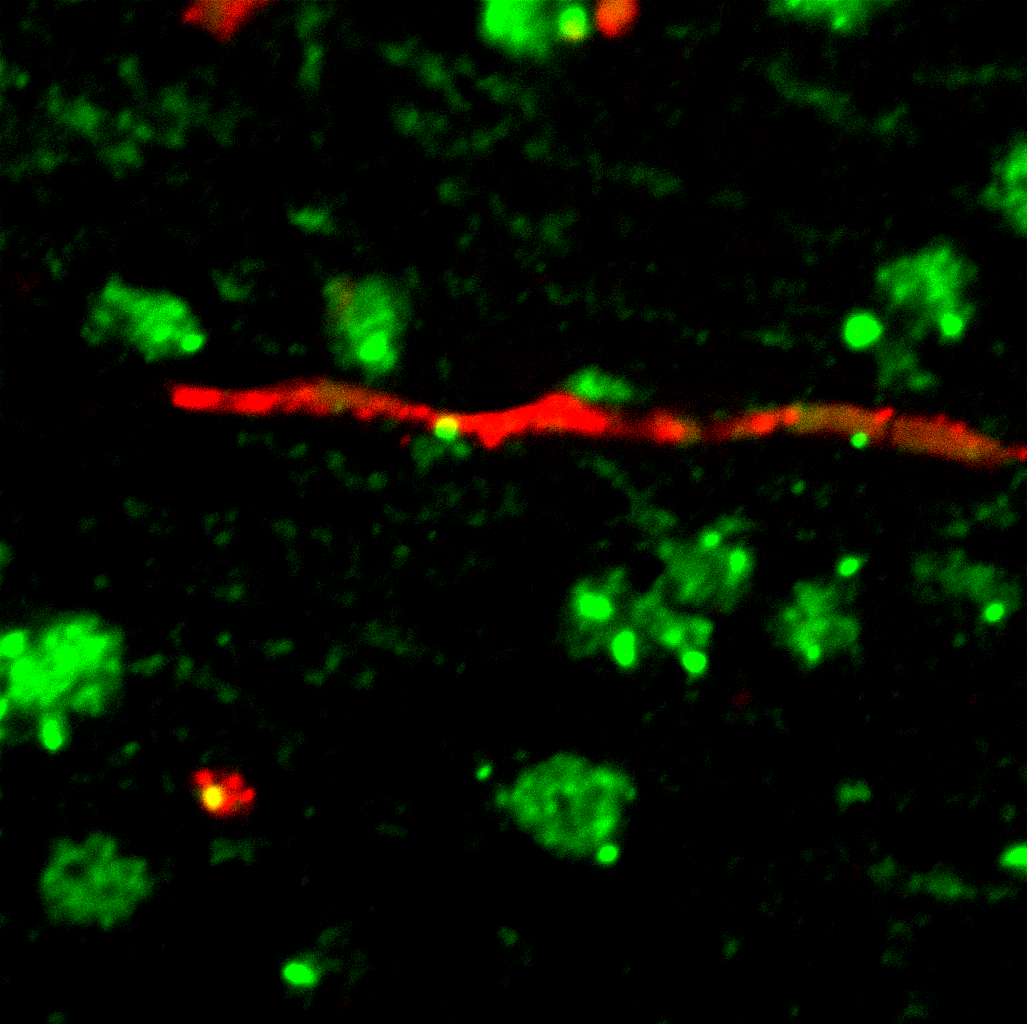

Supplement: Supplementary file 12 [file DataSheet6.ZIP › Immunofluorescence (Figure 6G, part1)/6/2/2S_c1+2.tif]

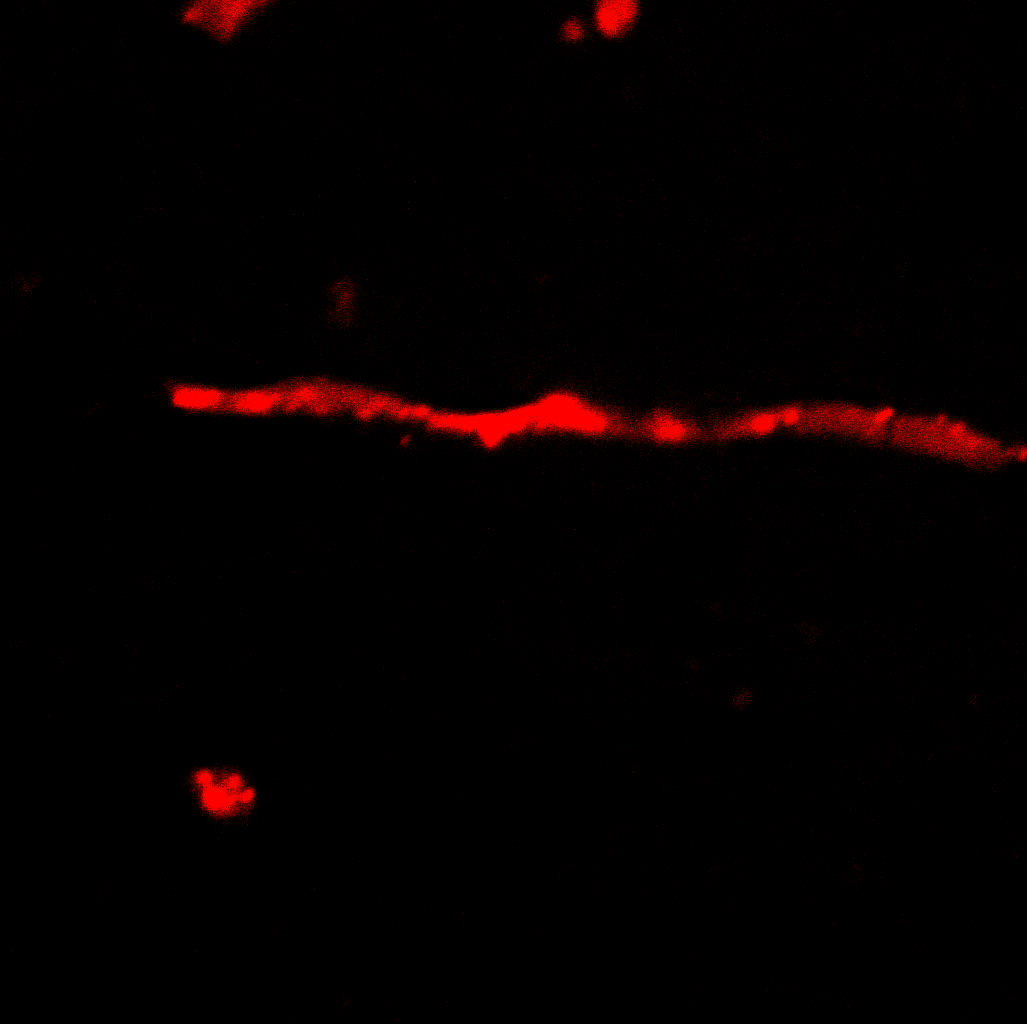

Supplement: Supplementary file 12 [file DataSheet6.ZIP › Immunofluorescence (Figure 6G, part1)/6/2/2S_c1.tif]

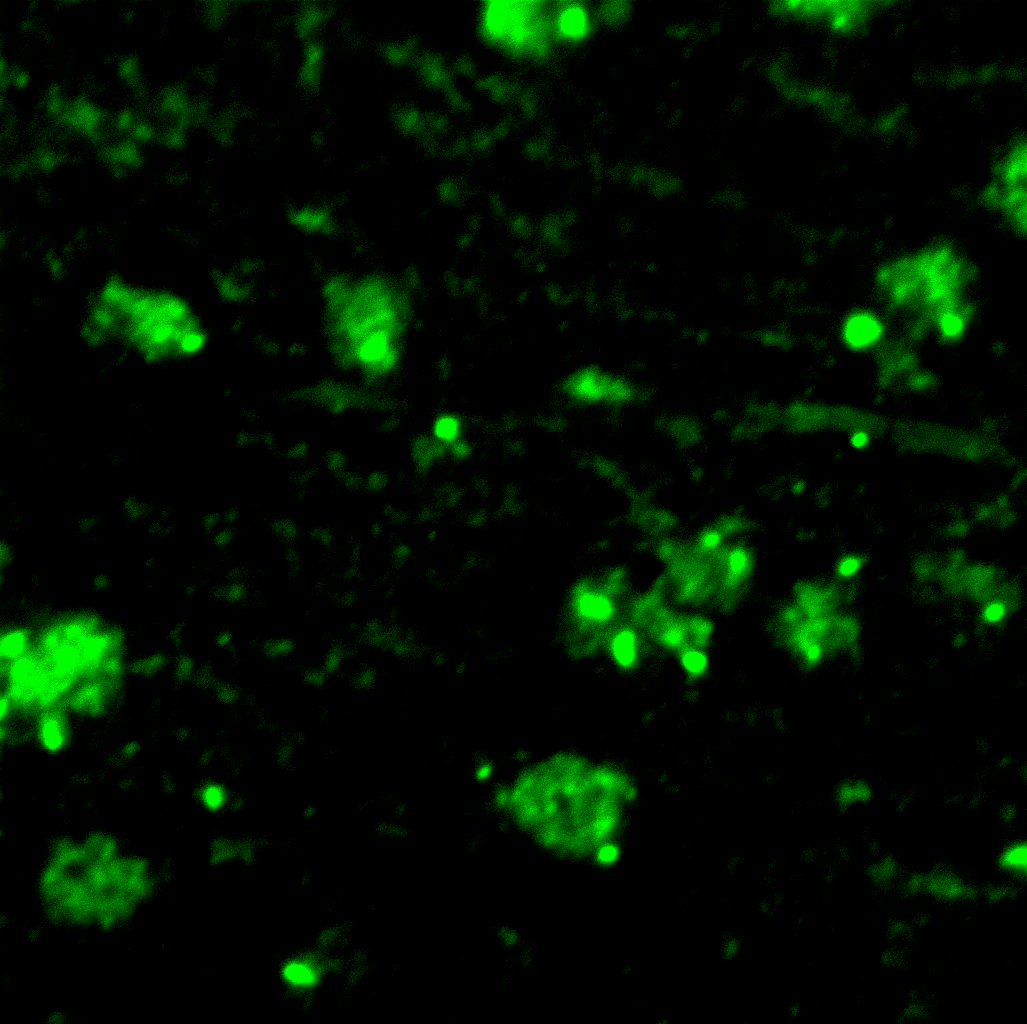

Supplement: Supplementary file 12 [file DataSheet6.ZIP › Immunofluorescence (Figure 6G, part1)/6/2/2S_c2.tif]

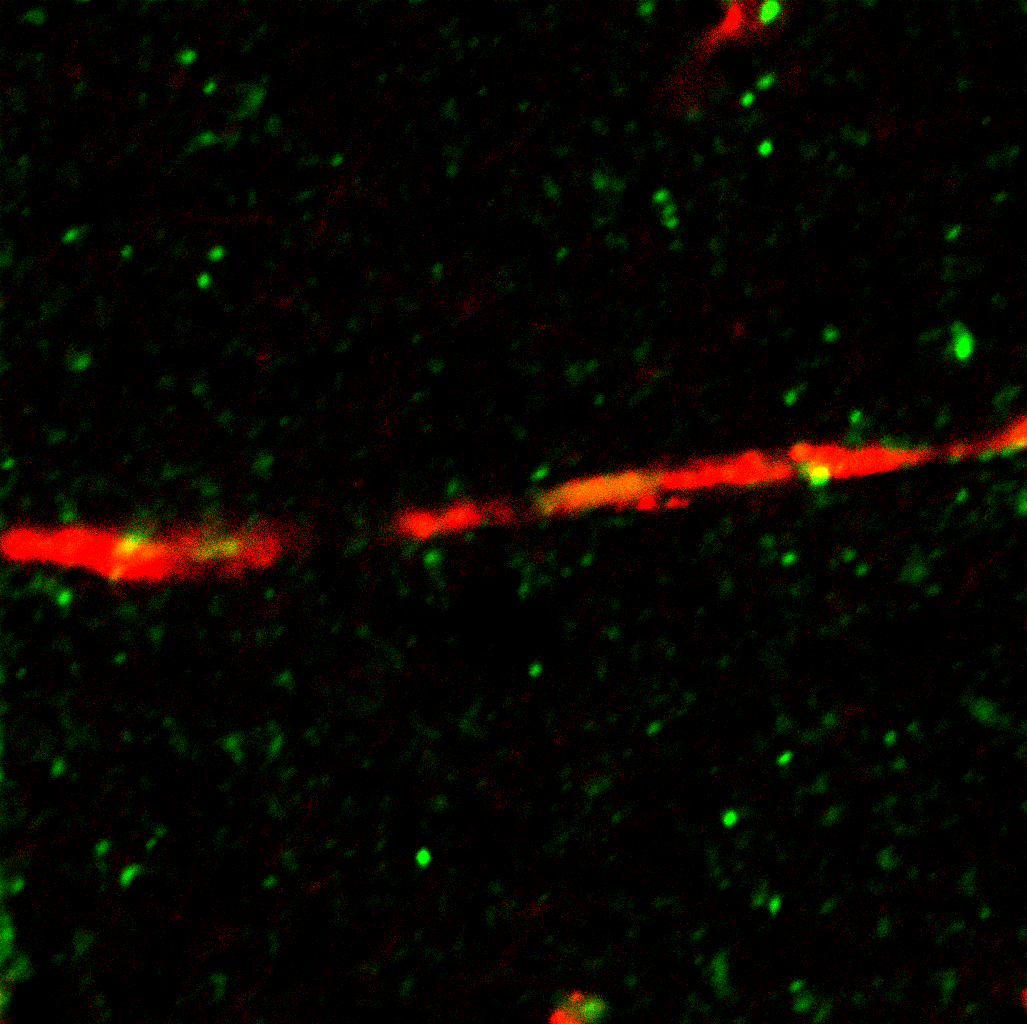

Supplement: Supplementary file 12 [file DataSheet6.ZIP › Immunofluorescence (Figure 6G, part1)/6/3/7S_c1+2.tif]

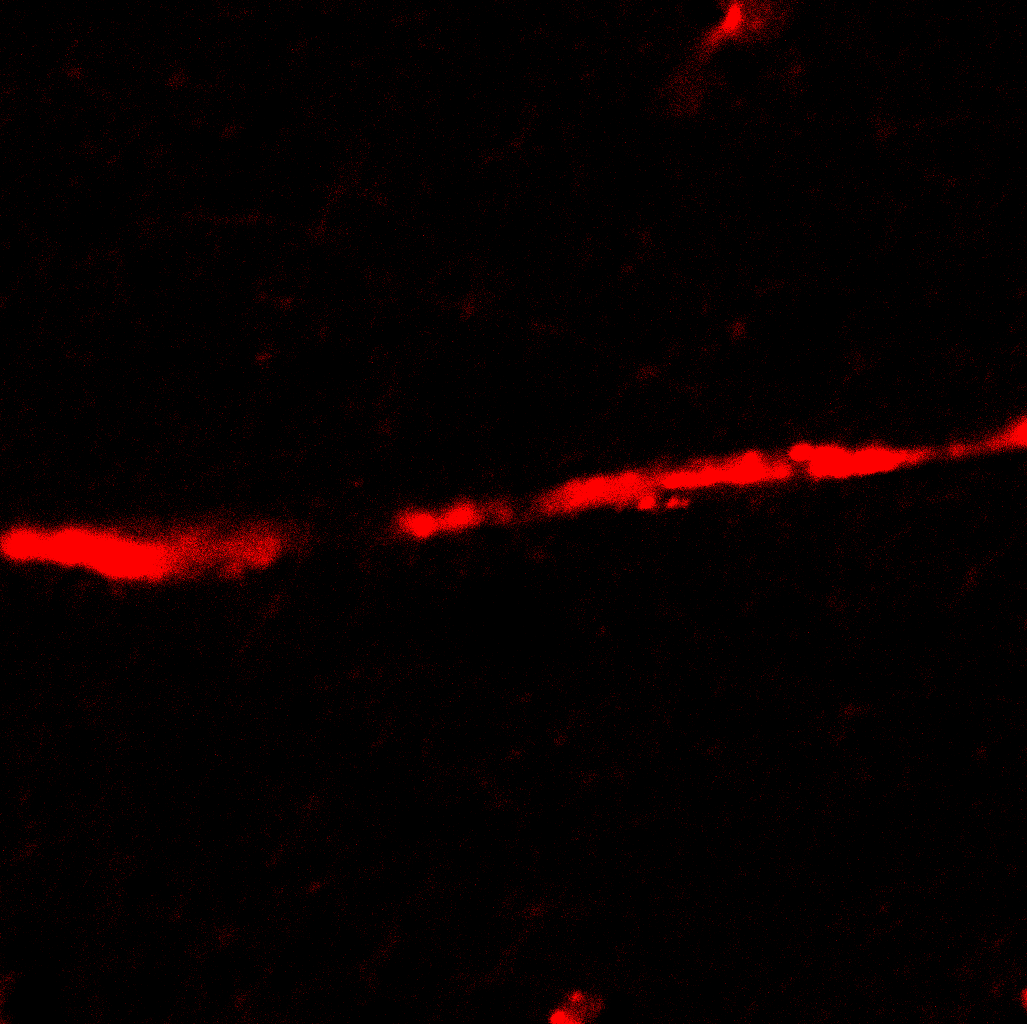

Supplement: Supplementary file 12 [file DataSheet6.ZIP › Immunofluorescence (Figure 6G, part1)/6/3/7S_c1.tif]

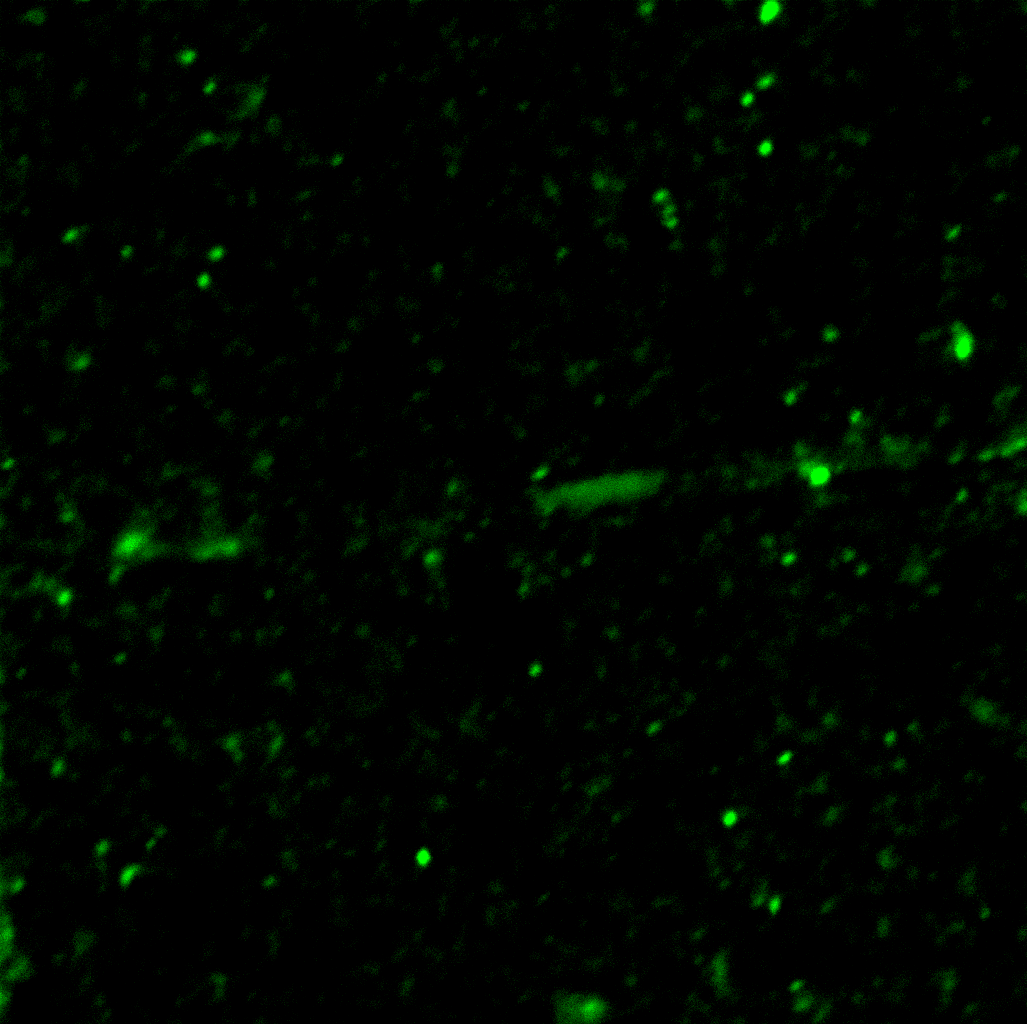

Supplement: Supplementary file 12 [file DataSheet6.ZIP › Immunofluorescence (Figure 6G, part1)/6/3/7S_c2.tif]

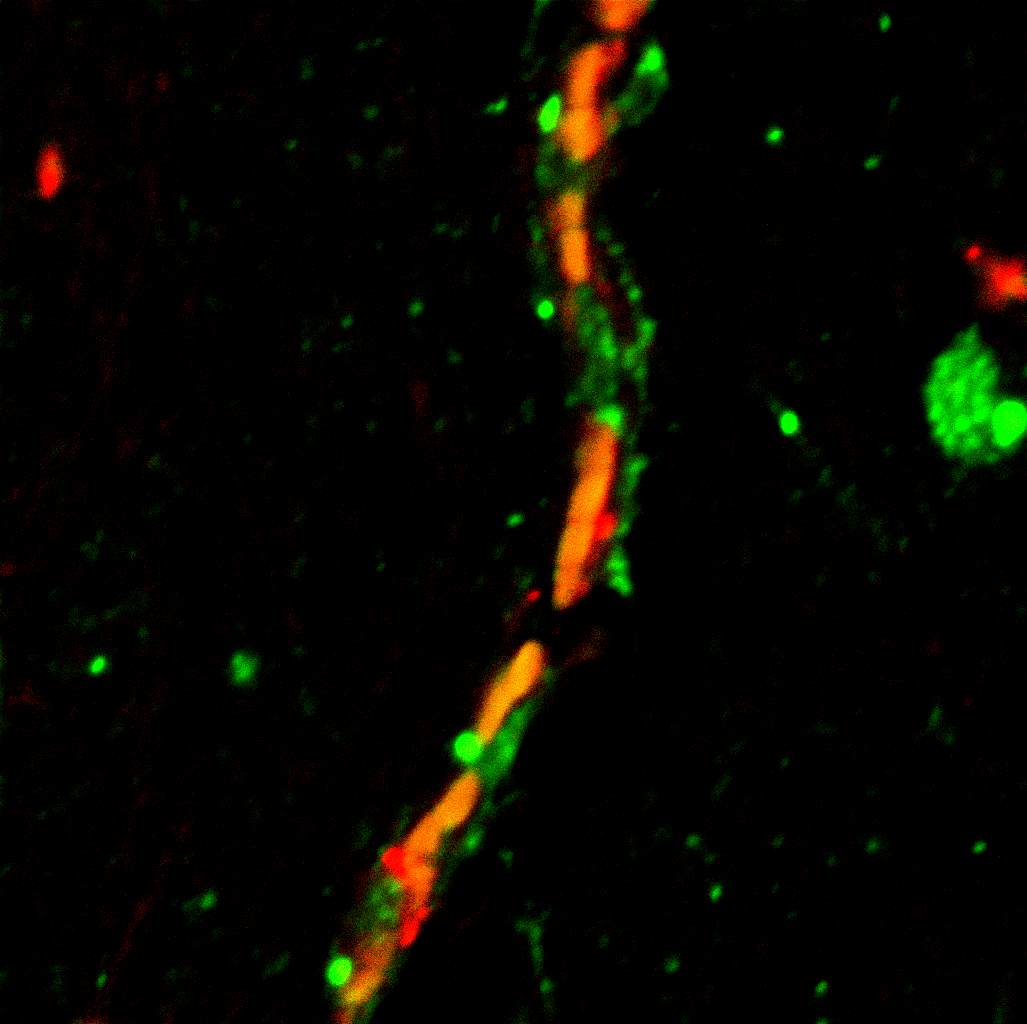

Supplement: Supplementary file 19 [file DataSheet7.ZIP › Immunofluorescence (Figure 6G, part2)/10 (presented in manuscript)/1 (presented in manuscript)/2S_c1+2.tif]

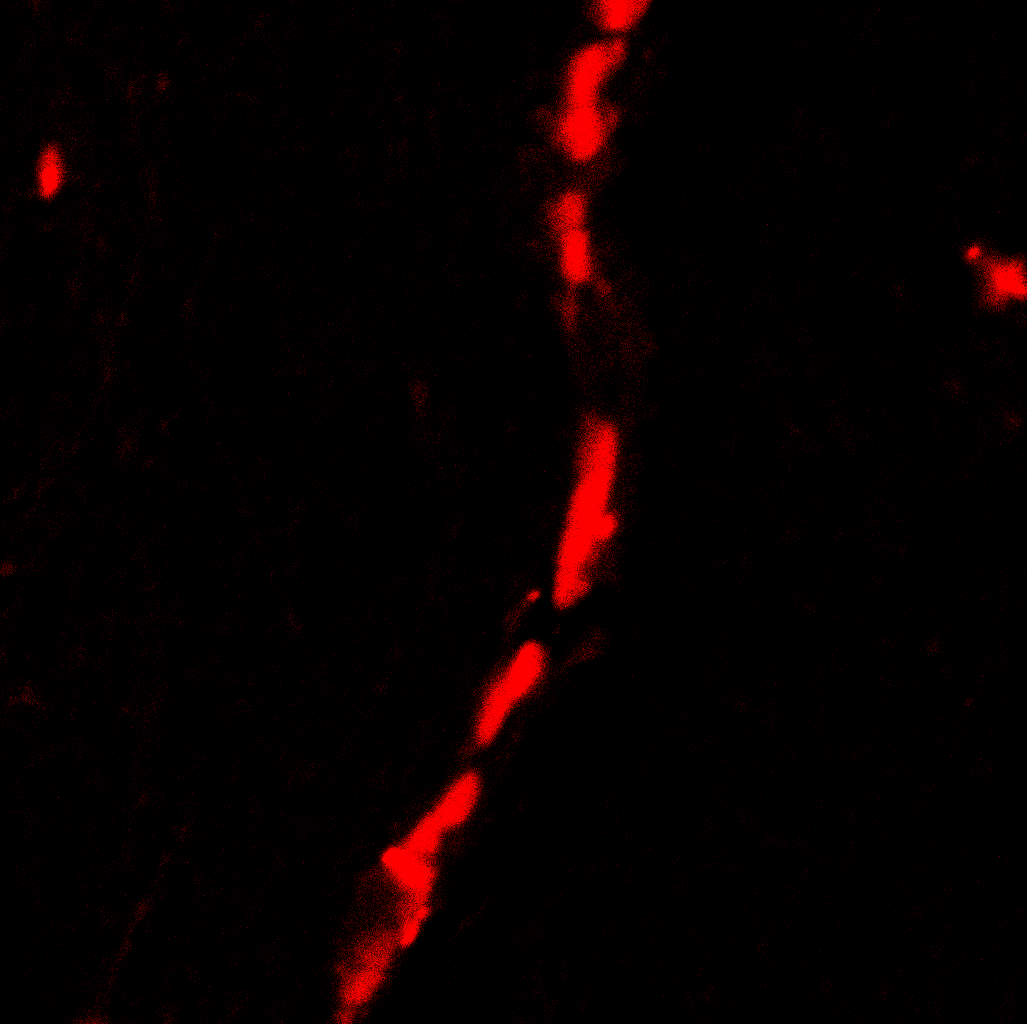

Supplement: Supplementary file 19 [file DataSheet7.ZIP › Immunofluorescence (Figure 6G, part2)/10 (presented in manuscript)/1 (presented in manuscript)/2S_c1.tif]

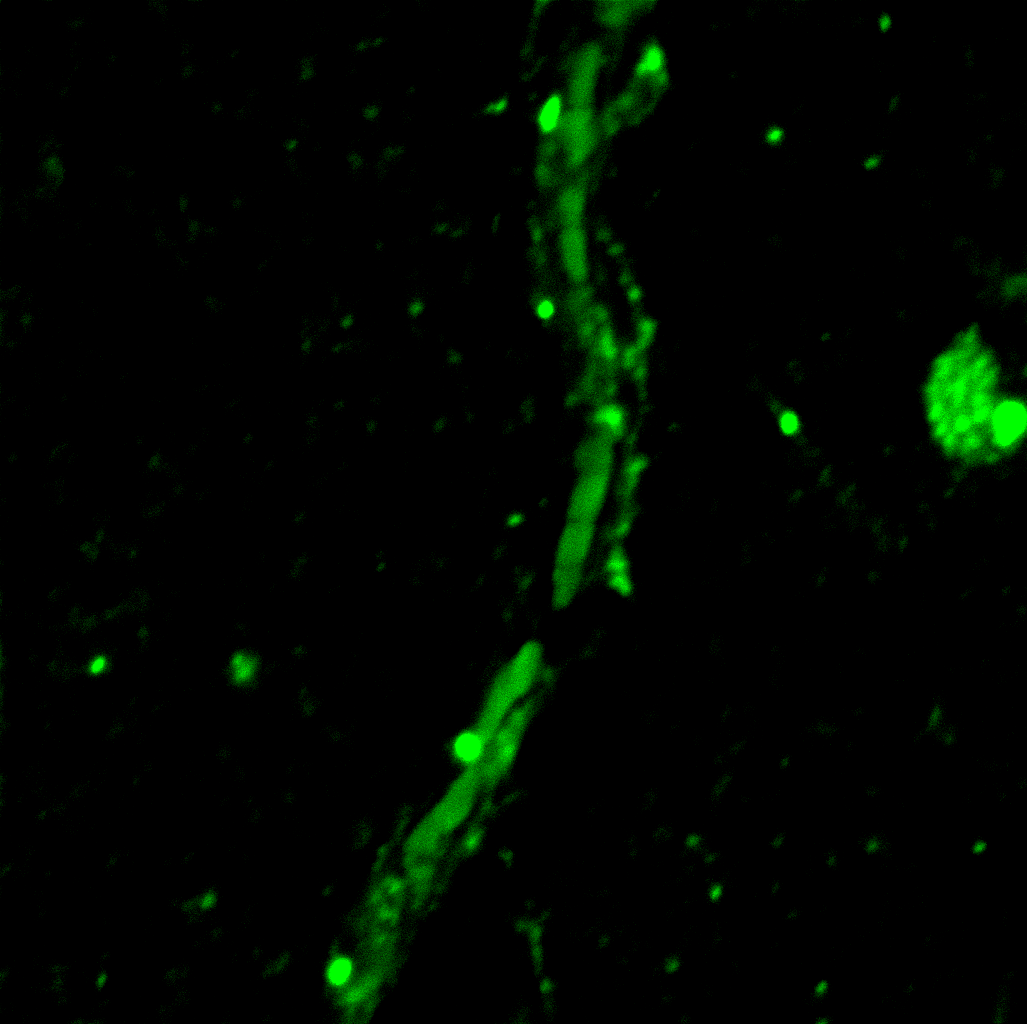

Supplement: Supplementary file 19 [file DataSheet7.ZIP › Immunofluorescence (Figure 6G, part2)/10 (presented in manuscript)/1 (presented in manuscript)/2S_c2.tif]

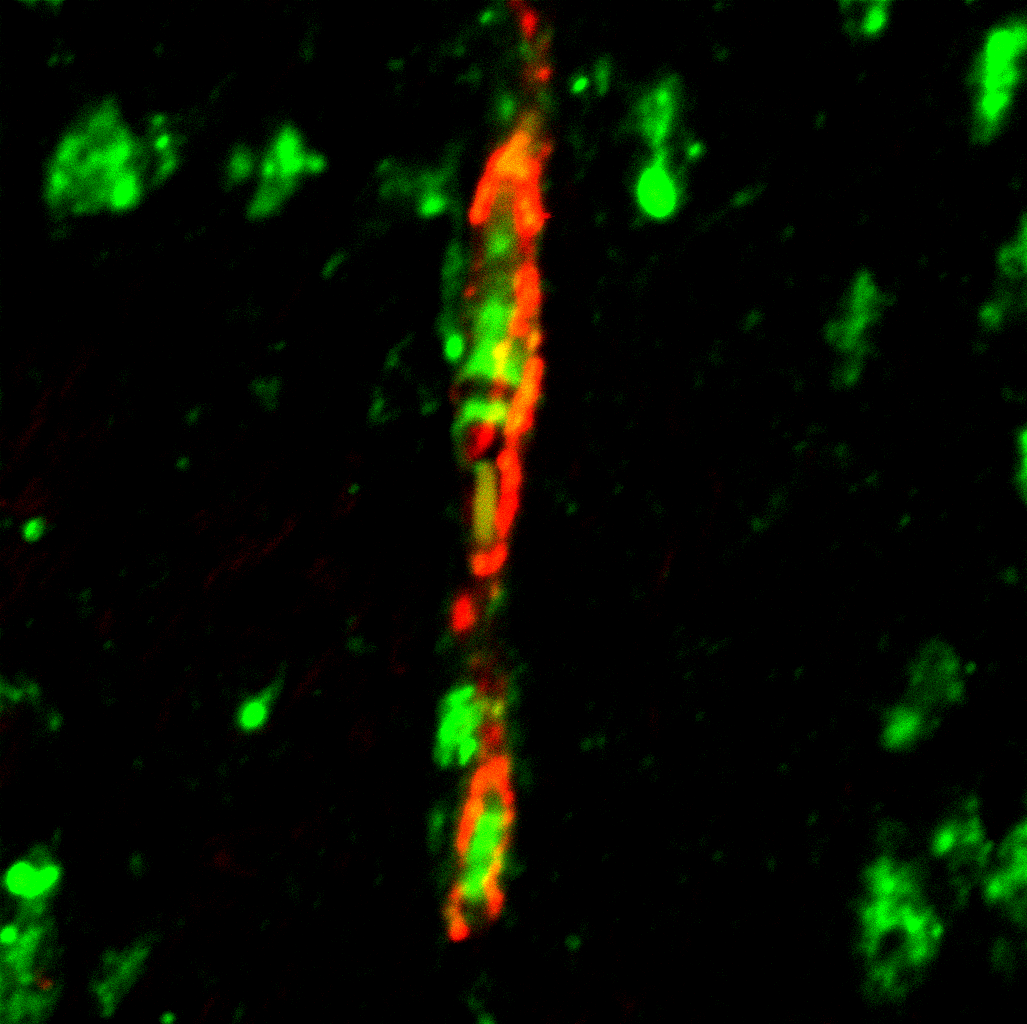

Supplement: Supplementary file 19 [file DataSheet7.ZIP › Immunofluorescence (Figure 6G, part2)/10 (presented in manuscript)/2/4S_c1+2.tif]

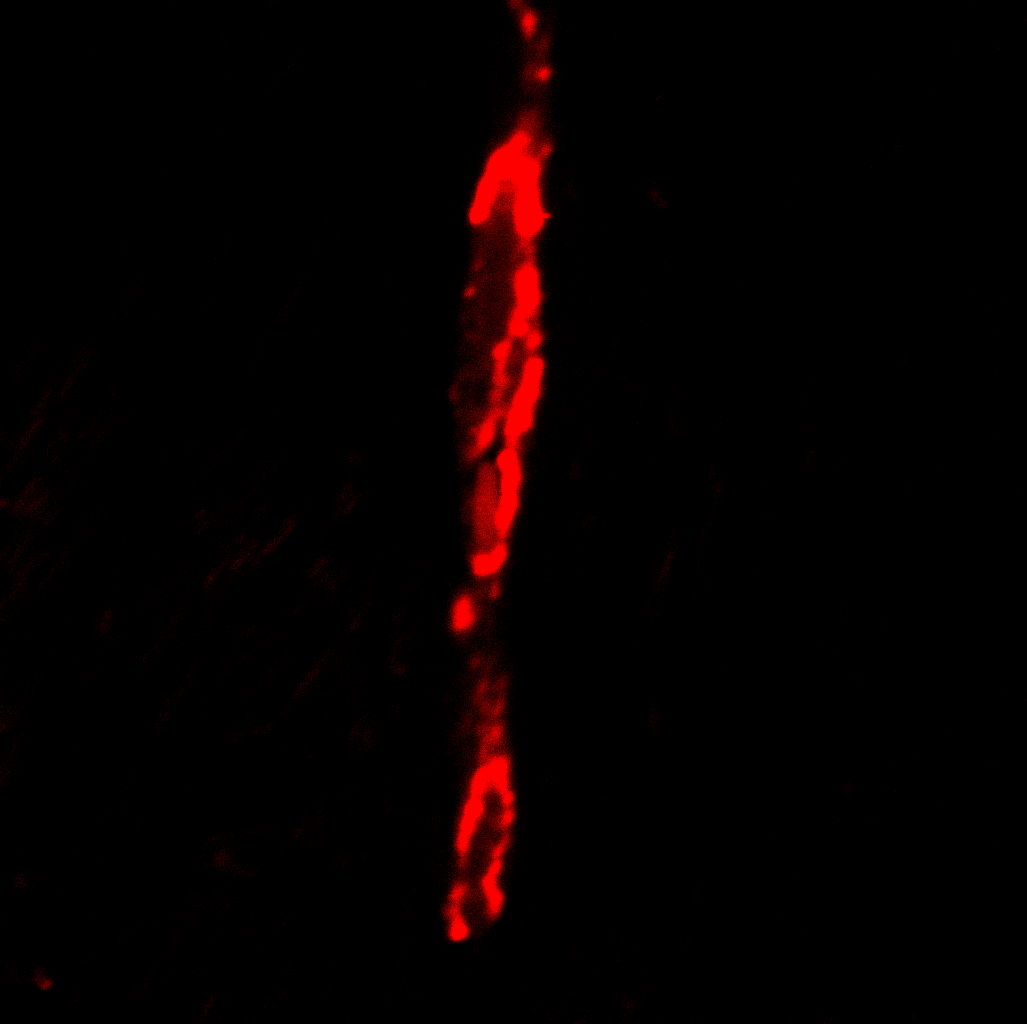

Supplement: Supplementary file 19 [file DataSheet7.ZIP › Immunofluorescence (Figure 6G, part2)/10 (presented in manuscript)/2/4S_c1.tif]

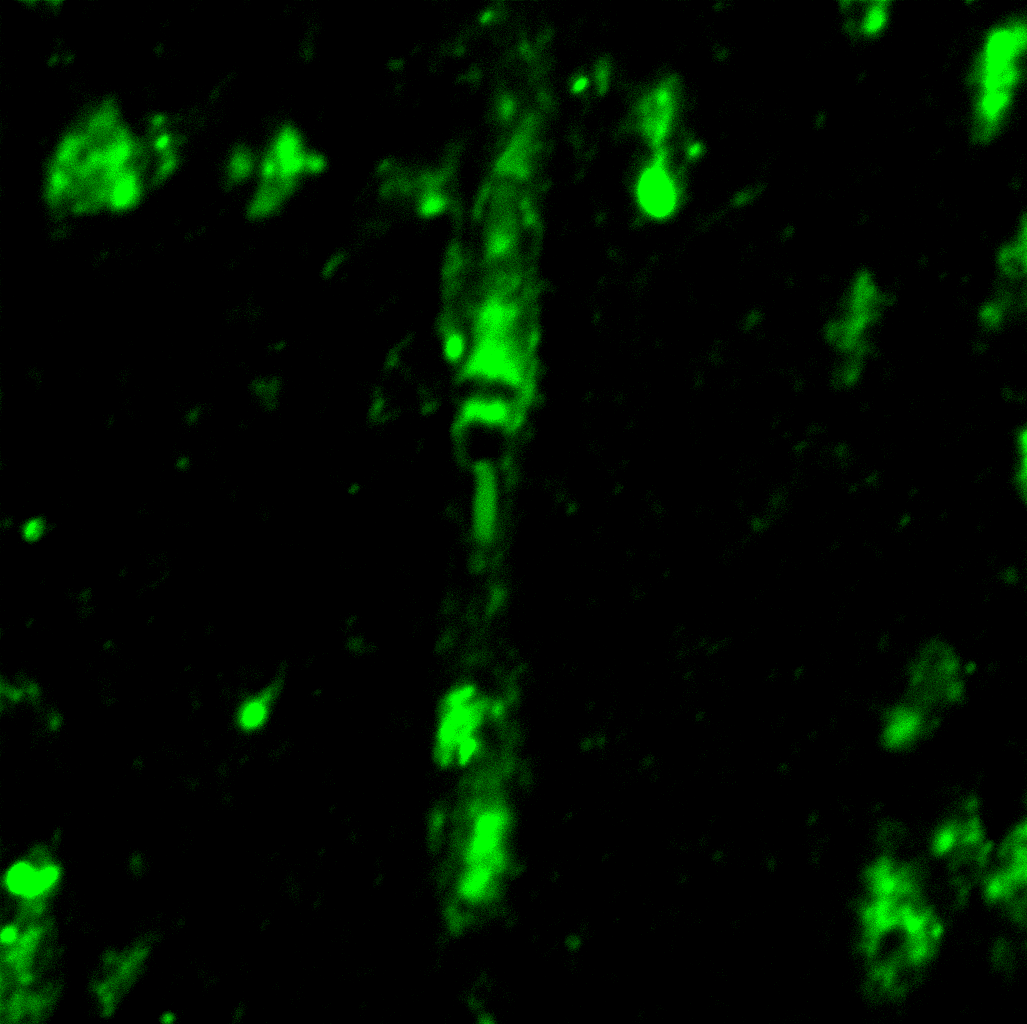

Supplement: Supplementary file 19 [file DataSheet7.ZIP › Immunofluorescence (Figure 6G, part2)/10 (presented in manuscript)/2/4S_c2.tif]

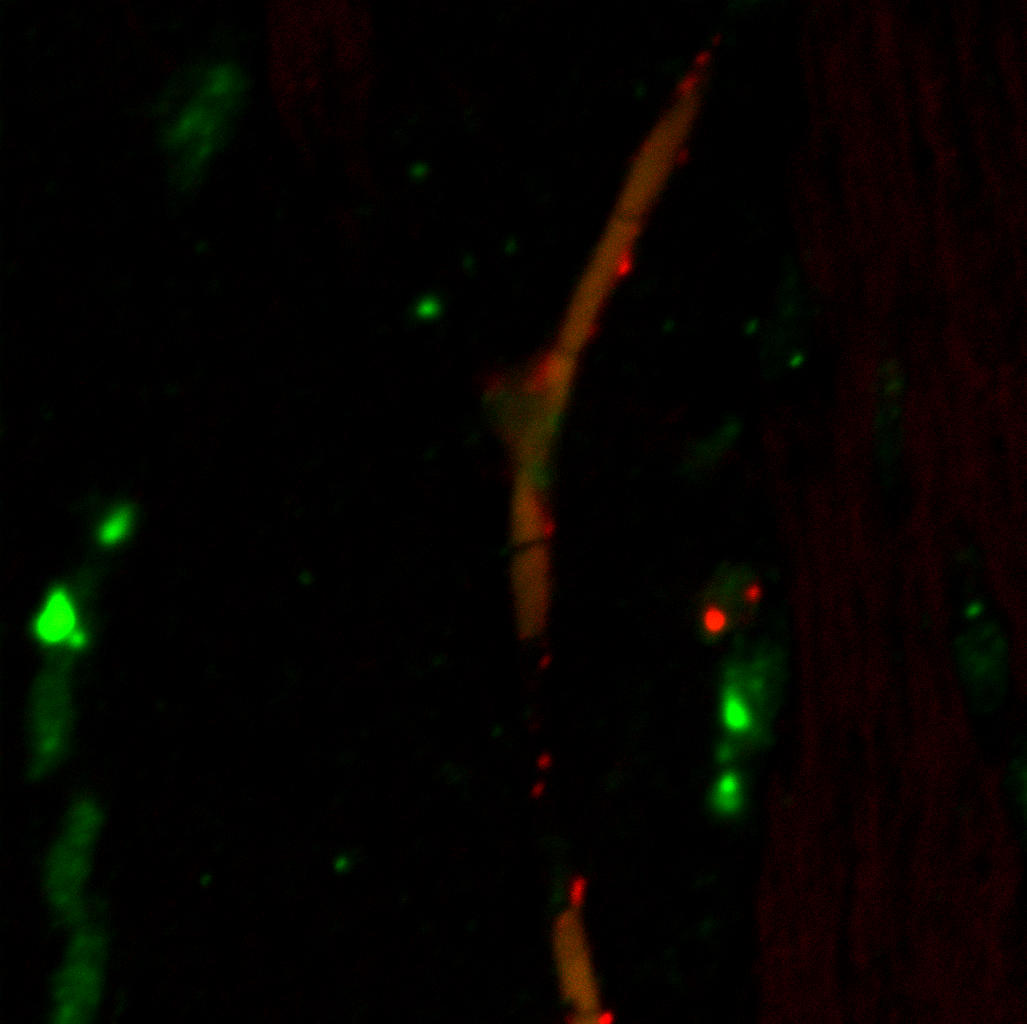

Supplement: Supplementary file 19 [file DataSheet7.ZIP › Immunofluorescence (Figure 6G, part2)/10 (presented in manuscript)/3/5S_c1+2.tif]

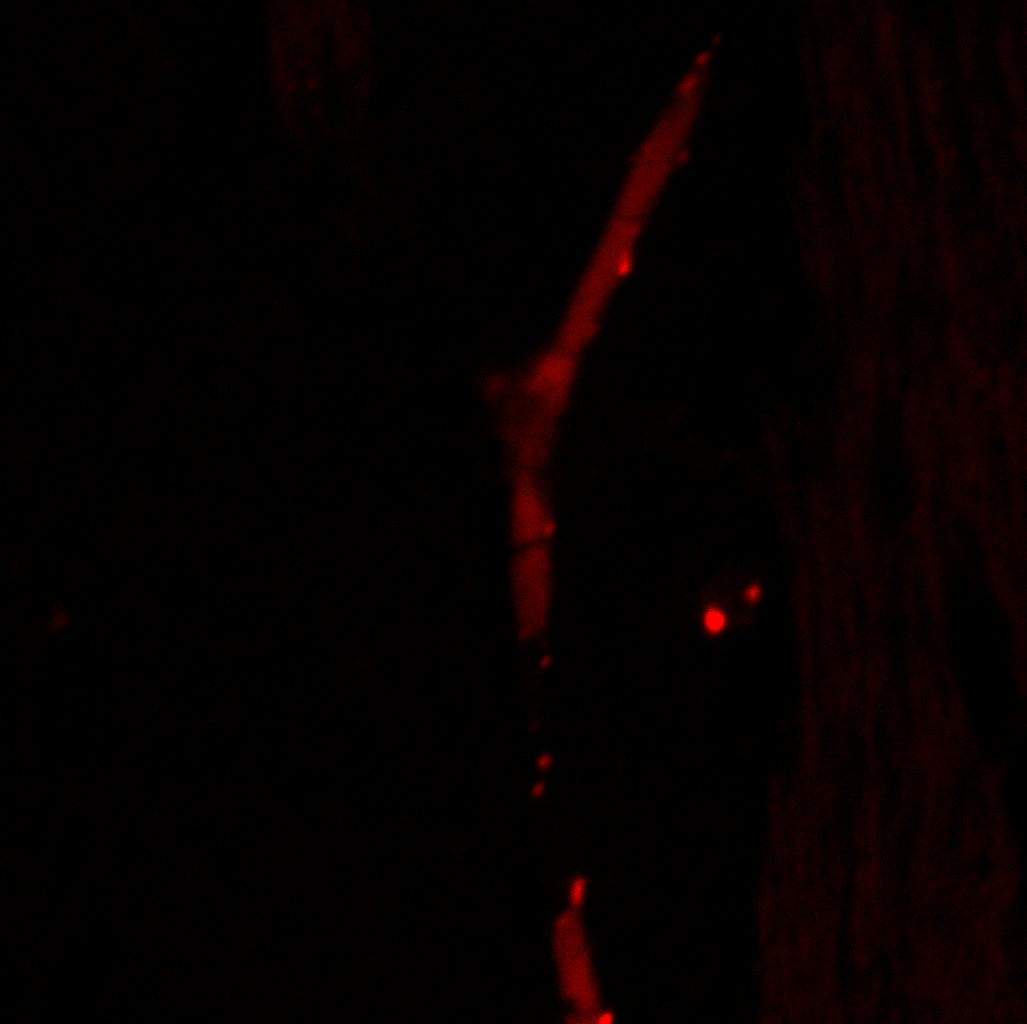

Supplement: Supplementary file 19 [file DataSheet7.ZIP › Immunofluorescence (Figure 6G, part2)/10 (presented in manuscript)/3/5S_c1.tif]

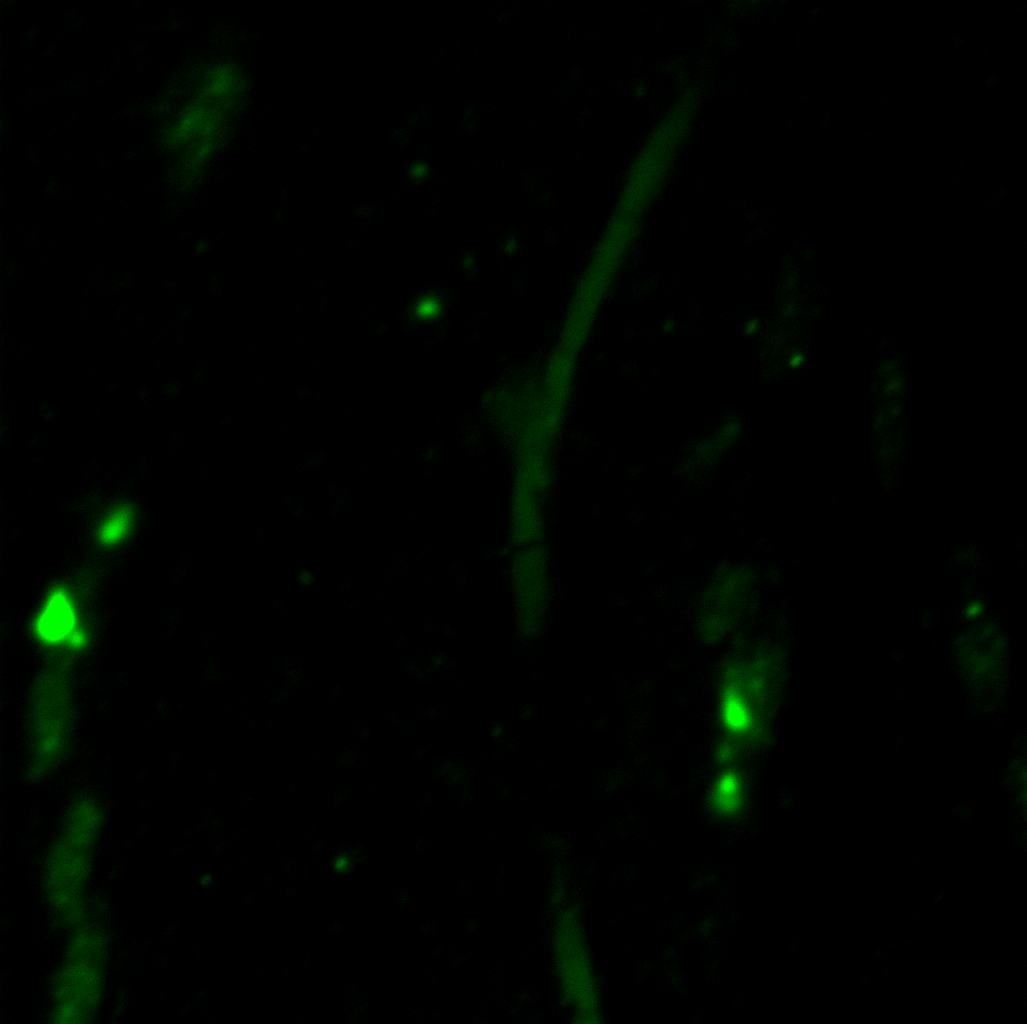

Supplement: Supplementary file 19 [file DataSheet7.ZIP › Immunofluorescence (Figure 6G, part2)/10 (presented in manuscript)/3/5S_c2.tif]

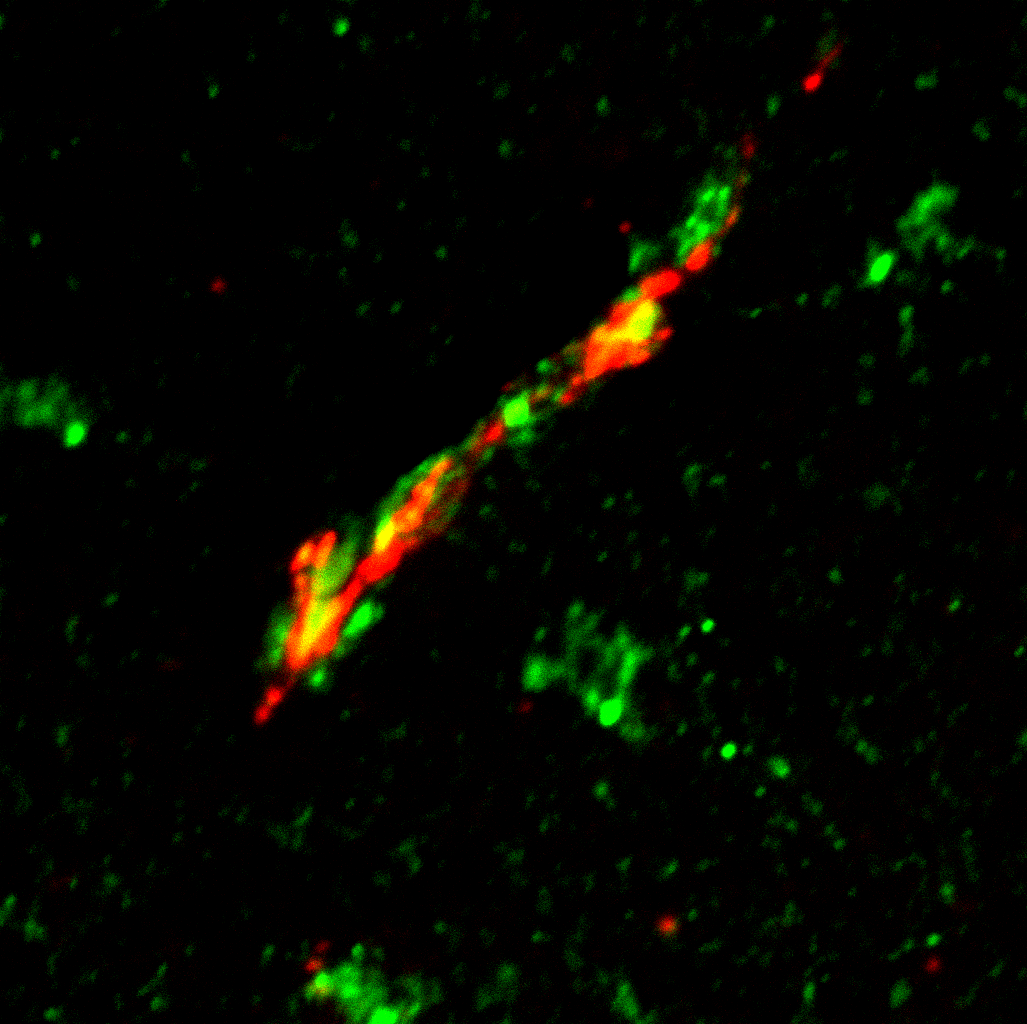

Supplement: Supplementary file 19 [file DataSheet7.ZIP › Immunofluorescence (Figure 6G, part2)/11/1/3S_c1+2.tif]

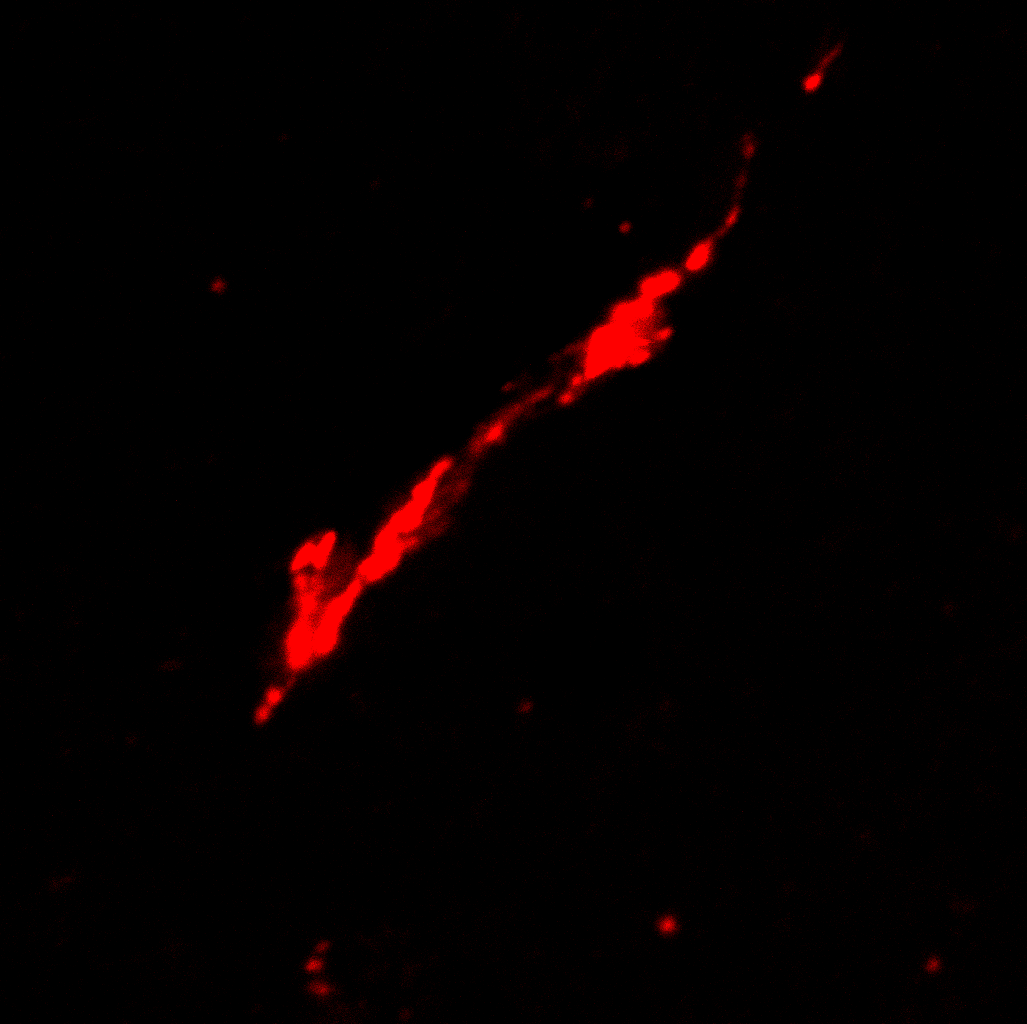

Supplement: Supplementary file 19 [file DataSheet7.ZIP › Immunofluorescence (Figure 6G, part2)/11/1/3S_c1.tif]

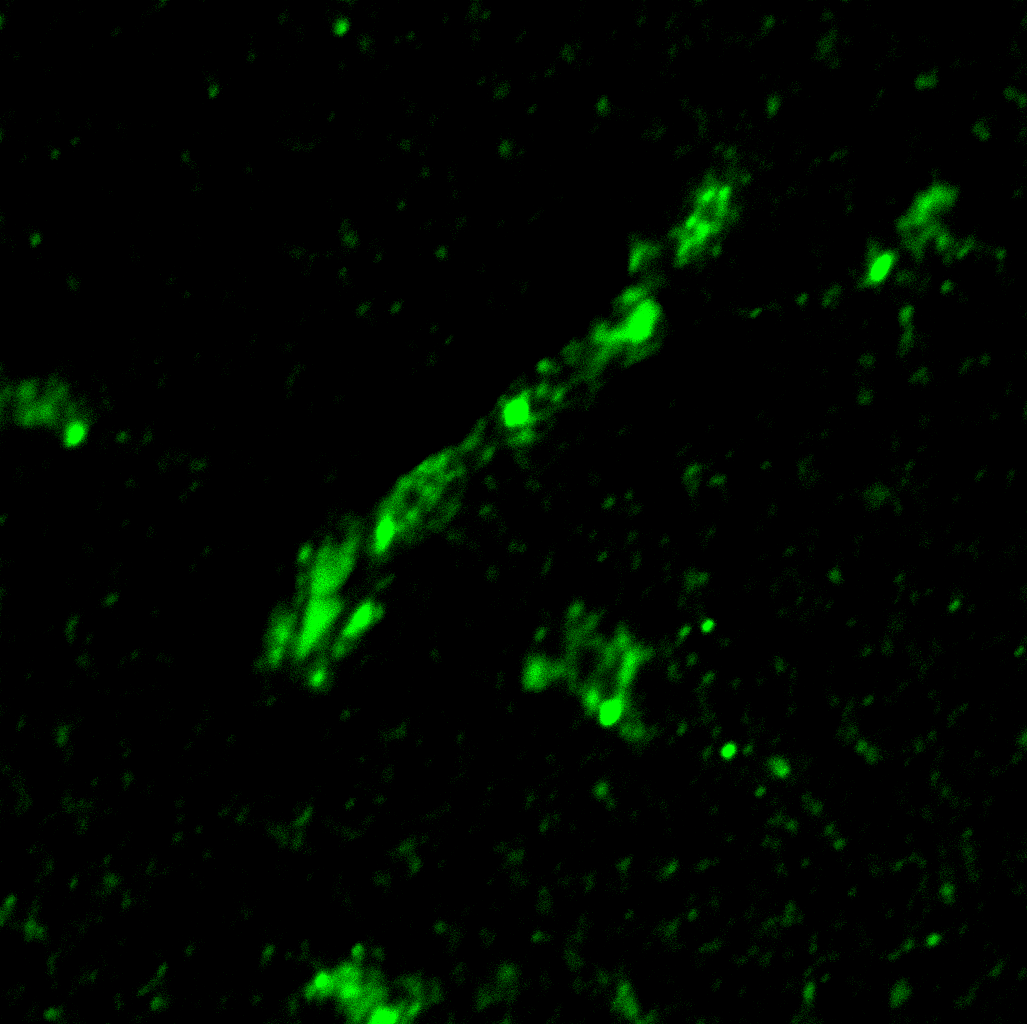

Supplement: Supplementary file 19 [file DataSheet7.ZIP › Immunofluorescence (Figure 6G, part2)/11/1/3S_c2.tif]

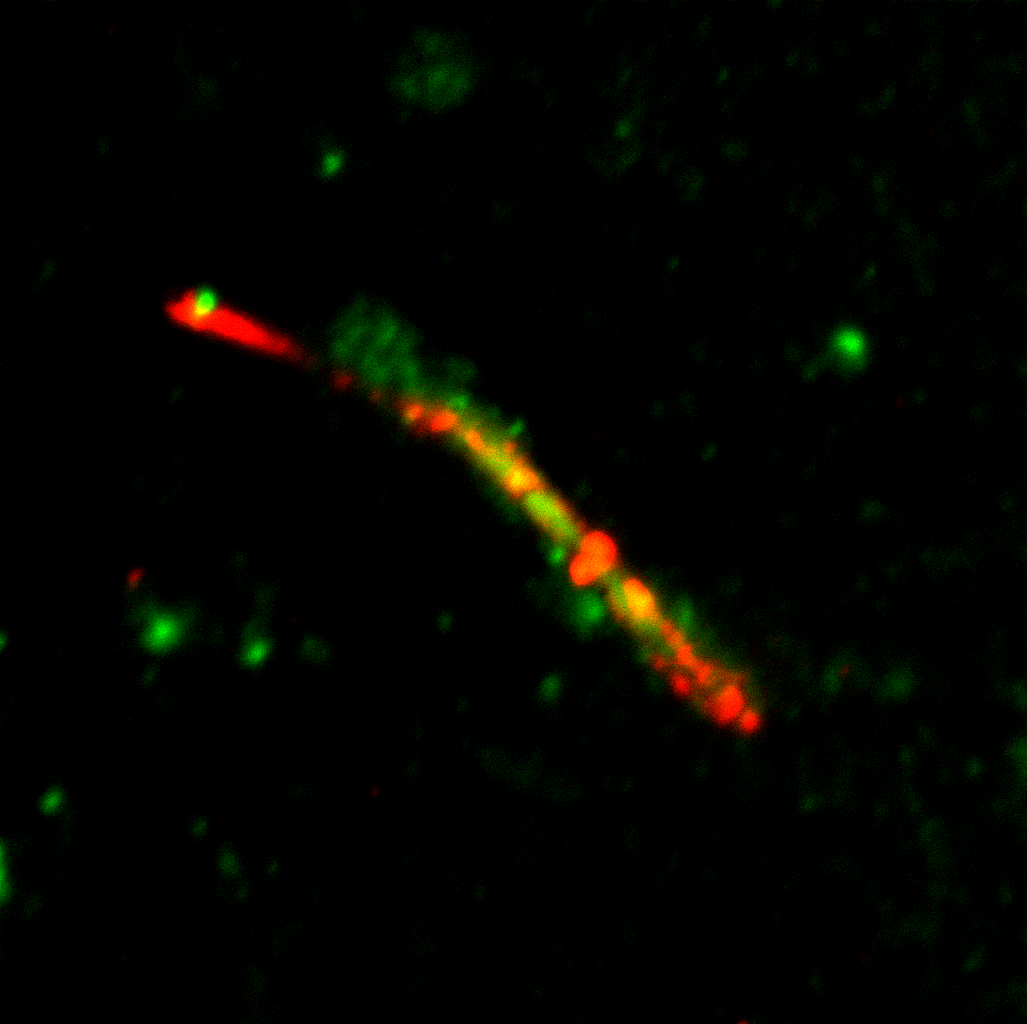

Supplement: Supplementary file 19 [file DataSheet7.ZIP › Immunofluorescence (Figure 6G, part2)/11/2/4S_c1+2.tif]

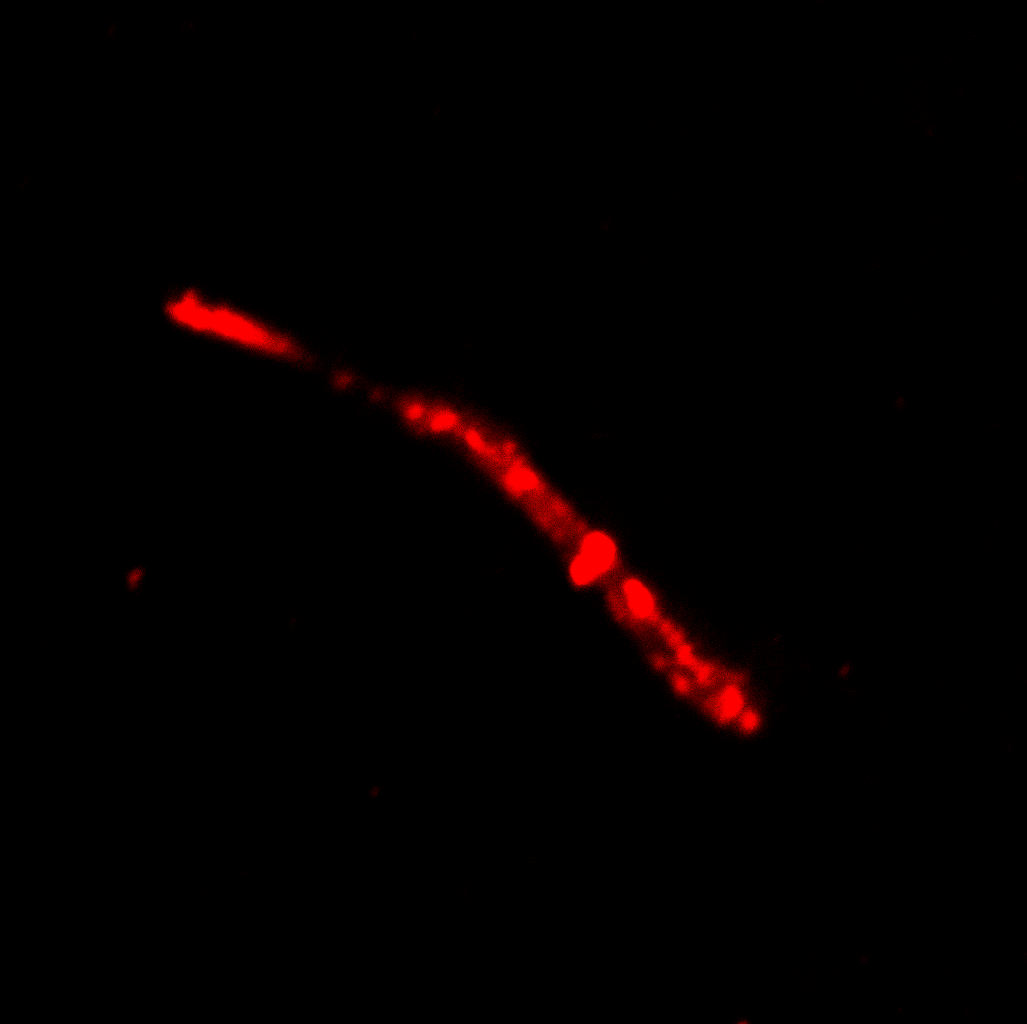

Supplement: Supplementary file 19 [file DataSheet7.ZIP › Immunofluorescence (Figure 6G, part2)/11/2/4S_c1.tif]

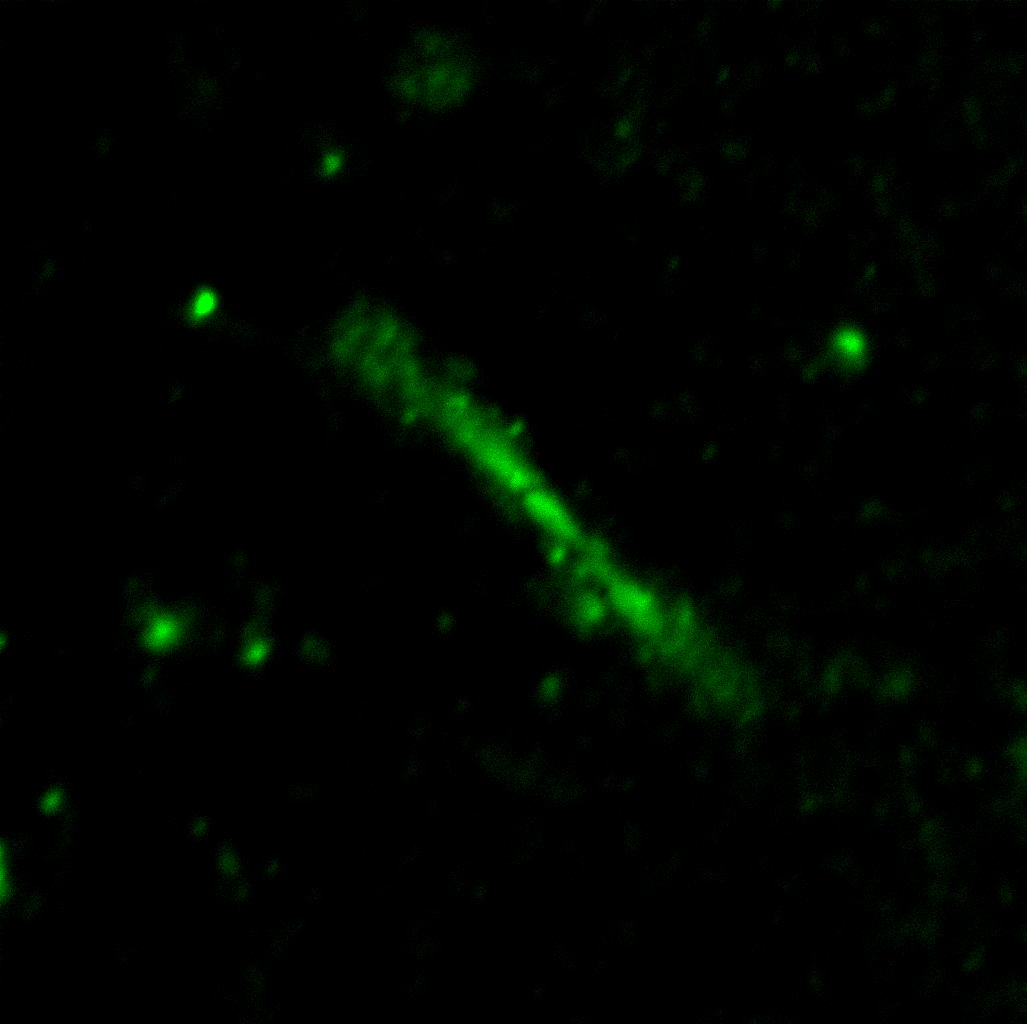

Supplement: Supplementary file 19 [file DataSheet7.ZIP › Immunofluorescence (Figure 6G, part2)/11/2/4S_c2.tif]

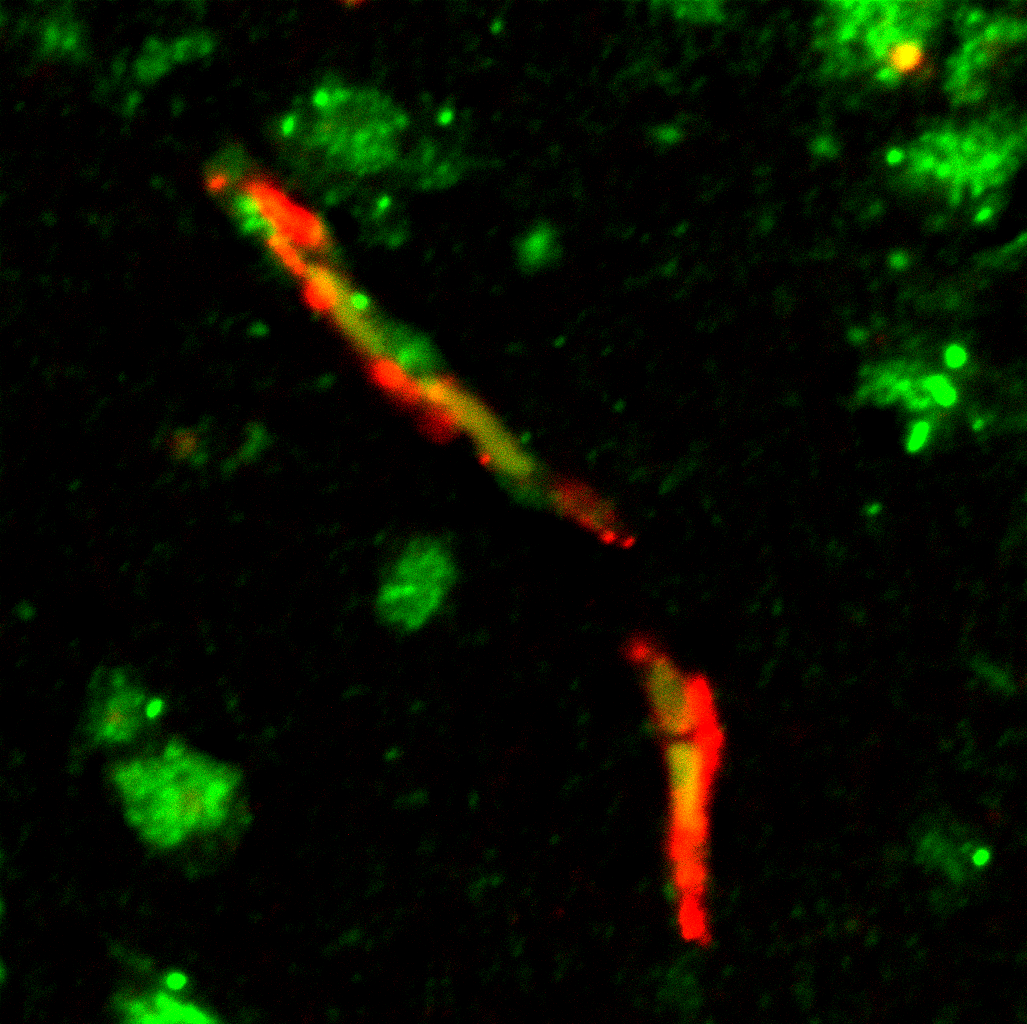

Supplement: Supplementary file 19 [file DataSheet7.ZIP › Immunofluorescence (Figure 6G, part2)/11/3/7S_c1+2.tif]

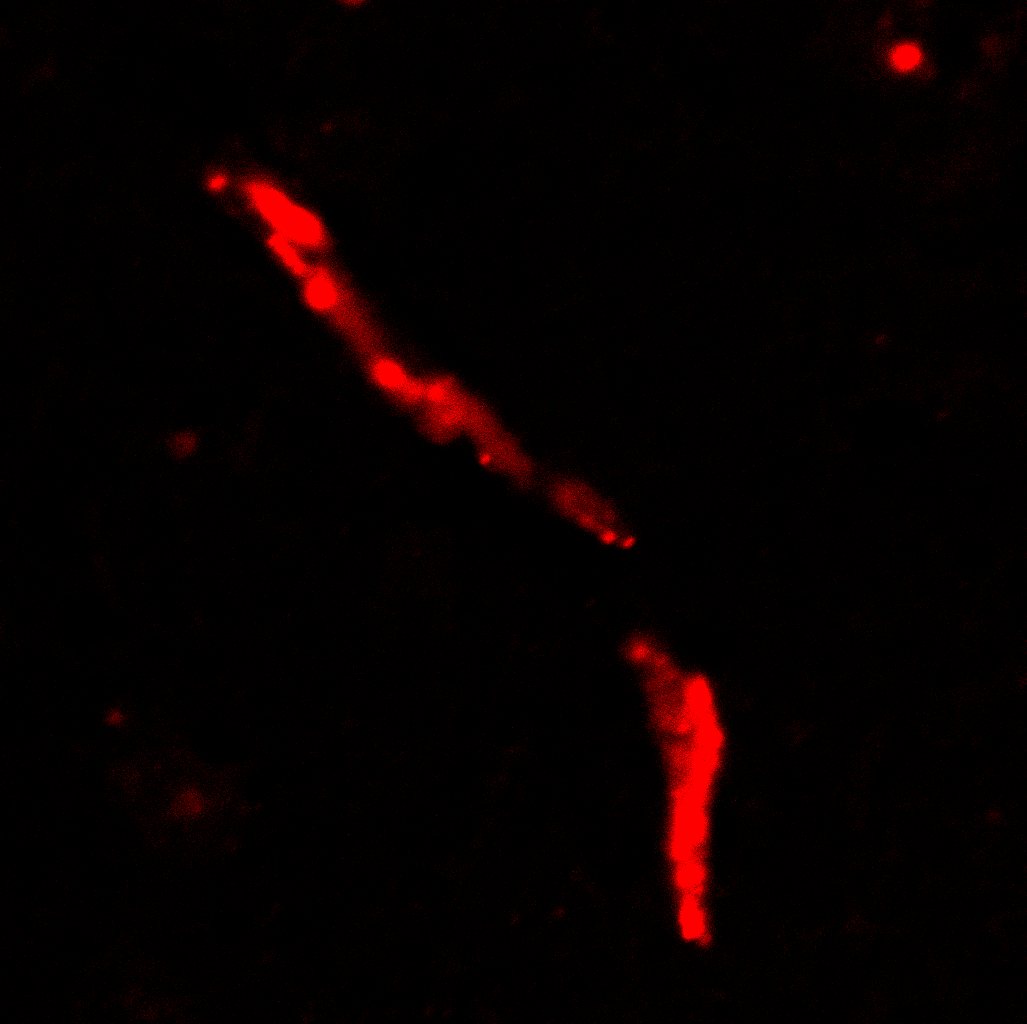

Supplement: Supplementary file 19 [file DataSheet7.ZIP › Immunofluorescence (Figure 6G, part2)/11/3/7S_c1.tif]

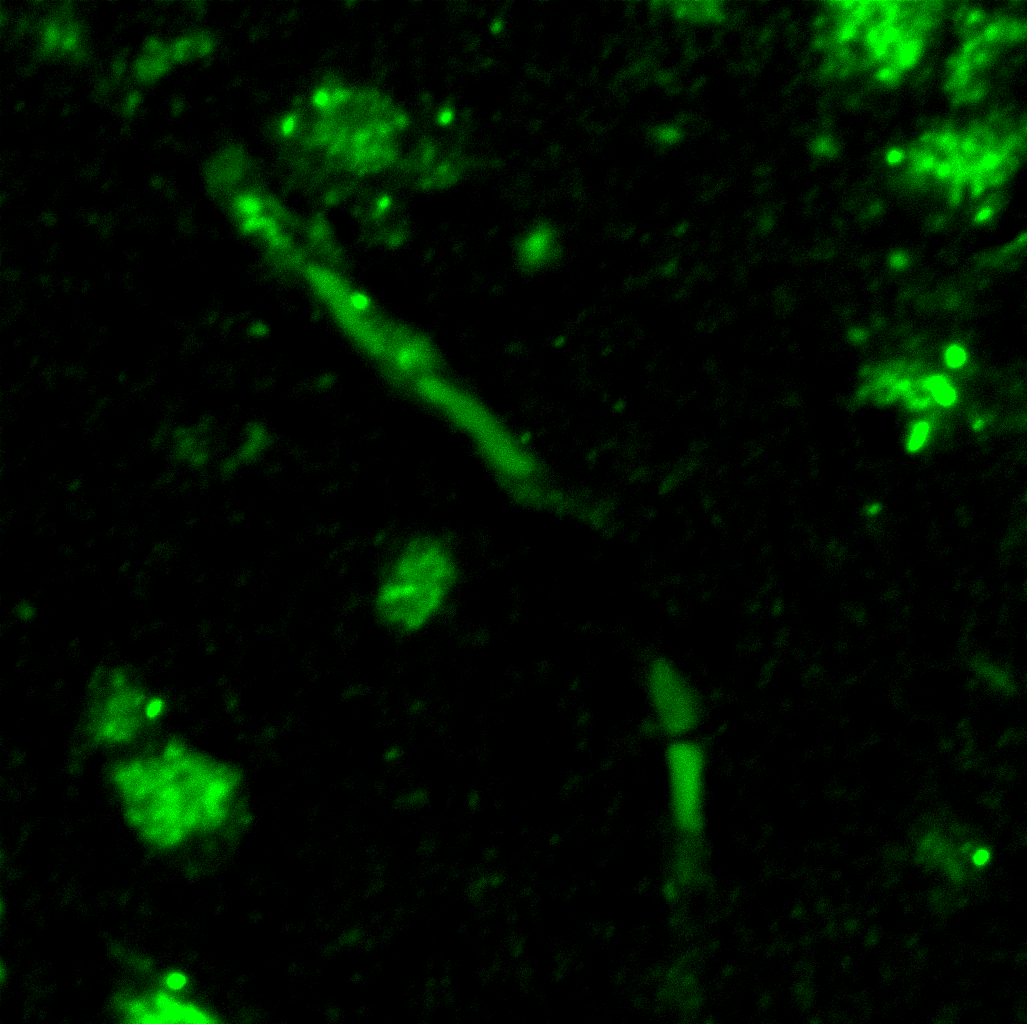

Supplement: Supplementary file 19 [file DataSheet7.ZIP › Immunofluorescence (Figure 6G, part2)/11/3/7S_c2.tif]

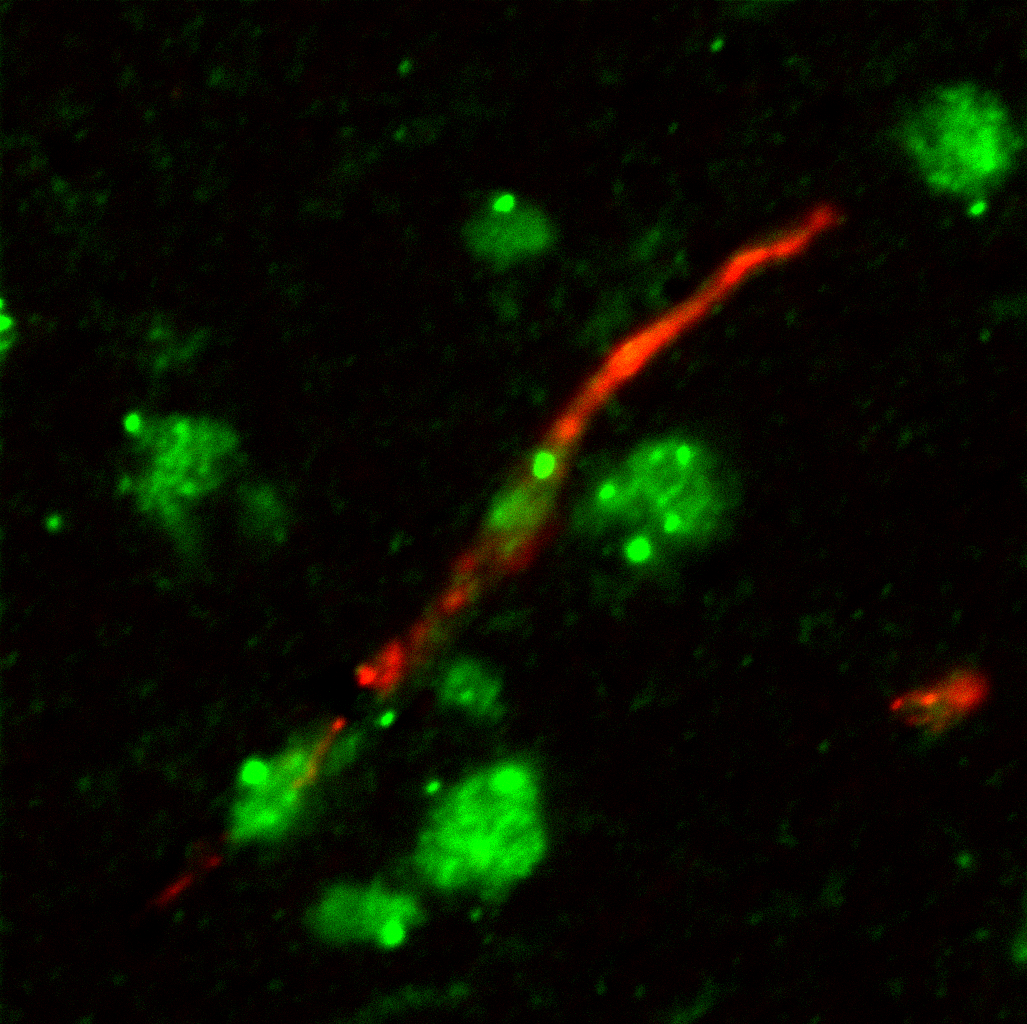

Supplement: Supplementary file 19 [file DataSheet7.ZIP › Immunofluorescence (Figure 6G, part2)/12/1/2s_c1+2.tif]

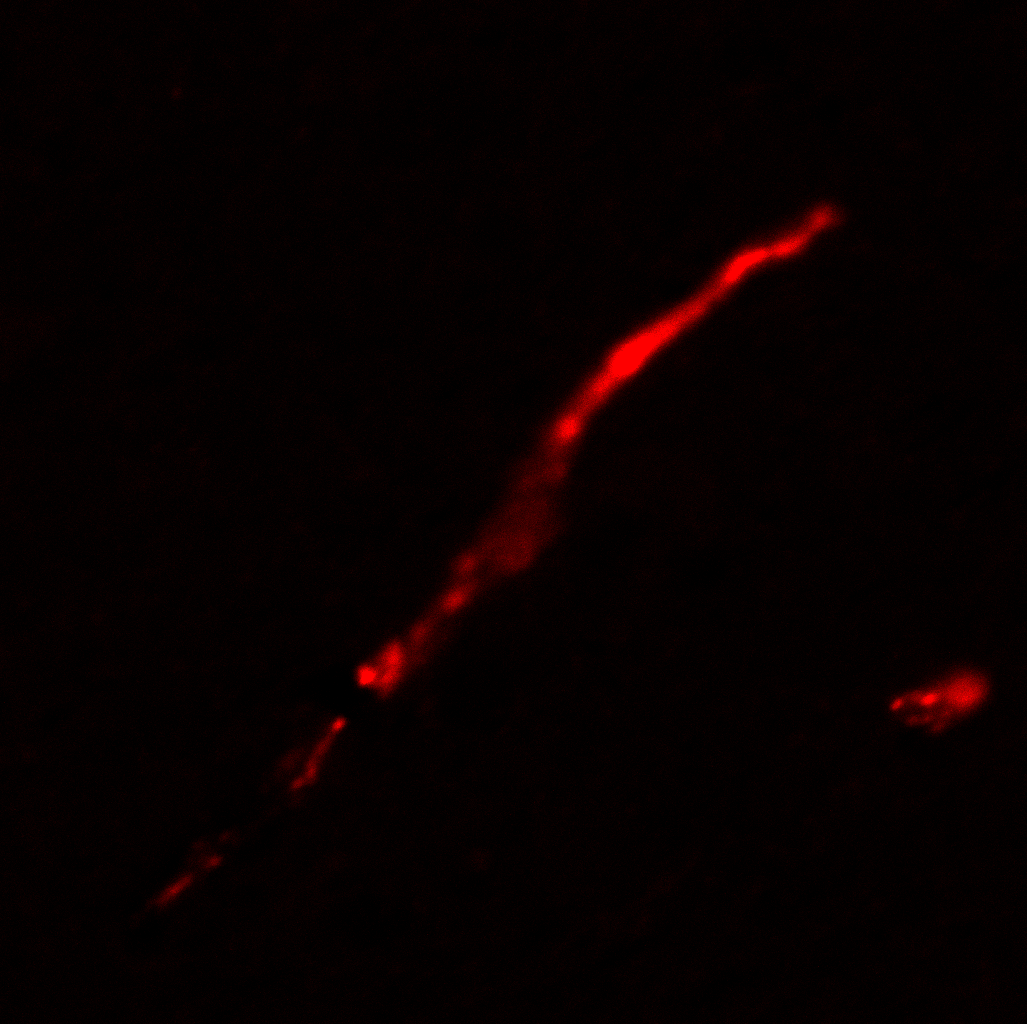

Supplement: Supplementary file 19 [file DataSheet7.ZIP › Immunofluorescence (Figure 6G, part2)/12/1/2s_c1.tif]

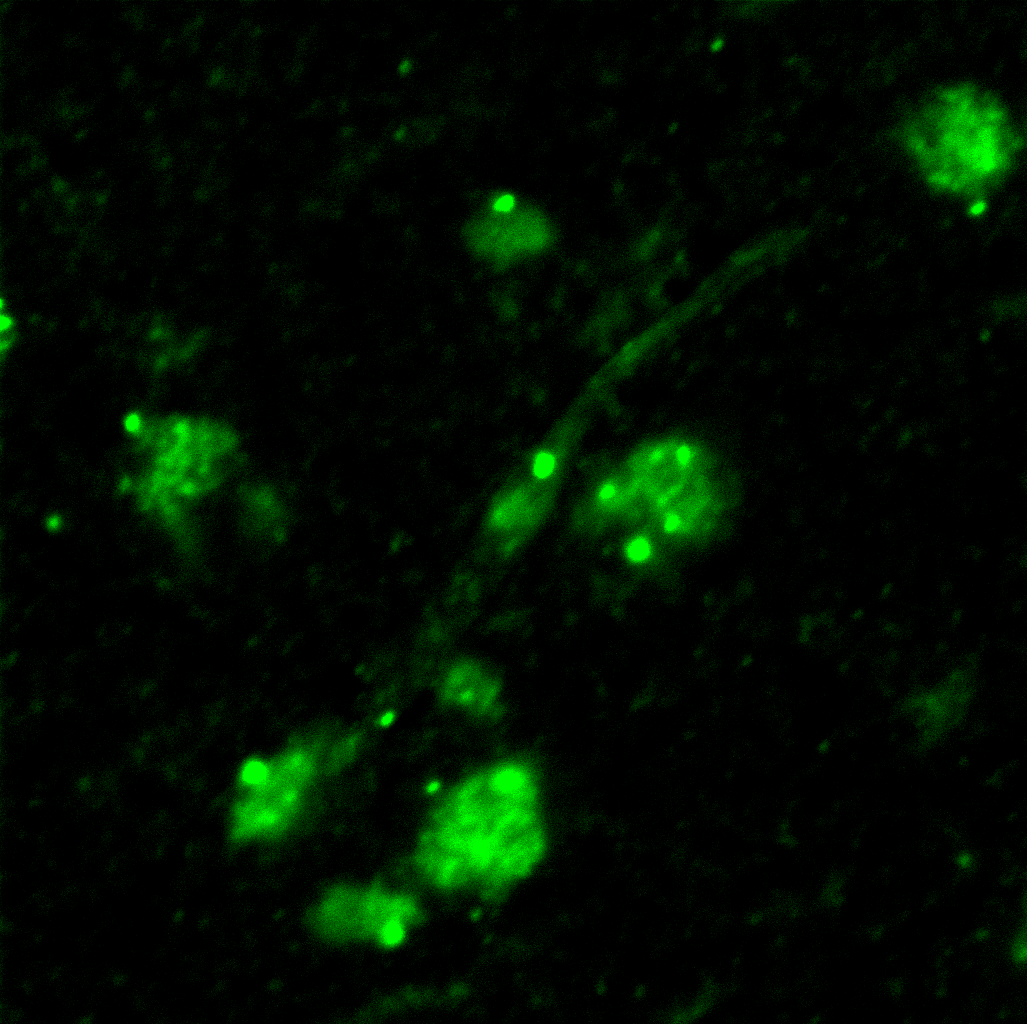

Supplement: Supplementary file 19 [file DataSheet7.ZIP › Immunofluorescence (Figure 6G, part2)/12/1/2s_c2.tif]

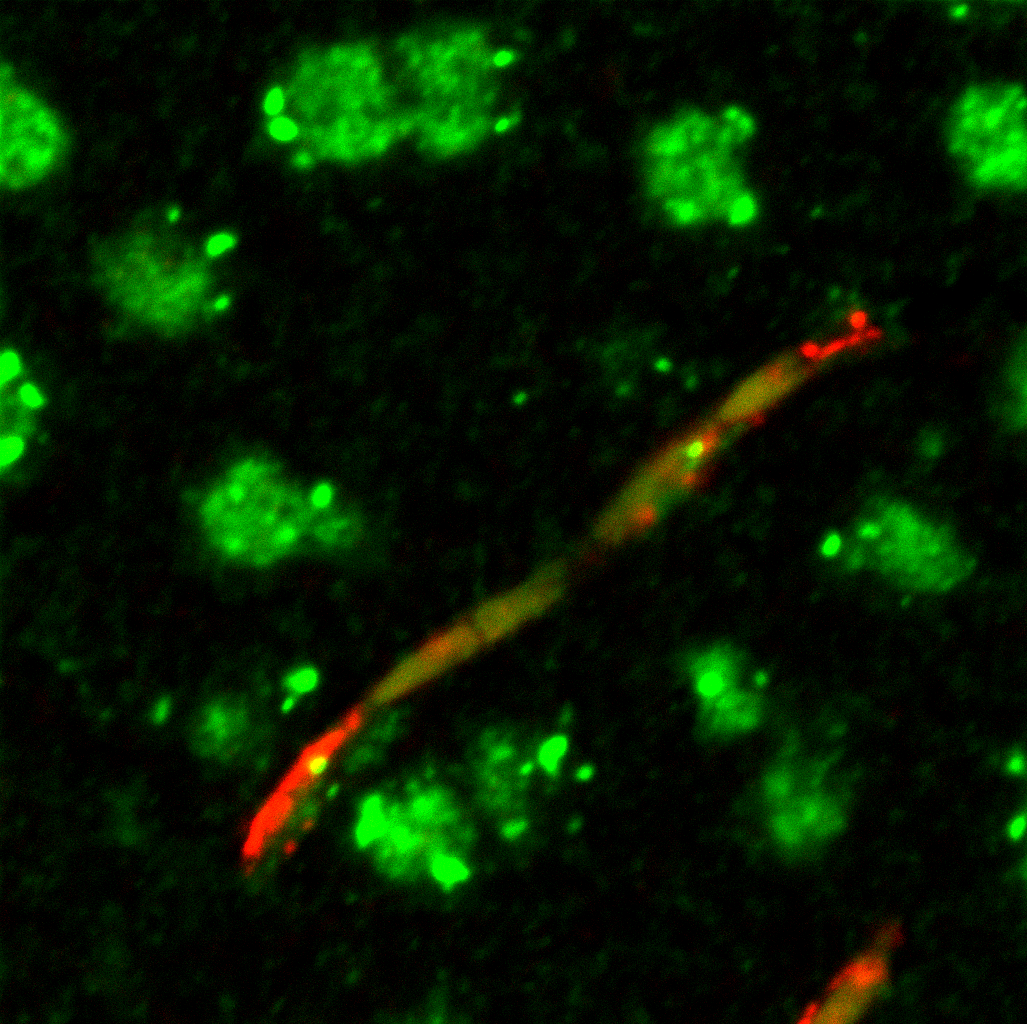

Supplement: Supplementary file 19 [file DataSheet7.ZIP › Immunofluorescence (Figure 6G, part2)/12/2/3s_c1+2.tif]

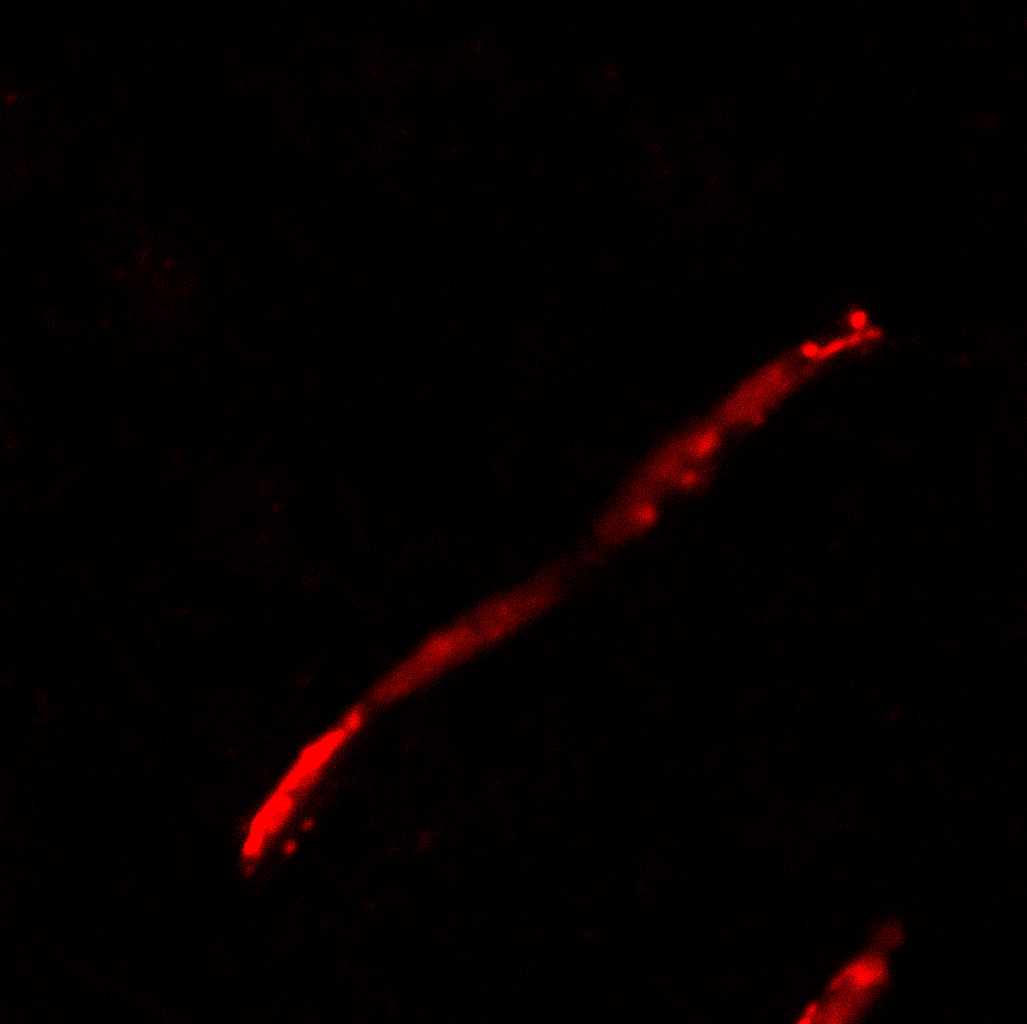

Supplement: Supplementary file 19 [file DataSheet7.ZIP › Immunofluorescence (Figure 6G, part2)/12/2/3s_c1.tif]

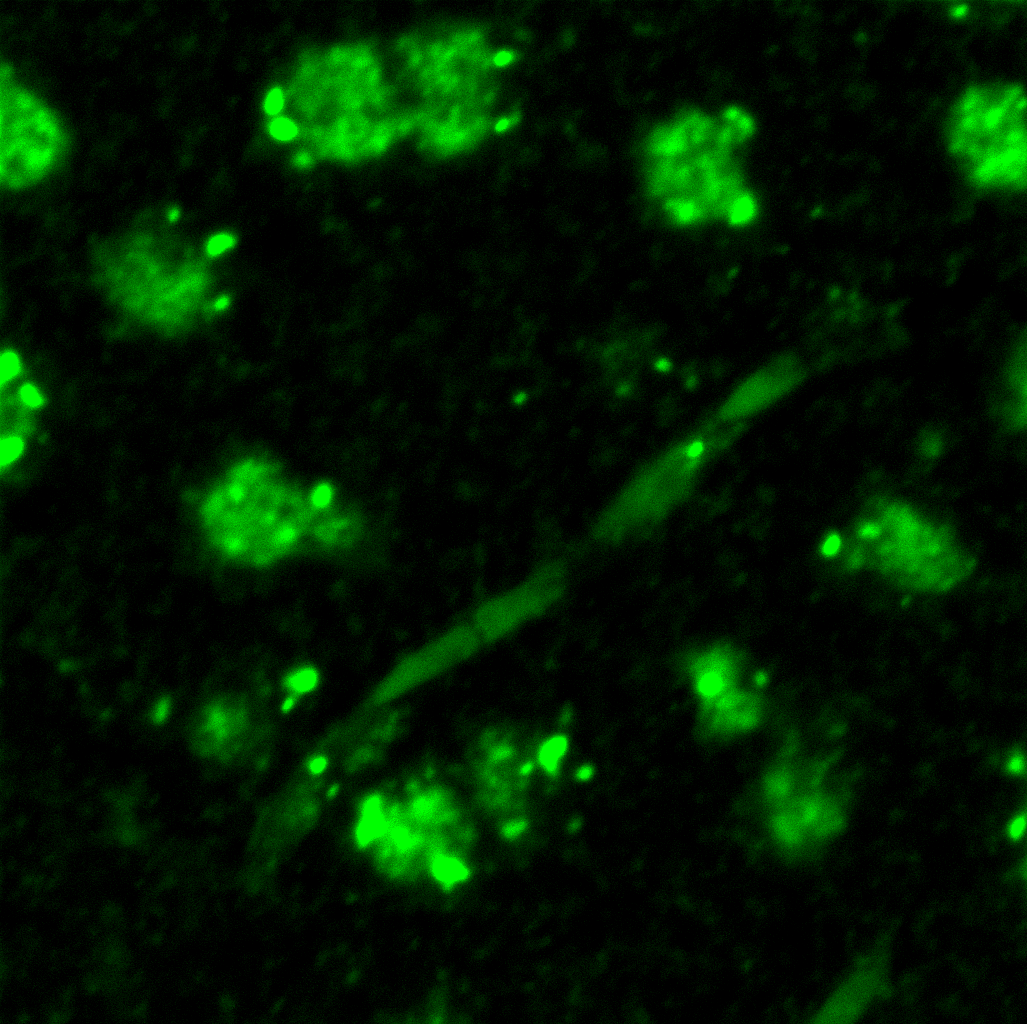

Supplement: Supplementary file 19 [file DataSheet7.ZIP › Immunofluorescence (Figure 6G, part2)/12/2/3s_c2.tif]

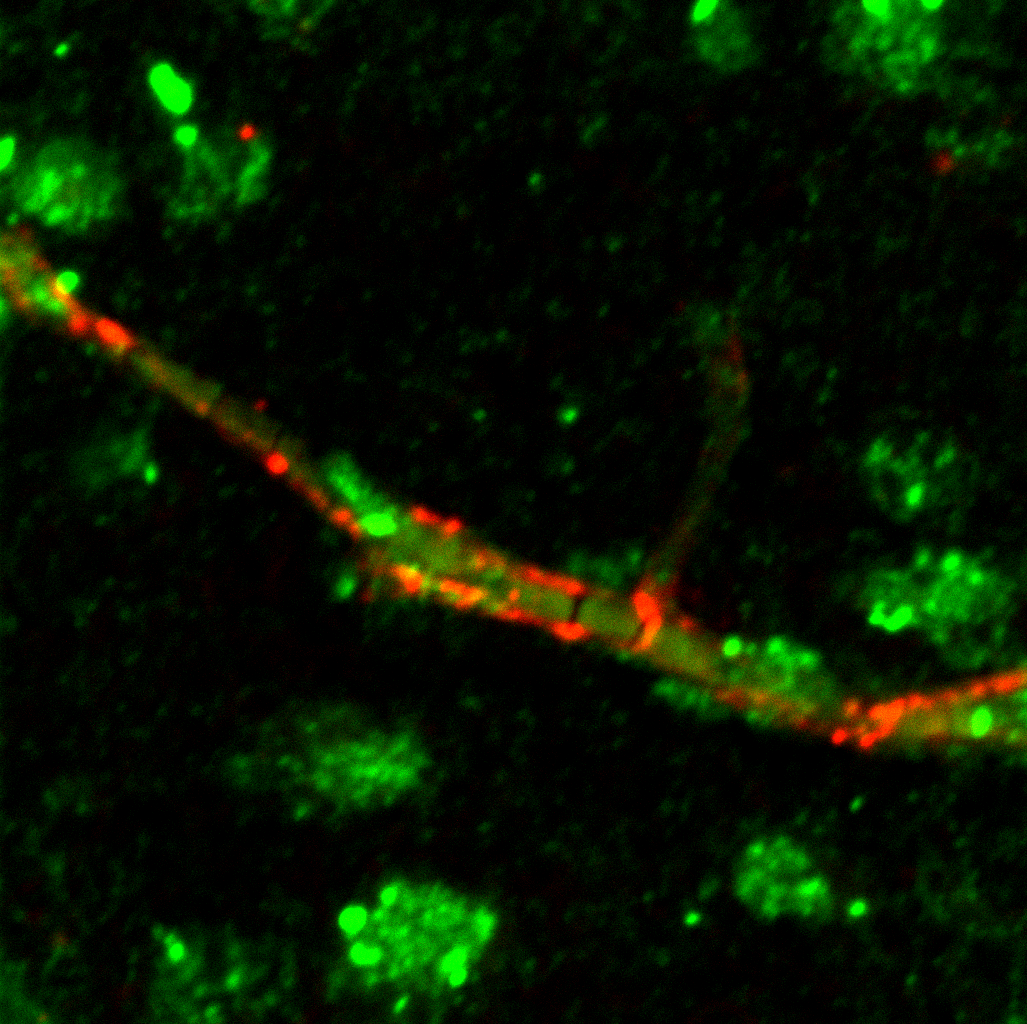

Supplement: Supplementary file 19 [file DataSheet7.ZIP › Immunofluorescence (Figure 6G, part2)/12/3/6s_c1+2.tif]

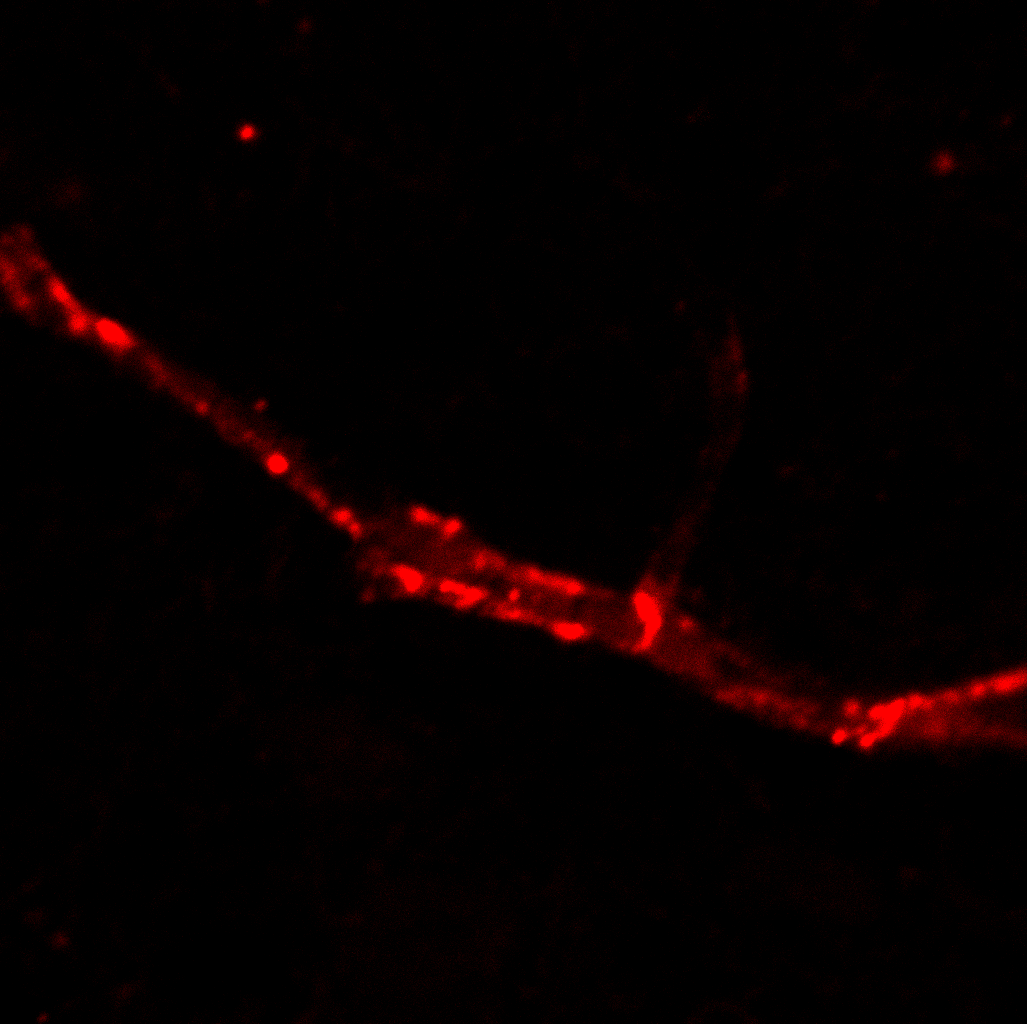

Supplement: Supplementary file 19 [file DataSheet7.ZIP › Immunofluorescence (Figure 6G, part2)/12/3/6s_c1.tif]

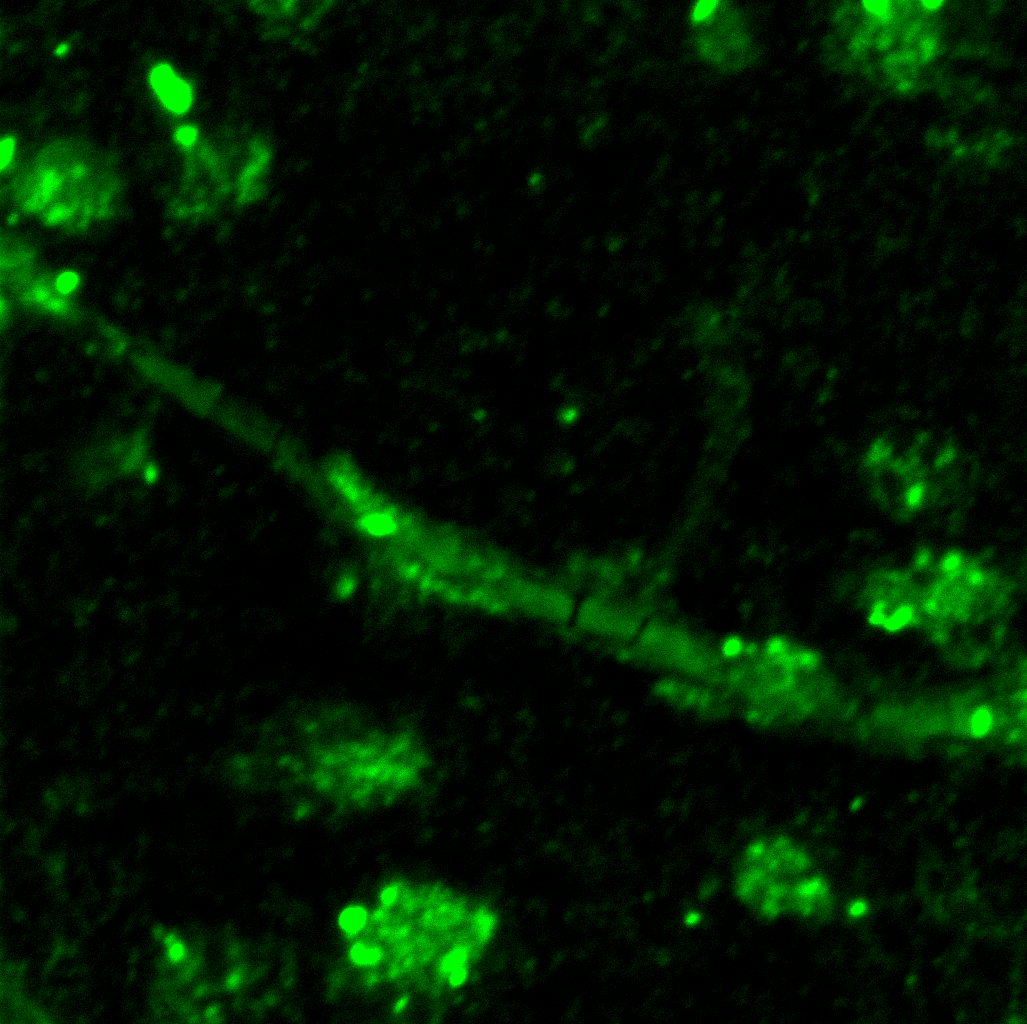

Supplement: Supplementary file 19 [file DataSheet7.ZIP › Immunofluorescence (Figure 6G, part2)/12/3/6s_c2.tif]

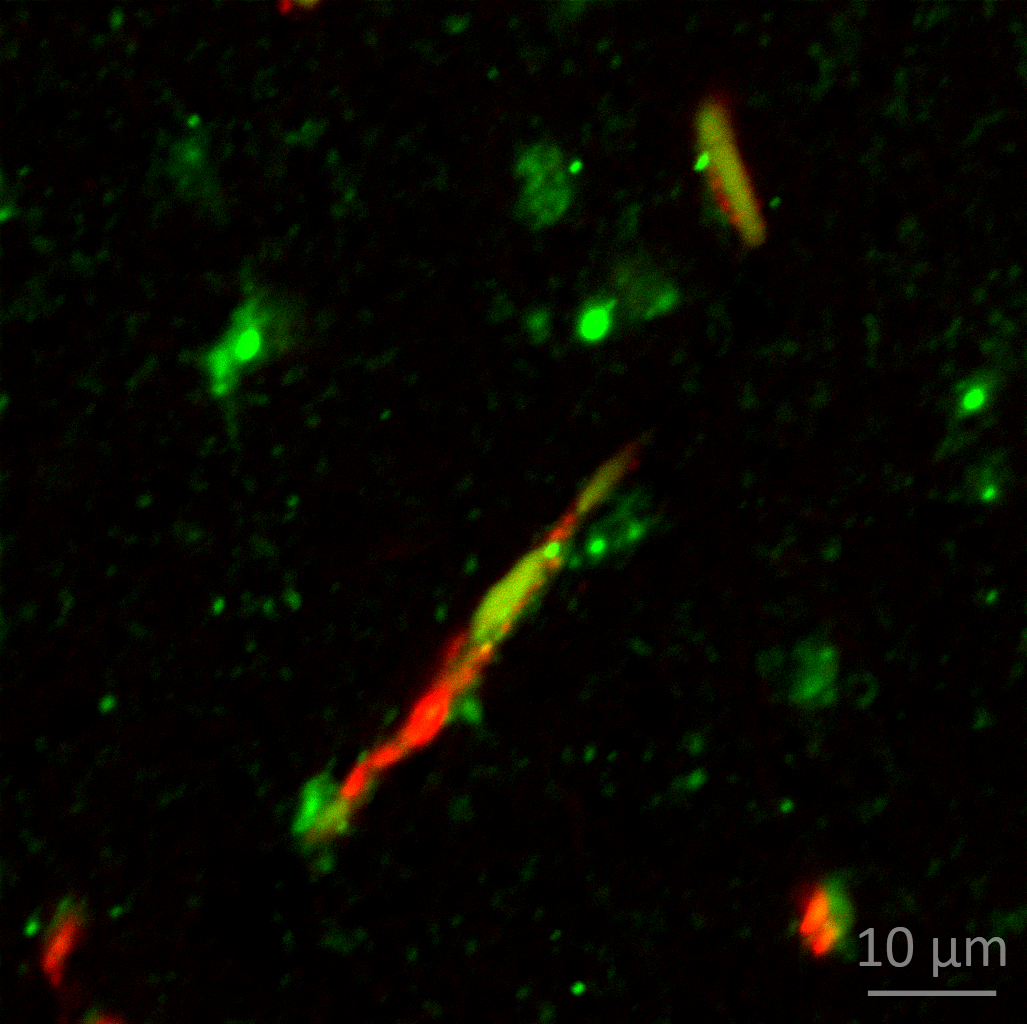

Supplement: Supplementary file 19 [file DataSheet7.ZIP › Immunofluorescence (Figure 6G, part2)/7/1/3s_c1+2.tif]

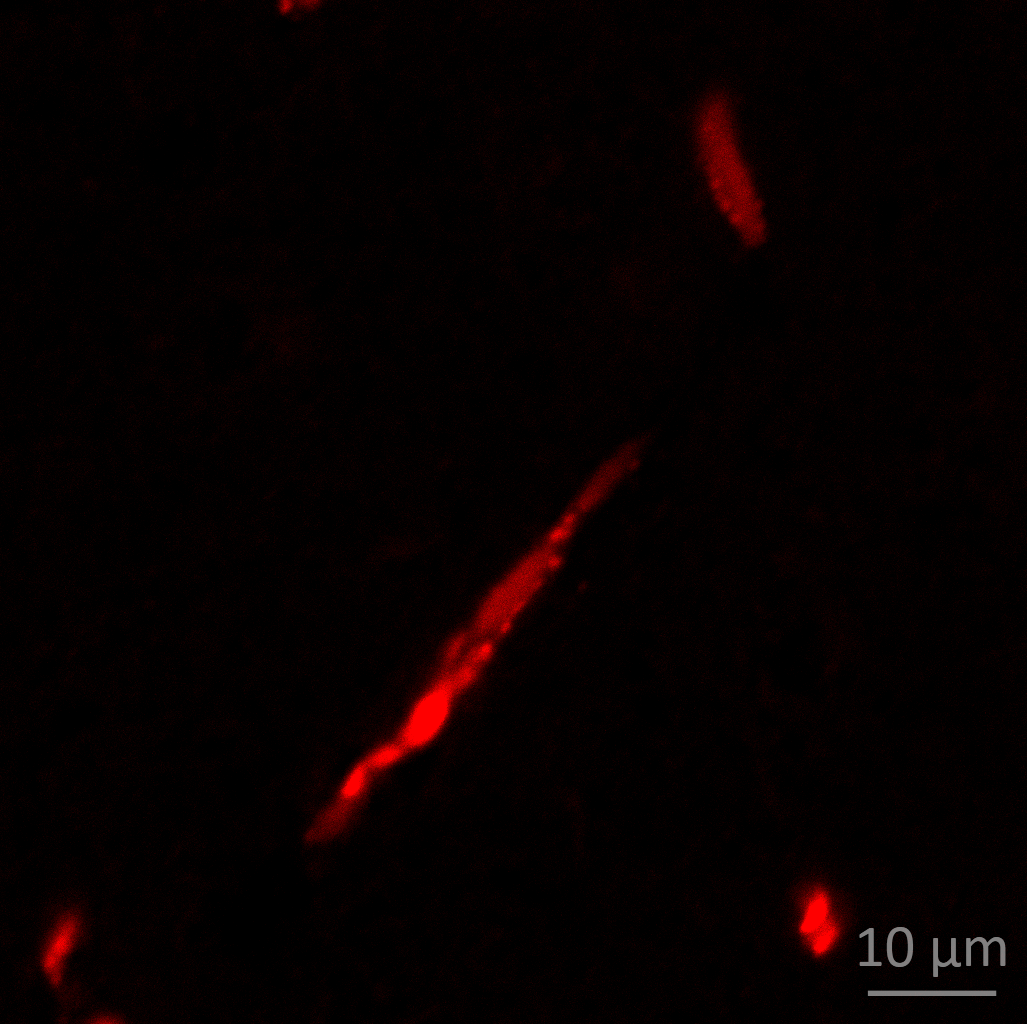

Supplement: Supplementary file 19 [file DataSheet7.ZIP › Immunofluorescence (Figure 6G, part2)/7/1/3s_c1.tif]

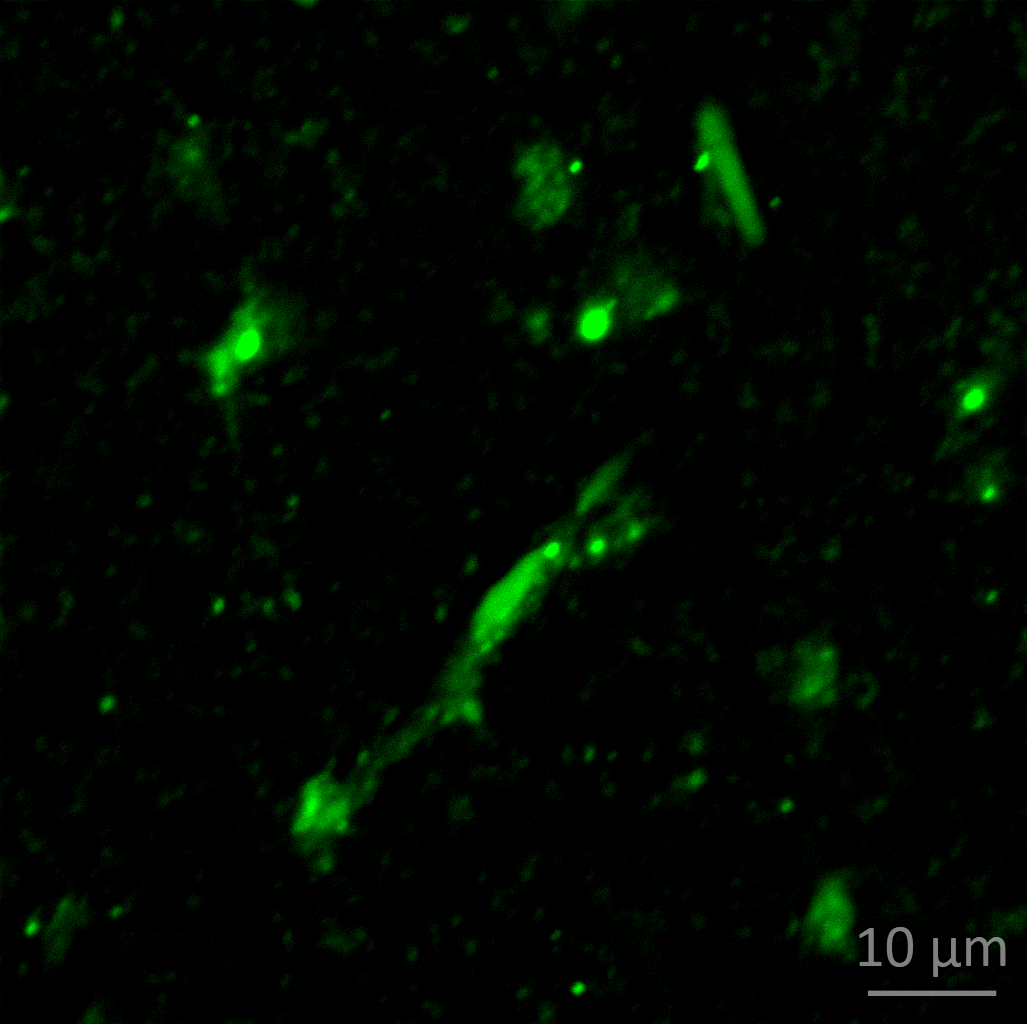

Supplement: Supplementary file 19 [file DataSheet7.ZIP › Immunofluorescence (Figure 6G, part2)/7/1/3s_c2.tif]

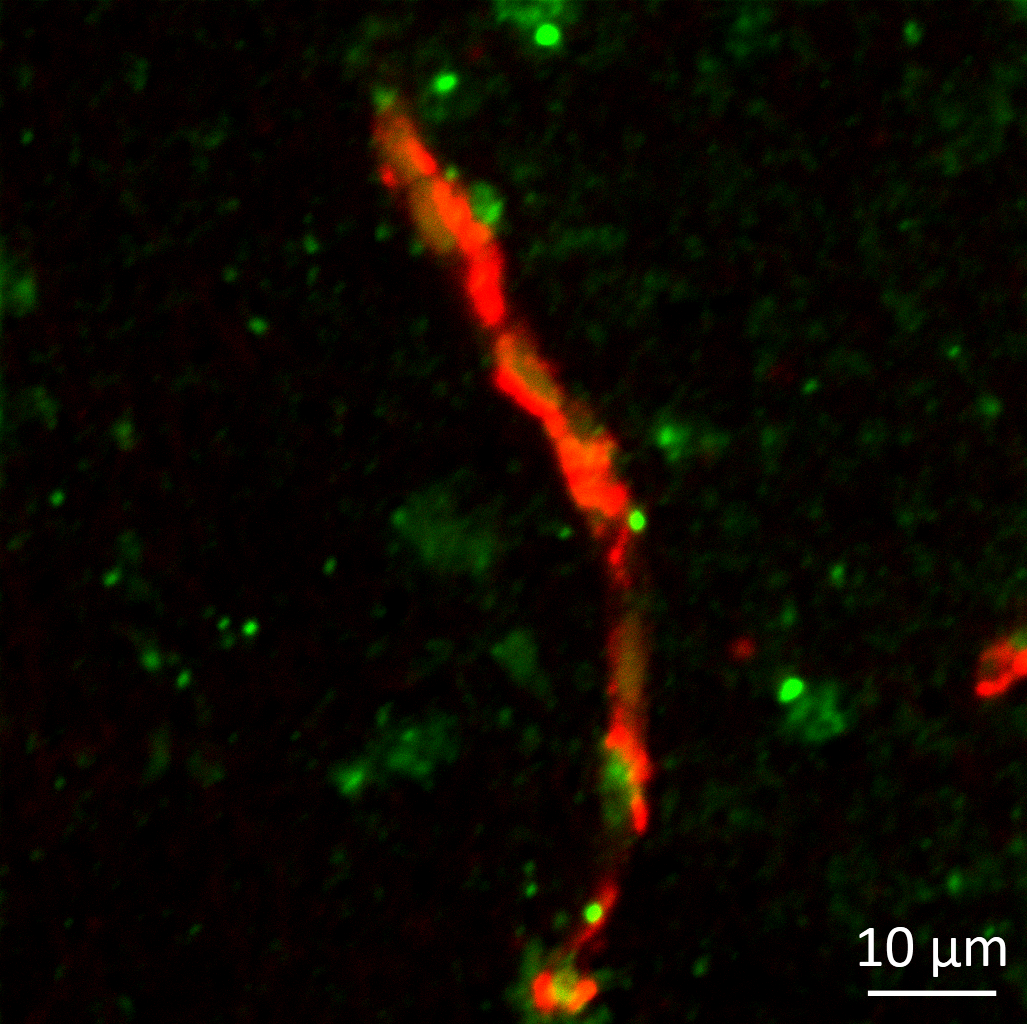

Supplement: Supplementary file 19 [file DataSheet7.ZIP › Immunofluorescence (Figure 6G, part2)/7/2/5s_c1+2.tif]

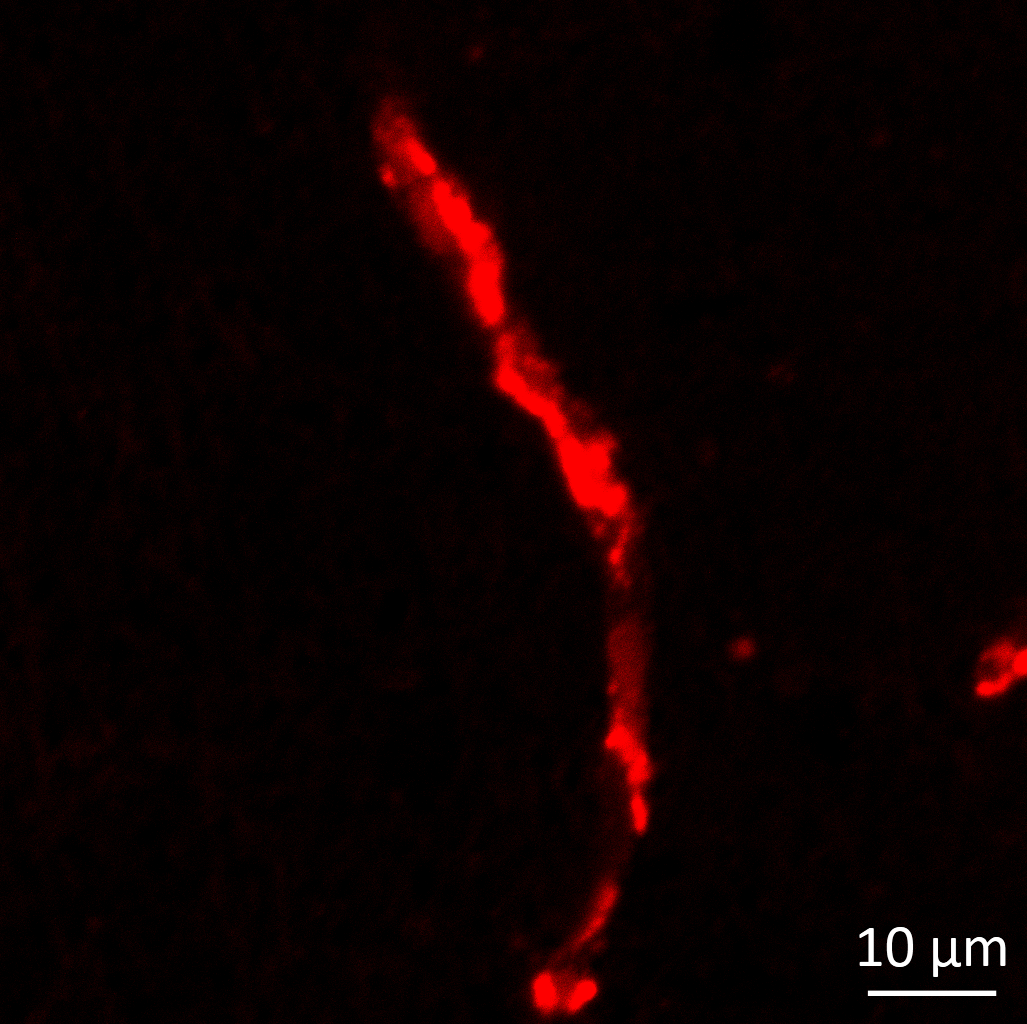

Supplement: Supplementary file 19 [file DataSheet7.ZIP › Immunofluorescence (Figure 6G, part2)/7/2/5s_c1.tif]

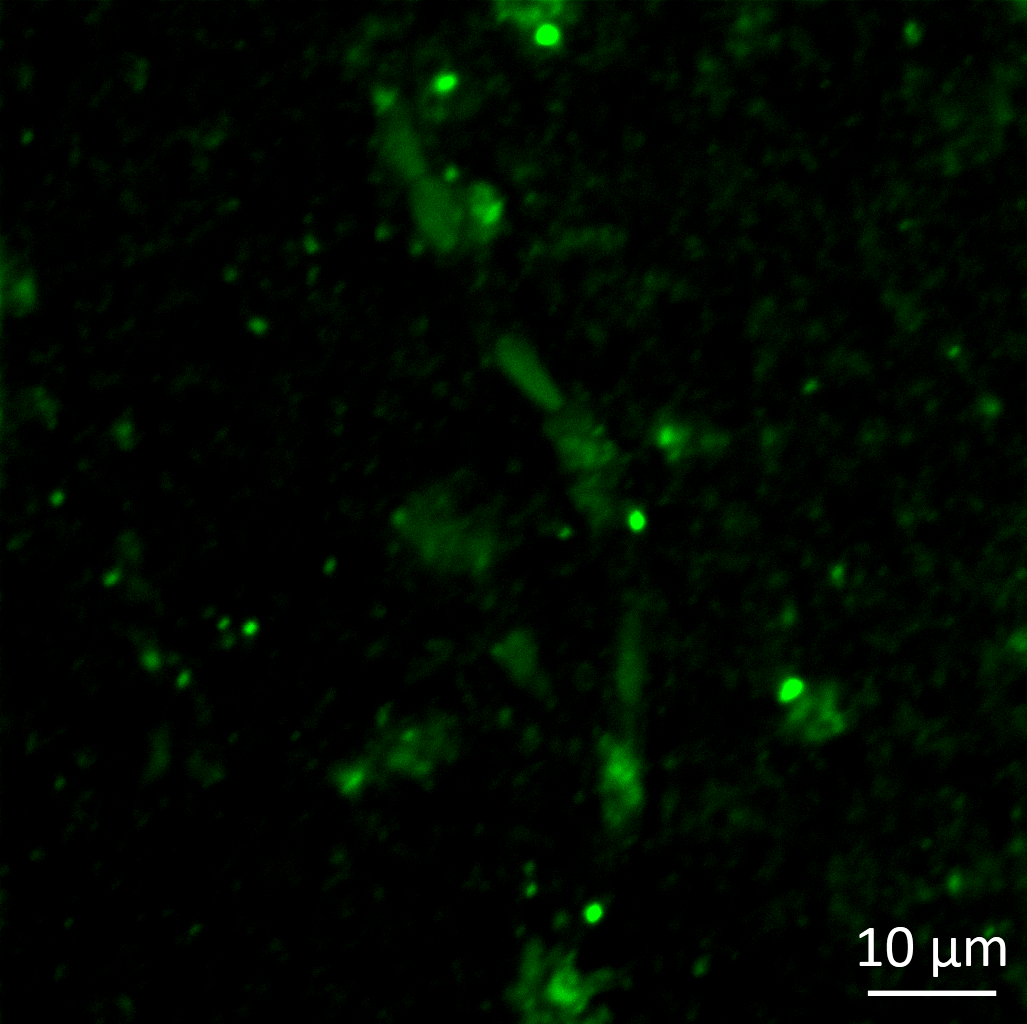

Supplement: Supplementary file 19 [file DataSheet7.ZIP › Immunofluorescence (Figure 6G, part2)/7/2/5s_c2.tif]

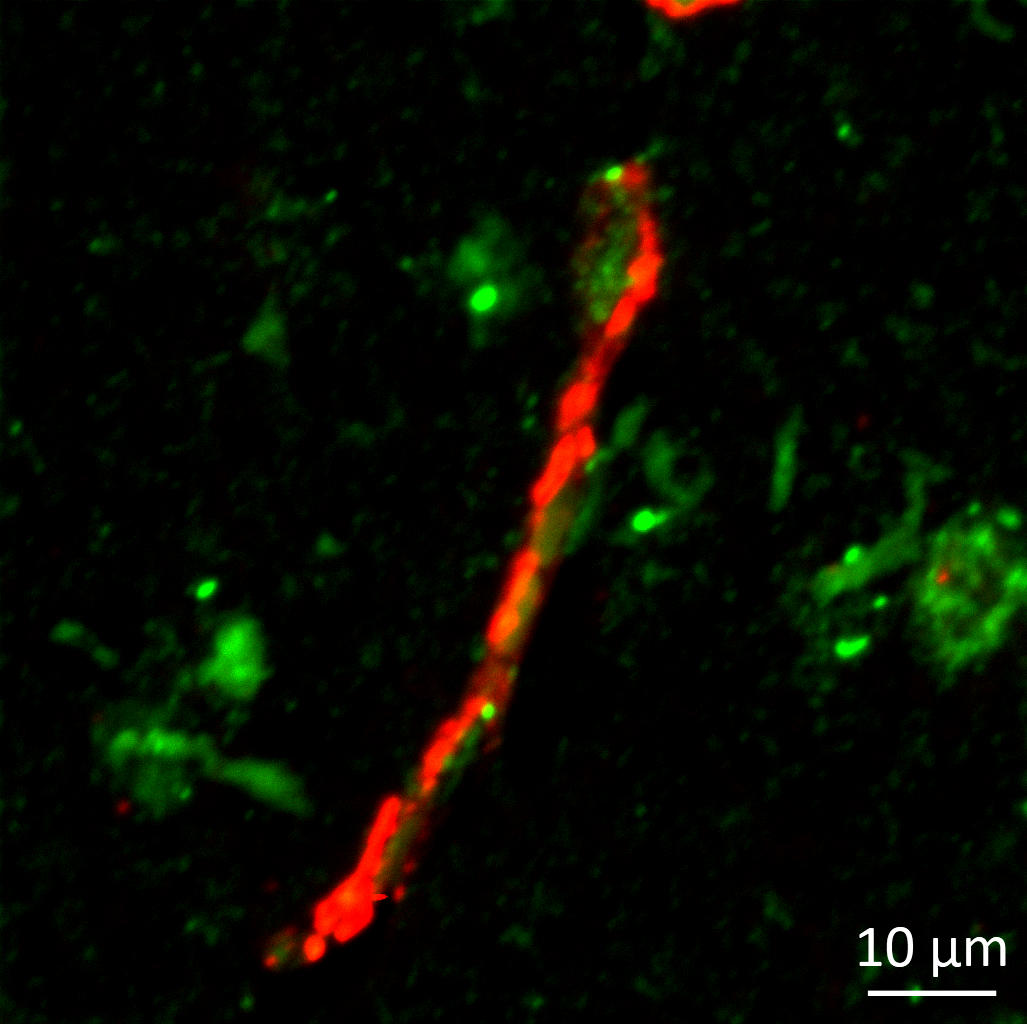

Supplement: Supplementary file 19 [file DataSheet7.ZIP › Immunofluorescence (Figure 6G, part2)/7/3/7s_c1+2.tif]

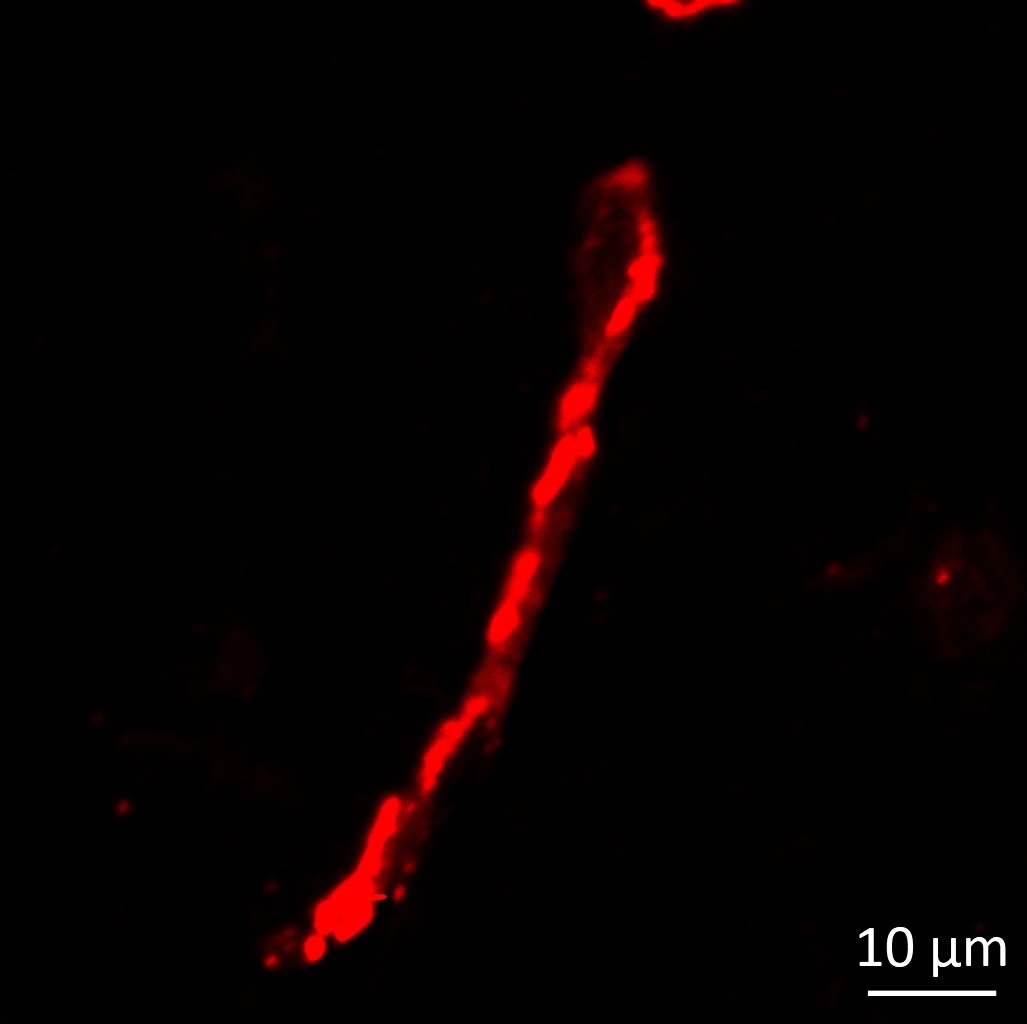

Supplement: Supplementary file 19 [file DataSheet7.ZIP › Immunofluorescence (Figure 6G, part2)/7/3/7s_c1.tif]

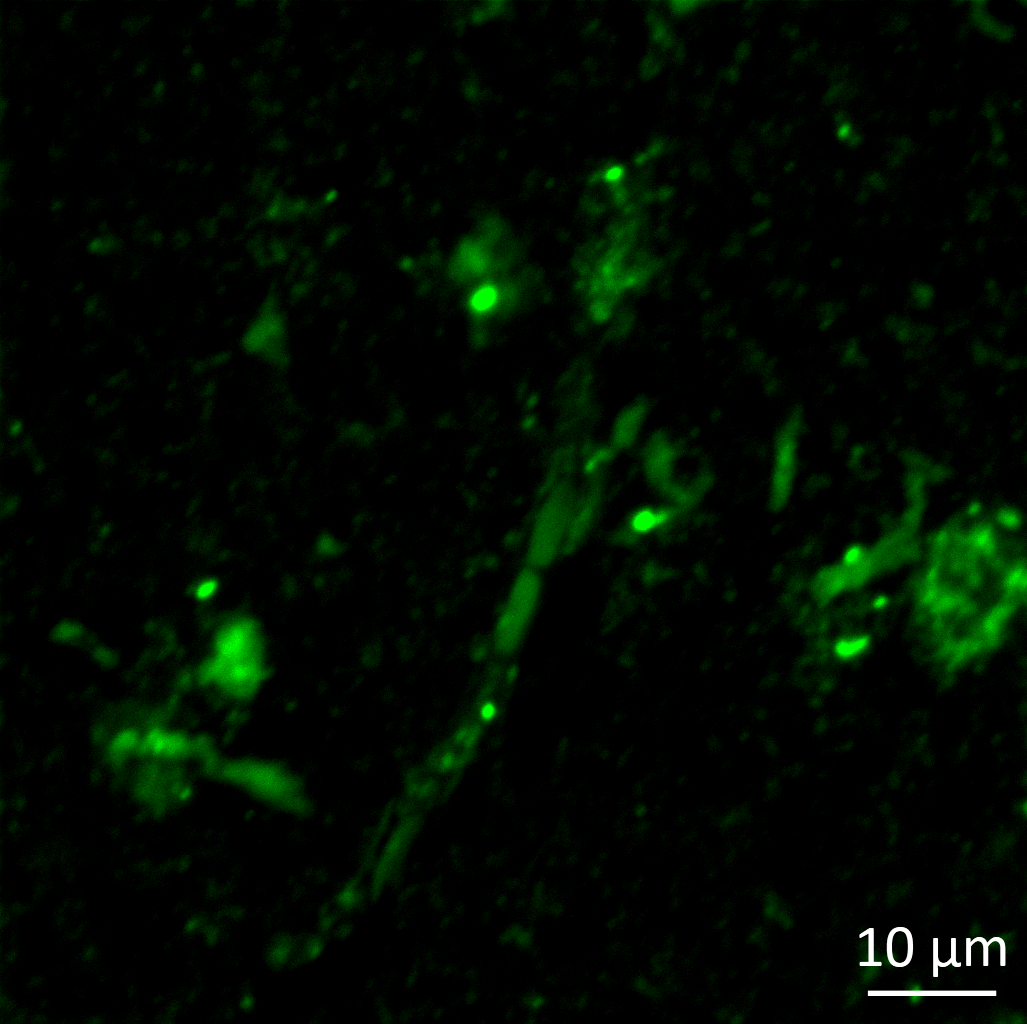

Supplement: Supplementary file 19 [file DataSheet7.ZIP › Immunofluorescence (Figure 6G, part2)/7/3/7s_c2.tif]

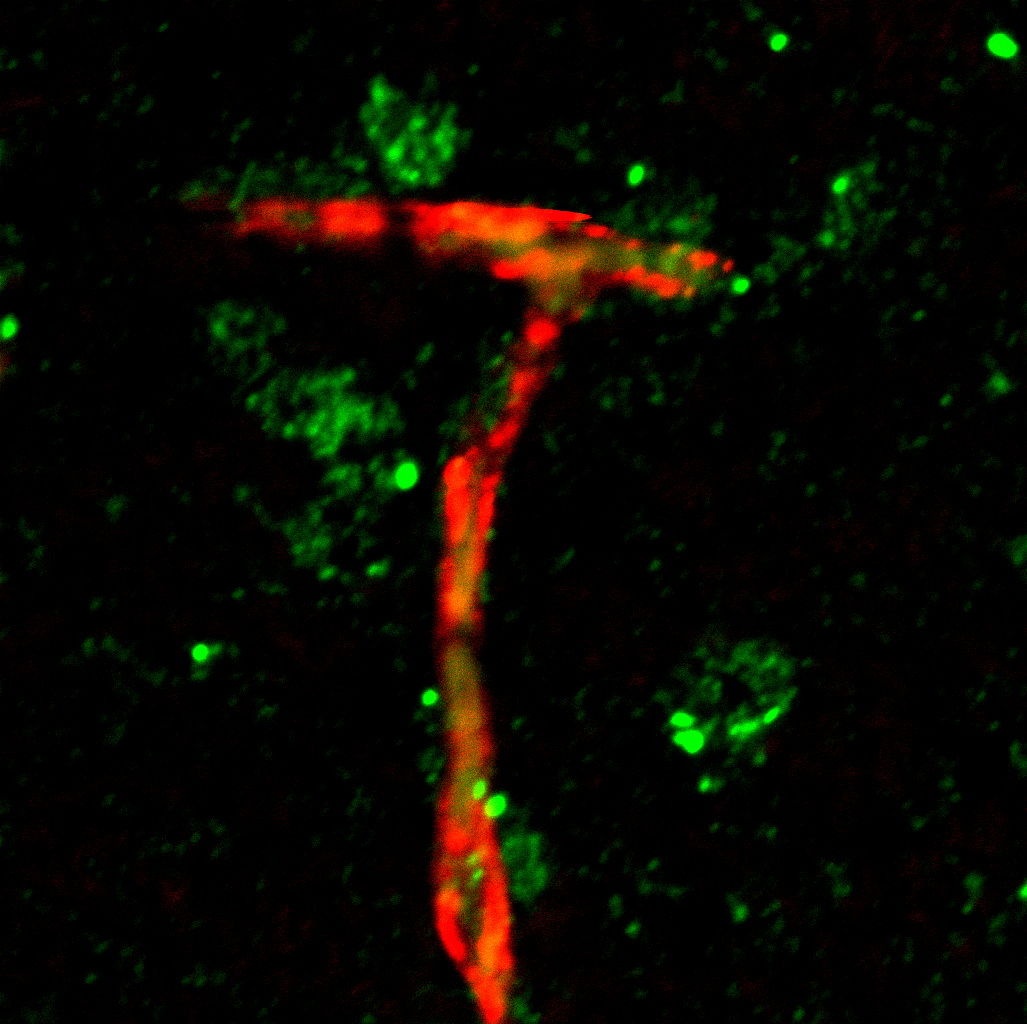

Supplement: Supplementary file 19 [file DataSheet7.ZIP › Immunofluorescence (Figure 6G, part2)/8/1/1s_c1+2.tif]

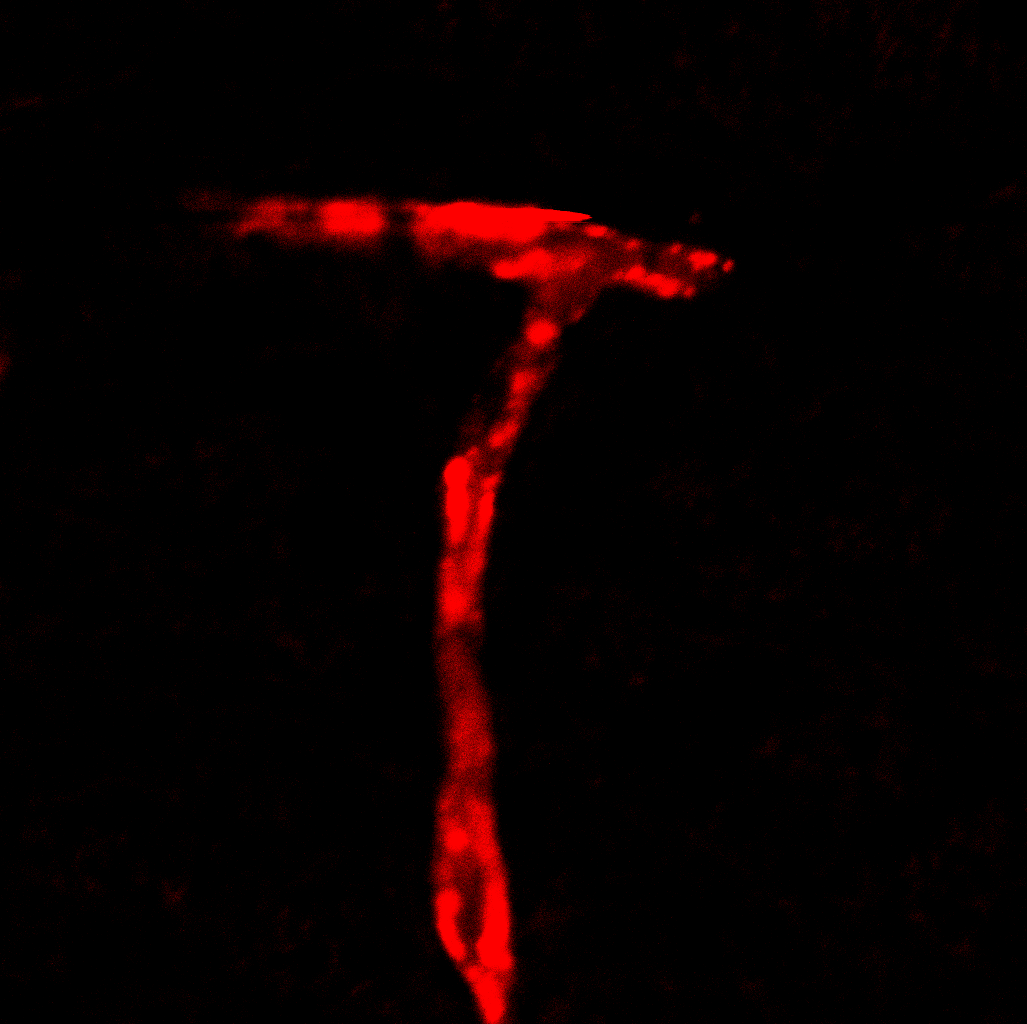

Supplement: Supplementary file 19 [file DataSheet7.ZIP › Immunofluorescence (Figure 6G, part2)/8/1/1s_c1.tif]

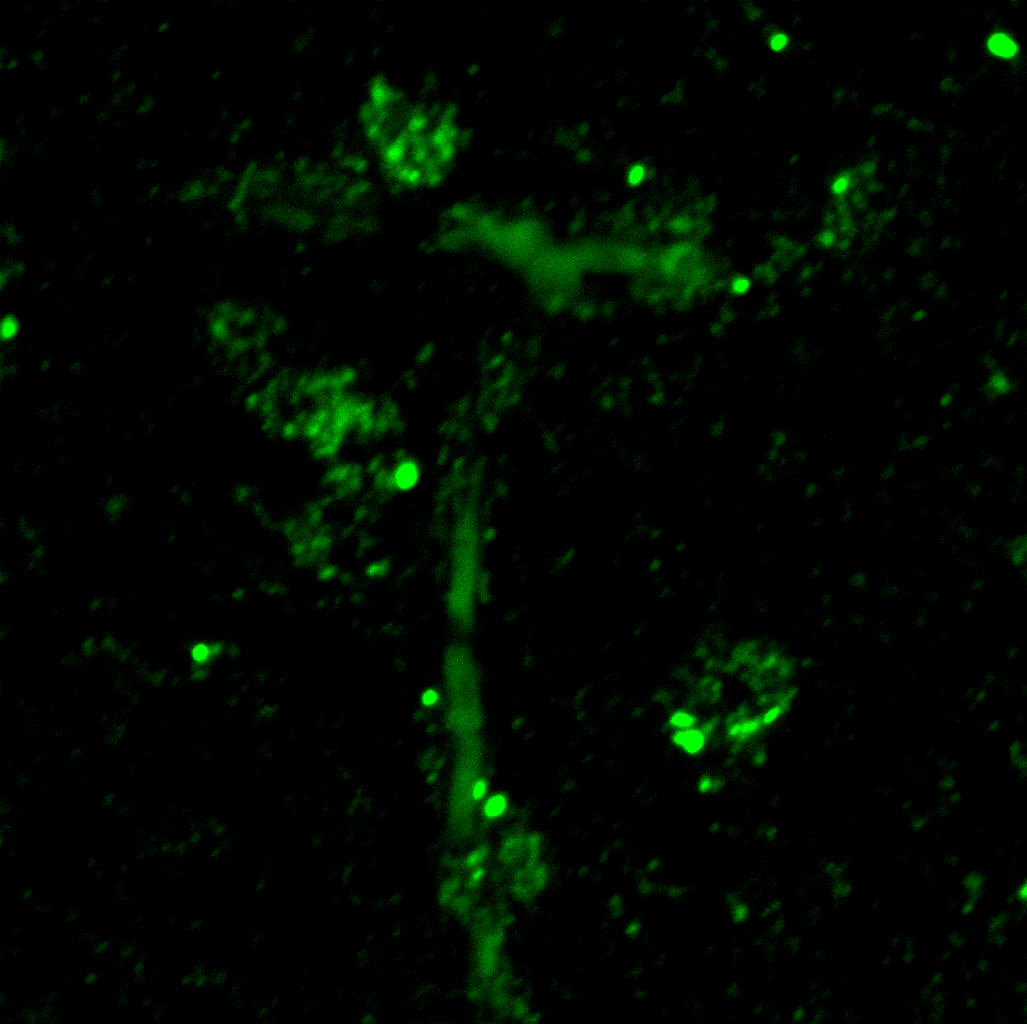

Supplement: Supplementary file 19 [file DataSheet7.ZIP › Immunofluorescence (Figure 6G, part2)/8/1/1s_c2.tif]

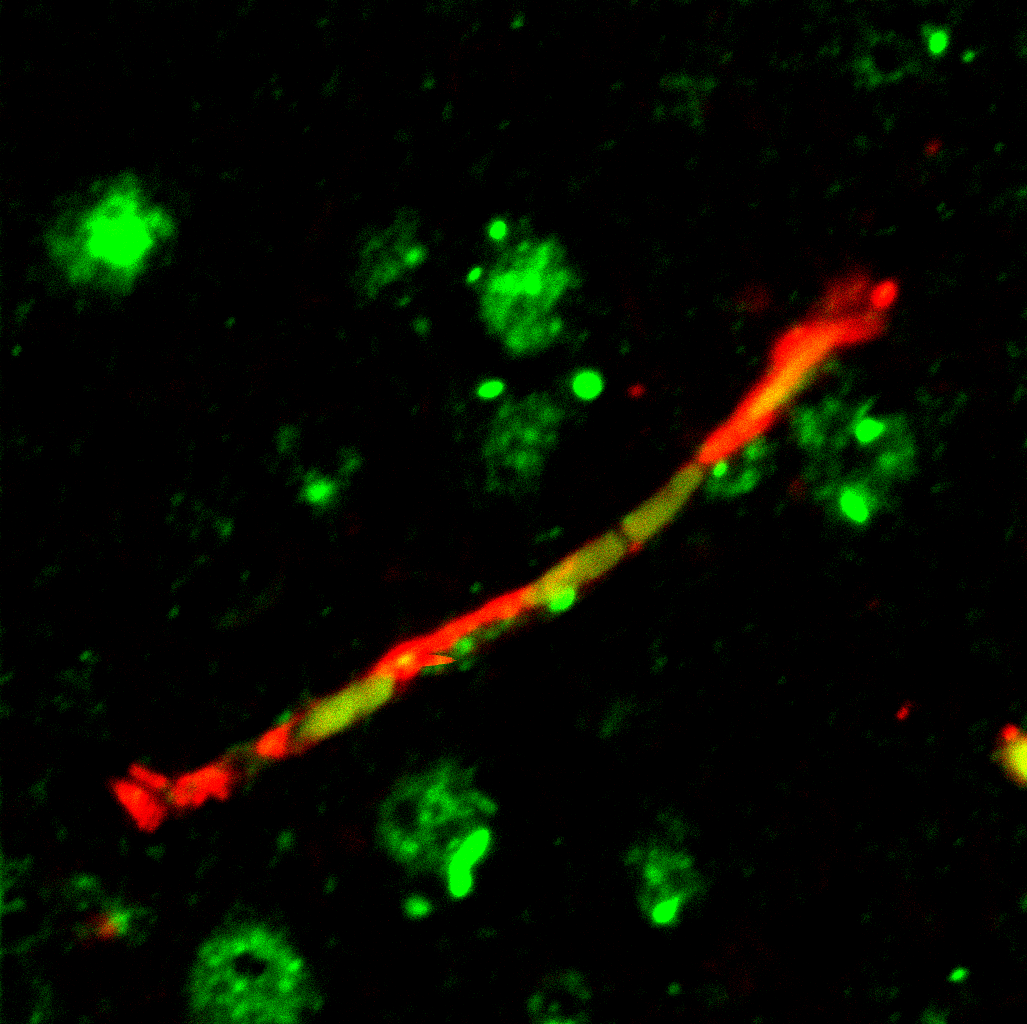

Supplement: Supplementary file 19 [file DataSheet7.ZIP › Immunofluorescence (Figure 6G, part2)/8/2/4s_c1+2.tif]

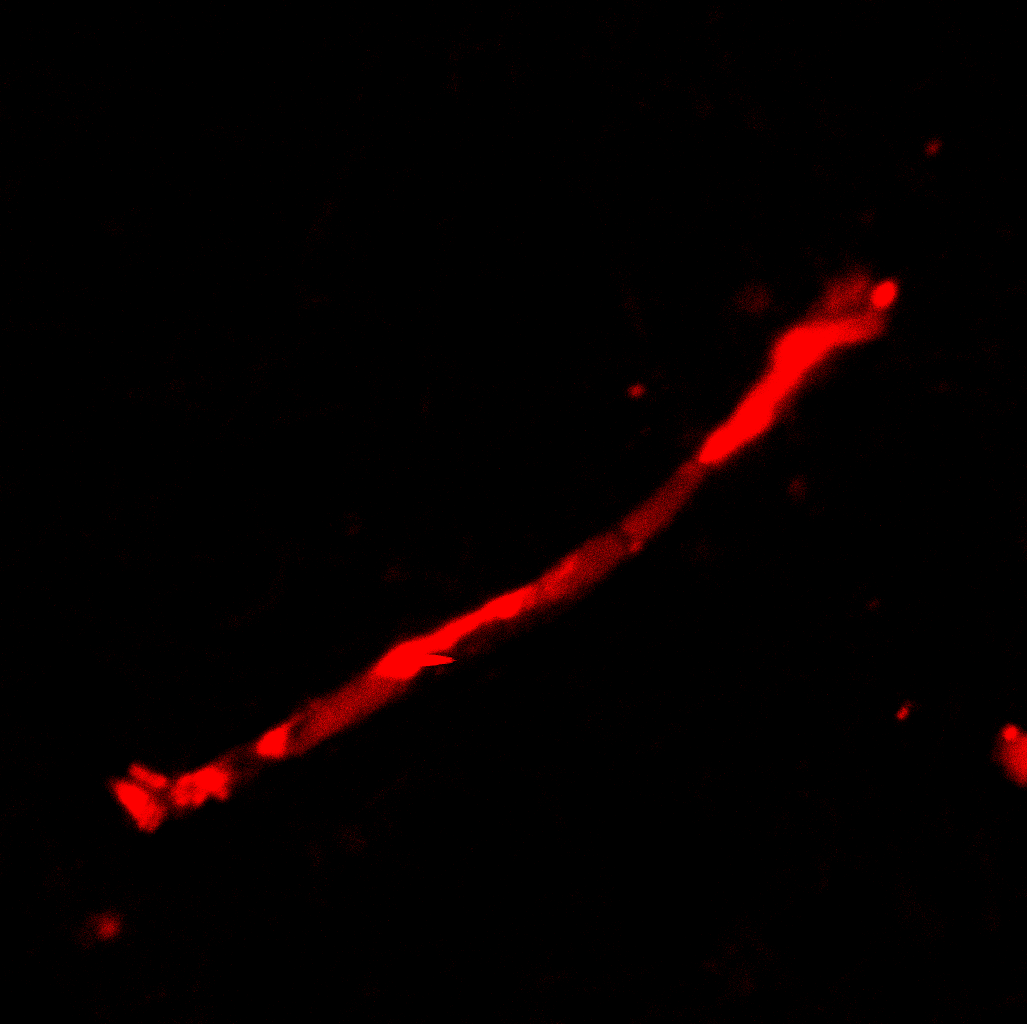

Supplement: Supplementary file 19 [file DataSheet7.ZIP › Immunofluorescence (Figure 6G, part2)/8/2/4s_c1.tif]

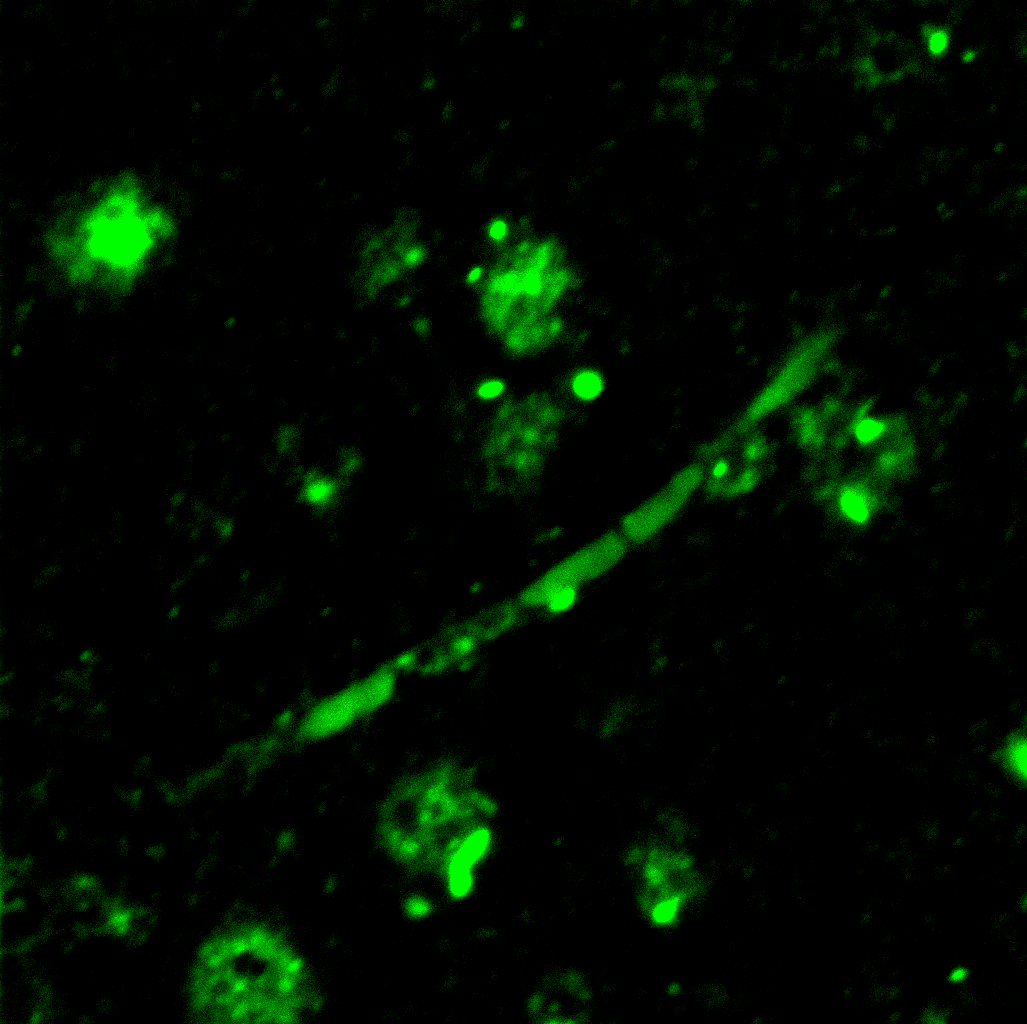

Supplement: Supplementary file 19 [file DataSheet7.ZIP › Immunofluorescence (Figure 6G, part2)/8/2/4s_c2.tif]

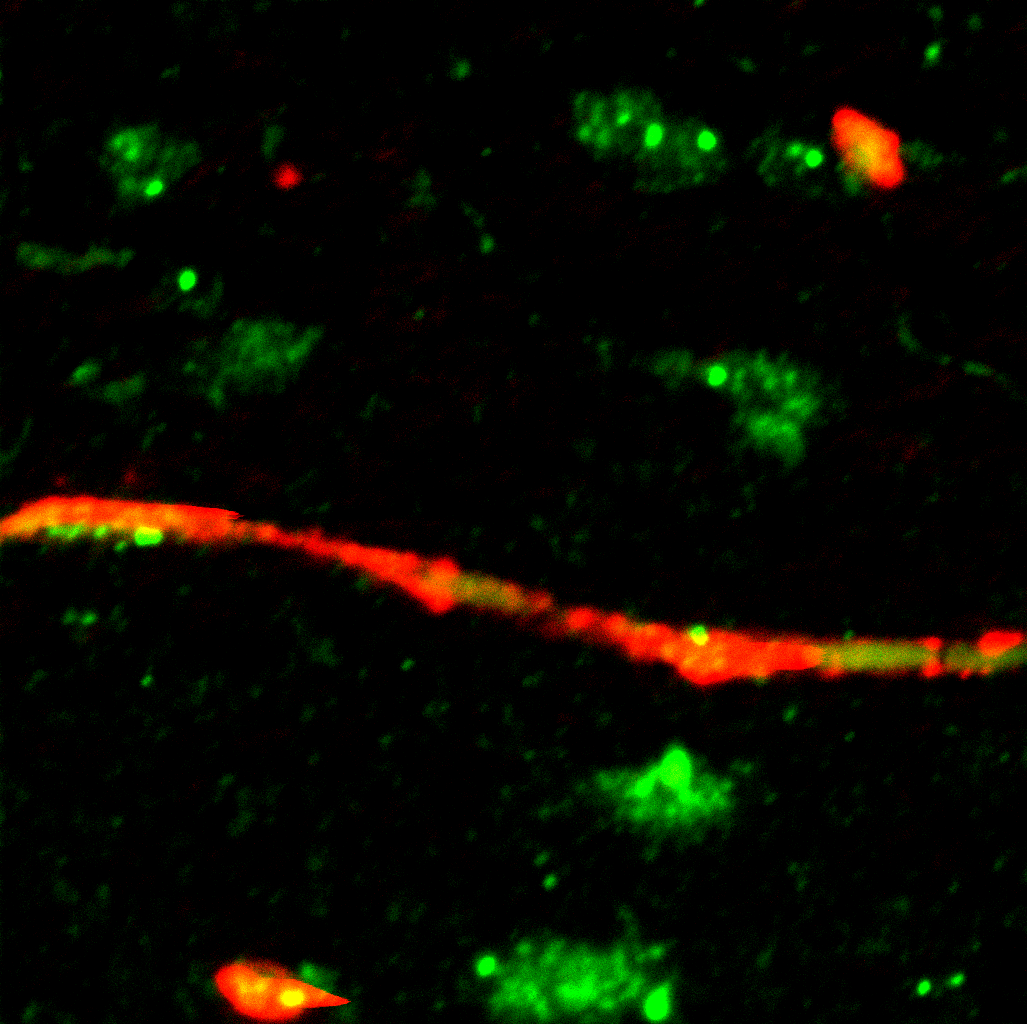

Supplement: Supplementary file 19 [file DataSheet7.ZIP › Immunofluorescence (Figure 6G, part2)/8/3/7s_c1+2.tif]

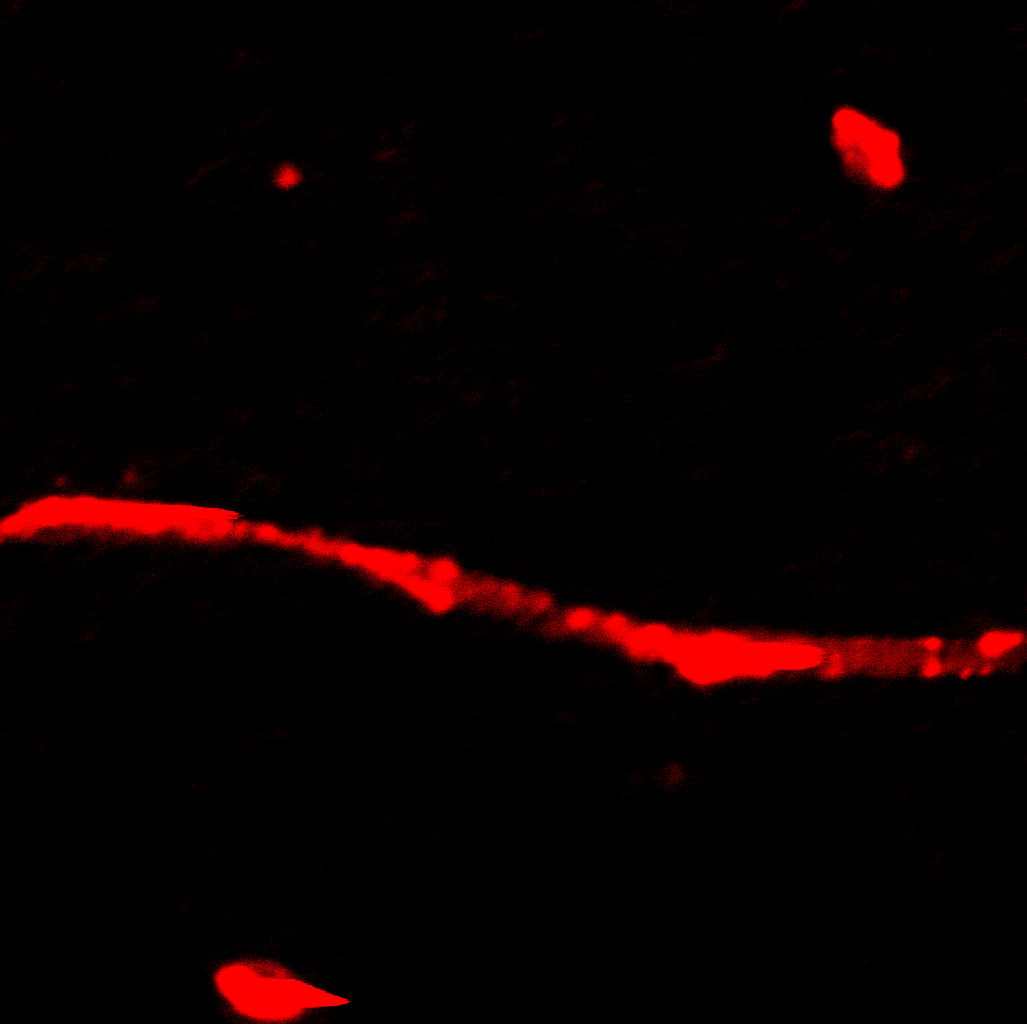

Supplement: Supplementary file 19 [file DataSheet7.ZIP › Immunofluorescence (Figure 6G, part2)/8/3/7s_c1.tif]

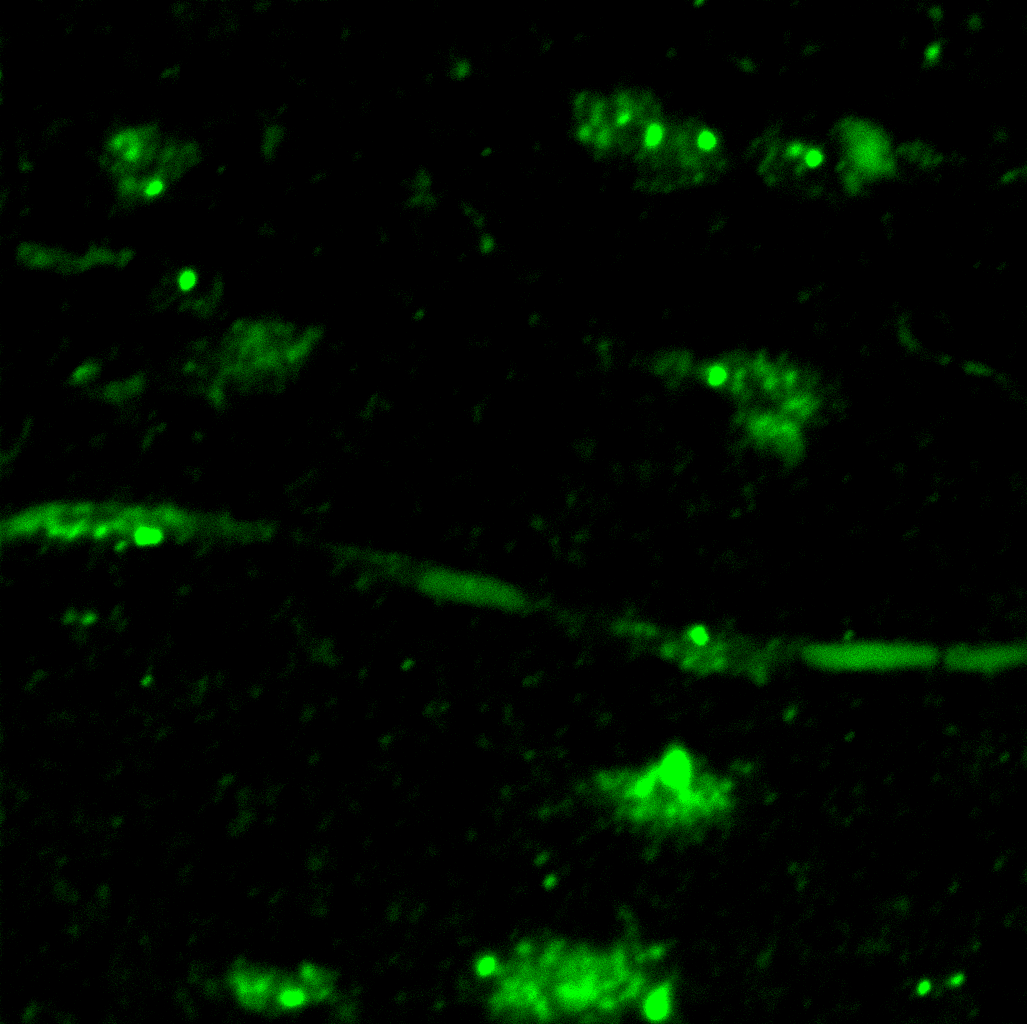

Supplement: Supplementary file 19 [file DataSheet7.ZIP › Immunofluorescence (Figure 6G, part2)/8/3/7s_c2.tif]

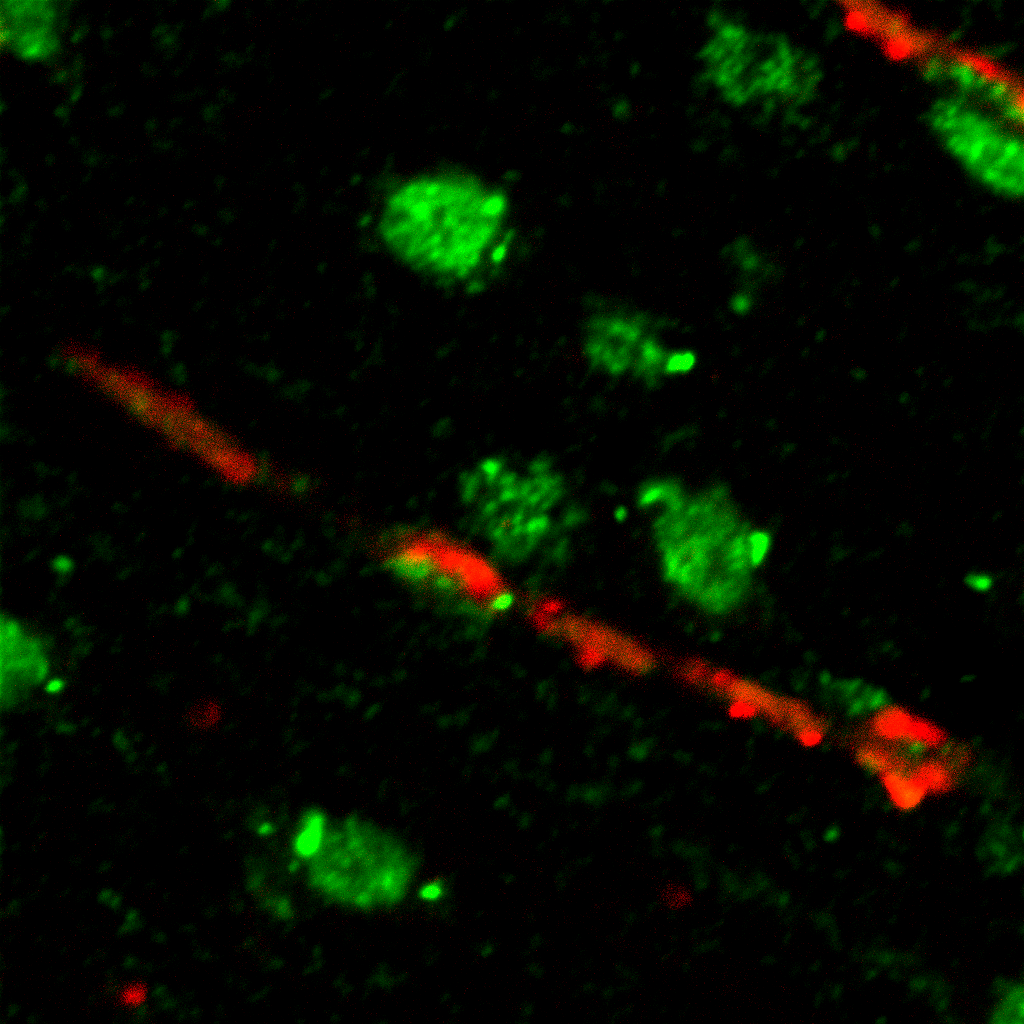

Supplement: Supplementary file 19 [file DataSheet7.ZIP › Immunofluorescence (Figure 6G, part2)/9/1/1ss_c1+2.tif]

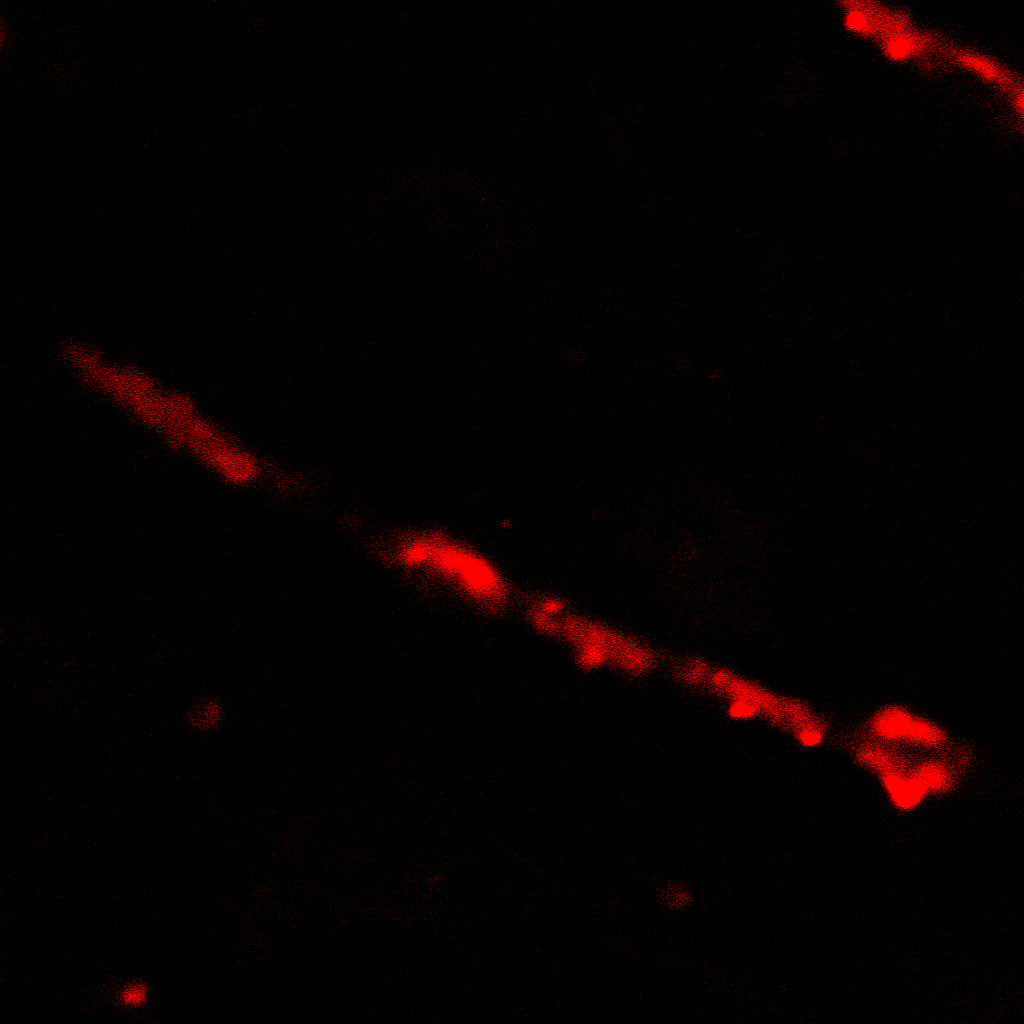

Supplement: Supplementary file 19 [file DataSheet7.ZIP › Immunofluorescence (Figure 6G, part2)/9/1/1ss_c1.tif]

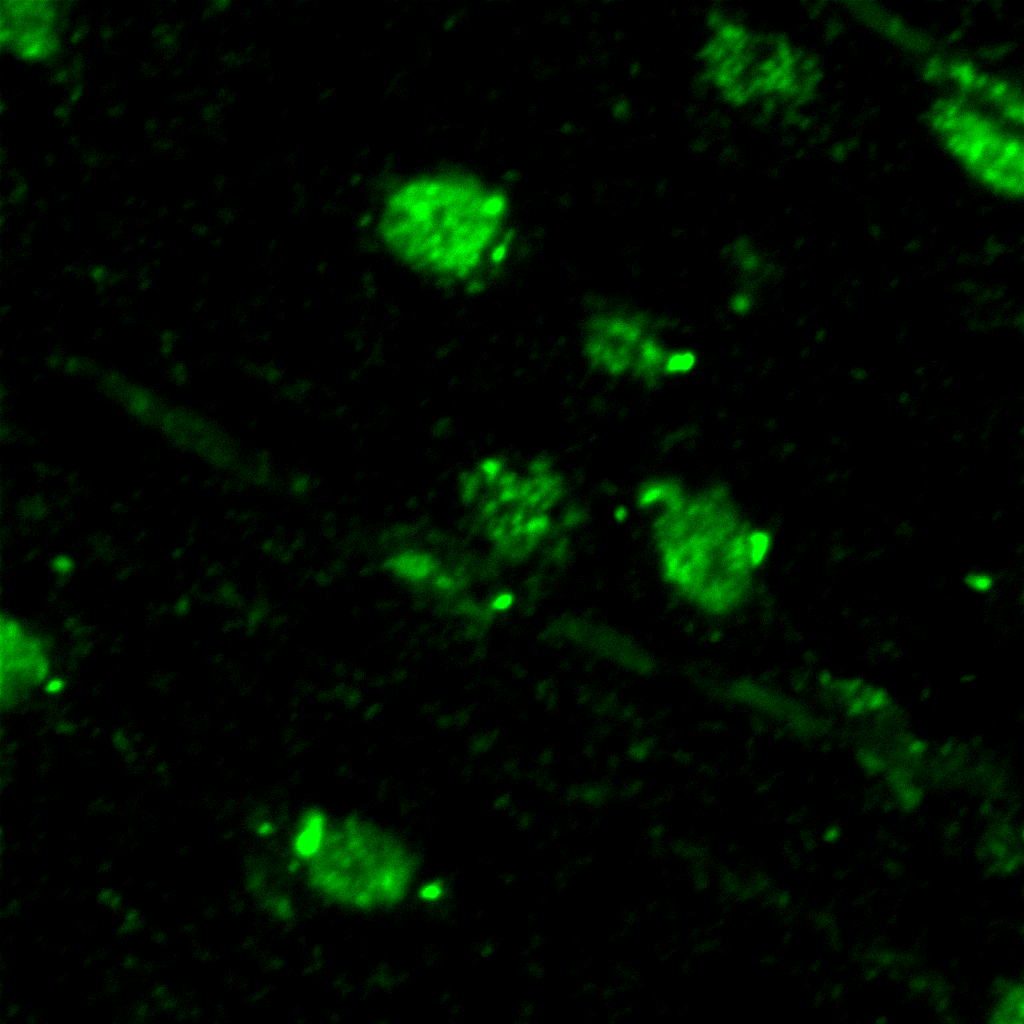

Supplement: Supplementary file 19 [file DataSheet7.ZIP › Immunofluorescence (Figure 6G, part2)/9/1/1ss_c2.tif]

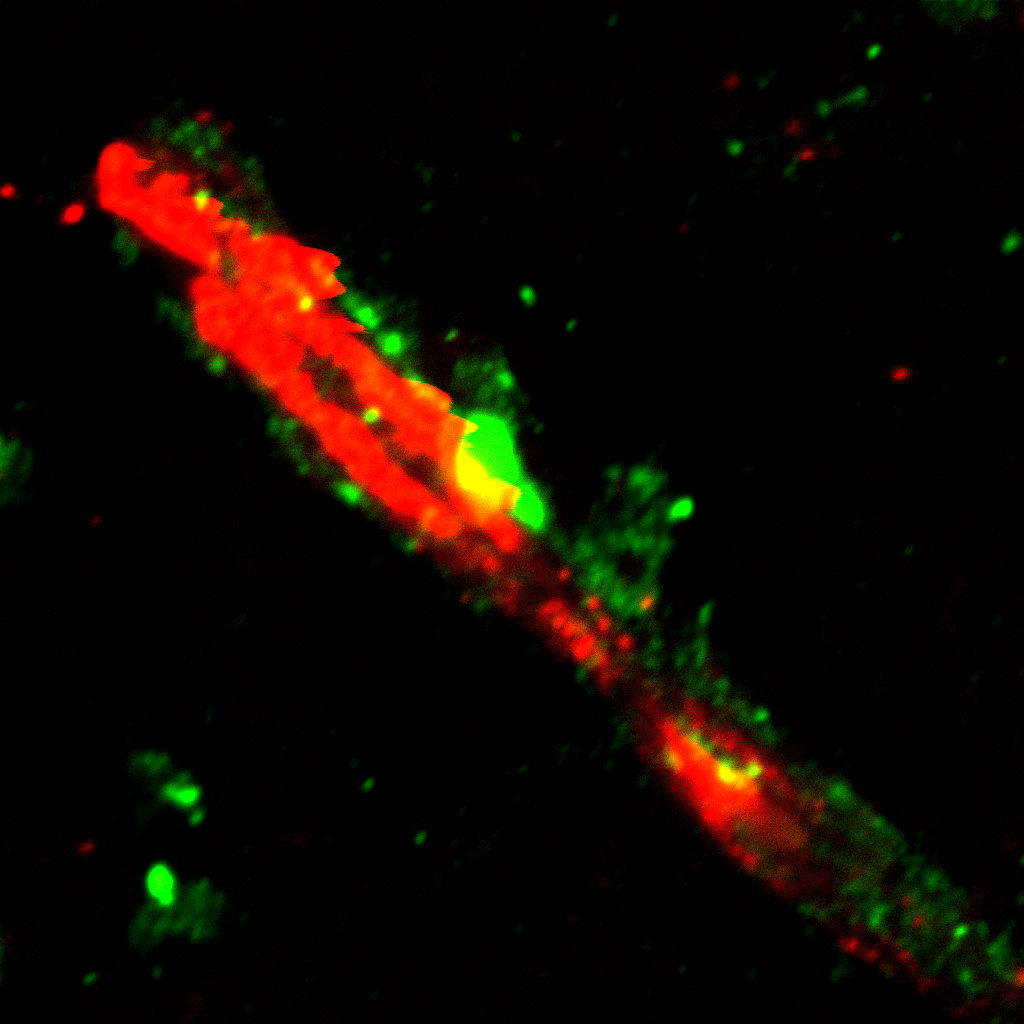

Supplement: Supplementary file 19 [file DataSheet7.ZIP › Immunofluorescence (Figure 6G, part2)/9/2/3ss_c1+2.tif]

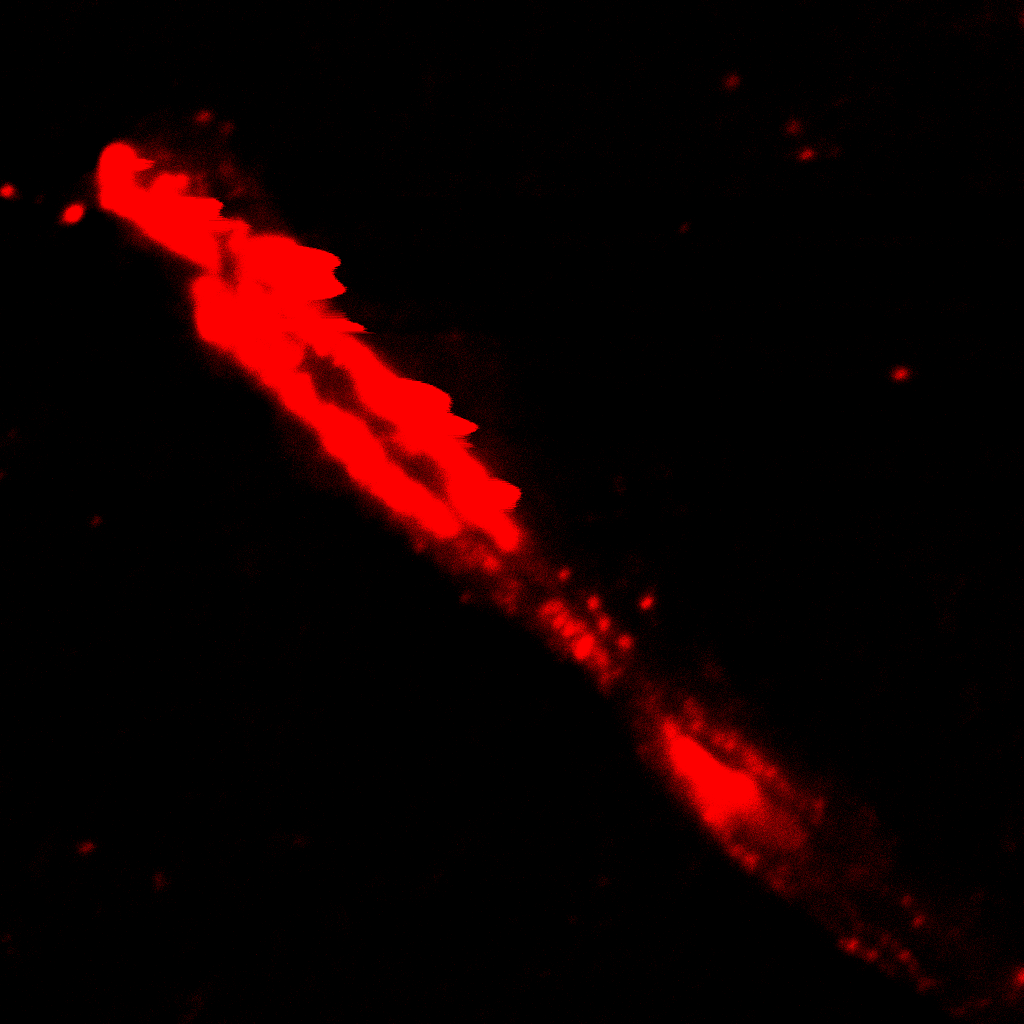

Supplement: Supplementary file 19 [file DataSheet7.ZIP › Immunofluorescence (Figure 6G, part2)/9/2/3ss_c1.tif]

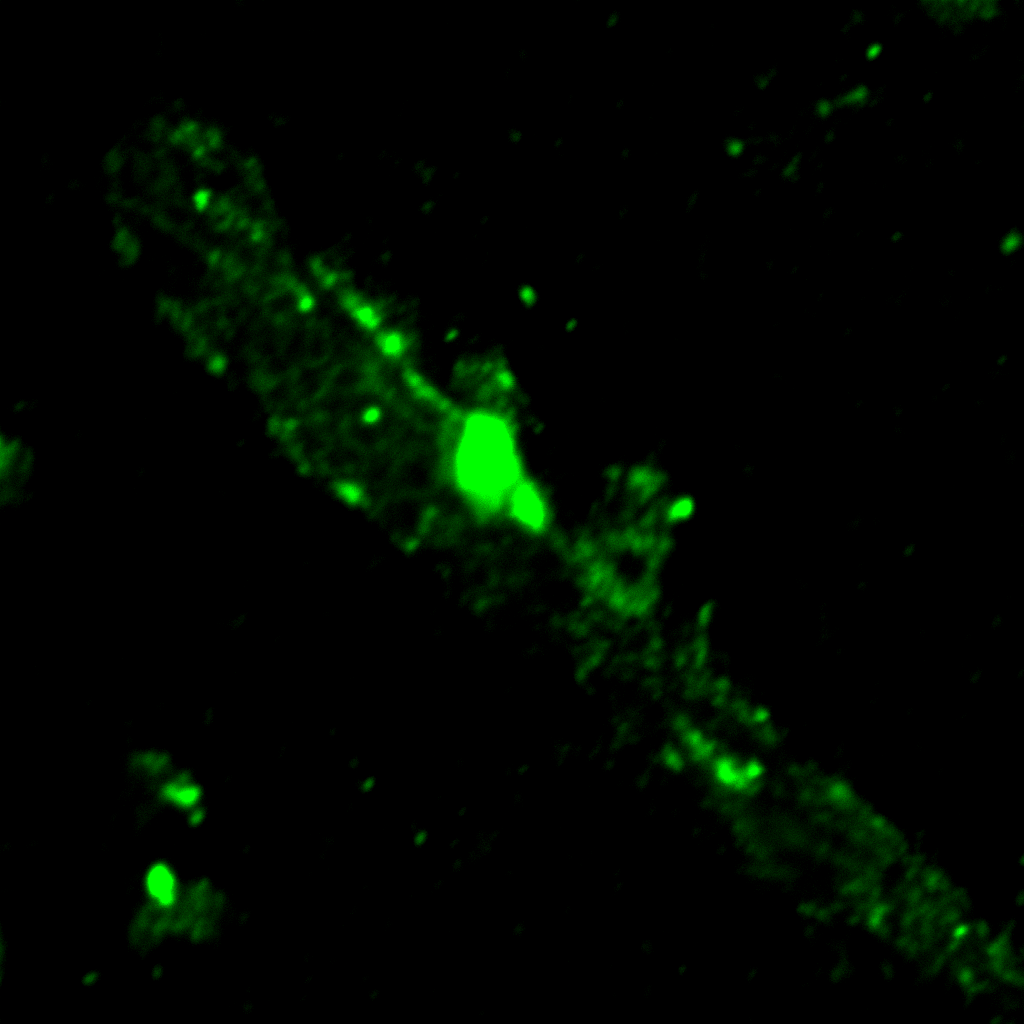

Supplement: Supplementary file 19 [file DataSheet7.ZIP › Immunofluorescence (Figure 6G, part2)/9/2/3ss_c2.tif]

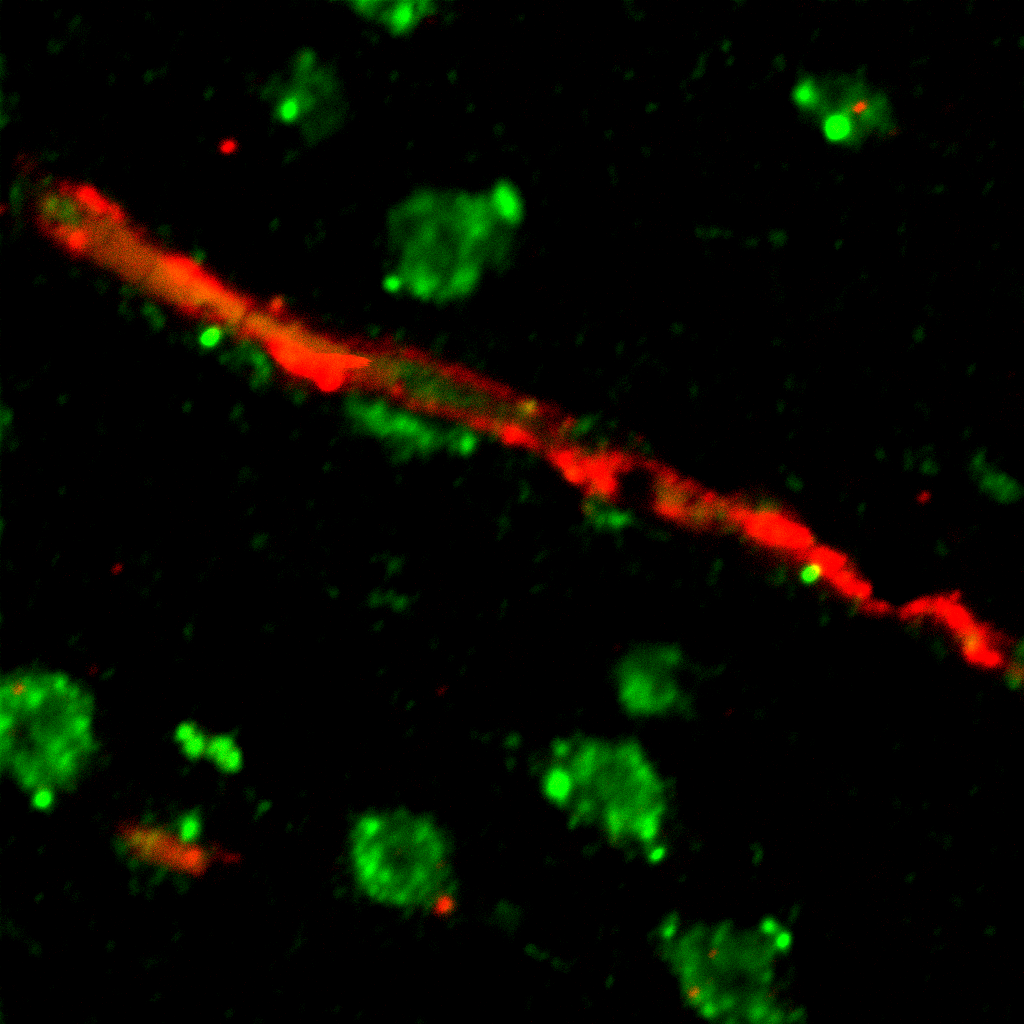

Supplement: Supplementary file 19 [file DataSheet7.ZIP › Immunofluorescence (Figure 6G, part2)/9/3/6ss_c1+2.tif]

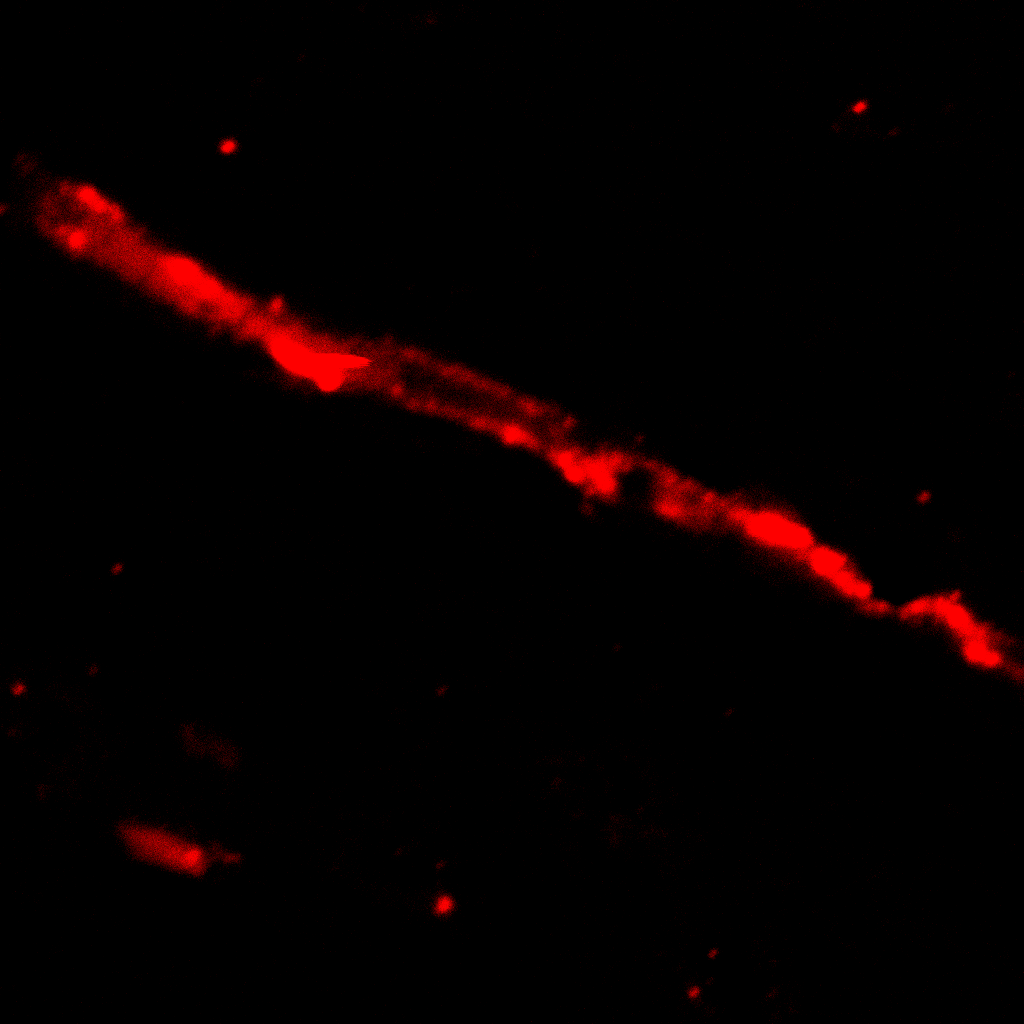

Supplement: Supplementary file 19 [file DataSheet7.ZIP › Immunofluorescence (Figure 6G, part2)/9/3/6ss_c1.tif]

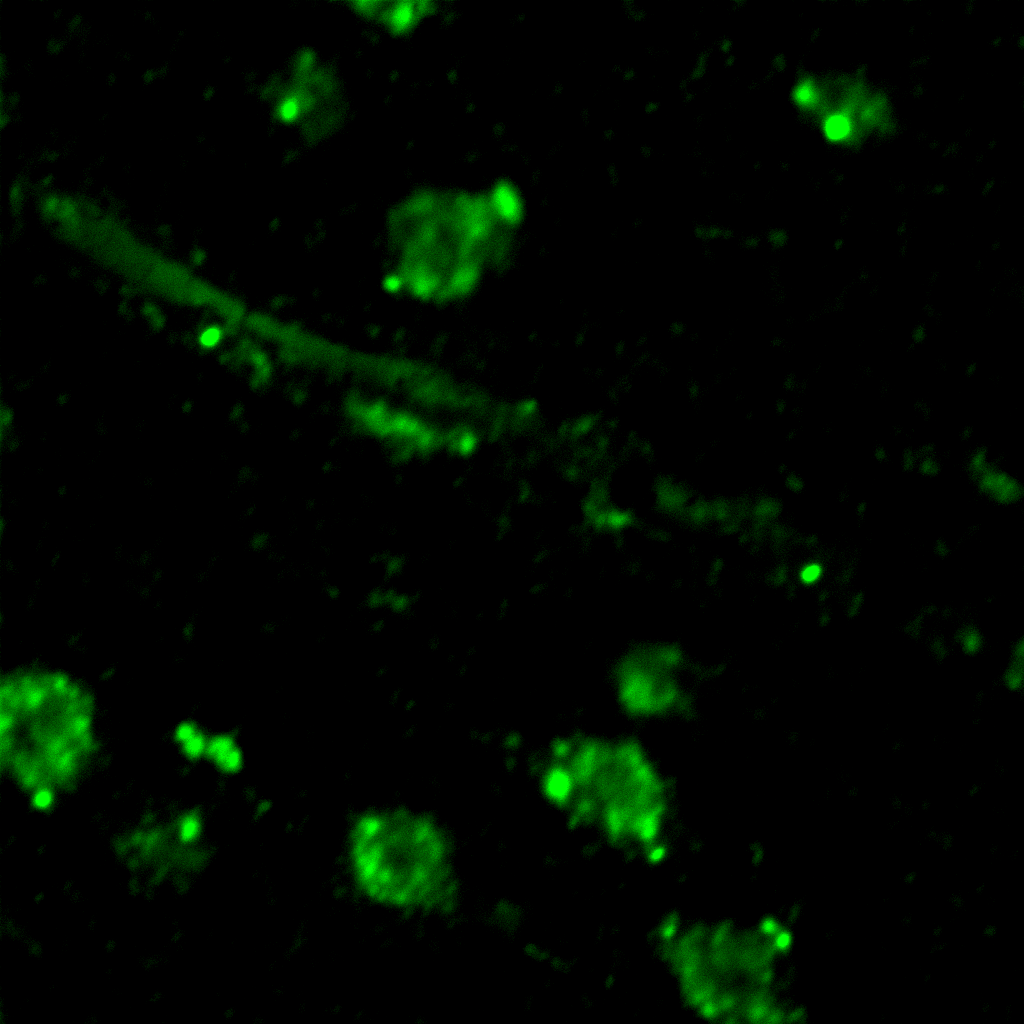

Supplement: Supplementary file 19 [file DataSheet7.ZIP › Immunofluorescence (Figure 6G, part2)/9/3/6ss_c2.tif]
